# Long Mu Qing Xin mixture improves behavioral performance in spontaneously hypertensive rats (SHR/NCrl) by upregulating catecholamine neurotransmitters in prefrontal cortex and striatum via DRD1/cAMP/PKA-CREB signaling pathway

**DOI:** 10.3389/fphar.2024.1387359

**Published:** 2024-07-04

**Authors:** Xuejun Li, Zhen Xiao, Zhiyan Jiang, Wenyan Pu, Xiufeng Chen, Shumin Wang, Anqi Liu, Hongyu Zhang, Zihao Xu

**Affiliations:** ^1^ Pediatrics, Longhua Hospital Affiliated to Shanghai University of Traditional Chinese Medicine, Shanghai, China; ^2^ Longhua Clinical Medical College, Shanghai University of Traditional Chinese Medicine, Shanghai, China

**Keywords:** long Mu Qing Xin Mixture, attention deficit hyperactivity disorder, DRD1/cAMP/PKA-CREB signaling pathway, catecholamine neurotransmitters, behavioral manifestations, spontaneously hypertensive rats

## Abstract

**Background:**

Attention deficit hyperactivity disorder (ADHD), a prevalent neurodevelopmental disorder in children, can be effectively alleviated by the herbal preparation Long Mu Qing Xin Mixture (LMQXM), but its mechanism has not been fully elucidated.

**Objective:**

To scrutinize the potential pharmacological mechanisms by which LMQXM improves behavior in spontaneously hypertensive rats (SHR/NCrl).

**Methods:**

The SHR/NCrl rats were randomly stratified into the model (SHR) group, the methylphenidate hydrochloride (MPH) group, and groups subjected to varying dosages of LMQXM into the medium dose (MD) group with a clinically effective dose, the low dose (LD) group with 0.5 times the clinically effective dose, and high dose (HD) group with 2 times the clinically effective dose. Furthermore, the WKY/NCrl rats constituted the control group. The evaluation of behavior involved the open field test and the Morris water maze test. HPLC, LC-MS, ELISA, immunohistochemistry, Western blot, and RT-qPCR were utilized to scrutinize the catecholamine neurotransmitter content and the expression of proteins and genes associated with the dopamine receptor D1 (DRD1)/cAMP/protein kinase A (PKA)-cAMP response element-binding (CREB) pathway in prefrontal cortex (PFC) and striatum.

**Results:**

MPH and LMQXM ameliorated hyperactivity and learning and memory deficits of SHR/NCrl rats. Among them, LMQXM-MD and MPH also upregulated dopamine (DA), norepinephrine (NE), adenylate cyclase (AC) and cAMP levels, and the expression of proteins and genes associated with the DRD1/cAMP/PKA-CREB pathway in PFC and striatum of SHR/NCrl rats. PFC and striatum DA levels were also upregulated in the LMQXM-LD group as well as the striatum DA levels in the LMQXM-HD group, but there were no statistically significant differences in their NE levels compared to the SHR group. LMQXM-LD and LMQXM-HD also upregulated some DRD1/cAMP/PKA-CREB pathway-related proteins and gene expression, but the effects were discernibly disparate in PFC and striatum. Upon comprehensive analysis, LMQXM-MD appeared to be the most effective dose.

**Conclusion:**

Our study tentatively suggests that LMQXM may rectify hyperactivity and learning and memory deficits of SHR/NCrl rats by elevating catecholamine neurotransmitters in the PFC and striatum. This effect may be attributed to the potential activation of the DRD1/cAMP/PKA-CREB signaling pathway, which appears to achieve an optimal response at moderate doses.

## 1 Introduction

Attention Deficit Hyperactivity Disorder (ADHD) stands as the most prevalent, enduring neurodevelopmental condition in childhood, characterised by inattention, hyperactivity, and impulsivity. ADHD shows an estimated prevalence ranging from 5.0% to 7.1% (Gallo and Posner, 2026) and has a higher incidence in boys (Kelly, 2018). Notably, 80% of affected children concurrently present with one or more comorbidities, including intellectual disability, mood disorders, sleep disturbances, and Tourette’s disorder (Naguy, 2017). These comorbidities persist across the lifespan, profoundly affect personal development, career progression, social stability, also imposing a substantial burden on families and the national economy. Given the multifaceted and strongly heritable nature of ADHD, around 90% of children with ADHD eventually resort to pharmacological interventions (Caye et al., 2019). Methylphenidate hydrochloride (MPH) ameliorates the executive function deficits in ADHD by elevating central dopamine (DA) and norepinephrine (NE) activity (Mechler et al., 2022). However, it is accompanied by adverse effects such as induced anxiety, depression, appetite loss, sleep disturbances, and a potential constraint on height growth (approximately 1–3 cm) (Rapport and Moffitt, 2002). Moreover, non-pharmacological treatments lack substantial support from evidence-based medicine (Nimmo-Smith et al., 2020; Posner et al., 2020). Consequently, there arises an urgent need for the development of novel, safe, and efficacious therapies to meet the pressing clinical requirements.

Deficiencies in catecholamine neurotransmitters, specifically DA and NE, represent a central element in the pathogenesis of ADHD (Oades, 1987). The hydroxylation of DA is instrumental in the generation of NE, and both neurotransmitters play a pivotal role in executive function through their neuromodulatory impact on the frontal-striato-cerebellar circuits (Del Campo et al., 2011). Lower concentrations of DA and NE have been detected in the serum of children with ADHD, which may indirectly mirror a disrupted state of central catecholaminergic neurotransmission (Xiong et al., 2021). Rectifying this aberrant catecholaminergic neurotransmission stands as a critical objective in the treatment of ADHD. DA’s executive function is mediated by the cyclic adenosine monophosphate (cAMP)/protein kinase A (PKA) signaling pathway. DA is coupled to the dopamine D1 receptor (DRD1), and through the G-protein subunits of DRD1, mainly the Gαs and Gαolf isoforms, it can catalyze the hydrolysis of ATP to cAMP by adenylate cyclase (AC), which initiates a cascade of reactions in the cAMP/PKA signaling pathway. (Nishi and Snyder, 2010; Sun et al., 2017; Gao et al., 2022). This process regulates the expression and transcription of cAMP response element-binding protein (CREB) and brain-derived neurotrophic factor (BDNF), thereby participating in learning and memory formation, neuronal development, synaptic plasticity, and neuroprotection (Wang et al., 2018; Cicek et al., 2022). The influence of the cAMP/PKA-CREB signaling cascade initiated by DA binding to DRD1 on the synthesis of catecholaminergic neurotransmitters and brain executive functions has emerged as a prominent focus in the study of ADHD pathophysiology. Mitigating the core symptoms of ADHD by modulating the DRD1/cAMP/PKA-CREB pathway presents a viable approach to its treatment.

Long Mu Qing Xin Mixture (LMQXM) is a traditional Chinese medicinal preparation designed for ADHD. It comprises of radix astragali, radix angelicae sinensis, ramulus uncariae cum uncis, fructus jujubae, radix paeoniae alba, fructus schisandrae, radix scutellariae, cortex phellodendri, calcined dragon bone, calcined oyster shell, concha margaritifera usta, magnetitum, mix-fried licorice, light wheat, and caulis polygoni multiflori. Our prior investigations (Chen and Xiao, 2016) have substantiated LMQXM’s efficacy in alleviating hyperactivity, impulsivity, and learning challenges in children afflicted by ADHD. Network pharmacology analyses have proposed the cAMP signalling pathway as a probable key avenue through which LMQXM exerts its therapeutic action in ADHD. Furthermore, pharmacological inquiries have corroborated LMQXM’s ability to ameliorate the fundamental symptoms of ADHD by elevating dopamine (DA) levels within the prefrontal cortex (PFC) and striatum of spontaneously hypertensive rats (SHRs) (Li et al., 2023). However, the specific mechanism underlying LMQXM’s influence on catecholamine neurotransmitters necessitates further elucidation.

**FIGURE 1:**
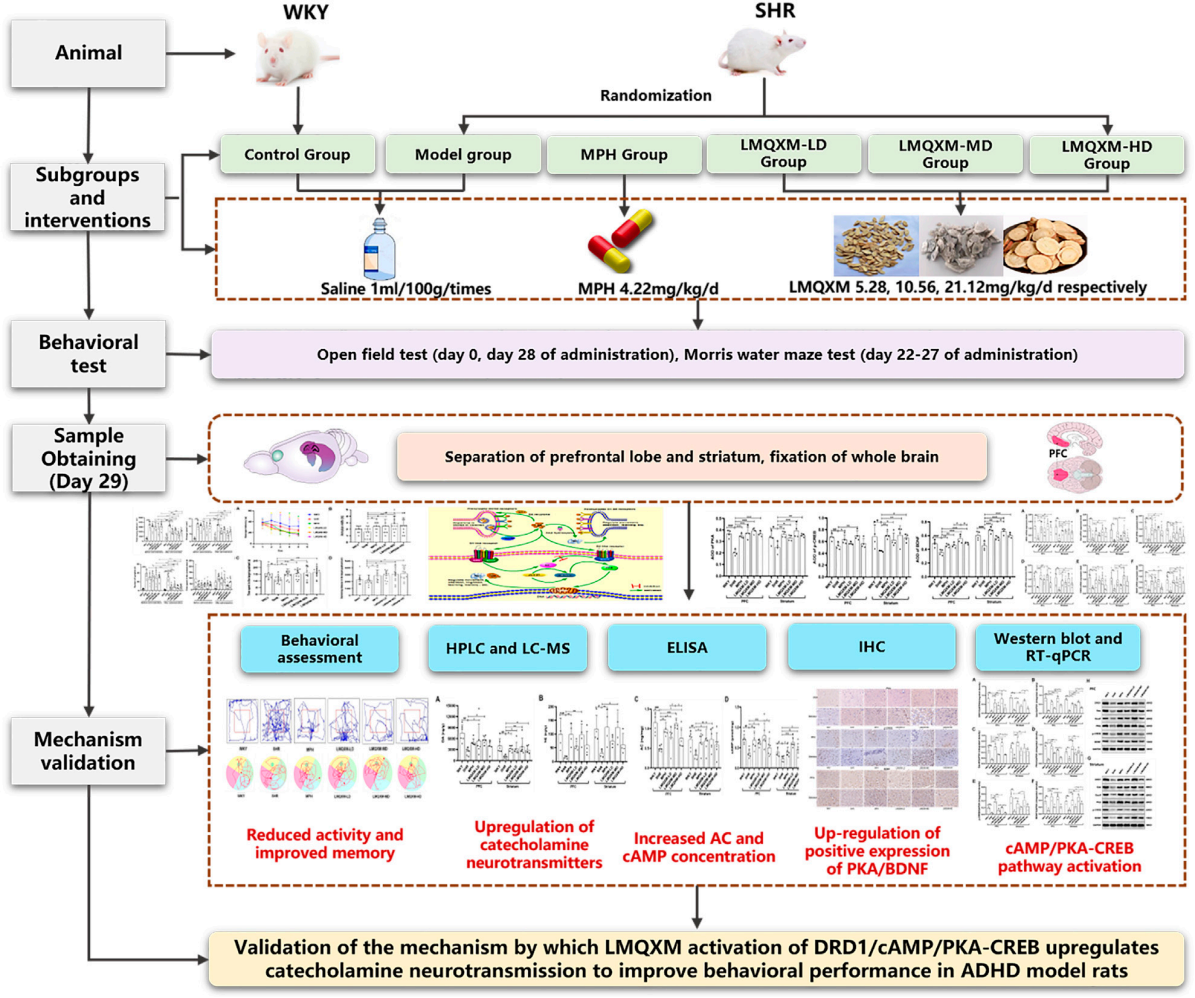
Graphical abstract.

In the scope of this study, we will employ juvenile SHR/NCrl rats from Charles River, a well-acknowledged animal model for ADHD that best represents the combined subtype (Miller et al., 2012), to scrutinise the impact of LMQXM on the behavioural profile of SHRs. Young SHR/NCrl rats can mimic the ADHD-typical behaviors that closely resemble childhood symptoms of ADHD (Meneses et al., 2011), and also exhibit reduced ATP-producing capacity and reduced neurotransmitter content, which best fits the surface, structure, and predictive validity of animal models of ADHD (Dupuy et al., 2021). In accordance with established practice, we designated Charles River WKY/NCrl rats as natural controls for SHR/NCrl rats (Thanos et al., 2010). Our objective is to appraise whether LMQXM’s elevation of catecholaminergic neurotransmission is orchestrated via the DRD1/cAMP/PKA and CREB signalling pathway. Visual representations summarising our findings are furnished in Figure 1.

## 2 Materials and methods

### 2.1 Experimental animals

Specific pathogen-free (SPF) grade four-week-old male SHR/NCrl rats (Charles River, Germany, n = 60) and WKY/NCrl rats (Charles River, Germany, n = 12) were procured from Beijing Vital River Laboratory Animal Technology Co. Ltd (Beijing, China, SCXK (Jing) 2021-0006). Male rats were specified because ADHD shows a higher prevalence in male children (Mourek and Pokorný, 2022). These animals were housed in the Laboratory Animal Center of Shanghai University of Traditional Chinese Medicine under controlled conditions: temperature set at 23°C ± 2°C, relative humidity maintained between 50% and 60%, with a 12-h light/dark cycle and *ad libitum* access to food and water. All experimental procedures strictly adhered to the guidelines of the National Institute of Laboratory Animal Health of China and internationally recognised principles governing the care and use of laboratory animals. Furthermore, the Laboratory Animal Ethics Committee of Shanghai University of Traditional Chinese Medicine approved the study on 17 October 2022 (No. PZSHUTCM221017003).

### 2.2 Drug preparation and composition analysis of LMQXM

LMQXM (batch No. 2206001, Shanghai Pharmaceutical Preparation Code: Z05170218) was purchased from Longhua Hospital affiliated with Shanghai University of Traditional Chinese Medicine. Table 1 provides the quantitative information of all the constituent Chinese medicines of LMQXM, and the herbal mixture was into concentrated to a concentration of 4.17 g/mL via a rotary evaporator before the experiment. We analyzed about 40 chemical components of LMQXM by ultra-high-performance liquid chromatography-quadrupole time-of-flight tandem mass spectrometry (UPLC-Q-TOF-MS), and the qualitative analysis about the active ingredients of LMQXM can be found in Supplementary Table S1.

**TABLE 1:** Quantitative information of LMQXM constituent herbs.

Chinese medicine	Chinese name	Content (mg/mL)
Radix astragali	Huang Qi	225
Radix angelicae sinensis	Dang Gui	135
Ramulus uncariae cum uncis	Gou Teng	225
Fructus jujubae	Da Zao	450
Radix paeoniae alba	Bai Shao	450
Fructus schisandrae	Wu Wei Zi	84
Radix scutellariae	Huang Qin	135
Cortex phellodendri	Huang Bai	135
Calcined dragon bone	Duan-Long Gu	450
Calcined oyster shell	Duan-Mu Li	450
Concha margaritifera usta	Zhen Zhu Mu	450
Magnetitum	Ci Shi	450
Mix-fried licorice	Zhi Gan Cao	84
Light wheat	Fu Xiao Mai	225
Caulis polygoni multiflori	Shou Wu Teng	225

### 2.3 Chemicals and reagents

Methylphenidate hydrochloride extended-release tablets, batch No. 1KE744, were sourced from Xi’an Janssen Pharmaceutical Co Ltd (Xi’an, China). Primary antibodies, specifically DRD1 (ab81296), BDNF (ab108319), and PKA (ab75991), were acquired from Abcam (Cambridge, UK), whereas phospho-CREB (9198S) and HRP-labelled goat anti-rabbit IgG (7074P2) were procured from Cell Signaling Technology (Boston, USA). Gαolf (DF4108) and GAPDH (AF7021) were sourced from Affinity (Texas, USA), and Gαs (10150-2-AP) was obtained from proteintech (Wuhan, China). Furthermore, the First Strand cDNA synthesis Kit (K1622) was purchased from Invitrogen (California, USA), while the QuantityNova SYBR Green PCR Kit (208,054) was procured from QIAGEN (Düsseldorf, Germany).

### 2.4 Drug interventions

Following a 3-day acclimatisation period in a controlled environment, SHR/NCrl rats were randomly allocated to distinct groups: the model group (SHR), the methylphenidate hydrochloride group (MPH, 4.22 mg/kg/d), the LMQXM low-dose group (LMQXM-LD), the LMQXM medium-dose group (LMQXM-MD), and the LMQXM high-dose group (LMQXM-HD). Concurrently, the WKY/NCrl rats were designated as the normal control group. The clinically effective dose of LMQXM for children was 75 mL/d, which was concentrated to three-fifths of the original volume, i.e., 45 mL/d, prior to the experiment, taking into account the limitations of the animals’ gavage volume. The dose administered to the animals was based on the body surface area of a 9-year-old (body mass 26 kg) child (0.991 m^2^, Stevenson’s formula = 0.0061×height (cm)+0.0128×body weight (kg)-0.1529) and 4-week-old (body mass 60 g) SHR pups body surface area (0.013947 m^2^, Meeh-Rubner formula = 9.1 × body weight (g)⅔/10,000) were converted in parallel (Song et al., 2021; Li et al., 2023). The clinically equivalent dose of SHR/NCrl rats was calculated to be 10.56 mL/kg/d. The LMQXM groups were administered with a clinically equivalent dose as the medium dose (10.56 mL/kg/d), 0.5 times the clinically equivalent dose (5.28 mL/kg/d) as the low dose, and 2 times the clinically equivalent dose (21.12 mL/kg/d) as the high dose, in two doses (8:00-9:00a m; 14:00-15:00 p.m.), with a volume of gavage of 1 mL/100 g/times; the control group and the model group were given equal volume of saline gavage; the duration of administration lasted for a total of 28 days. Previously, our clinical studies have shown that clinical doses of LMQXM have been applied without significant adverse effects (Chen and Xiao, 2016), and in the present experiments, high doses of gavage may irritate the gastric mucosa in animals.

### 2.5 Behavioral testing

#### 2.5.1 Open field test (OFT)

The open field test (OFT) was conducted both prior to and post-treatment (Yuan et al., 2019). The test setup consisted of a black square arena (100 cm × 100 cm × 48 cm), a camera system, a peripheral stand compatible with the dimensions of the arena, a computer, and specialised software for the analysis of animal behaviour. Animals underwent an 1 hour acclimatisation within the behavioural laboratory prior to the commencement of the test. Within the open field arena, a central region measuring 50 cm × 50 cm was defined as the focal area from which the rats were placed. A camera system tracked and recorded the animals’ movement trajectories. Subsequent analysis of the recorded data was conducted utilising ImageJ and MATLAB. Parameters examined included the total distance travelled, average speed, time spent within the central area, and the number of upright postures, serving as an indicator of the animals’ levels of spontaneous activity, all measured over a 5-min interval.

#### 2.5.2 Morris water maze (MWM) test

The MWM assessments were conducted on the 22nd day of the treatment regimen, spanning six consecutive days (Yuan et al., 2019). The MWM apparatus comprised a black circular pool (150 cm in diameter and 50 cm in depth), equipped with a camera and a computerised behavioural tracking system. The pool was filled with an appropriate volume of water, ensuring that the liquid level remained below the spatial control marker on the pool wall, with the water temperature maintained at 23°C ± 2°C. Additionally, 80 mL of black ink was introduced to create a contrast against the rats’ natural colouration. The Ethovision XT behavioural software subdivided the water maze into four quadrants, designating the northwest as the Ⅰ quadrant, the northeast as the Ⅱ quadrant, the southeast as the Ⅲ quadrant, and the southwest as the Ⅳ quadrant. Within the setup, a black circular platform (12 cm in diameter) was positioned in the second quadrant and submerged approximately 1 cm beneath the water surface.

During days 1–5, the concealed platform trial was executed, with the rats undergoing daily training sessions from each of the four quadrants, each session lasting for 60 s. The primary measure was the time taken by the rats to locate the hidden platform within the allocated 60-s time frame, referred to as escape latency. Timing commenced upon the rat’s introduction into the pool was automatically recorded by the system. If a rat successfully reached the platform within 60 s, the system ceased timing and allowed the rat a 10-s respite on the platform before concluding the session. In cases where the rat failed to locate the platform within the 60-s period, it was gently guided to the platform and permitted to remain there for 10 s, with the system registering an escape latency of 60 s for that particular attempt.

On the sixth day, probe trials were executed, with the platform removed. Rats were placed into the pool from quadrants Ⅳ and Ⅰ. The evaluation criteria included the number of times the rats crossed the previous platform location, defined as the “annulus visits” based on previous studies (Song et al., 2021), the time spent in the target quadrant, and the distance swam within this quadrant during the 60-s trial period. The average values derived from the two-quadrant assessments were employed to evaluate the rats’ spatial learning and memory capabilities.

### 2.6 Sample collection

Subsequent to the behavioural assessments, the rats underwent a 12-h fasting period, followed by anaesthetisation with Zoletil^®^ 50 (50 mg/kg). The rats’ heads were then severed utilising a decapitation device. Following removal of skin and skull, the PFC and striatum were swiftly stripped on the ice-cooled surface using a glass splitting needle. Any residual blood and hair were meticulously washed away with cold PBS, and the dissected PFC and striatum were freezed rapidly with liquid nitrogen and preserved at −80°C thereafter. For sample collection for immunohistochemistry, we randomly selected four rats from each group and stripped the whole brain, which was rapidly fixed in pre-cooled 4% paraformaldehyde overnight at 4°C before isolating the prefrontal cortex (PFC) and the striatum and sectioning them serially in the sagittal position.

### 2.7 High-performance liquid chromatography (HPLC)

HPLC was used for the determination of DA content in the PFC and striatum. The HPLC device was Rigol L3000 high-performance liquid chromatography analyser, and the column selected was the Sepax C18 reversed-phase column (250 mm × 4.6 mm, 5 μm). The chromatographic conditions included a mobile phase composed of a 20 mmol/L potassium dihydrogen phosphate aqueous solution, an injection volume of 10 μL, a flow rate of 0.8 mL/min, a column temperature set at 30°C, and a sample run time of 30 min. Additionally, the excitation wavelength was 285 nm, and the emission wavelength was 318 nm. The sample was prepared by taking an appropriate quantity and mixing it with methanol (100 mg: 1 mL). After grinding, the mixture was centrifuged at 4,000 rpm/min for 10 min. Subsequently, the supernatant was separated and divided. From the supernatant, 50 μL was extracted and then diluted fourfold with 150 μL of an extraction solution (comprising a 0.1 mol/L hydrochloric acid solution and methanol in a 9:1 ratio). The final step involved filtration, and the sample was then subjected to the machine for testing.

### 2.8 Ultra-high performance liquid chromatography-mass spectrometry (UPLC-MS)

For the determination of norepinephrine (NE) content in the PFC and striatum, UPLC-MS was utilised. The instrumentation included the Waters Acquity Ultra-High Performance Liquid Chromatograph (Waters Corporation, USA) and the API5500 Triple Quadrupole Mass Spectrometer (AB Sciex Corporation, USA). The liquid phase conditions encompassed a mobile phase comprising A (2 mM ammonium acetate containing 0.1% formic acid) and B (acetonitrile). A gradient elution was executed as follows: 0–1.5 min at 22% A, 1.5–2.5 min at 22%–50% A, 2.5–3.0 min at 50%–60% A, 3.0–4.0 min at 60% A, and 4.0–6.0 min at 22% A. The flow rate was 0.3 mL/min, and a volume of 2 μL was injected into the UPLC-MS/MS system. Chromatographic separations took place on a Waters Acquity UPLC BEH AMIDE column (2.1 mm × 100 mm, 1.7 μm) set at 40°C. The mass spectrometry conditions were as follows: ion spray voltage at 5500 V, curtain gas at 241.3 kPa, CAD at 48.3 kPa, source temperature at 550°C, nebulizer gas (Gas 1) at 413.7 kPa, and heater gas (Gas 2) at 413.7 kPa. All instruments were controlled and synchronised using Analyst software (version 1.6.3; Applied Biosystems/MDS Sciex).

### 2.9 Enzyme-linked immunosorbent assay (ELISA)

Rat AC and cAMP ELISA Kits (ER9268M, ER9351M) were procured from Biotech Well (Shanghai, China) for the detection of AC and cAMP concentration levels in the PFC and striatum. The assays were conducted in accordance with the manufacturer’s provided protocols. All reagents were returned to room temperature before the experiment. The PFC and striatum were fully homogenized at a ratio of tissue (g):1×PBS (mL) = 1:9, centrifuged at 3,000 rpm for 20 mintues, and the supernatant was collected for BCA protein quantification, and the washing solution and standard solutions were configured according to the instructions. The 50 μL standard dilution was added into one blank well of 96T ELISA plate, 50 μL standard solutions or samples were added into the rest of the wells accordingly, and three replicate wells were set up for each sample, which were mixed well and incubated at 37°C for 50 mintues. After the plate was washed with washing solution, 100 μL biotinylated antibody working solution was added and incubated at 37°C for 50 mintues. After washing the plate again, SABC complex working solution was added to each well and incubated for 30 min at 37°C. The plate was washed repeatedly, color development solution and termination solution were added, and the absorbance value (OD) was measured at 450 nm using the multifunctional enzyme-linked immunosorbent assay (ELISA) reader (Synergy H1MF, USA). The standard curve was fitted using a four-parameter logistic curve, and the AC and cAMP concentrations of the corresponding samples were calculated based on the OD values of the samples.

### 2.10 Immunohistochemistry

Four rats were randomly selected from each group, and the whole brains were rapidly peeled off on ice and fixed with 4% paraformaldehyde. After overnight at 4°C, the PFC and striatum samples of the whole brain were isolated, paraffin-embedded, and sliced into sections of 3–4 μm thickness. The sections were subjected to dewaxing, antigen retrieval, endogenous peroxidase blocking, and blocking with 3% BSA. Following this, they were incubated overnight at 4°C with primary antibodies, including PKA (1:200), p-CREB (1:200), and BDNF (1:100). Subsequently, the sections were exposed to an HRP-labelled goat anti-rabbit IgG (1:200) as the secondary antibody, and this incubation was carried out at room temperature for 50 min. The sections were further processed by staining the nuclei with DAB, hematoxylin restaining, dehydration, and sealing. High-resolution scans were performed on the sections at ×400 magnification, with positive expression exhibited as brownish-yellow. For each sample, five high-magnification fields of view were randomly selected, and the average optical density (AOD = SUM IOD/SUM Area) of the brownish-yellow positively stained cells was analyzed using Image-Pro Plus 6.0 software, and the mean value of AOD of the five high-magnification fields of view was considered as the final result expression for that sample.

### 2.11 Western blotting

Appropriate quantities of the PFC and striatum were extracted, homogenized, and subjected to lysis. The resulting supernatant was subsequently centrifuged, and its concentration was determined using a BCA protein quantification kit (P0012, Beyotime, China). The protein content of Western blot was set to 60ug, and based on the protein concentration, the protein volume was calculated and the protein electrophoresis system solution was configured with 5×Loading buffer (P0015, Beyotime, China) and PBS. After the protein electrophoresis system solution was denatured at 95°C for 10 min, it was electrophoresed using sulfate-polyacrylamide gel electrophoresis (SDS-PAGE) equipment (1703810, Bio-Rad, USA) and then transferred to PVDF membranes using a membrane transfer device (1658004, Bio-Rad, USA). After the completion of membrane transfer, the PVDF membranes were incubated with 5% BSA for 1 h at room temperature, after which they were hybridized with primary antibodies DRD1 (1:2000, 49KD), Gαs (1:2000, 46KD), Gαolf (1:700, 40KD), PKA (1:1000, 40KD), p-CREB (1:500, 43KD), and BDNF (1:2000, 15KD) and incubated overnight at 4°C. GAPDH (1:2000, 36KD) was selected as a standardized internal reference according to the molecular weight of the target protein (>40KD or <30KD). On the second day, the membrane was washed with TBST for 10 min, five times. Subsequently, the membrane was incubated with the secondary antibody (1:2000) at room temperature for 1 h. After another five washes with TBST, the ultra-sensitive chemiluminescent solution (BL520A, Biosharp, China) was added dropwise onto the PVDF membrane and perform development on the gel imaging analysis system (Chemiscope 6,300, Shanghai, China). After the ImageJ software converted the protein bands to 8 bit format and removed the background, the gray scale values of the bands were analyzed and the ratio of the target protein to GAPDH was used as the final expression of the results. Each index was run three times for each group.

### 2.12 Real-time fluorescence quantitative reverse transcription PCR (RT-qPCR)

Total RNA was extracted from the PFC and striatum using Trizol, and the RNA concentration was measured at 260 nm using an ultramicro spectrophotometer (Nanodrop 2000). When the OD260/280 of the detected RNA = 1.9-2.1, the RNA purity is proved to be good. According to the instructions, the total amount of RNA was set to 2ug, and the RNA was reverse transcribed into cDNA using the Invitrogen First Strand cDNA synthesis Kit with the following reverse transcription conditions: 25°C for 5 min, 42°C for 60 min, and 70°C for 5 min. In the PCR reaction system, primers were designed and synthesized by NCBI Primer-blast with reference to the sequences of the target genes in the Gene Bank database, and the primer sequences are shown in Table 2. The cDNA was subsequently amplified using a SYBR Green PCR Kit with the following thermal cycling conditions: 95°C for 2 min, followed by 45 cycles of 95°C for 5 s and 60°C for 10 s, and the specific reaction system was shown in Table 3. The fluorescence quantitative PCR instrument outputted the experimental results, and GAPDH was used as an standardized internal reference to calculate the △Ct value (△Ct = CT _target gene_ - CT _GAPDH_), and then the first sample of the control group was used as the control to calculate the △△Ct value (△△Ct = △CT - △CT _control_), and finally, the 2^−ΔΔCt^ algorithm (Livak and Schmittgen, 2001) was utilised to analyse the relative expression of DRD1, Gαs, Gαolf, PKA, CREB, and BDNF mRNAs. The biological sample size of each group of RT-qPCR is equal to 5.

**TABLE 2:** Primer sequences used for RT-qPCR.

Gene	Forward (5′- 3′)	Rewerse (5′- 3′)
DRD1 (103bp)	TCG​AAC​TGT​ATG​GTG​CCC​TT	AAGAATTCACCCAAAC
Gαs (181bp)	GAA​ACA​GCC​CTA​TCA​GCA​GC	CCG​ATC​TGA​AGA​ACT​GCG​TG
Gαolf (160bp)	TTT​CCC​GGA​GTA​TGC​CAA​CT	GGT​GAA​GTG​AGG​GTA​GCA​GT
PKA (88bp)	AAC​CTT​CTC​ATC​GAC​CAG​CA	CAC​ACA​AGG​TCC​AAG​TTC​GG
CREB (87bp)	ACC​AGC​AGA​GTG​GAG​ATG​CT	GGG​CTA​ATG​TGG​CAA​TCT​GT
BDNF (141bp)	GCG​GCA​GAT​AAA​AAG​ACT​GC	GCA​GCC​TTC​CTT​CGT​GTA​AC
GAPDH (128bp)	GGC​AAG​TTC​AAC​GGC​ACA​GT	ATG​ACA​TAC​TCA​GCA​CCG​GC

**TABLE 3:** Reaction system of qPCR.

Reagents	Volume (ul)
RNase-free water	1.5
Forward Primer (10uM)	0.5
Reverse Primer (10uM)	0.5
2×SYBR Green PCR Master Mix	5
cDNA	2.5

### 2.13 Statistical analysis

The data were subjected to analysis using SPSS 25.0 software, and GraphPad Prism 8.0 software was utilised for creating graphical representations. Measurements were presented as mean values ±standard deviation. Between-group comparisons were conducted using one-way analysis of variance (ANOVA), and *post hoc* multiple comparisons were performed using LSD. For the OFT, data encompassing pre- and post-treatment measurements, involving the variables of time and outcome, necessitated the use of two-way ANOVA. In the MWM test, data pertaining to escape latency over 5 days were analysed using a two-way repeated measures ANOVA. A significance level of *p* < 0.05 was considered indicative of statistical significance.

## 3 Results

### 3.1 Effect of LMQXM on body weight of rats

The body weights of the rats in each group are shown in Table 4, and LMQXM had no significant effect on the weight gain of the rats.

**TABLE 4:** Changes in body weight of rats in each group.

Groups	Before administration (g)	7 days of administration (g)	14 days of administration (g)	21 days of administration (g)	28 days of administration (g)
WKY	102.53 ± 6.15	134.03 ± 8.69	174.42 ± 12.67	201.33 ± 14.26	236.38 ± 15.9
SHR	100.32 ± 6.33	135.24 ± 9.56	178.42 ± 12.06	210.33 ± 14.2	240.05 ± 17.55
MPH	98.47 ± 5.46	131.23 ± 8.56	170.24 ± 10.71	204.18 ± 9.99	235.89 ± 12.39
LMQXM-LD	98.9 ± 8.86	130.96 ± 12.41	171.43 ± 17.9	206.43 ± 18.04	241.81 ± 16.07
LMQXM-MD	101.97 ± 9.49	132.63 ± 12.25	174.68 ± 14.82	189.77 ± 56.69	232.29 ± 16.4
LMQXM-HD	100.87 ± 7.3	132.19 ± 9.57	162.91 ± 10.62	203.8 ± 11.08	237.76 ± 12.86

### 3.2 Effect of LMQXM on the behavioral performance of SHR/NCrl rats

#### 3.2.1 Effect of LMQXM on the activity level of SHR/NCrl rats

**FIGURE 2:**
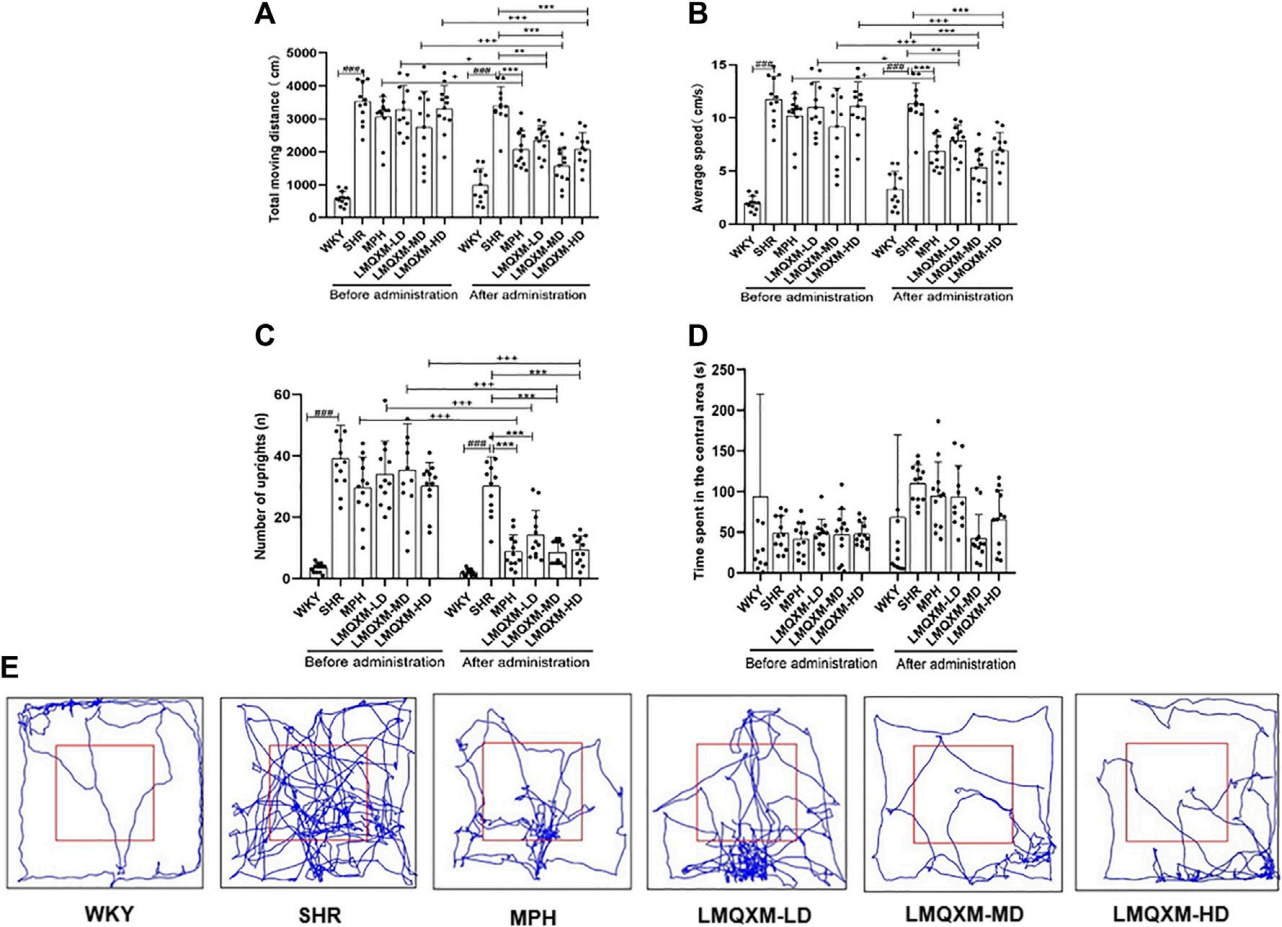
Effects of LMQXM on the activity level of rats. **(A)** Changes in the total moveing distance of rats in each group; **(B)** Changes in the average speed of rats in each group; **(C)** Changes in the number of uprights of rats in each group; **(D)** Changes in the time spent in the central area of rats in each group; **(E)** Representative movement trajectories of rats in each group. Data are expressed as mean ± standard deviation, *n* = 12 for each group. ^###^ *p* < 0.001 compared with WKY group. ^*^ *p* < 0.05, ^**^ *p* < 0.01, and ^***^ *p* < 0.001 compared with SHR group. ^+^ *p* < 0.05, ^++^ *p* < 0.01, and ^+++^ *p* < 0.001 compared with the same group before treatment.

To evaluate the impact of LMQXM on the activity level of the rats, we conducted assessments of total moving distance, average speed, the number of uprights, and time spent in the central area using the OFT. We tracked the movement patterns of the rats before and after treatment. In comparison to WKY/NCrl rats, SHR/NCrl rats displayed significantly greater total moving distance, average speed, and the number of uprights (Figures 2A–C), which indicated significantly excessive activity levels in the SHR/NCrl rats, supporting the surface validity of these behaviours in this model (Yuan et al., 2019). Following treatment, the MPH, LMQXM-LD, LMQXM-MD, and LMQXM-HD groups exhibited substantial reductions in total moving distance, average speed, and the number of uprights in comparison to their pre-treatment levels (Figures 2A–C). However, after treatment, SHR/NCrl rats still displayed higher total moving distance, average speed, and the number of uprights compared to WKY/NCrl rats (Figures 2A–C). Nevertheless, these parameters were significantly reduced in the MPH, LMQXM-LD, LMQXM-MD, and LMQXM-HD groups in comparison to the SHR group (Figures 2A–C). These findings demonstrate that LMQXM effectively ameliorated hyperactive behaviours in SHR/NCrl rats. Notably, the time spent in the central area, often indicative of anxiety in rats, did not exhibit any statistically significant differences between SHR/NCrl rats and WKY/NCrl rats, neither before nor after treatment (Figure 2D). Typical trajectory diagrams are presented in Figure 2E.

Specific statistical descriptions and statistical reports on the effects of LMQXM on rat activity levels can be found in Supplementary Tables S2-S11.

#### 3.2.2 Effects of LMQXM on spatial learning and memory of SHR/NCrl rats

**FIGURE 3:**
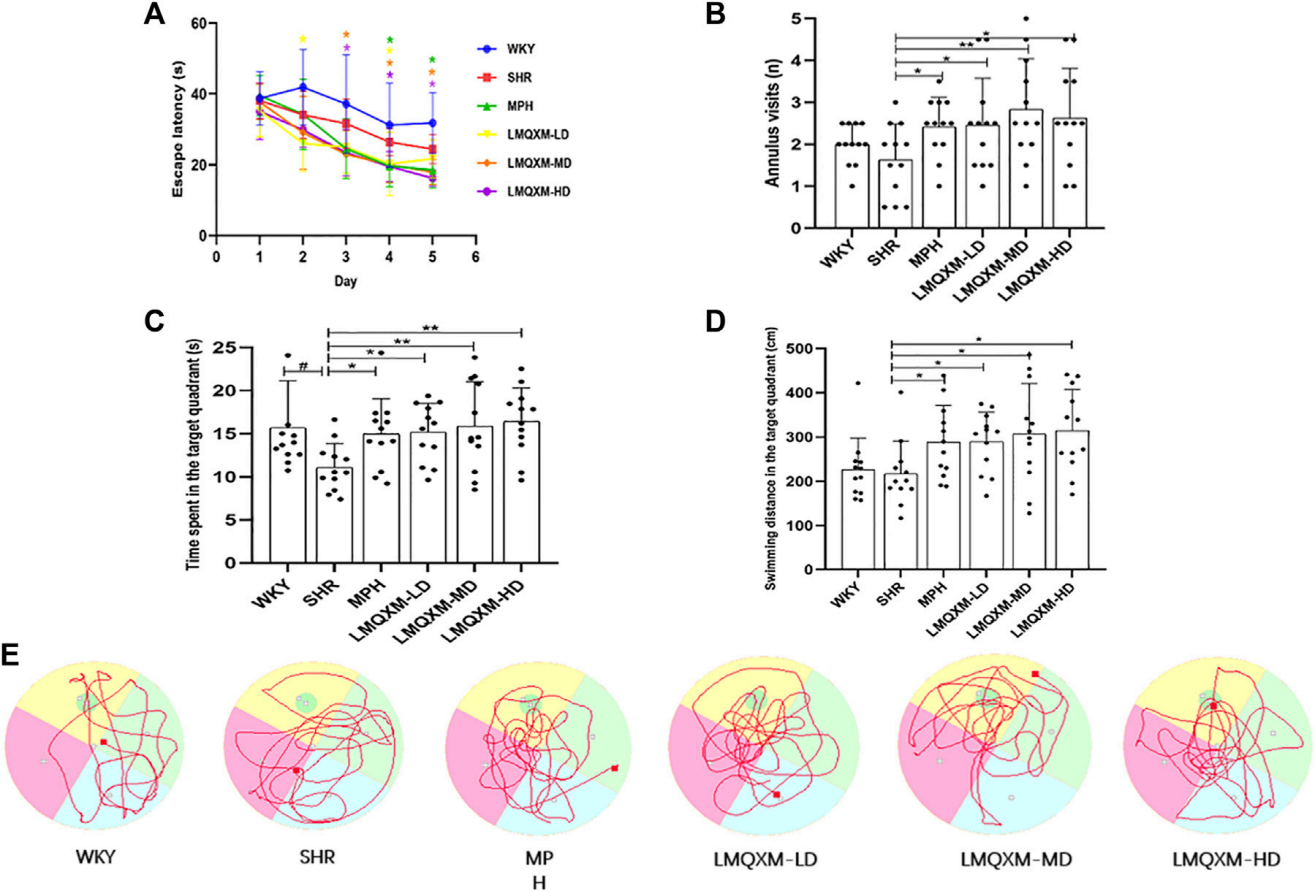
Effects of LMQXM on spatial learning and memory in rats. **(A)** Changes in escape latency of rats in each group; **(B)** Annulus visits of rats in each group; **(C)** Time spent in the target quadrant of rats in each group; **(D)** Swimming distance in the target quadrant in each group; **(E)** Locomotor trajectories of rats in each group in the WMW test. Data are expressed as mean ± standard deviation, *n* = 12 for each group. Compared with the WKY group, ^#^ *p* < 0.05, and compared with the SHR group, ^*^ *p* < 0.05, ^**^ *p* < 0.01.

To evaluate the impact of LMQXM on learning and memory in the rats, the MWM tests were conducted on the 22nd day of drug administration and continued for 6 days. Key measurements included escape latency, annulus visits (number of times crossing platforms), swimming time and distance in the target quadrant. Throughout the concealed platform test, escape latency for all groups displayed a declining trend as time progressed. There were no statistically significant differences in escape latency between the WKY group and the SHR group (Figure 3A). In comparison to the SHR group, the MPH group exhibited significant reductions in escape latency on days 4 and 5 (Figure 3A), the LMQXM-LD group displayed decreased escape latency on days 2 and 4 (Figure 3A), and the LMQXM-MD and LMQXM-HD groups showed reduced escape latency on days 3, 4, and 5 (Figure 3A). In the probe trials, SHR/NCrl rats showed a decrease in time spent in the target quadrant in contrast to WKY/NCrl rats (Figure 3C), indicating impaired spatial learning and memory in SHR/NCrl rats (Yuan et al., 2019). The MPH group, the LMQXM-LD group, the LMQXM-MD group, and the LMQXM-HD group exhibited significantly more annulus visits, longer time spent in the target quadrant, and increased swimming distance in the target quadrant compared to the SHR group (Figures 3B–D). Representative trajectories of rats from each group in the MWM test are depicted in Figure 3E.

Specific statistical descriptions and statistical reports of the effects of LMQXM on learning and memory on rats can be found in Supplementary Table S12-15.

### 3.3 Effects of LMQXM on brain tissue DA and NE levels in SHR/NCrl rats

**FIGURE 4:**
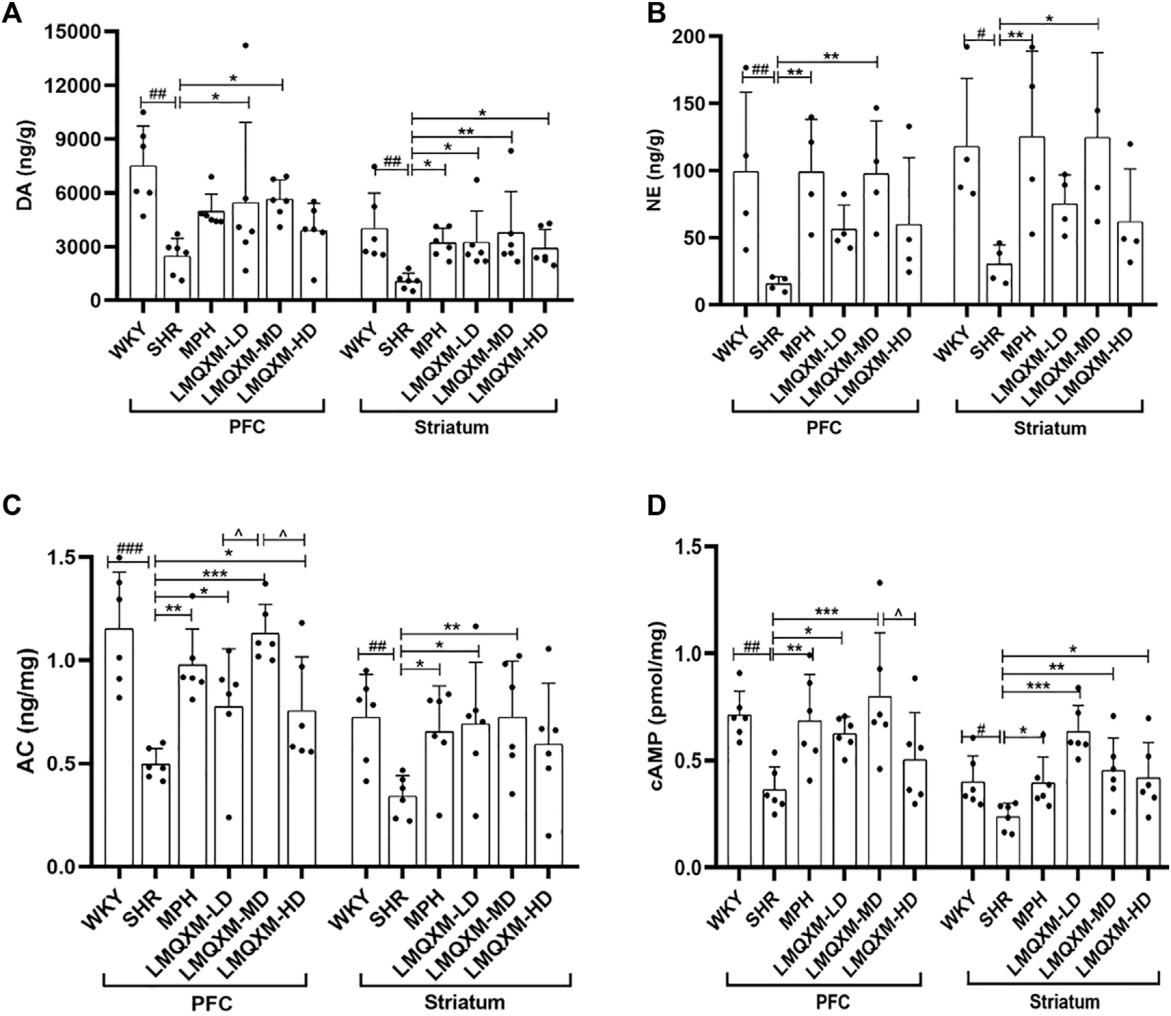
Effects of LMQXM on DA, NE, AC, and cAMP levels in rat PFC and striatum. **(A)** DA level; **(B)** NE level; **(C)** AC level; **(D)** cAMP level. Data are expressed as mean ± standard deviation, *n* = 6 for each group of DA, AC, and cAMP, and *n* = 4 for each group of NE. Compared with the WKY group, ^#^ *p* < 0.05, ^##^ *p* < 0.01, and ^###^ *p* < 0.001. Compared with the SHR group, ^*^ *p* < 0.05, ^**^ *p* < 0.01, ^***^ *p* < 0.001 and compared with the LMQXM-MD group, ^^^ *p* < 0.05.

DA and NE are pivotal catecholamine neurotransmitters that underpin various executive functions of the brain (Del Campo et al., 2011). We employed HPLC and LC-MS to assess the levels of DA and NE in the PFC and striatum, respectively. In comparison to WKY/NCrl rats, the PFC and striatum of SHR/NCrl rats exhibited a significant reduction in DA and NE levels (Figures 4A,B). Following treatment, the LMQXM-MD group displayed a noteworthy increase in DA and NE levels in the PFC and striatum when compared to the SHR group (Figures 4A,B). Additionally, the MPH group exhibited elevated DA levels in the striatum, along with higher NE levels in the PFC and striatum in contrast to the SHR group (Figures 4A,B). Notably, the LMQXM-LD group demonstrated an upregulation of DA levels in both the PFC and striatum, while the LMQXM-HD group specifically enhanced DA levels in the striatum (Figure 4A). These findings substantiate the capacity of LMQXM to augment catecholamine neurotransmitter content within brain tissue.

Statistical descriptions and statistical reports of rat catecholamine neurotransmitter content in the PFC and striatum are in Supplementary Table S16, 17.

### 3.4 Effects of LMQXM on AC and cAMP levels in brain tissue of SHR/NCrl rats

cAMP serves as a downstream effector of DA, produced through the activation of AC and the hydrolysis of ATP. Our results indicated a downregulation of AC and cAMP levels in the PFC and striatum of SHR/NCrl rats compared to WKY/NCrl rats (Figures 4C,D). Post-treatment, the MPH, LMQXM-LD, and LMQXM-MD groups exhibited a substantial increase in AC and cAMP levels in the PFC and striatum relative to the SHR group (Figures 4C,D). In addition, the LMQXM-HD group upregulated AC levels in the PFC and cAMP levels in the striatum (Figures 4C, D). Furthermore, it is noteworthy that the LMQXM-MD group was more effective in up-regulating the AC level in the PFC compared to the LMQXM-LD and LMQXM-HD groups (Figure 4C). These results suggest that LMQXM effectively activates the cAMP signaling pathway with AC as the principal effector enzyme.

Statistical descriptions and statistical reports on AC, cAMP levels in rat PFC and striatum are in Supplementary Table S18, 19.

### 3.5 Effect of LMQXM on the expression of PKA, p-CREB, and BDNF-positive cells in brain tissues of SHR/NCrl rats

**FIGURE 5:**
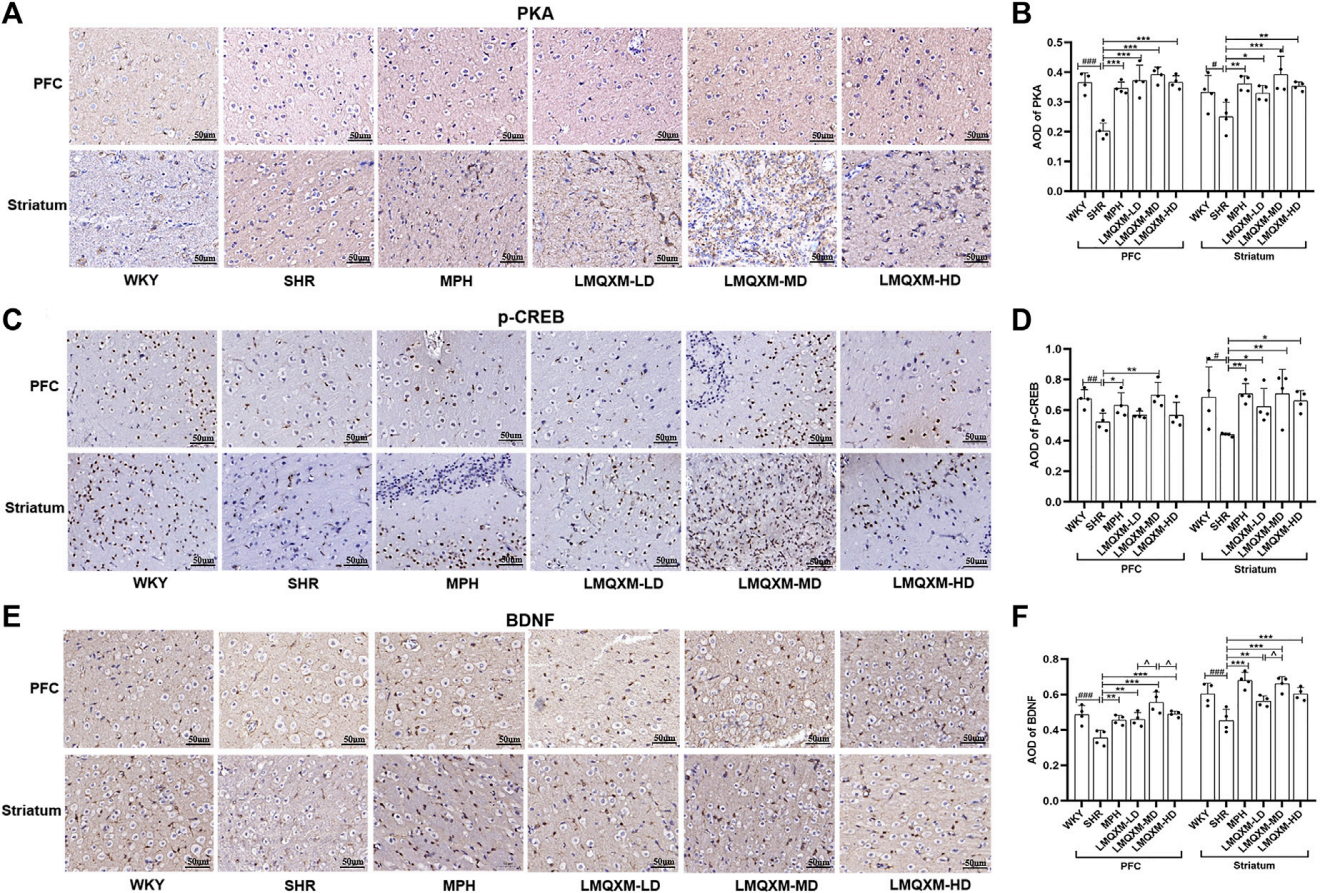
Effect of LMQXM on AOD of PKA, p-CREB, and BDNF in rat PFC and striatum. **(A)** Immunohistochemical staining of PKA (Scale: 50um); **(B)** AOD of PKA; **(C)** Immunohistochemical staining of p-CREB (Scale: 50um); **(D)** AOD of p-CREB; **(E)** Immunohistochemical staining of BDNF (Scale: 50um). **(F)** AOD of BDNF. The data are expressed as the mean ± the standard deviation, *n* = 4 for each group. Compared with the WKY group, ^#^ *p* < 0.05, ^##^ *p* < 0.01, and ^###^ *p* < 0.001. Compared with the SHR group, ^*^ *p* < 0.05, ^**^ *p* < 0.01, ^***^ *p* < 0.001, and compared with the LMQXM-MD group, ^^^ *p* < 0.05, ^^^^ *p* < 0.01.

PKA, the first identified cAMP effector, is catalytically activated by cAMP to phosphorylate a range of substrates, including CREB and BDNF, which play a role in the regulation of learning and memory through neuroprotective mechanisms (Guo et al., 2017). Immunohistochemistry was employed to assess the influence of LMQXM on the AOD of PKA, p-CREB, and BDNF in the PFC and striatum of the rats. The AOD of PKA, p-CREB, and BDNF was significantly reduced in the PFC and striatum of SHR/NCrl rats in comparison to WKY/NCrl rats (Figures 5A–F). However, following treatment, the MPH and LMQXM-MD groups exhibited a considerable increase in the AOD of PKA, p-CREB, and BDNF in the PFC and striatum compared to the SHR group (Figures 5A–F). In the LMQXM-LD and LMQXM-HD groups, the AOD of PKA and BDNF in the PFC and striatum, along with the AOD of p-CREB in the striatum, demonstrated an increase when compared to the SHR group (Figures 5A–F). Intriguingly, the AOD of BDNF in the LMQXM-MD group was notably superior to that in the LMQXM-LD and LMQXM-HD groups within the PFC (Figures 5C,D). In the striatum, there was a significant difference in the AOD expression of BDNF between the LMQXM-MD group and the LMQXM-LD group (Figures 5C,D). These findings suggest that the medium dose may be the optimal dosage for the activation of PKA and its downstream substrates.

Average optical density values and detailed statistical reports of PKA, p-CREB, and BDNF in the rat prefrontal and striatum can be read in Supplementary Table S20.

### 3.6 Effects of LMQXM on DRD1, Gαs, Gαolf, PKA, p-CREB, and BDNF protein expression in brain tissues of SHR/NCrl rats

**FIGURE 6:**
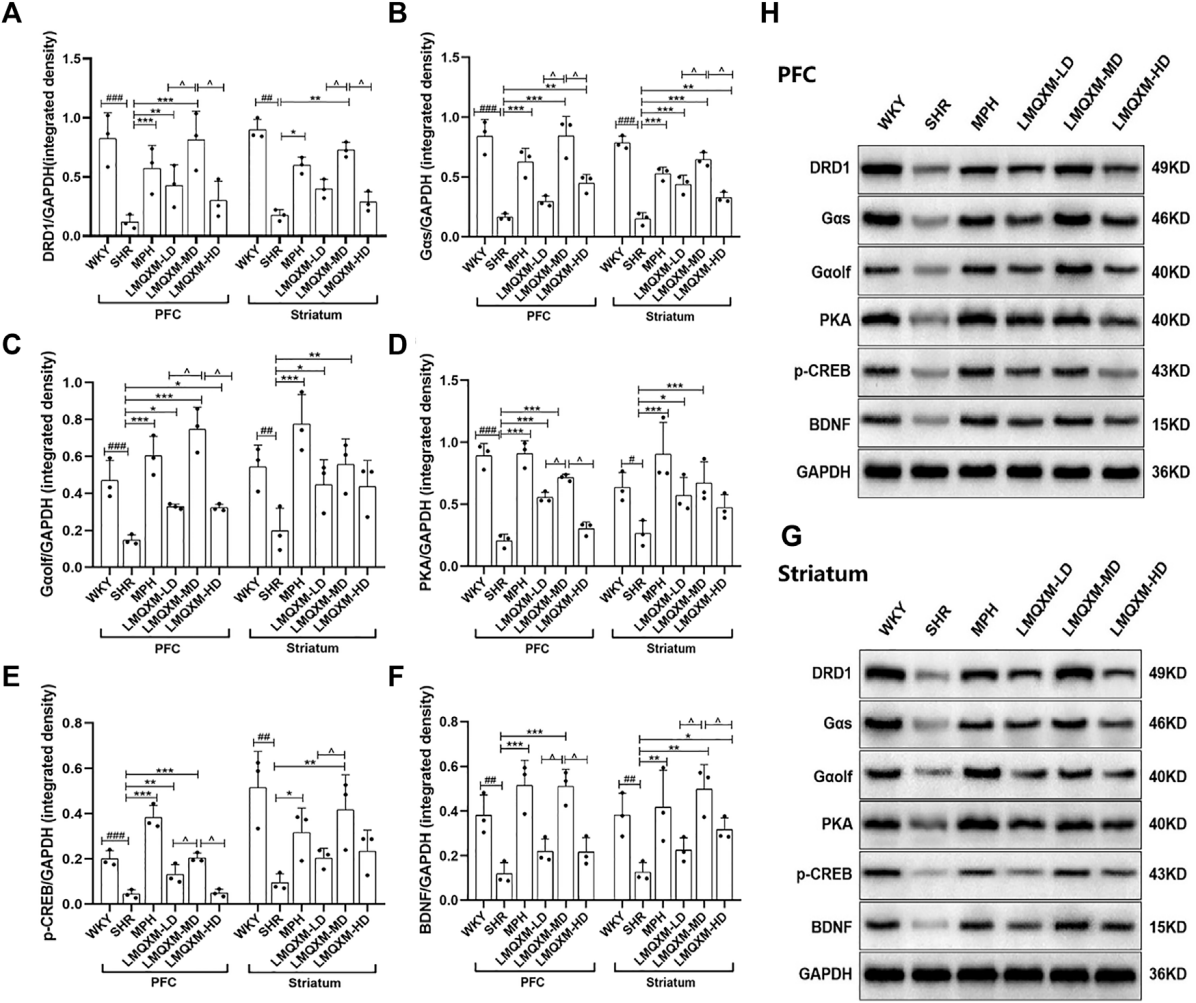
Effects of LMQXM on the relative expression of DRD1, Gαs, Gαolf, PKA, p-CREB, and BDNF proteins in rat PFC and striatum. **(A)** Relative expression of DRD1 proteins; **(B)** Relative expression of Gαs proteins; **(C)** Relative expression of Gαolf proteins; **(D)** Relative expression of PKA proteins; **(E)** Relative expression of p-CREB proteins; **(F)** Relative expression of BDNF proteins. **(H)** Western blot analysis images of each group of proteins in PFC; **(G)** Western blot analysis images of each group of proteins in striatum. Data are expressed as mean ± standard deviation, *n* = 3 for each group. Compared with the WKY group, ^#^ *p* < 0.05, ^##^ *p* < 0.01, ^###^ *p* < 0.001, compared with the SHR group, ^*^ *p* < 0.05, ^**^ *p* < 0.01, ^***^ *p* < 0.001 and compared with the LMQXM-MD group, ^^^ *p* < 0.05.

To elucidate the impact of LMQXM on the DRD1/cAMP/PKA-CREB signaling pathway in the brain tissue of SHR/NCrl rats, we conducted a comprehensive analysis of the protein expression of relevant pathway in the PFC and striatum using Western blotting. Our findings revealed a notable reduction in DRD1, Gαs, Gαolf, PKA, p-CREB, and BDNF protein expression in both the PFC and striatum of SHR/NCrl rats when compared to WKY/NCrl rats (Figures 6A–G). Following the administration, the MPH and LMQXM-MD groups exhibited a marked increase in the protein expression of DRD1, Gαs, Gαolf, PKA, p-CREB, and BDNF in the PFC and striatum, relative to the SHR group (Figures 6A–G). In the LMQXM-LD groups, we observed elevated protein expression of Gαolf and PKA in both the PFC and striatum, along with increased DRD1 and p-CREB in the PFC and Gαs in the striatum, when compared to the SHR group (Figures 6A–E). The LMQXM-HD group demonstrated a significant upregulation of Gαs in both the PFC and striatum, Gαolf in the PFC, and BDNF in the striatum when compared to the SHR group (Figures 6B, C, F). Further investigation into the differences in protein expression among the three LMQXM dose groups indicated that the medium dose was the most effective at elevating DRD1, Gαs, and BDNF proteins in the PFC and striatum, as well as enhancing Gαolf, PKA, and p-CREB proteins in the PFC, outperforming the low and high doses (Figures 6A–G).

Statistical descriptions and statistical reports on the expression of DRD1, Gαs, Gαolf, PKA, p-CREB, and BDNF proteins in rat and striatum can be found in Supplementary Tables S21-S24.

### 3.7 Effects of LMQXM on DRD1, Gαs, Gαolf, PKA, CREB, and BDNF mRNA expression in brain tissue of SHR/NCrl rats

**FIGURE 7:**
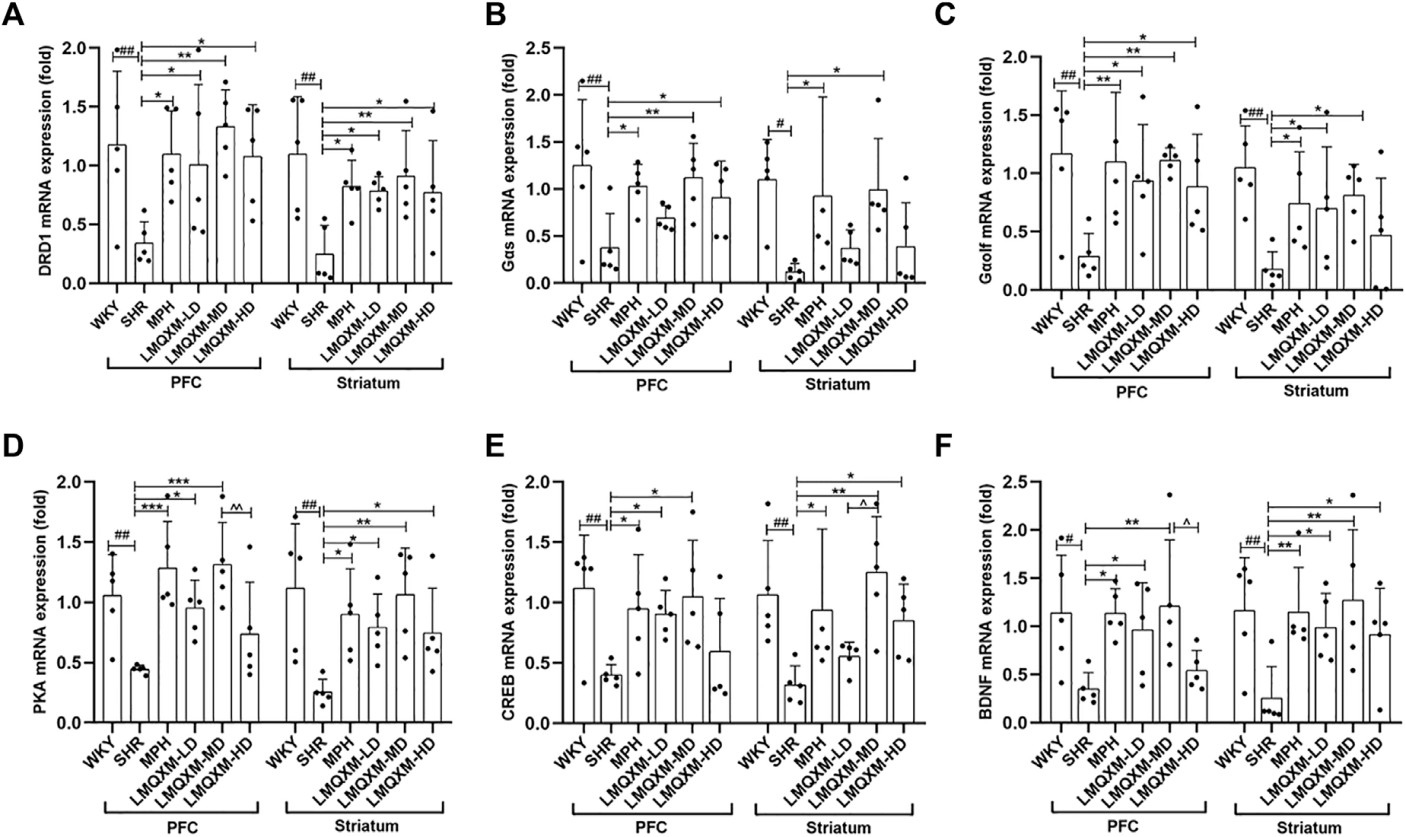
Effect of LMQXM on the relative expression of DRD1, Gαs, Gαolf, PKA, p-CREB, and BDNF mRNA in rat PFC and striatum. **(A)** DRD1 mRNA relative expression; **(B)** Gαs mRNA relative expression; **(C)** Gαolf mRNA relative expression; **(D)** PKA mRNA relative expression; **(E)** CREB mRNA relative expression; **(F)** BDNF mRNA relative expression. Data are expressed as mean ± standard deviation, *n* = 5 for each group. Compared with the WKY group, ^#^ *p* < 0.05, ^##^ *p* < 0.01, and ^###^ *p* < 0.001, compared with the SHR group, ^*^ *p* < 0.05, ^**^ *p* < 0.01, ^***^ *p* < 0.001and compared with the LMQXM-MD group, ^^^ *p* < 0.05.

We employed RT-qPCR to evaluate the impact of LMQXM on mRNA expression of factors associated with the DRD1/cAMP/PKA-CREB signaling pathway in the PFC and striatum. The relative expression of DRD1, Gαs, Gαolf, PKA, CREB, and BDNF mRNA was significantly reduced in the PFC and striatum of SHR/NCrl rats when compared to WKY/NCrl rats (Figures 7A–F). Following treatment, the MPH and LMQXM-MD groups demonstrated a substantial increase in the relative expression of DRD1, Gαs, Gαolf, PKA, CREB, and BDNF mRNA in both the PFC and striatum relative to the SHR group (Figures 7A–F). The LMQXM-LD group exhibited a notable upregulation in the relative expression of DRD1, Gαolf, PKA, and BDNF mRNA in the PFC and striatum, and CREB mRNA in the PFC, when compared to the SHR group (Figure 7AC-F). Slightly different effects from LMQXM-LD, the LMQXM-HD group displayed increased relative expression of DRD1 mRNA in both the PFC and striatum, Gαs and Gαolf mRNA in the PFC, and PKA, CREB, and BDNF mRNA in the striatum compared to the SHR group (Figures 7A–F). Notably, the relative expression of PKA and BDNF mRNA in the PFC was significantly higher in the LMQXM-MD group than in the LMQXM-HD group (Figures 7D,F), and furthermore, we observed a significant advantage in the upregulation of the relative expression of CREB mRNA in the striatum in the LMQXM-MD group as compared to the LMQXM-LD group (Figure 7E).

Specific statistical descriptions and statistical reports of DRD1, Gαs, Gαolf, PKA, p-CREB, and BDNF mRNA expression in rat and striatum can be found in Supplementary Tables S25-S28.

## 4 Discussion

Previously, we preliminarily observed the efficacy of LMQXM in treating ADHD and its ability to promote DA synthesis through clinical small-sample studies and animal experiments, and in the present study, we delved deeper into the potential mechanisms by which LMQXM modulates the DRD1/cAMP/PKA-CREB signaling pathway to bolster the synthesis of catecholamine neurotransmitters.

Consistent with previous studies, in the present study, we found that compared to WKY/NCrl rats, SHR/NCrl rats still showed more higher activity levels and deficits in spatial learning and memory, findings that further strengthen the rationale for SHR/NCrl rats as an animal model of ADHD. Corresponding to the effects of MPH, the administration of LMQXM significantly reduced the total moving distance, average speed, and number of uprights in SHR/NCrl rats. Simultaneously, it markedly increased annulus visits, swim times and distance in the target quadrant. In terms of behavioral indicators, the total moving distance is usually used as an objective basis for assessing the level of hyperactivity and the amount of activity of animals, and the number of uprights reflects the exploratory behavior and the curiosity level of animals towards new environments (Song et al., 2021). Some scholars believe that the average speed can be regarded as an assessment standard for the degree of impulsivity in rats (Zhou et al., 2017), but this statement is still somewhat controversial at present. The results of our animal behavioral tests showed that LMQXM improved the hyperactive behaviors of SHR/NCrl rats and enhanced their spatial learning and memory abilities, importantly, all three dosages of LMQXM proved effective. It should be specified that we should also map out more objective measures, such as the 5-choice serial reaction time task (Bruinsma et al., 2019), to reflect the impulsive behavior of animals in future studies. Escape latency, as an indicator of long-term spatial memory in rats, was notably shortened by LMQXM-MD and LMQXM-HD on days 3–5, while LMQXM-LD significantly reduced escape latency on days 2 and 4. These findings imply that distinct doses of LMQXM yield differential effects on long-term memory in SHR/NCrl rats.

Abnormal behavior in ADHD is associated with downregulation of catecholamine neurotransmitters (Hayashi and Iwanami, 2018; Viggiano et al., 2004). Our study aligns with previous research by demonstrating decreased levels of DA and NE in the PFC and striatum of SHR/NCrl rats compared to WKY/NCrl rats. This deficiency in DA and NE expression in the PFC and striatum is a principal factor contributing to hyperactivity, impulsivity, and learning and memory deficits in SHRs (Russell, 2002). In the present study, we found that LMXQM-MD was able to increase DA and NE levels in the PFC and striatum of SHR/NCrl rats, while MPH was able to increase DA levels in the striatum, NE levels in the PFC and striatum of SHR/NCrl rats. This suggests that LMQXM and MPH improved the hyperactive-like behavior of SHR/NCrl rats by reversing the defects DA and NE expression in PFC and striatum and by promoting the normal transmission of DAergic and NEergic neurotransmitters, which is consistent with previous reports that MPH treats ADHD by increasing central DA and NE activity (Mechler et al., 2022). The LMQXM-LD group upregulated the DA levels of SHR/NCrl rats in the PFC and striatum, and the LMQXM-HD group only upregulated DA levels in the striatum, but they did not have a significant effect on NE levels, suggesting that both were less effective than LMQXM-MD in elevating catecholamine neurotransmitters. In addition, we believe that the mechanism by which LMQXM improves behavioral performance in ADHD is mainly about modulating DA levels in the PFC and striatum. A right dose is required to trigger NE, too high or too low of the dose may not have a significant effect on NE, and that NE is more closely related to higher impulsive behavior (Paterson et al., 2011). In contrast to previous studies, we found that LMQXM-HD did not produce significant changes in DA levels in PFC in either study, which we believe may be due to the fact that striatum DA is more sensitive to high doses of drugs, whereas the relatively sparse distribution of DA neurons in the PFC and the high turnover rate of DA resulted in a non-significant elevation of DA levels in the PFC. This also suggests that further studies on the kinetic mechanism of DA metabolism in the PFC are needed in the future.

The cAMP/PKA-CREB signaling pathway, which regulates the synthesis and release of DA and NE, has been shown to be a key target for the treatment of ADHD (Nishi et al., 2008; Wang et al., 2021) and is downregulated in SHR brain tissue (Zhou et al., 2017). Consistent with previous reports, we found that AC and cAMP levels in the PFC and striatum of SHR/NCrl rats were significantly lower than those of WKY/NCrl rats, and their expression of DRD1, Gαs, Gαolf, PKA, and downstream CREB and BDNF proteins and mRNAs were similarly defective. Downregulation of the cAMP/PKA-CREB signaling cascade in the brain of SHRs resulted in DA, NE production defects, which in turn led to behavioral disorders such as hyperactivity and impulsivity. MPH and LMQXM-MD administration were all able to promote an increase AC and cAMP content in PFC and striatum of SHRs, and upregulate the relative expression of DRD1, Gαs, Gαolf, PKA, p-CREB, and BDNF proteins and mRNA. It is reasonable to assume that the mechanism by which LMQXM elevates catecholamine neurotransmitters is through activation of the DRD1/cAMP/PKA-CREB signaling cascade. Previously, we found that DRD1 may play a more important role in neurotransmitter synthesis by observing DRD1 and DRD2, and this study of ours further confirms the previous conclusion that DRD1 is the main receptor subtype that activates the cAMP/PKA signaling pathway, and that this receptor, with its Gαs and Gαolf subunits, is involved in the synthesis of cAMP and catecholamine neurotransmitters.

Upon further investigation into the effects of LMQXM at low and high doses, we observed that both the LMQXM-LD and LMQXM-HD groups were capable of up-regulating AC and cAMP levels. However, they only exhibited partial upregulation of the proteins and genes within the DRD1/cAMP/PKA-CREB signaling pathway, and the extent of expression in the PFC and the striatum varied between these two groups. It is our contention that LMQXM at both low and high doses activates the cAMP/PKA-CREB signaling cascade to some extent, although this effect is not as pronounced as that of the medium dose. In the PFC, the LMQXM-MD group displayed superior effectiveness in elevating AC levels, BDNF positive cell expression, DRD1, Gαs, Gαolf, PKA, p-CREB, and BDNF protein expression compared to the LMQXM-LD and LMQXM-HD groups, and furthermore, its capacity to upregulate cAMP levels, PKA, and BDNF mRNA expression surpassed that of the LMQXM-HD group. In the striatum, the LMQXM-MD group exhibited superior effects in elevating DRD1, Gαs, BDNF protein expression when compared to the LMQXM-LD and LMQXM-HD groups, and in addition, its potential to enhance BDNF positive expression, p-CREB protein expression, and CREB mRNA expression exceeded that of the LMQXM-LD group.

Our findings highlight that the medium dose of LMQXM appear to optimally activates the cAMP/PKA-CREB signaling cascade. This is likely because the concentration of the drug components in the medium LMQXM dose closely approaches saturation in the bloodstream and brain tissues. Conversely, the low and high doses are influenced by the absorption rate of the intestinal epithelium and the passage rate through the blood-brain barrier, so that the concentration of the drug and its resultant effects are not as pronounced as those observed with the medium dose. Additionally, high-dose gavage may have a stimulating impact on the intestinal mucosa and the mental state of the rats. The relationship between LMQXM’s efficacy and the drug dosage, as well as the pharmacokinetics of LMQXM, warrants further investigation.

We potentially revealed an intrinsic relationship between defects in catecholamine neurotransmitters and DRD1/cAMP/PKA-CREB, which may be one of the mechanisms of action of LMQXM in alleviating ADHD. Nevertheless, factors contributing to catecholaminergic neurotransmitter deficits in ADHD are multifaceted. These factors include abnormalities in calcium signaling pathways, neuroinflammation, oxidative stress, environmental and genetic interactions, astrocyte-neuronal signalling abnormalities, and more (Nagai et al., 2019; Abu-Elfotuh et al., 2023). In future studies, we will delve deeper into the roles these factors play in the pathogenesis and treatment of ADHD.

Our study also has some limitations: for the Western blot analysis of each index, we used only three biological samples. The results obtained with this sample size limitation can only tentatively suggest that LMQXM has the potential to increase the levels of proteins associated with the cAMP/PKA-CREB signaling pathway, and therefore future studies should increase the number of biological samples studied to further enhance the statistical confidence. Another limitation involves the setup of control groups of SHR/NCrl rats. Although behavioral studies using WKY/NCrl rats as a control for SHR/NCrl rats can be very valuable as they show behavioral and neurobiological differences between the combined subtype (ADHD-C) and the predominantly inattentive subtype of ADHD (ADHD-PI) models (Sagvolden et al., 2009). It has been suggested that WKY/NCrl rats may not be suitable controls for SHR/NCrl rats due to the fact that WKY/NCrl rats have a similar genetic background (76.6% concordance) as SHR/NCrl rats, and behavioral changes can be manifested as inattentiveness as well as misleading differences in neurobiology (Sagvolden et al., 2009); instead, this genetic and behavioral characterization supports its use as a promising model for the ADHD-PI (Sagvolden et al., 2008; Dela Peña et al., 2015). The WKY/NHsd substrain exhibits lower genetic differences similar to SHR/NCrl rats (66.5% concordance) and both WH/HanTac and WKY/NCrl rats are genetically and behaviorally distinct from WKY/NHsd, so the available data support the use of WKY/NHsd rats as the most appropriate control for SHR/NCrl rats (Sagvolden et al., 2009). Therefore future studies should use the WKY/NHsd substrain as a control for the SHR/NCrl to further evaluate behavioral and molecular biological differences. In addition, our study was conducted with MPH, a first-line drug that can increase synaptic interstitial DA and NE activities for the treatment of ADHD (Reyes-Vasquez et al., 2023), as a positive control group. If the DA and NE monomer components can be added as positive controls, and the changes in the dopamine transporter (DAT), NE transporter (NET) and other targets related to the dynamics of DA and NE can be investigated, it will help to further unravel the potential pharmacological mechanism of LMQXM in alleviating ADHD through upregulating DA and NE.

## 5 Conclusion

In conclusion, our study preliminarily indicates that LMQXM may be improve hyperactivity and learning memory deficits in SHR/NCrl rats by enhancing catecholamine neurotransmitter levels in the prefrontal cortex and striatum. The mechanism behind this elevation of catecholamine neurotransmitters appears to be linked to the activation of the DRD1/cAMP/PKA-CREB signalling pathway, which is expected to yield potentially optimal results at moderate doses.

## Data Availability

The original contributions presented in the study are included in the article/Supplementary Material, further inquiries can be directed to the corresponding authors.
