# Supplementary figures and images for: Long Mu Qing Xin mixture improves behavioral performance in spontaneously hypertensive rats (SHR/NCrl) by upregulating catecholamine neurotransmitters in prefrontal cortex and striatum via DRD1/cAMP/PKA-CREB signaling pathway (part 1 of 4)
Source: Front Pharmacol. 2024 Jul 4;15:1387359. doi: 10.3389/fphar.2024.1387359 (PMC11254830; doi:10.3389/fphar.2024.1387359)

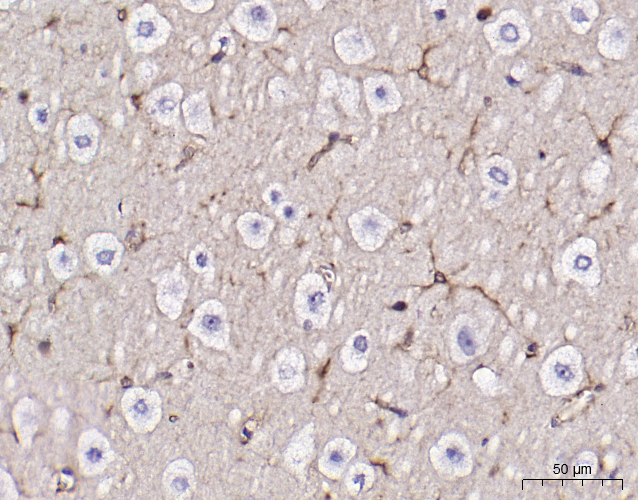

Supplement: Supplementary file 2 [file DataSheet3.ZIP › IHC Raw image of BDNF in PFC(3)/H34 1-100 BDNF_20.0x.tif-Q1.tif]

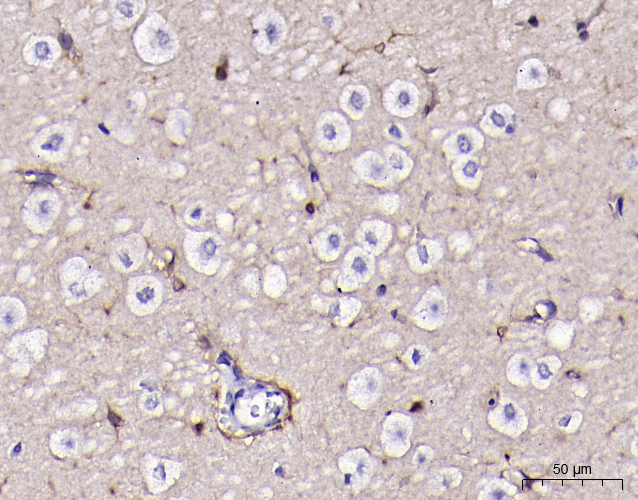

Supplement: Supplementary file 2 [file DataSheet3.ZIP › IHC Raw image of BDNF in PFC(3)/H34 1-100 BDNF_20.0x.tif-Q2.tif]

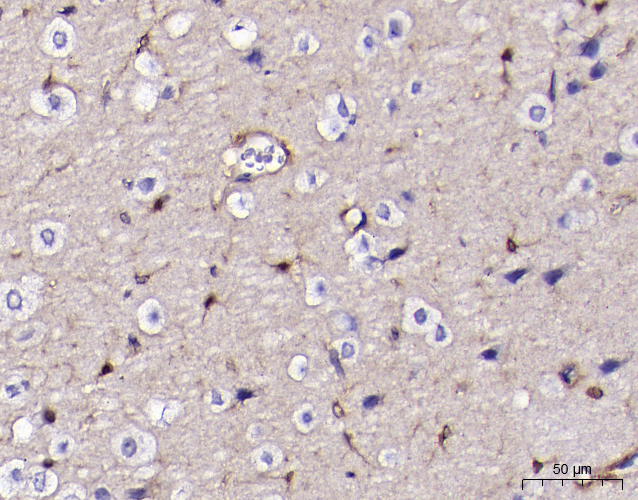

Supplement: Supplementary file 2 [file DataSheet3.ZIP › IHC Raw image of BDNF in PFC(3)/H34 1-100 BDNF_20.0x.tif-Q3.tif]

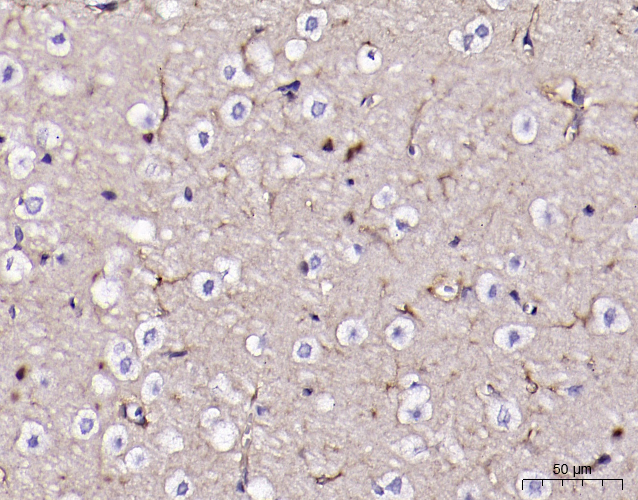

Supplement: Supplementary file 2 [file DataSheet3.ZIP › IHC Raw image of BDNF in PFC(3)/H34 1-100 BDNF_20.0x.tif-Q4.tif]

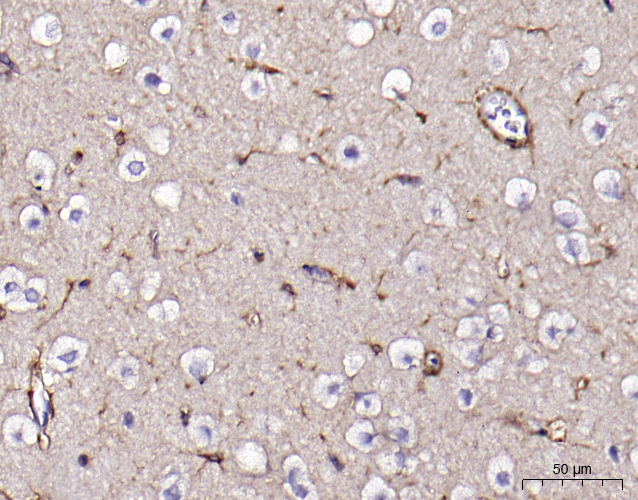

Supplement: Supplementary file 2 [file DataSheet3.ZIP › IHC Raw image of BDNF in PFC(3)/H34 1-100 BDNF_20.0x.tif-Q5.tif]

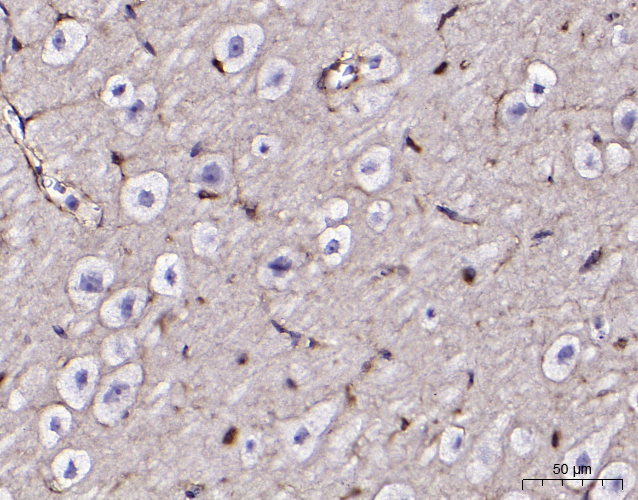

Supplement: Supplementary file 2 [file DataSheet3.ZIP › IHC Raw image of BDNF in PFC(3)/H41 1-100 BDNF_20.0x.tif-Q1.tif]

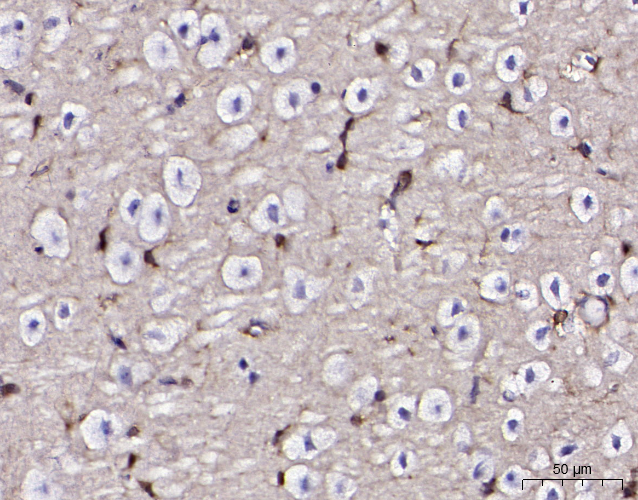

Supplement: Supplementary file 2 [file DataSheet3.ZIP › IHC Raw image of BDNF in PFC(3)/H41 1-100 BDNF_20.0x.tif-Q2.tif]

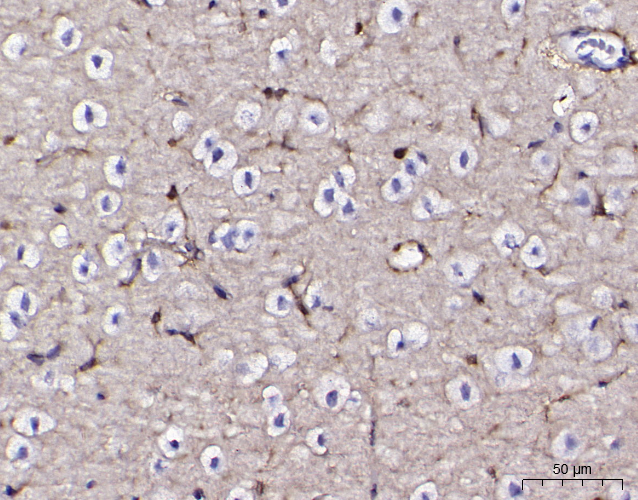

Supplement: Supplementary file 2 [file DataSheet3.ZIP › IHC Raw image of BDNF in PFC(3)/H41 1-100 BDNF_20.0x.tif-Q3.tif]

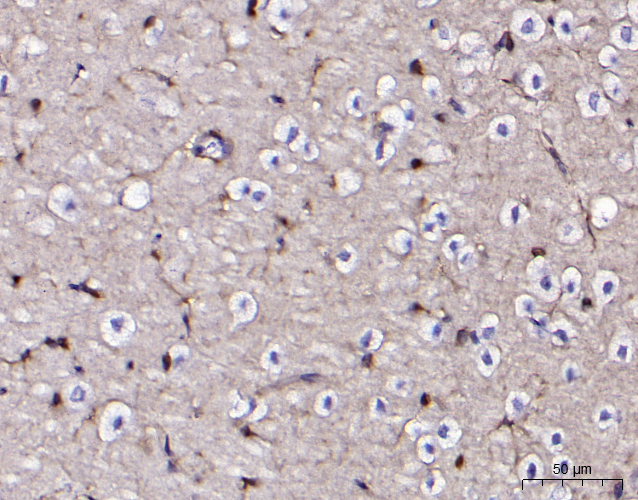

Supplement: Supplementary file 2 [file DataSheet3.ZIP › IHC Raw image of BDNF in PFC(3)/H41 1-100 BDNF_20.0x.tif-Q4.tif]

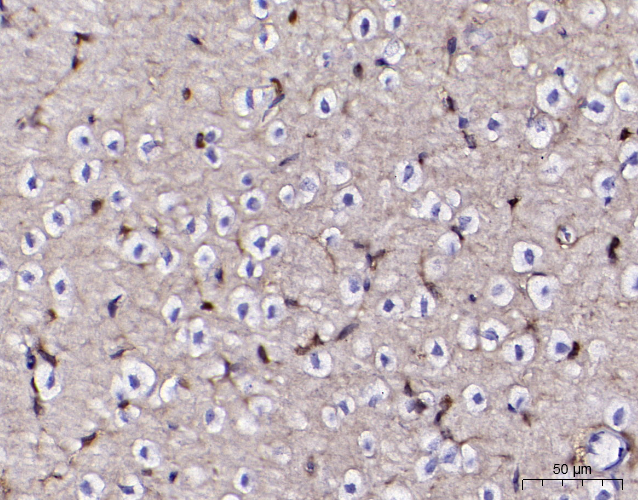

Supplement: Supplementary file 2 [file DataSheet3.ZIP › IHC Raw image of BDNF in PFC(3)/H41 1-100 BDNF_20.0x.tif-Q5.tif]

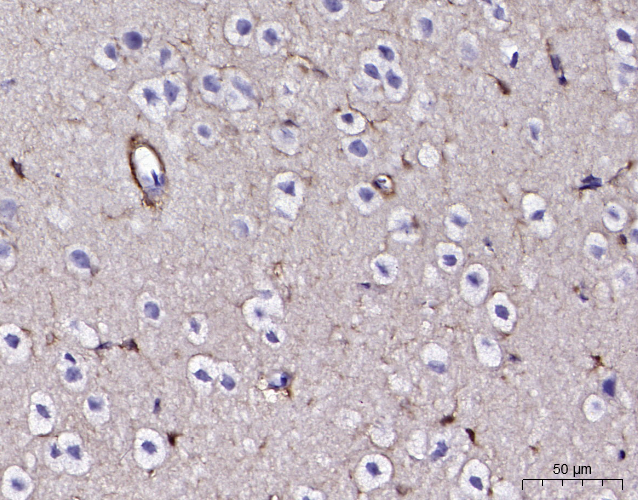

Supplement: Supplementary file 2 [file DataSheet3.ZIP › IHC Raw image of BDNF in PFC(3)/H48 1-100 BDNF_20.0x.tif-Q1.tif]

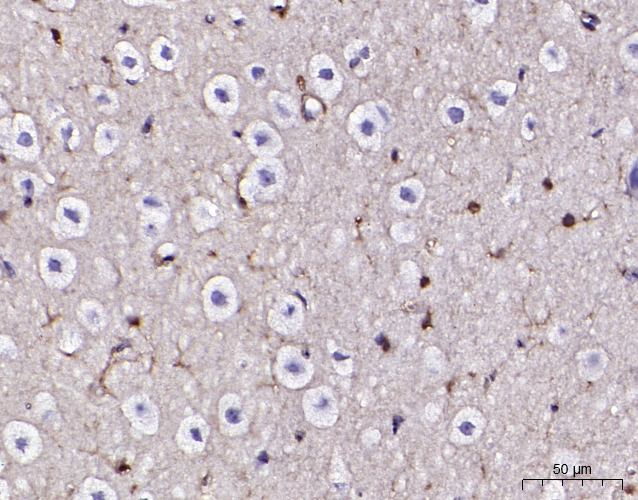

Supplement: Supplementary file 2 [file DataSheet3.ZIP › IHC Raw image of BDNF in PFC(3)/H48 1-100 BDNF_20.0x.tif-Q2.tif]

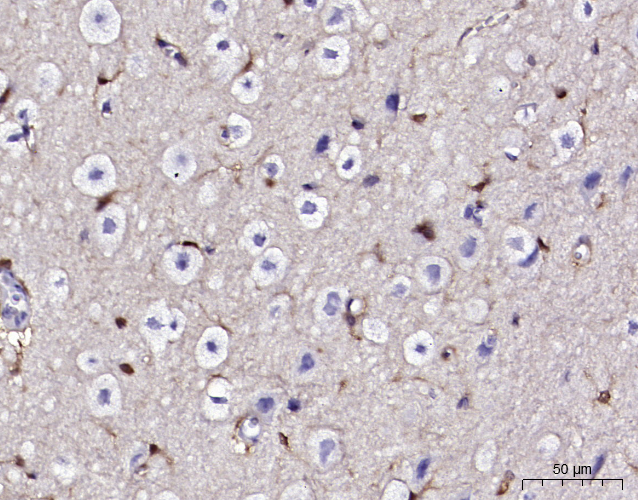

Supplement: Supplementary file 2 [file DataSheet3.ZIP › IHC Raw image of BDNF in PFC(3)/H48 1-100 BDNF_20.0x.tif-Q3.tif]

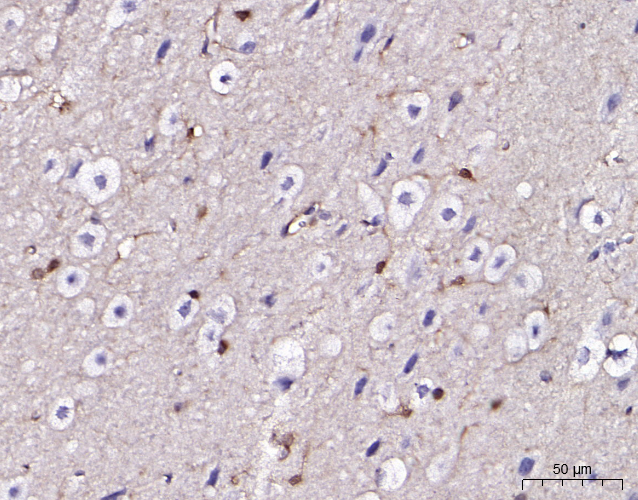

Supplement: Supplementary file 2 [file DataSheet3.ZIP › IHC Raw image of BDNF in PFC(3)/H48 1-100 BDNF_20.0x.tif-Q4.tif]

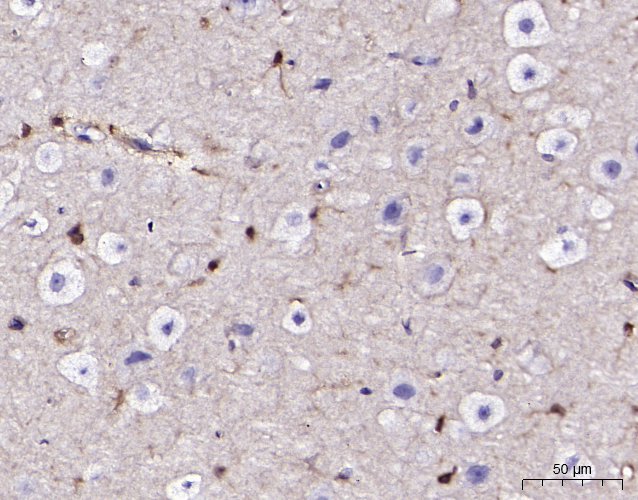

Supplement: Supplementary file 2 [file DataSheet3.ZIP › IHC Raw image of BDNF in PFC(3)/H48 1-100 BDNF_20.0x.tif-Q5.tif]

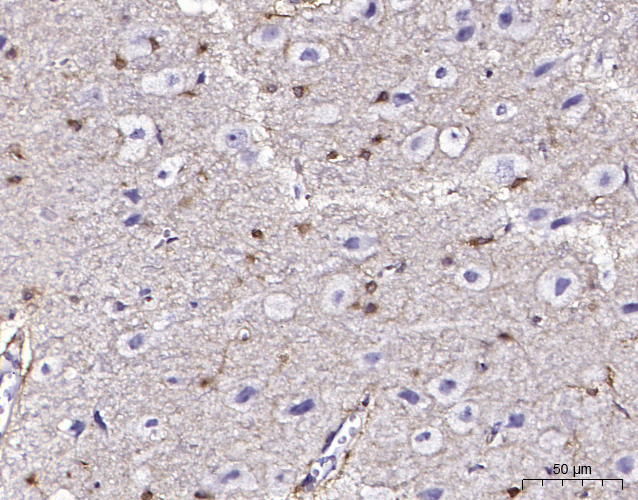

Supplement: Supplementary file 2 [file DataSheet3.ZIP › IHC Raw image of BDNF in PFC(3)/H53 1-100 BDNF_20.0x.tif-Q1.tif]

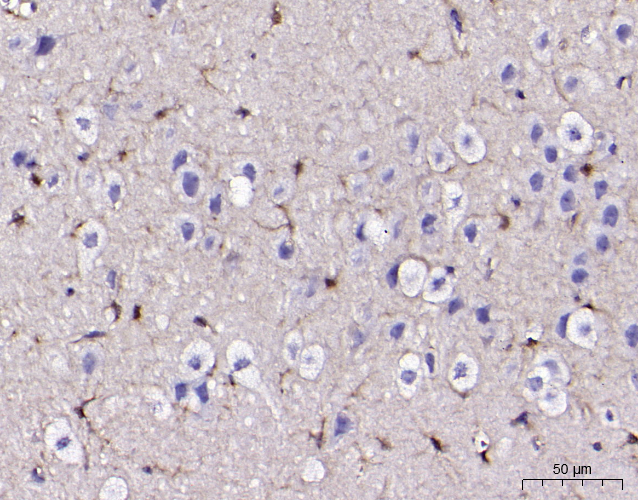

Supplement: Supplementary file 2 [file DataSheet3.ZIP › IHC Raw image of BDNF in PFC(3)/H53 1-100 BDNF_20.0x.tif-Q2.tif]

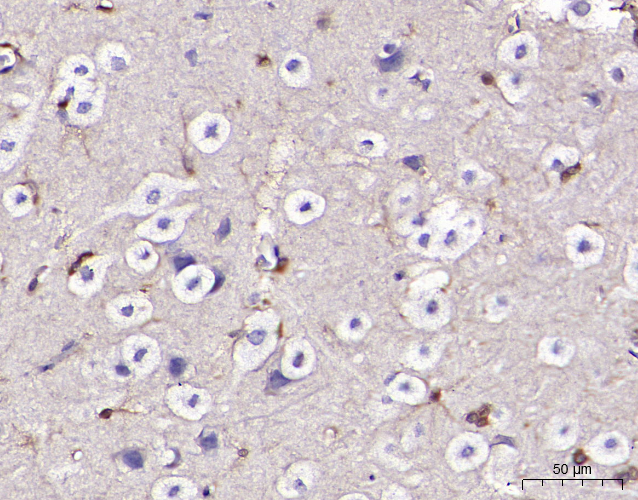

Supplement: Supplementary file 2 [file DataSheet3.ZIP › IHC Raw image of BDNF in PFC(3)/H53 1-100 BDNF_20.0x.tif-Q3.tif]

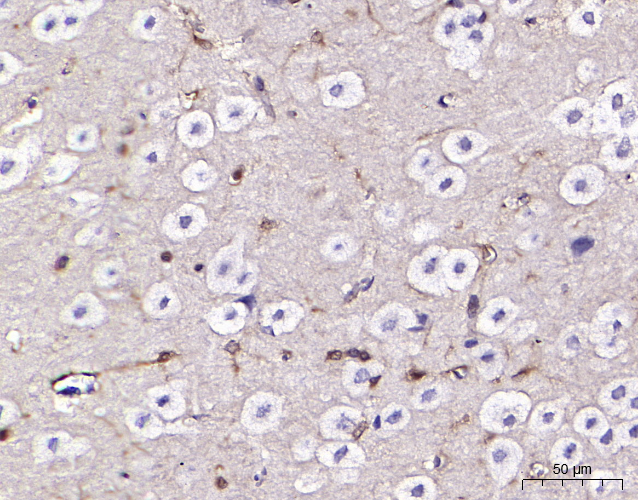

Supplement: Supplementary file 2 [file DataSheet3.ZIP › IHC Raw image of BDNF in PFC(3)/H53 1-100 BDNF_20.0x.tif-Q4.tif]

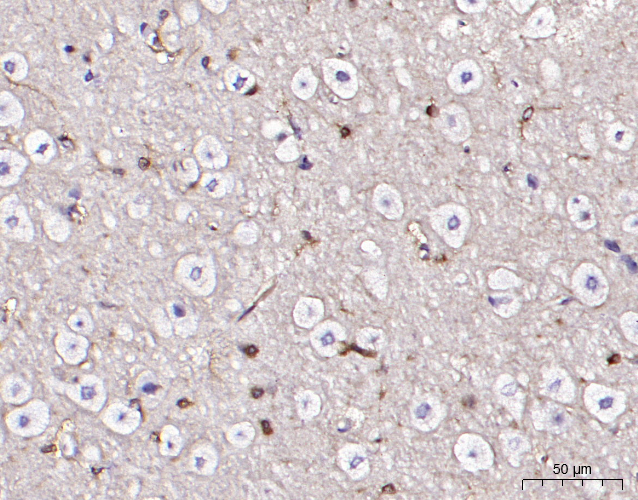

Supplement: Supplementary file 2 [file DataSheet3.ZIP › IHC Raw image of BDNF in PFC(3)/H53 1-100 BDNF_20.0x.tif-Q5.tif]

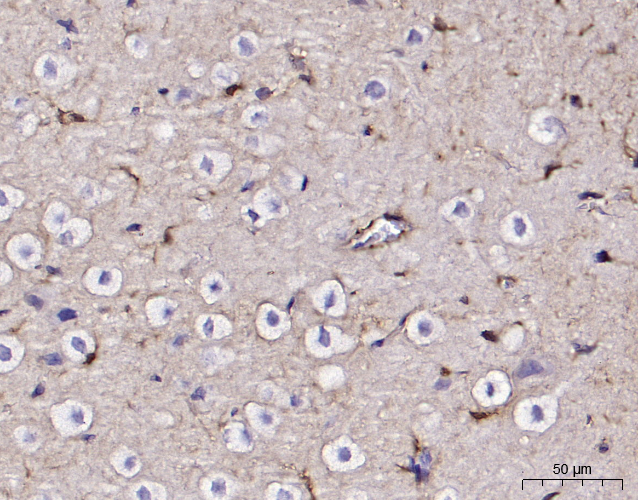

Supplement: Supplementary file 2 [file DataSheet3.ZIP › IHC Raw image of BDNF in PFC(3)/M11 1-100 BDNF_20.0x.tif-Q1.tif]

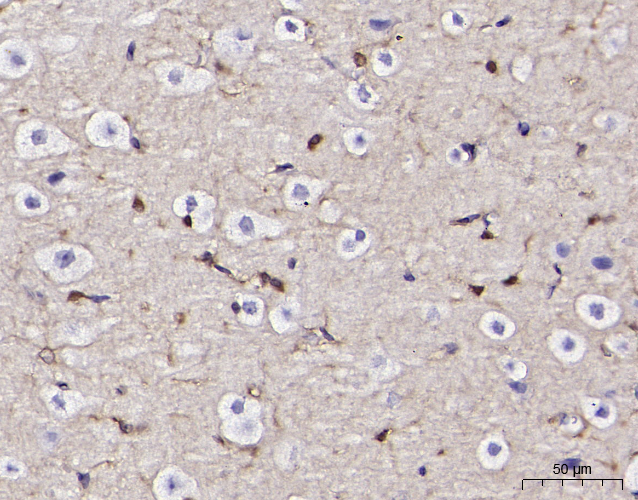

Supplement: Supplementary file 2 [file DataSheet3.ZIP › IHC Raw image of BDNF in PFC(3)/M11 1-100 BDNF_20.0x.tif-Q2.tif]

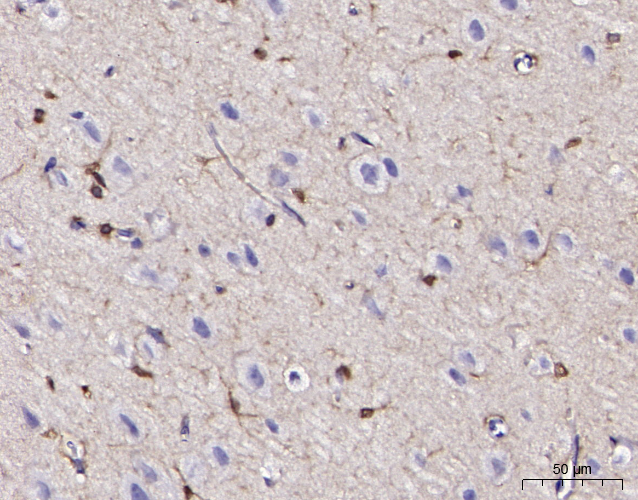

Supplement: Supplementary file 2 [file DataSheet3.ZIP › IHC Raw image of BDNF in PFC(3)/M11 1-100 BDNF_20.0x.tif-Q3.tif]

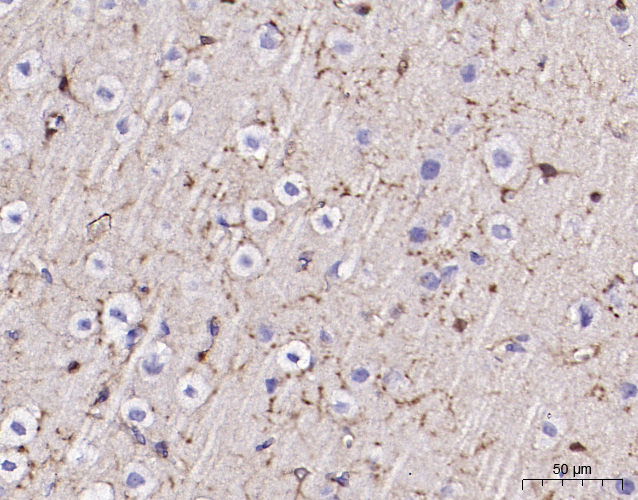

Supplement: Supplementary file 2 [file DataSheet3.ZIP › IHC Raw image of BDNF in PFC(3)/M11 1-100 BDNF_20.0x.tif-Q4.tif]

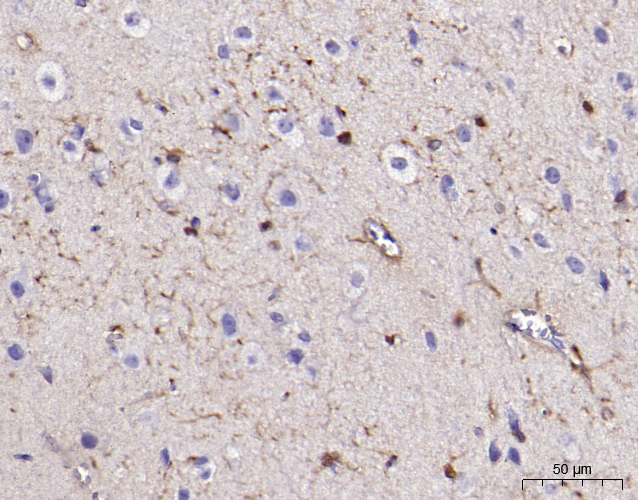

Supplement: Supplementary file 2 [file DataSheet3.ZIP › IHC Raw image of BDNF in PFC(3)/M11 1-100 BDNF_20.0x.tif-Q5.tif]

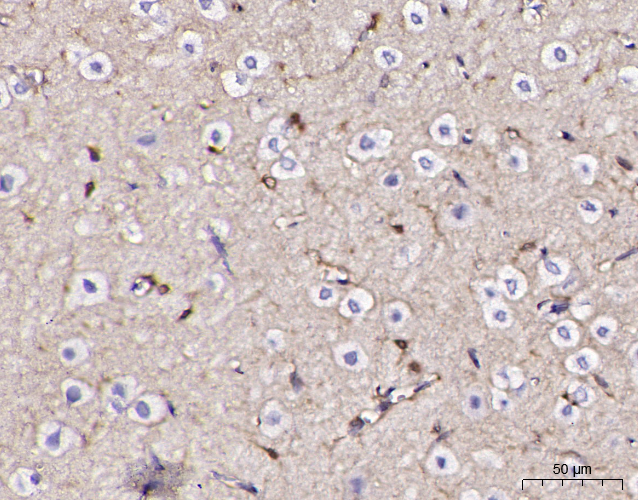

Supplement: Supplementary file 2 [file DataSheet3.ZIP › IHC Raw image of BDNF in PFC(3)/M27 1-100 BDNF_20.0x.tif-Q1.tif]

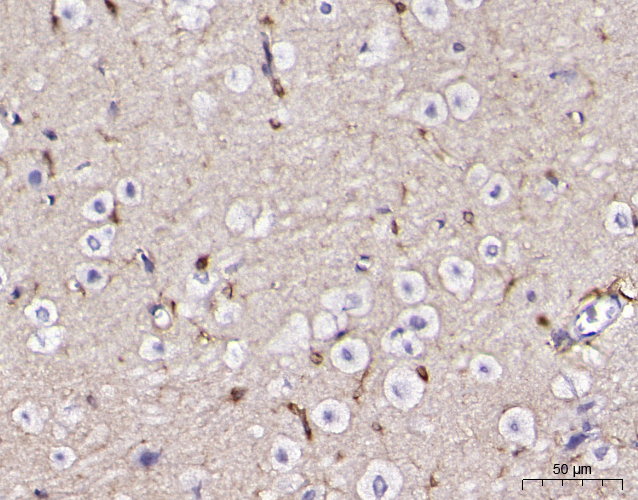

Supplement: Supplementary file 2 [file DataSheet3.ZIP › IHC Raw image of BDNF in PFC(3)/M27 1-100 BDNF_20.0x.tif-Q2.tif]

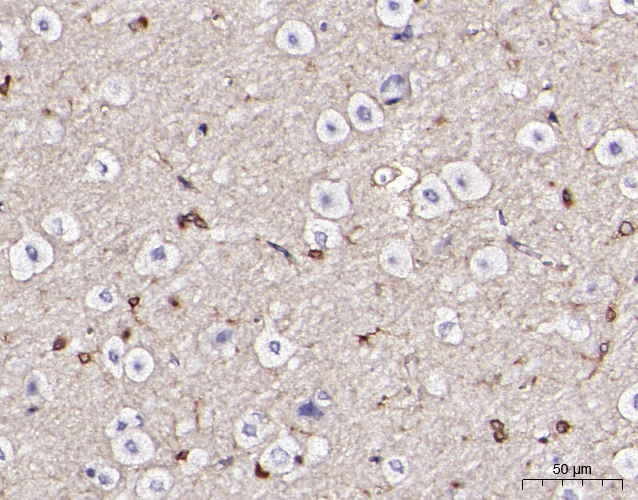

Supplement: Supplementary file 2 [file DataSheet3.ZIP › IHC Raw image of BDNF in PFC(3)/M27 1-100 BDNF_20.0x.tif-Q3.tif]

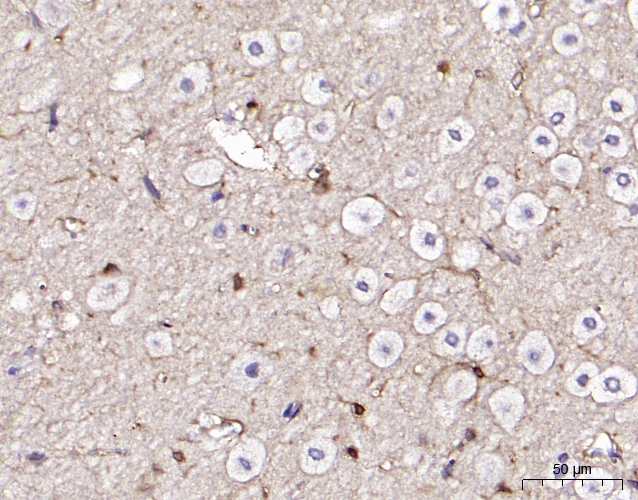

Supplement: Supplementary file 2 [file DataSheet3.ZIP › IHC Raw image of BDNF in PFC(3)/M27 1-100 BDNF_20.0x.tif-Q4.tif]

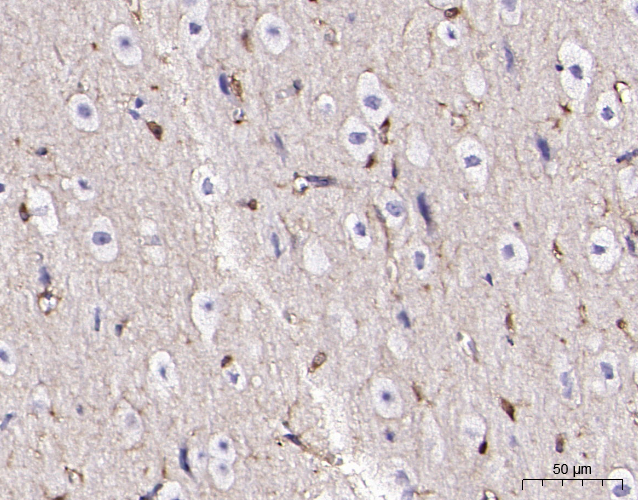

Supplement: Supplementary file 2 [file DataSheet3.ZIP › IHC Raw image of BDNF in PFC(3)/M27 1-100 BDNF_20.0x.tif-Q5.tif]

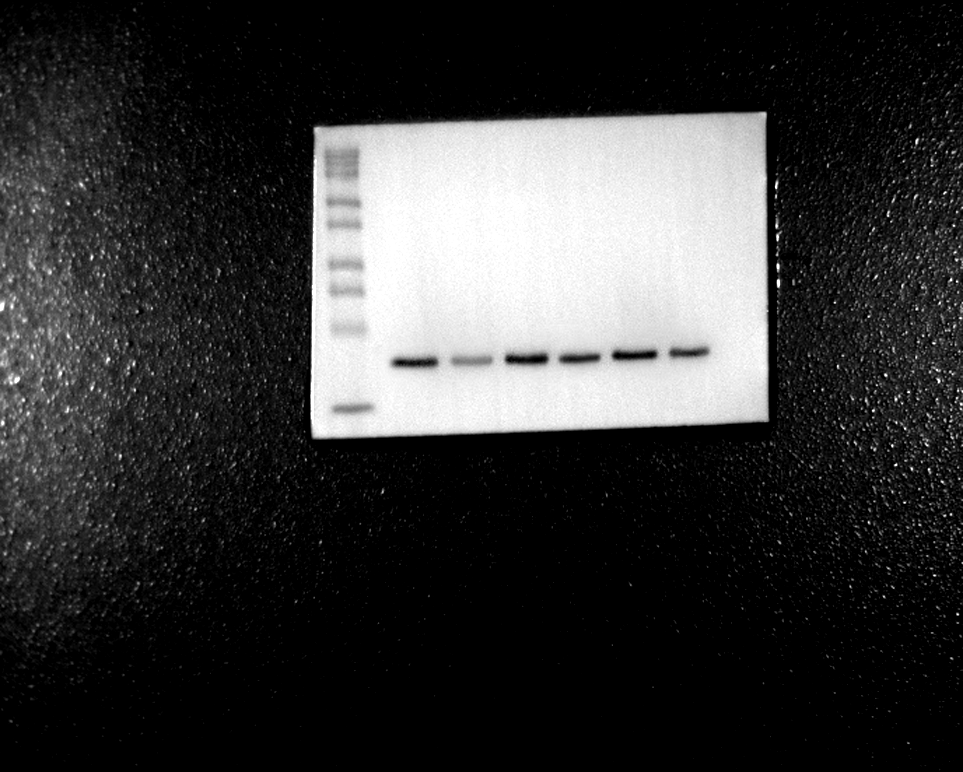

Supplement: Supplementary file 3 [file DataSheet14.ZIP › Raw image of Western blot in PFC/BDNF (1).tif]

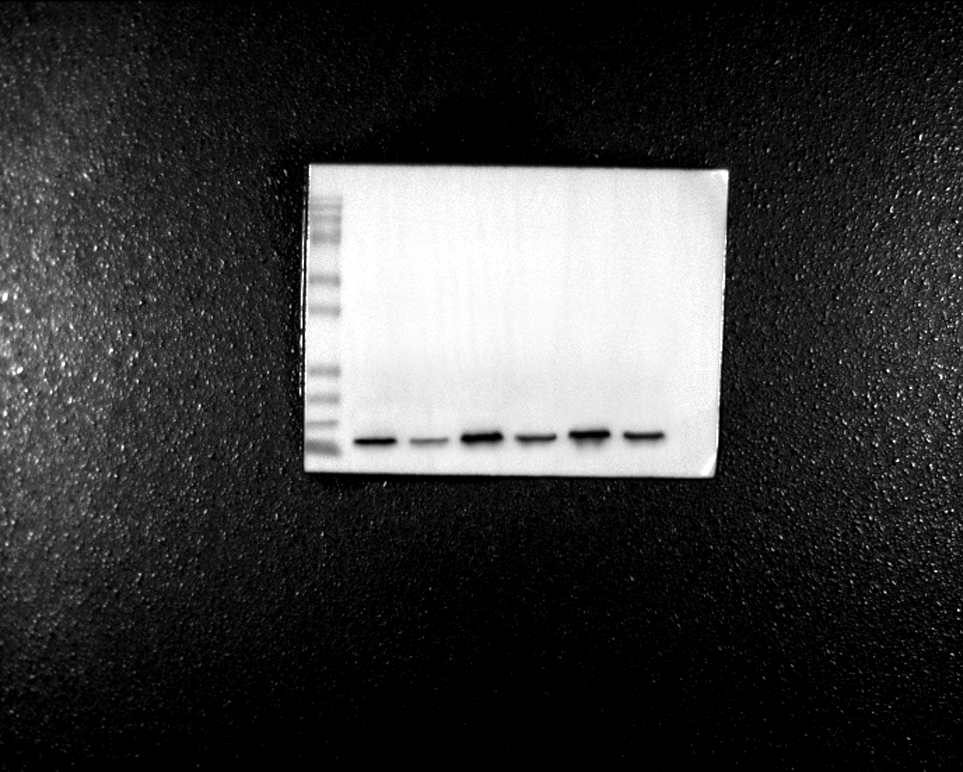

Supplement: Supplementary file 3 [file DataSheet14.ZIP › Raw image of Western blot in PFC/BDNF (2).tif]

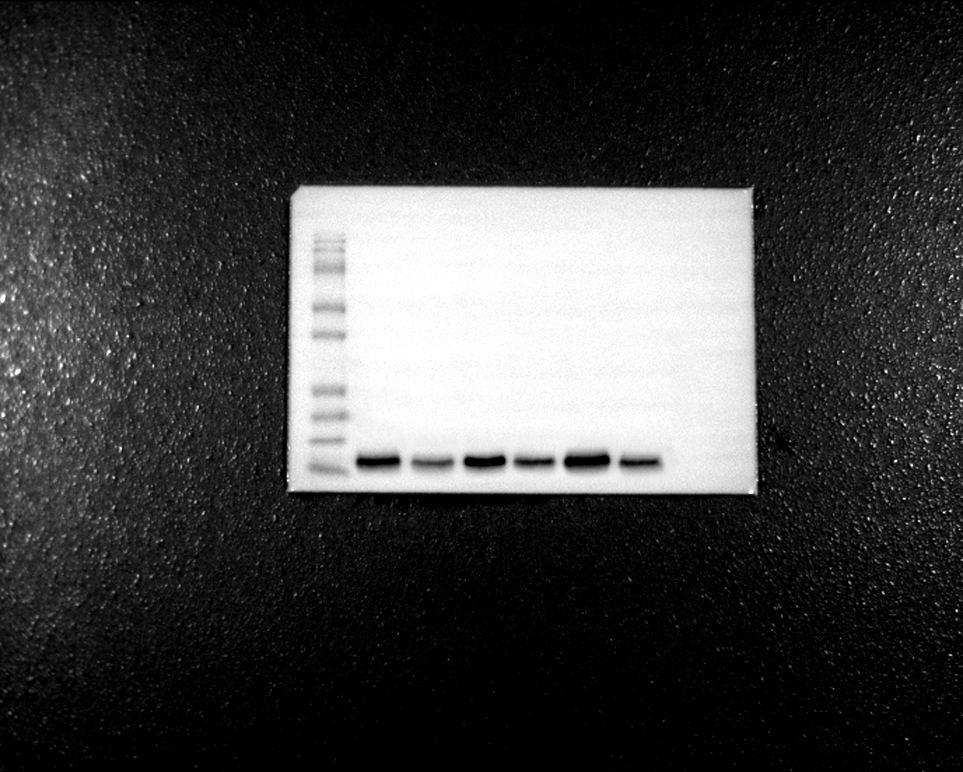

Supplement: Supplementary file 3 [file DataSheet14.ZIP › Raw image of Western blot in PFC/BDNF (3).tif]

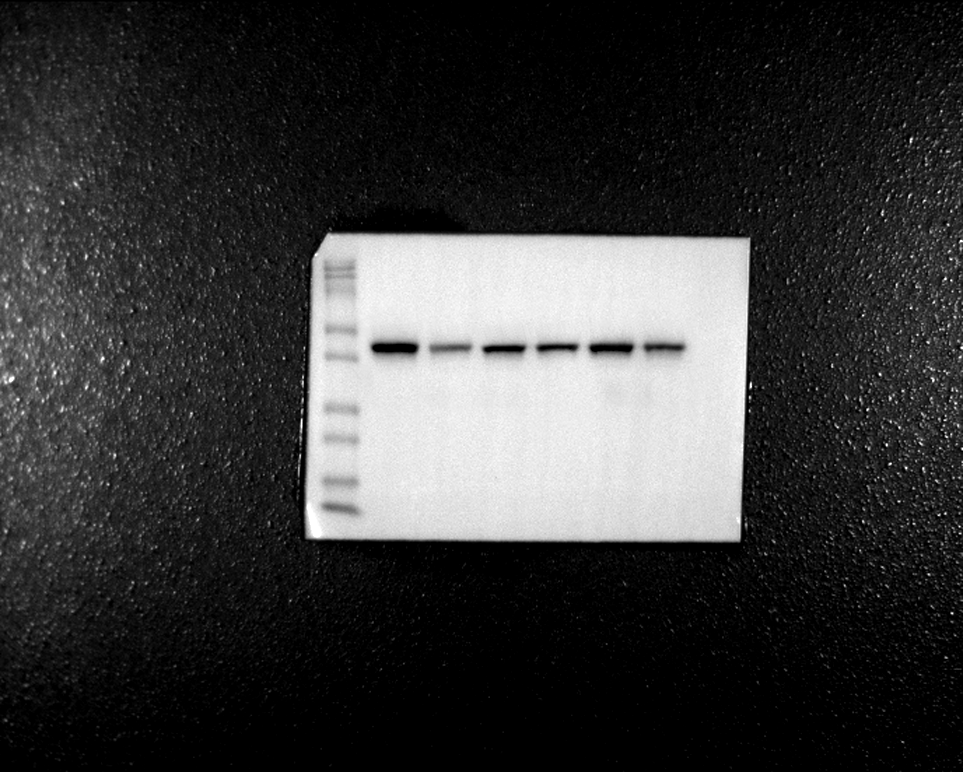

Supplement: Supplementary file 3 [file DataSheet14.ZIP › Raw image of Western blot in PFC/DRD1 (1).tif]

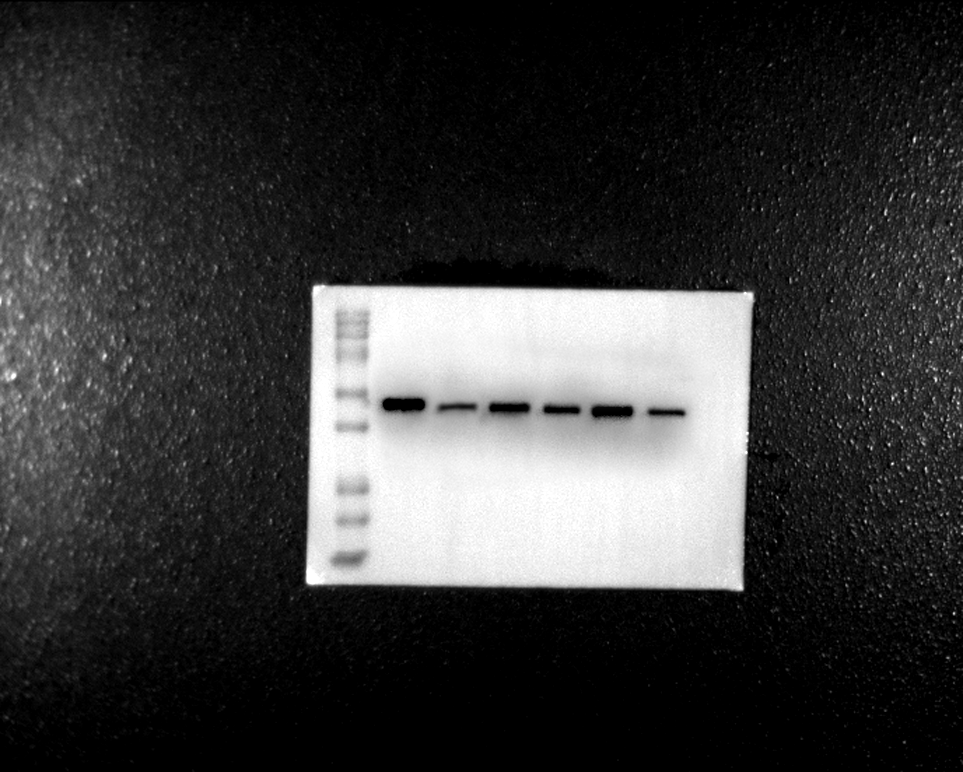

Supplement: Supplementary file 3 [file DataSheet14.ZIP › Raw image of Western blot in PFC/DRD1 (2).tif]

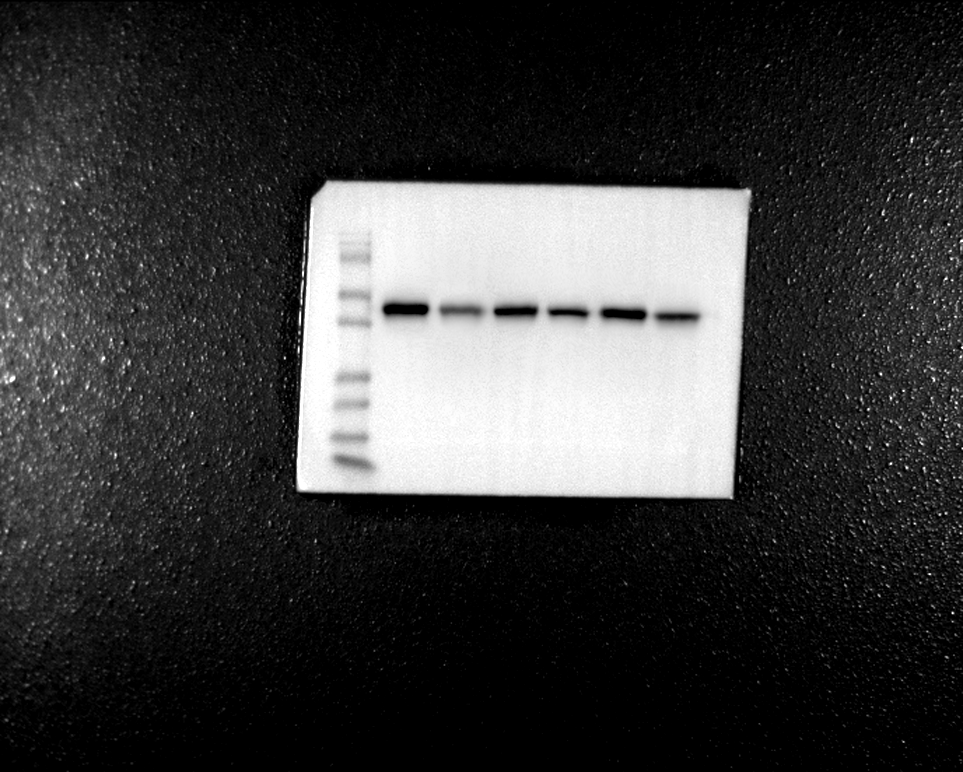

Supplement: Supplementary file 3 [file DataSheet14.ZIP › Raw image of Western blot in PFC/DRD1 (3).tif]

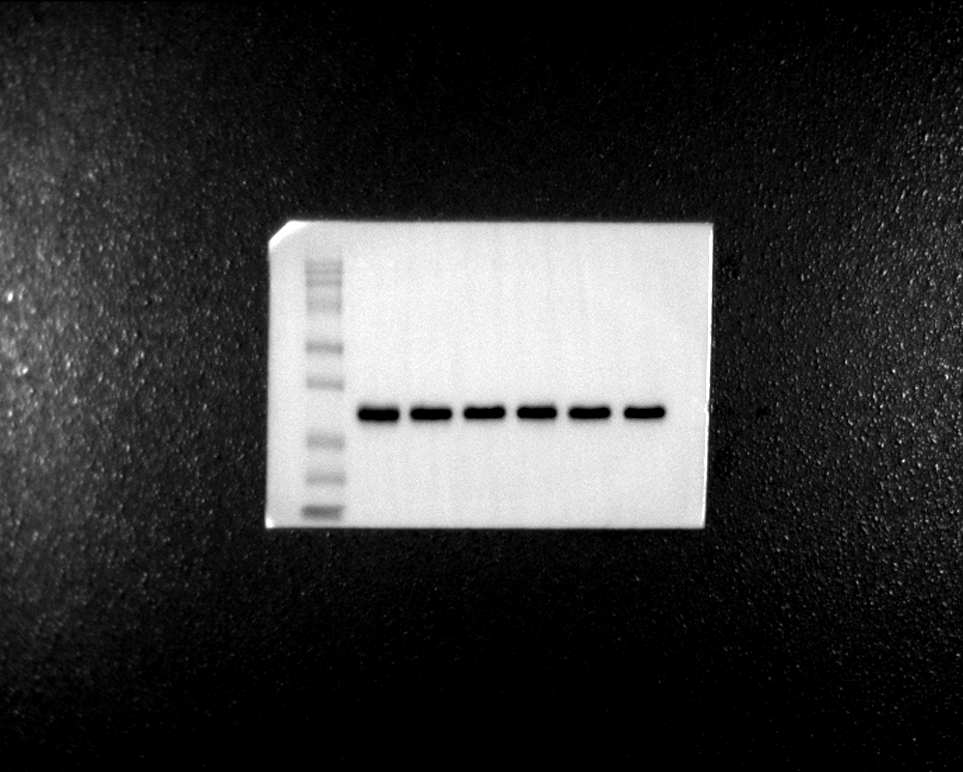

Supplement: Supplementary file 3 [file DataSheet14.ZIP › Raw image of Western blot in PFC/GAPDH (1).tif]

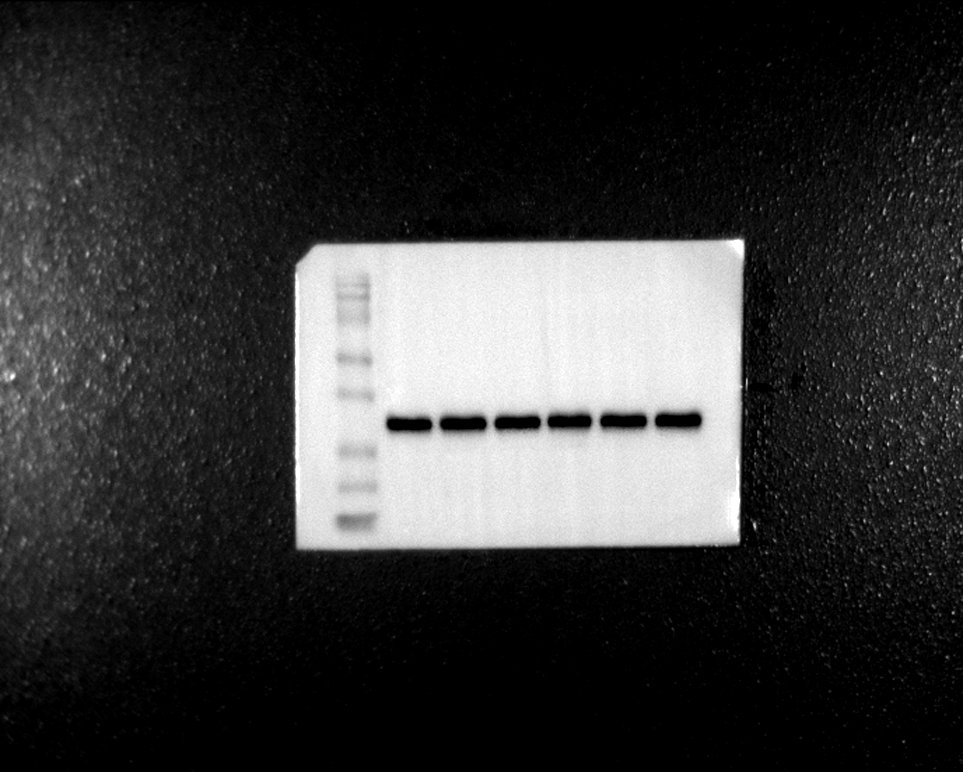

Supplement: Supplementary file 3 [file DataSheet14.ZIP › Raw image of Western blot in PFC/GAPDH (2).tif]

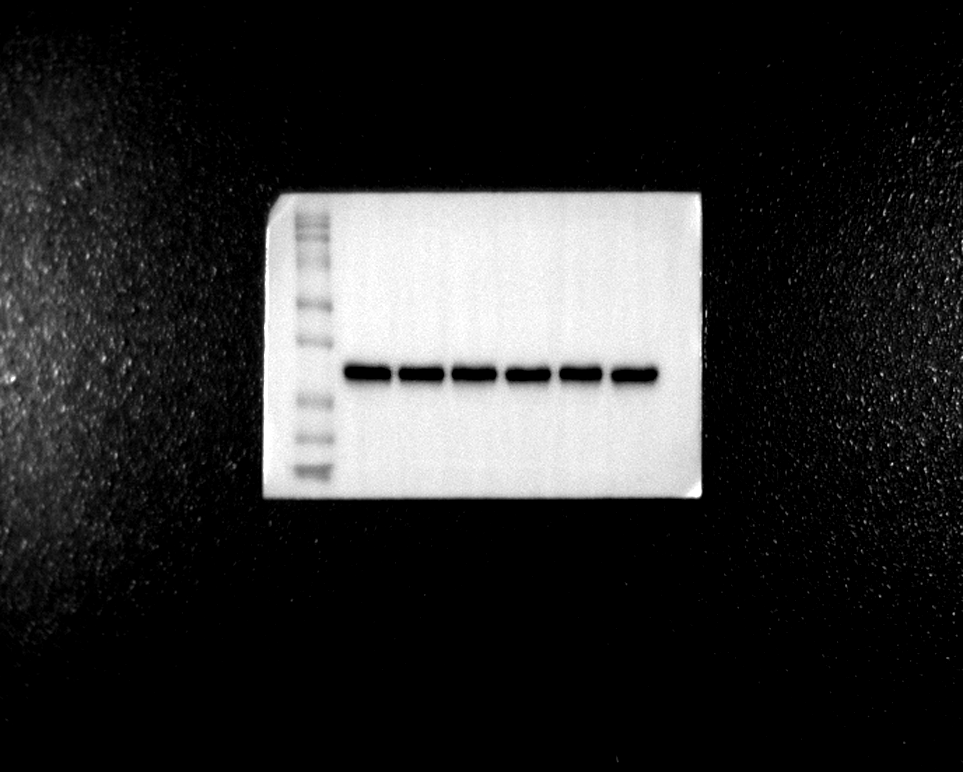

Supplement: Supplementary file 3 [file DataSheet14.ZIP › Raw image of Western blot in PFC/GAPDH (3).tif]

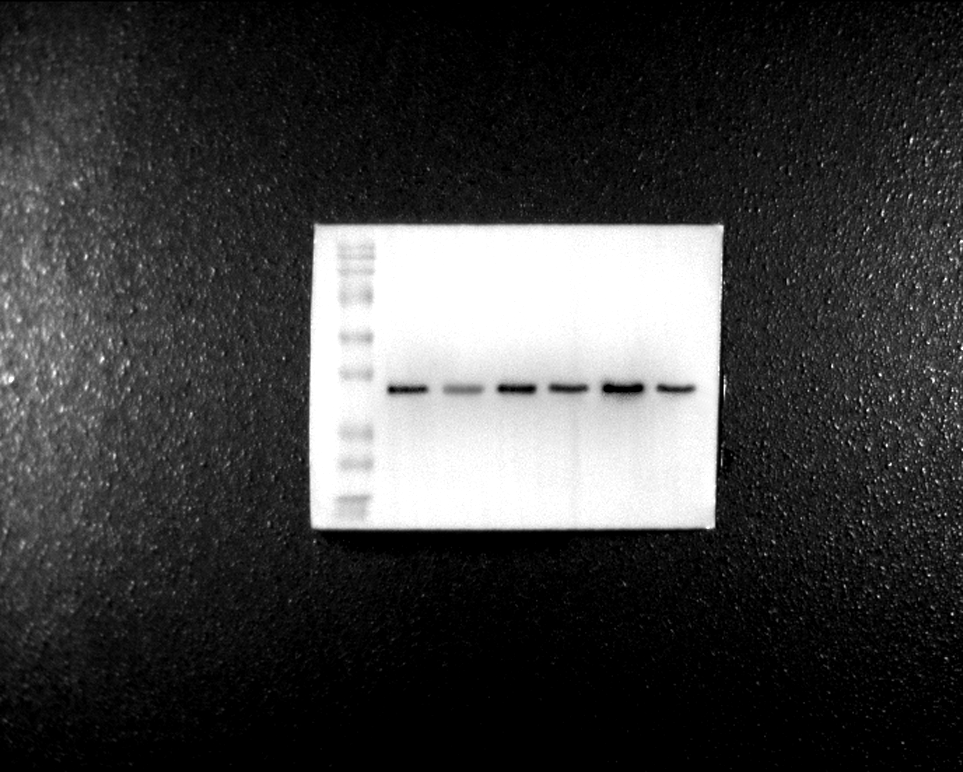

Supplement: Supplementary file 3 [file DataSheet14.ZIP › Raw image of Western blot in PFC/Ga┴olf (1).tif]

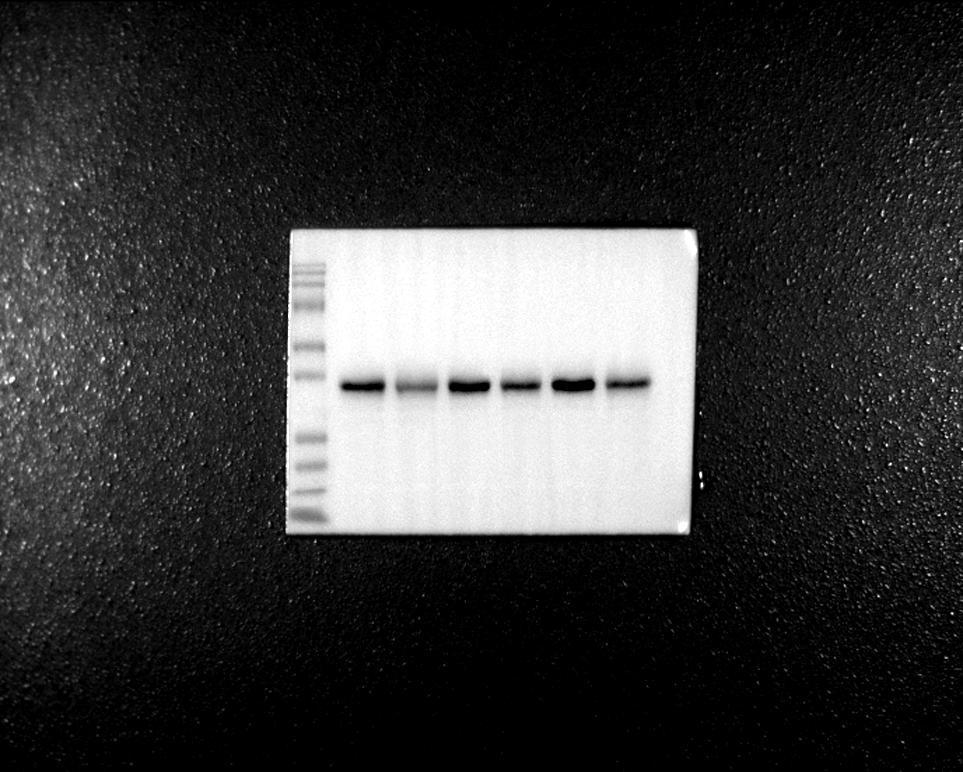

Supplement: Supplementary file 3 [file DataSheet14.ZIP › Raw image of Western blot in PFC/Ga┴olf (2).tif]

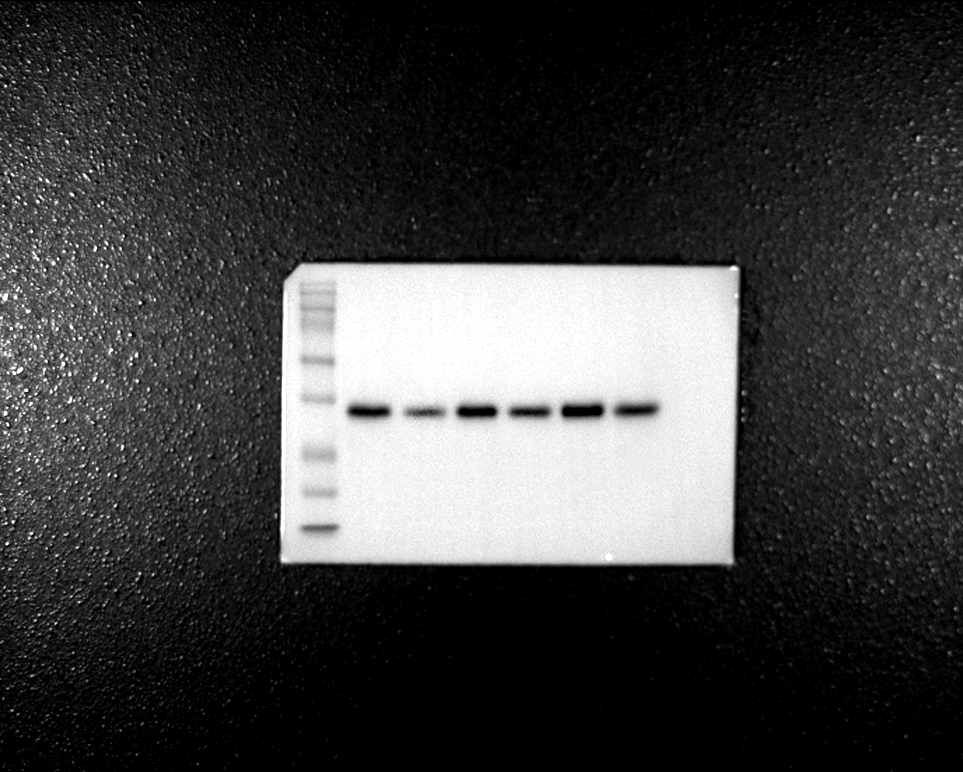

Supplement: Supplementary file 3 [file DataSheet14.ZIP › Raw image of Western blot in PFC/Ga┴olf (3).tif]

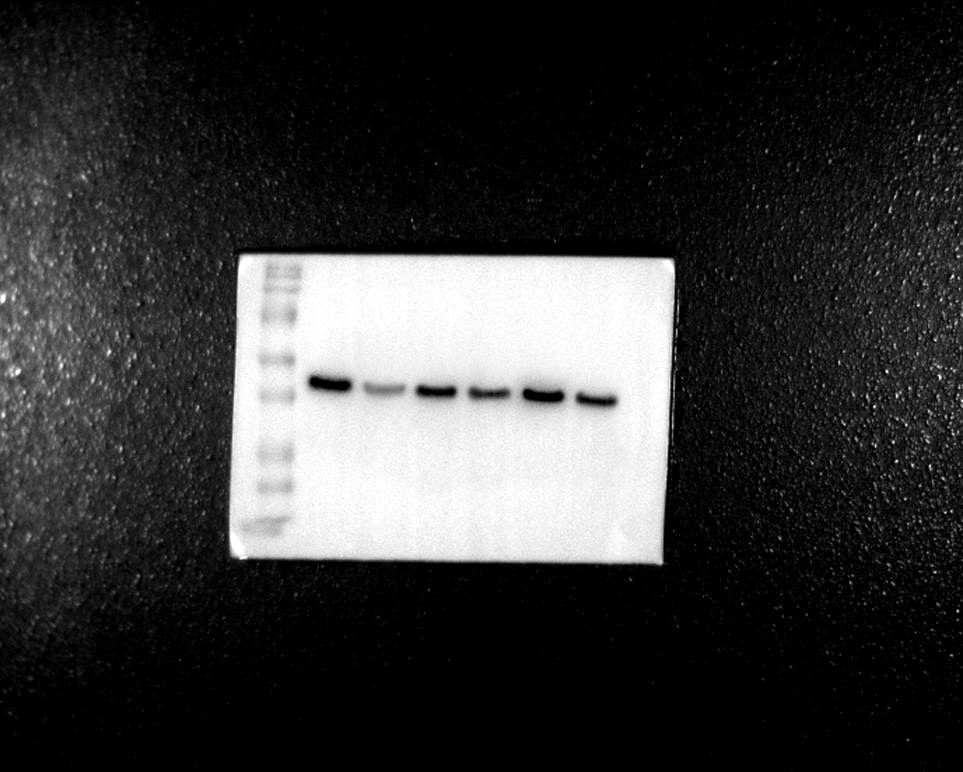

Supplement: Supplementary file 3 [file DataSheet14.ZIP › Raw image of Western blot in PFC/Ga┴s (1).tif]

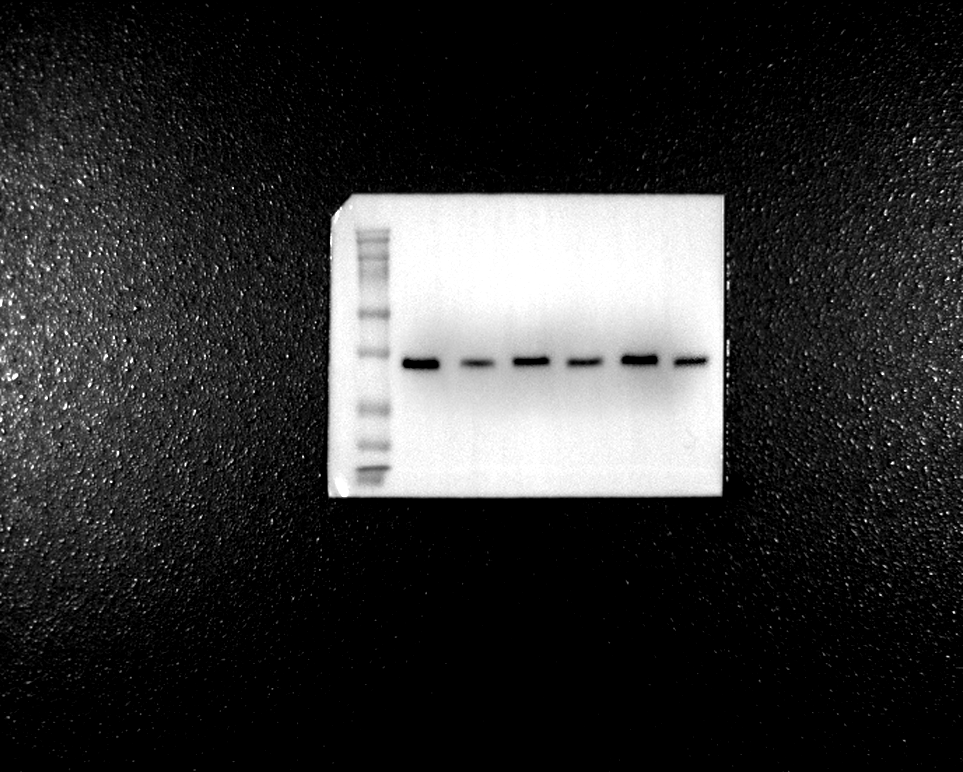

Supplement: Supplementary file 3 [file DataSheet14.ZIP › Raw image of Western blot in PFC/Ga┴s (2).tif]

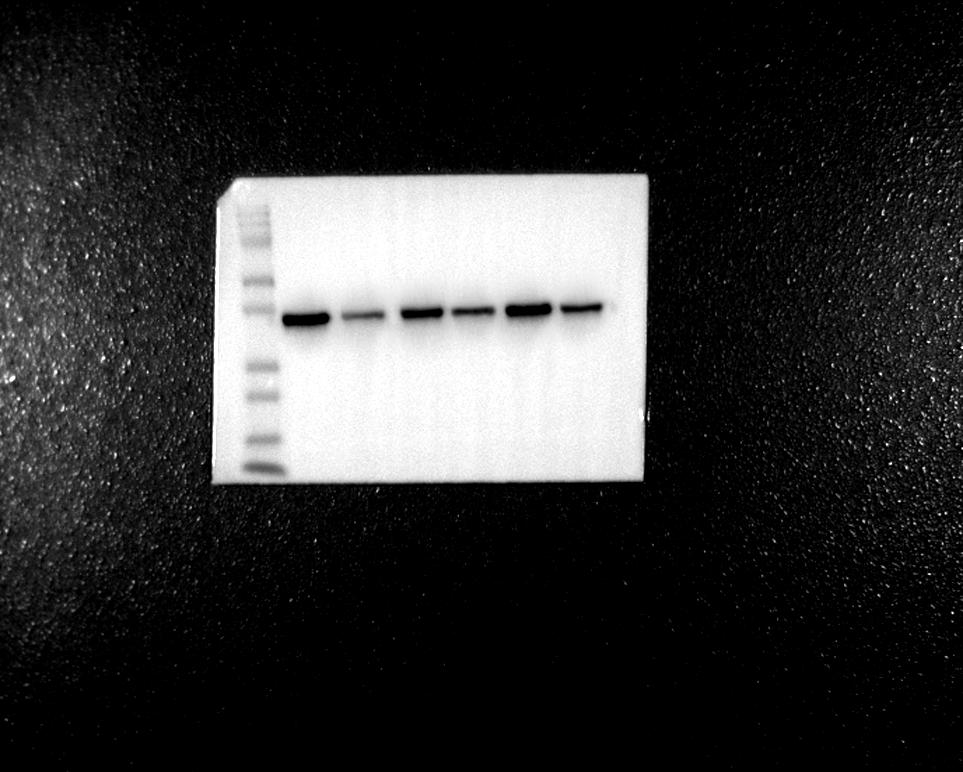

Supplement: Supplementary file 3 [file DataSheet14.ZIP › Raw image of Western blot in PFC/Ga┴s (3).tif]

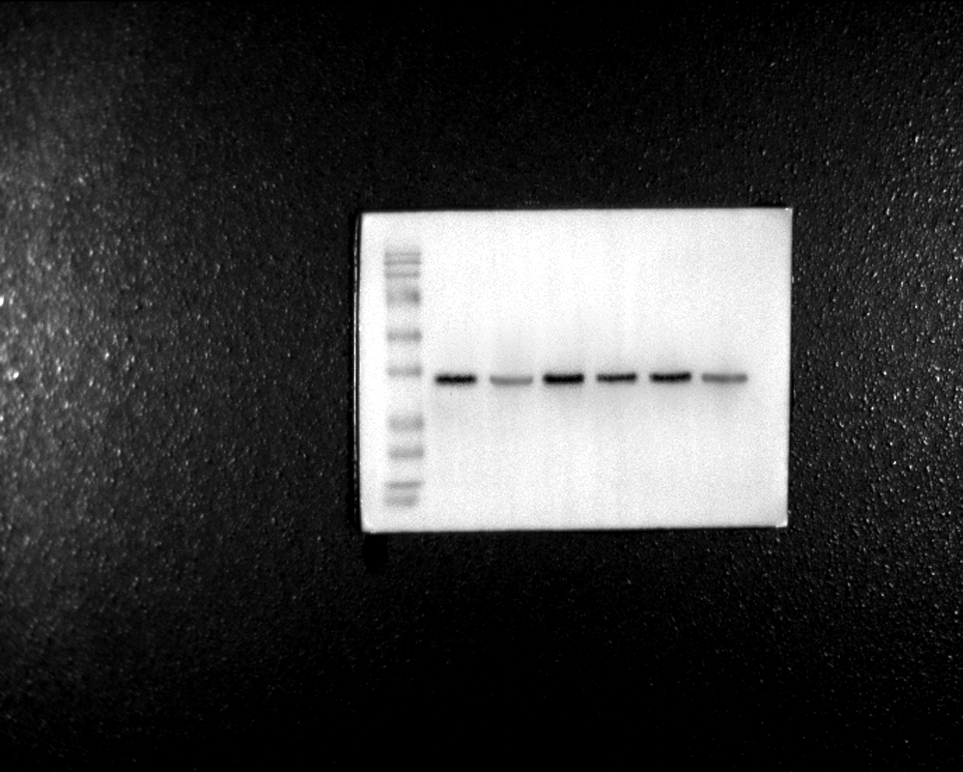

Supplement: Supplementary file 3 [file DataSheet14.ZIP › Raw image of Western blot in PFC/p-CREB (1).tif]

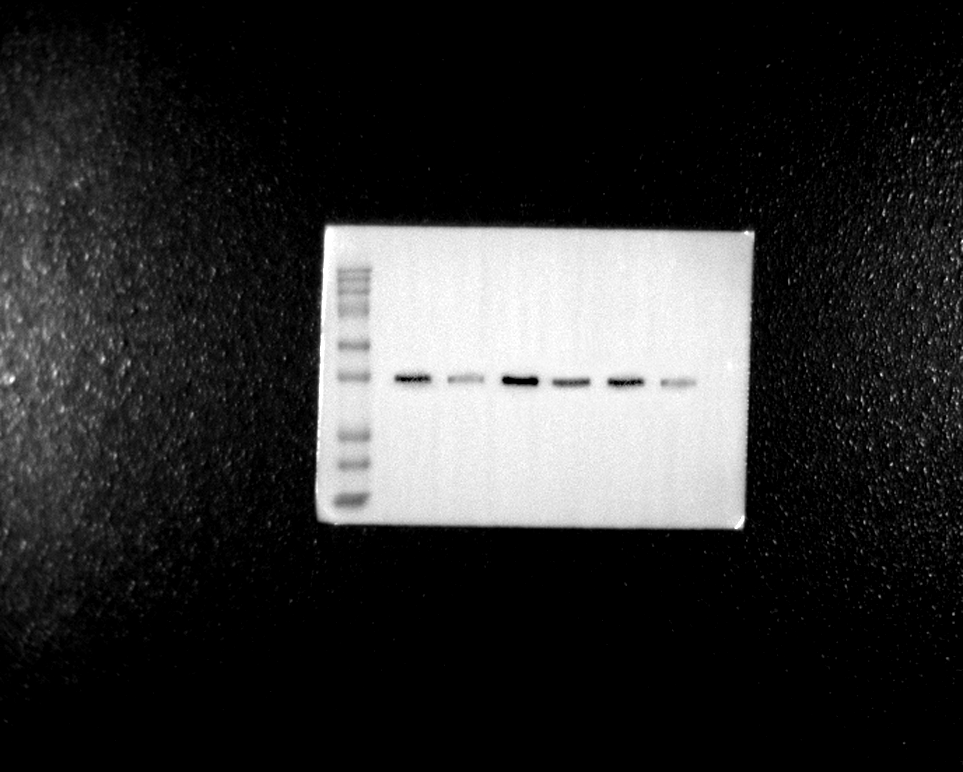

Supplement: Supplementary file 3 [file DataSheet14.ZIP › Raw image of Western blot in PFC/P-CREB (2).tif]

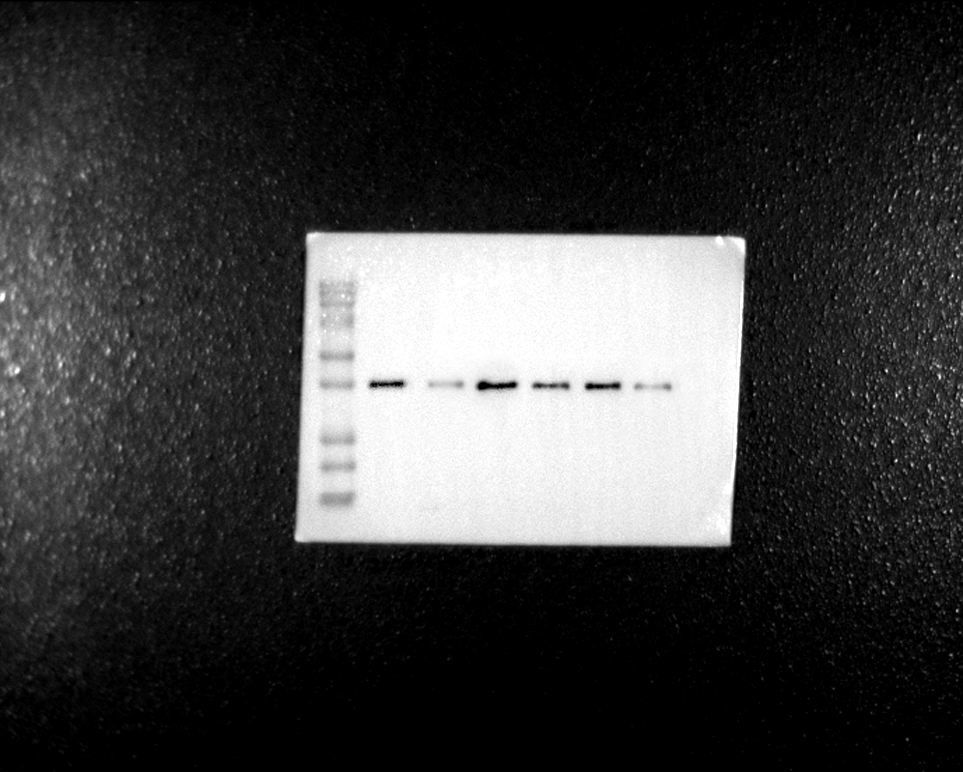

Supplement: Supplementary file 3 [file DataSheet14.ZIP › Raw image of Western blot in PFC/P-CREB (3).tif]

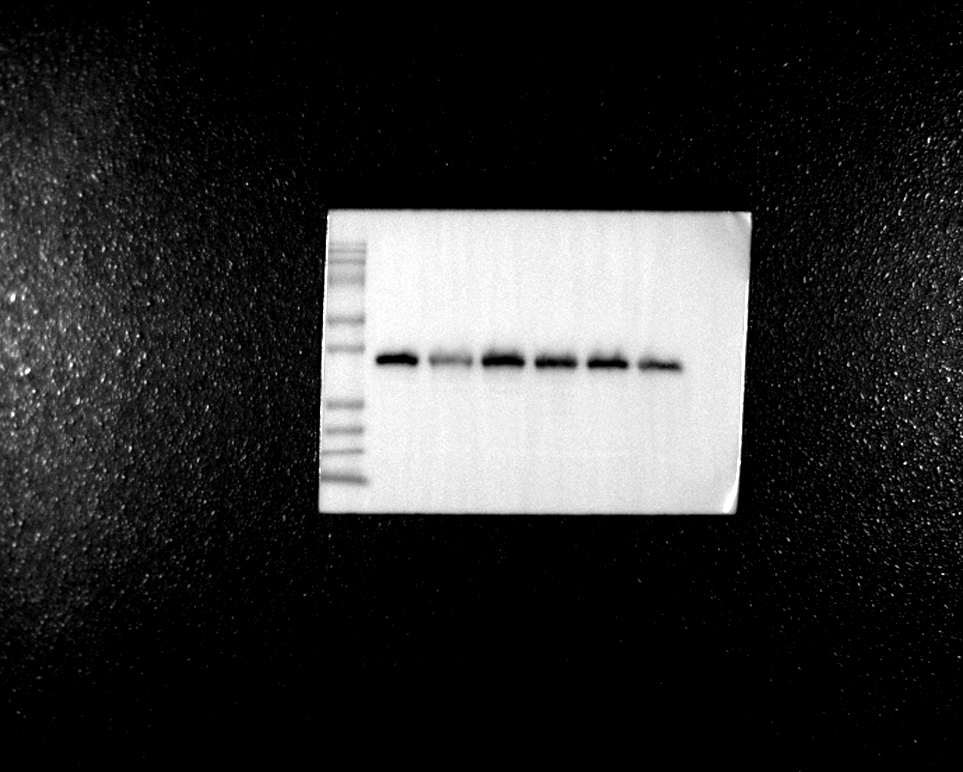

Supplement: Supplementary file 3 [file DataSheet14.ZIP › Raw image of Western blot in PFC/PKA (1).tif]

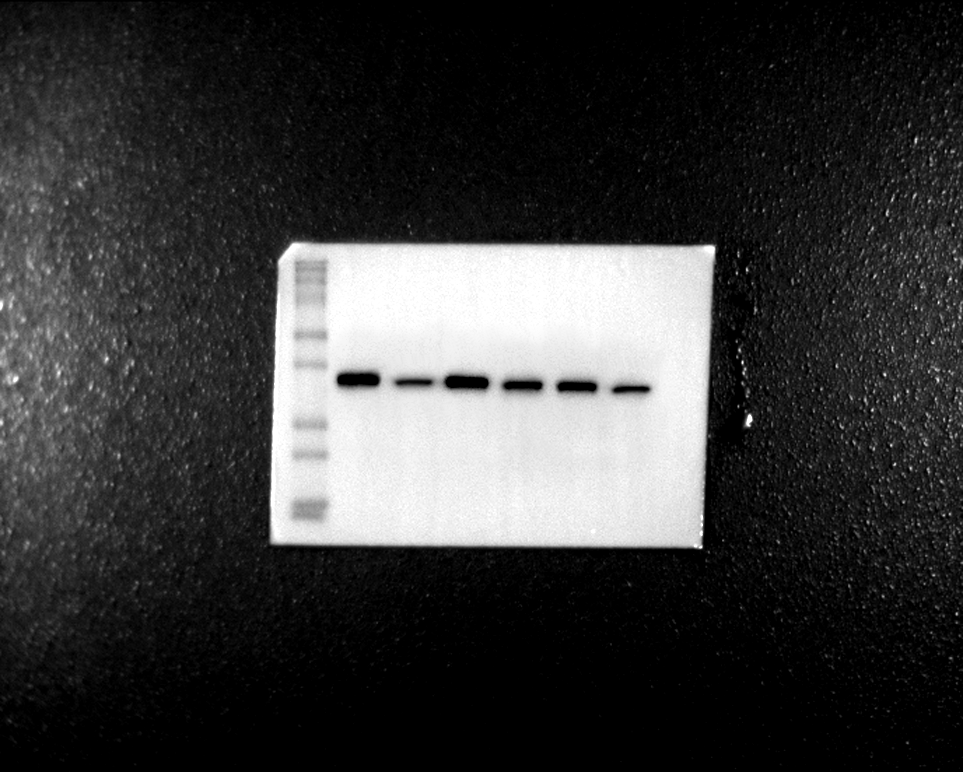

Supplement: Supplementary file 3 [file DataSheet14.ZIP › Raw image of Western blot in PFC/PKA (2).tif]

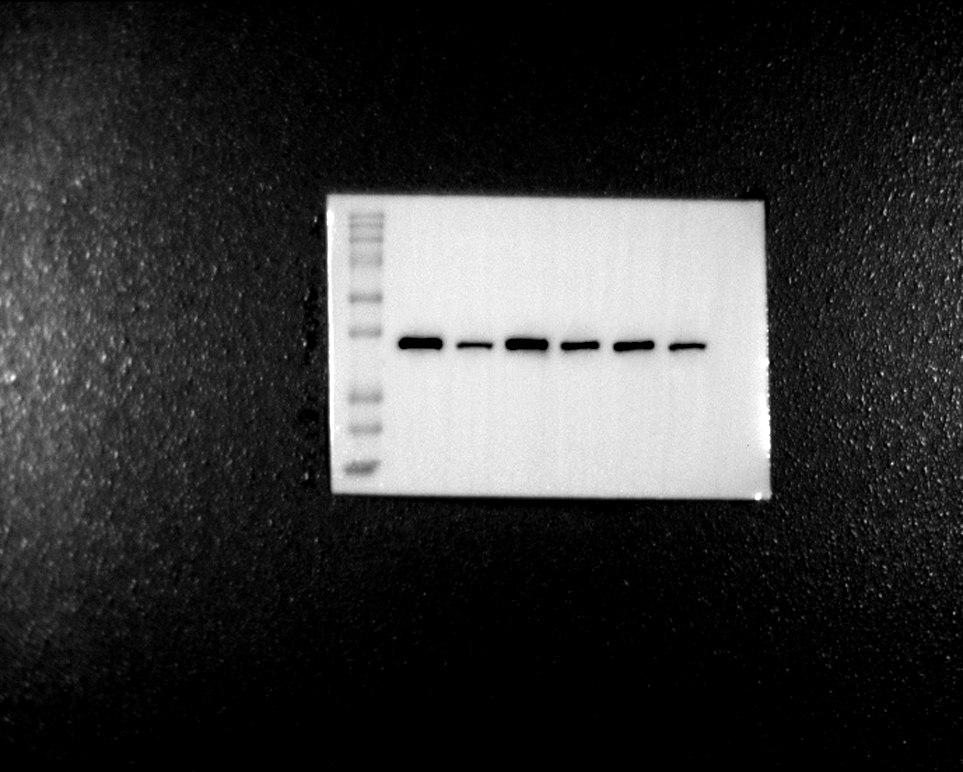

Supplement: Supplementary file 3 [file DataSheet14.ZIP › Raw image of Western blot in PFC/PKA (3).tif]

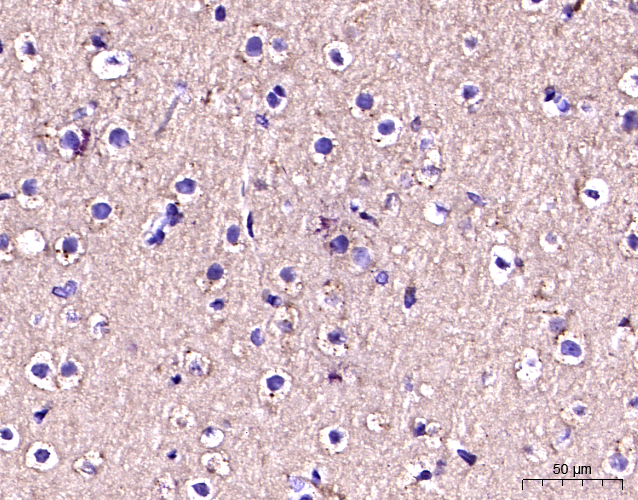

Supplement: Supplementary file 4 [file DataSheet11.ZIP › IHC Raw Image of PKA in PFC (1)/H34 1-200 PKA_20.0x.tif]

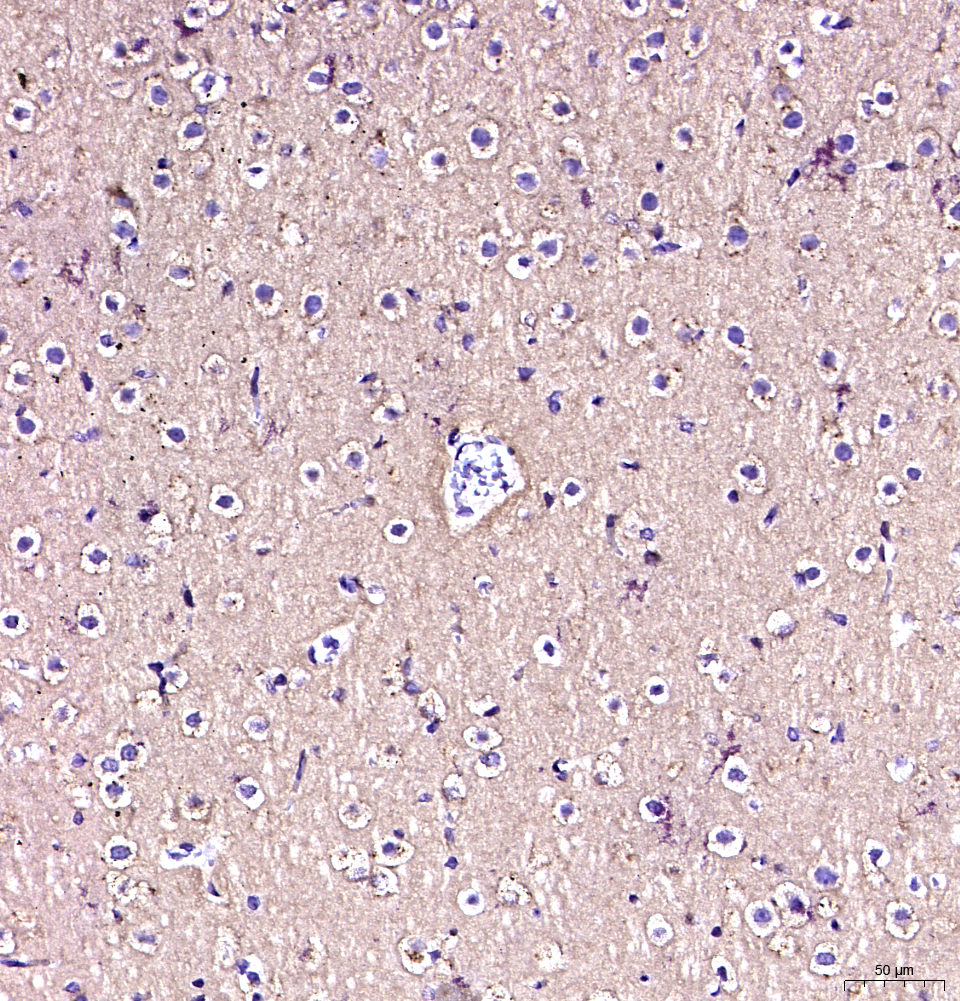

Supplement: Supplementary file 4 [file DataSheet11.ZIP › IHC Raw Image of PKA in PFC (1)/H34 1-200 PKA_20.0x-Q1.tif]

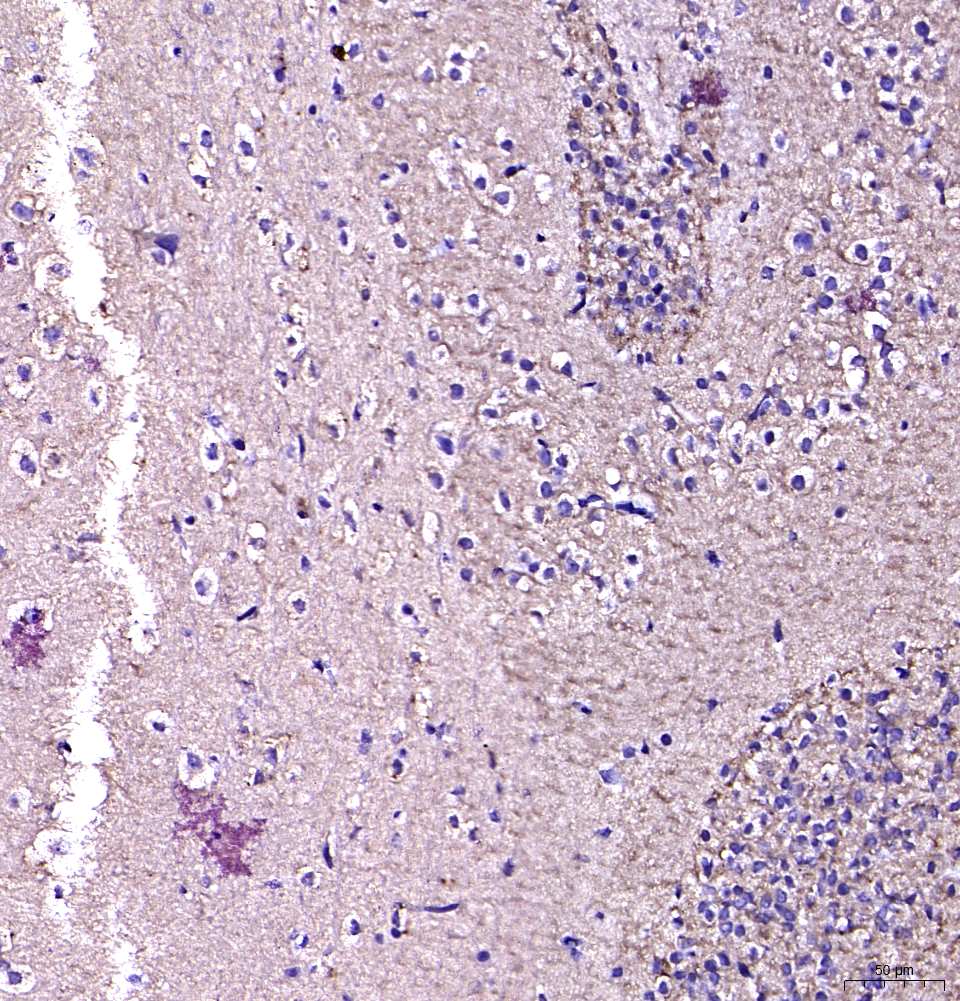

Supplement: Supplementary file 4 [file DataSheet11.ZIP › IHC Raw Image of PKA in PFC (1)/H48 1-200 PKA_20.0x-Q4.tif]

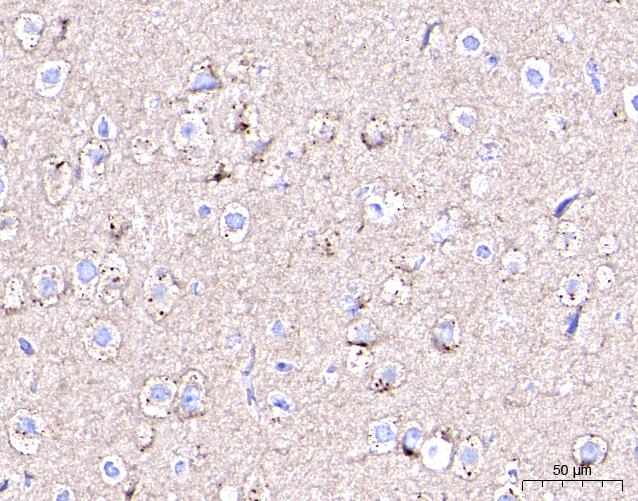

Supplement: Supplementary file 4 [file DataSheet11.ZIP › IHC Raw Image of PKA in PFC (1)/K61 1-200 PKA_20.0x.tif]

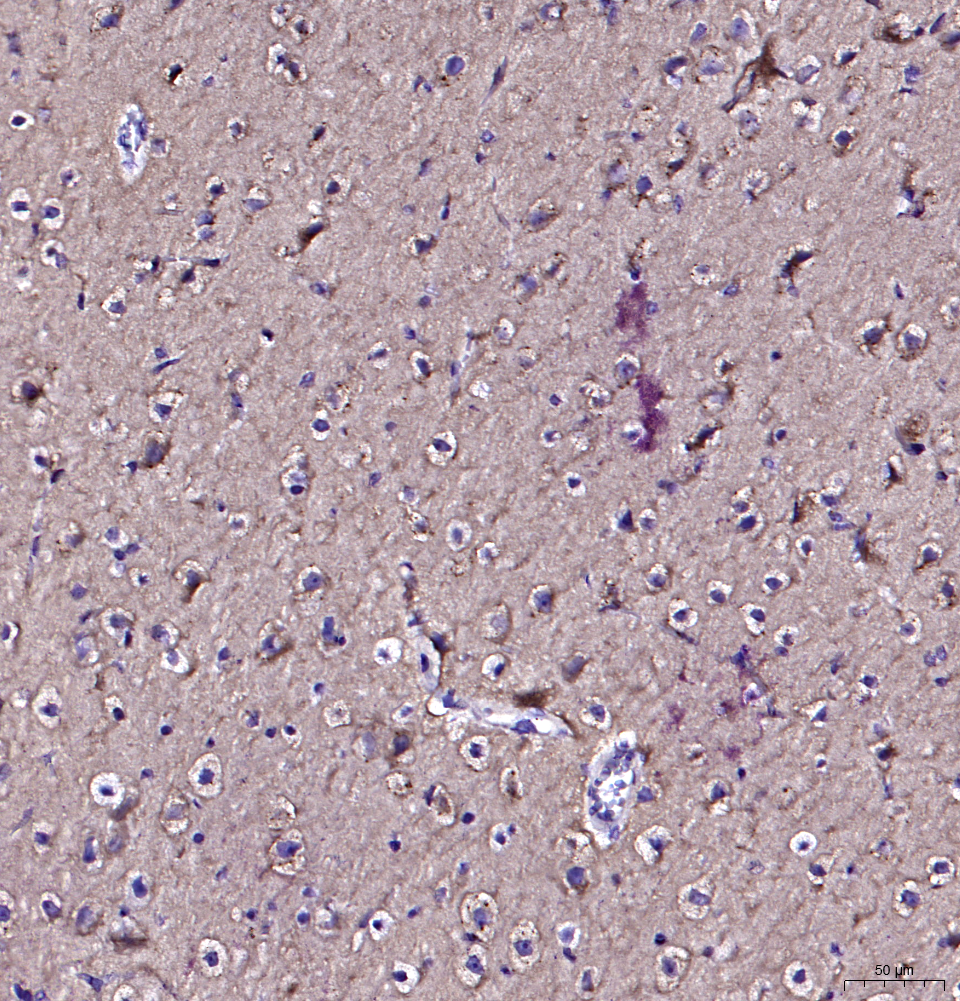

Supplement: Supplementary file 4 [file DataSheet11.ZIP › IHC Raw Image of PKA in PFC (1)/K65 1-200 PKA_20.0x-Q1.tif]

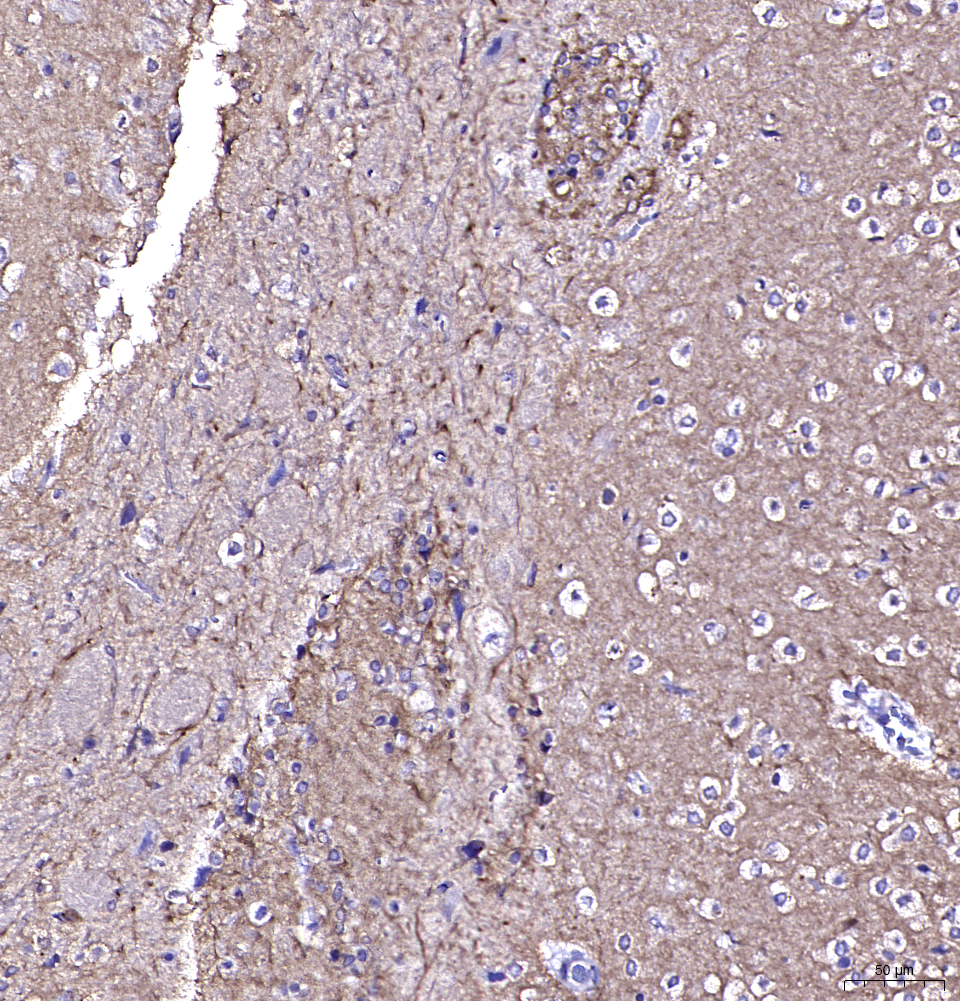

Supplement: Supplementary file 4 [file DataSheet11.ZIP › IHC Raw Image of PKA in PFC (1)/K69 1-200 PKA_20.0x-Q1.tif]

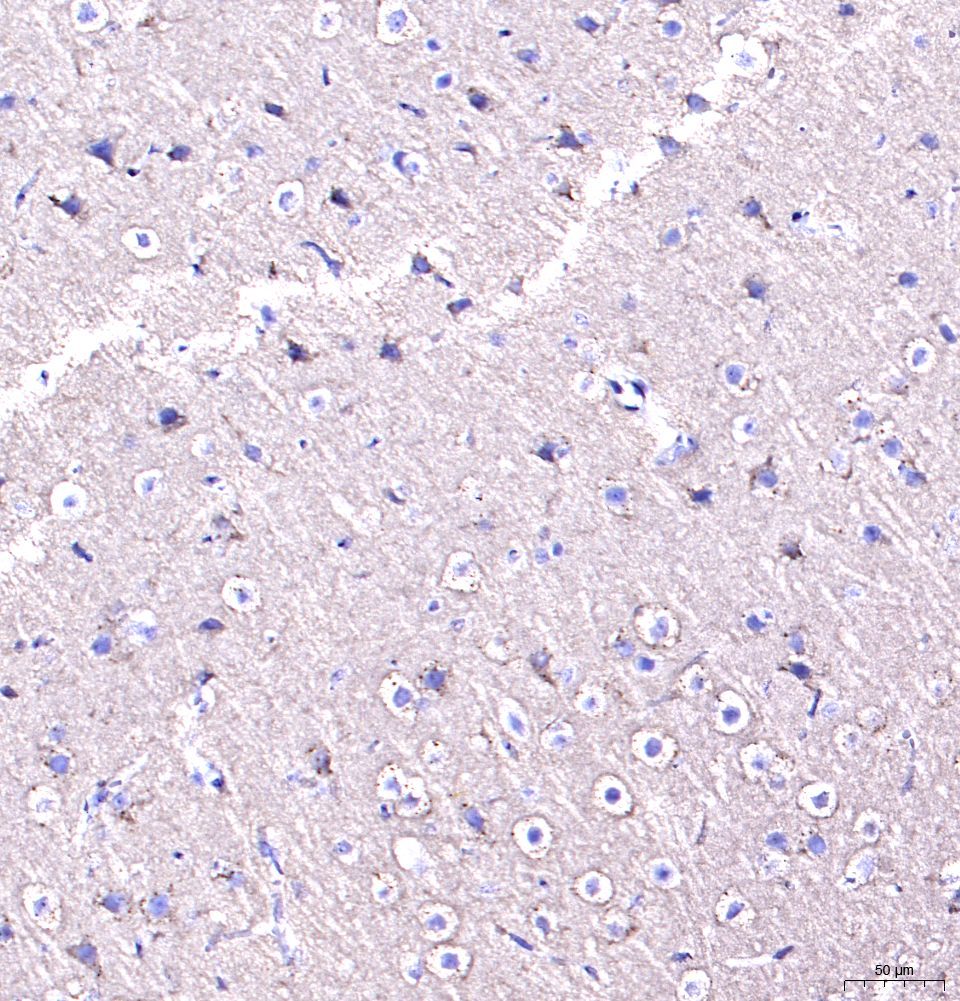

Supplement: Supplementary file 4 [file DataSheet11.ZIP › IHC Raw Image of PKA in PFC (1)/L7 1-200 PKA_20.0x-Q1.tif]

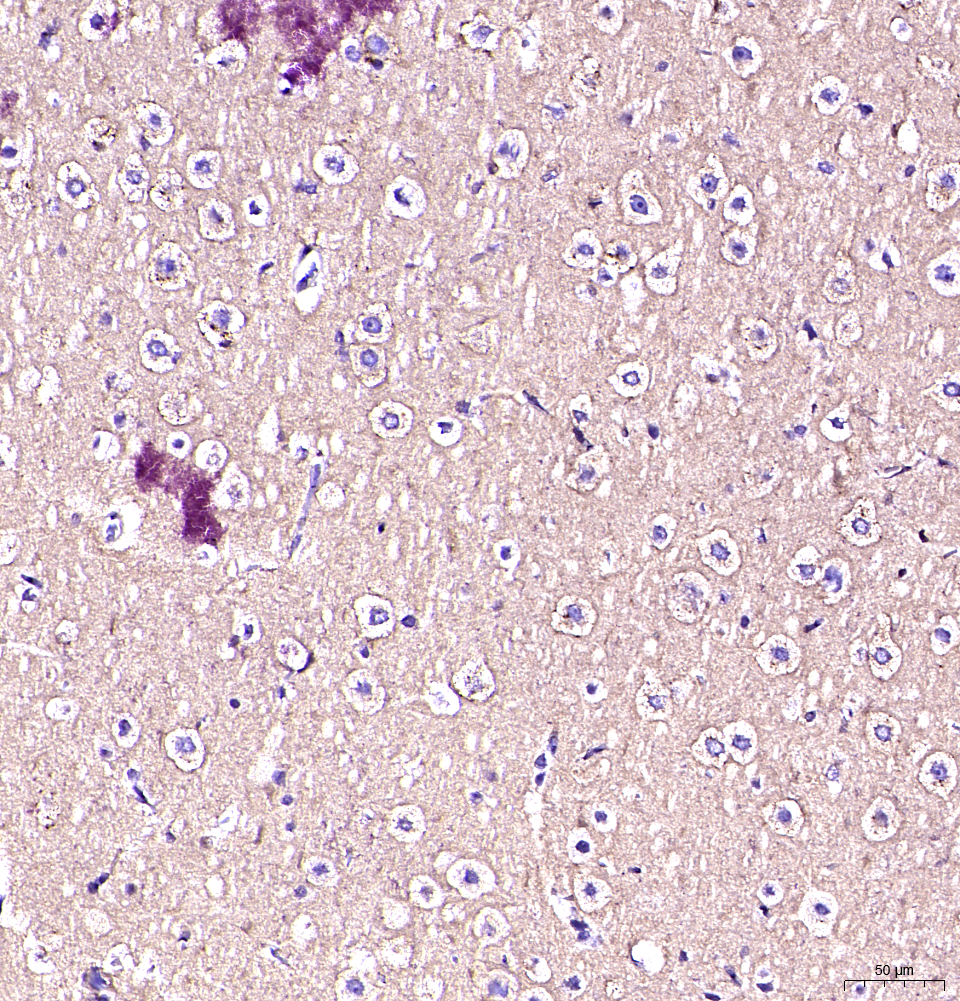

Supplement: Supplementary file 4 [file DataSheet11.ZIP › IHC Raw Image of PKA in PFC (1)/L9 1-200 PKA_20.0x-Q1.tif]

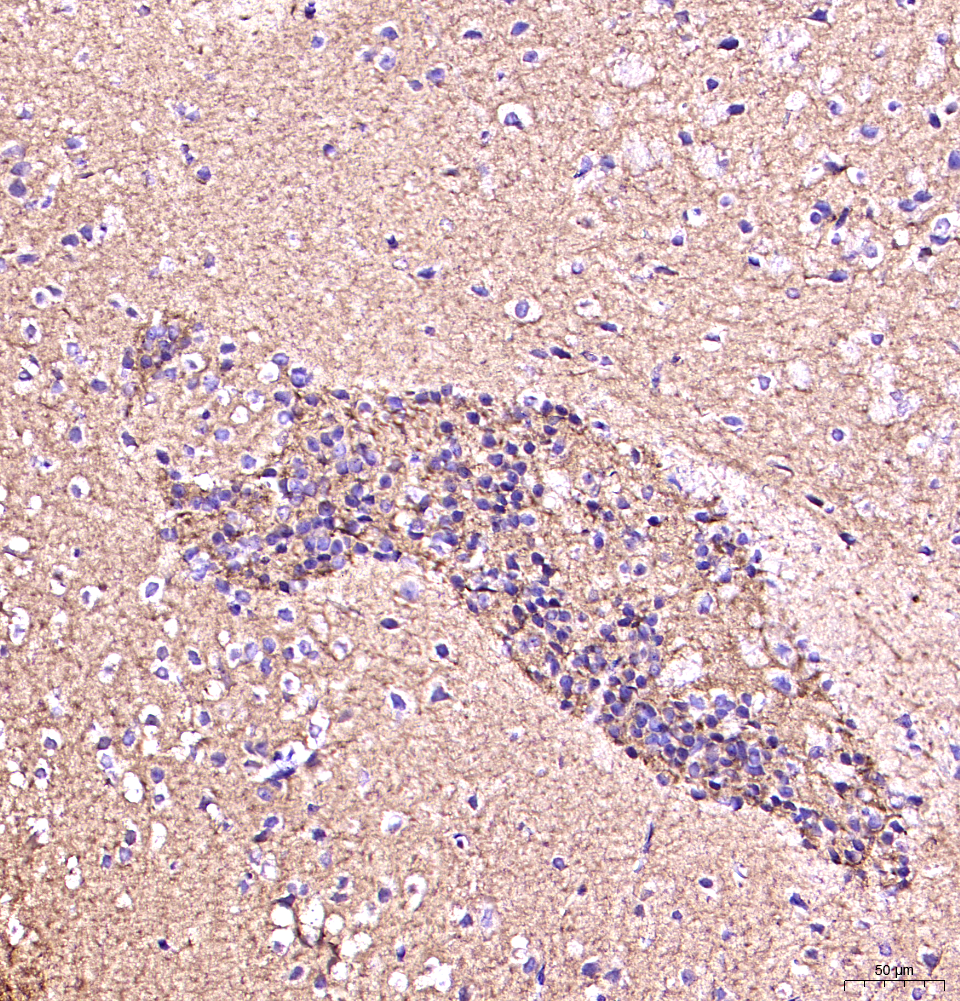

Supplement: Supplementary file 4 [file DataSheet11.ZIP › IHC Raw Image of PKA in PFC (1)/M11 1-200 PKA_20.0x-Q4.tif]

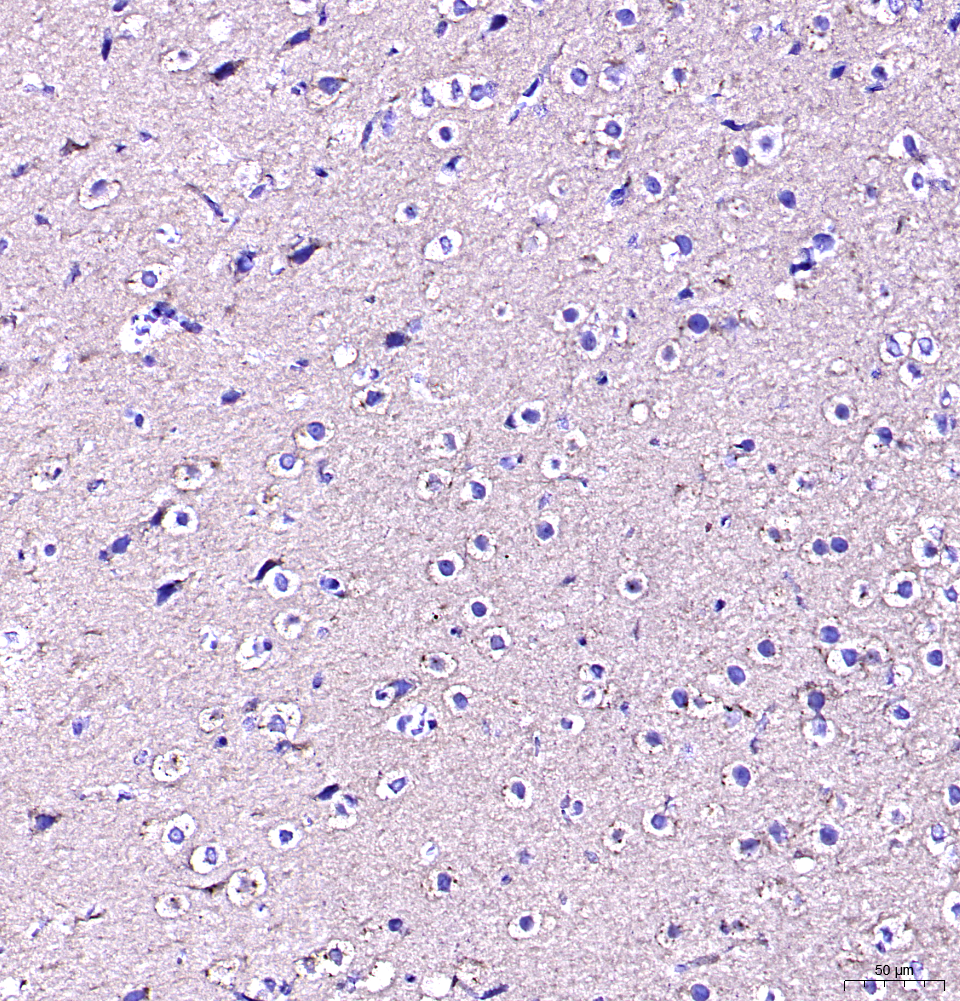

Supplement: Supplementary file 4 [file DataSheet11.ZIP › IHC Raw Image of PKA in PFC (1)/M27 1-200 PKA_20.0x-Q1.tif]

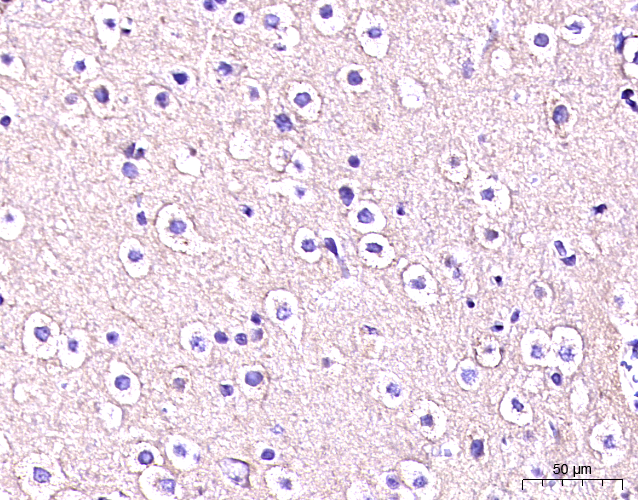

Supplement: Supplementary file 4 [file DataSheet11.ZIP › IHC Raw Image of PKA in PFC (1)/MX1 1-200 PKA_20.0x.tif]

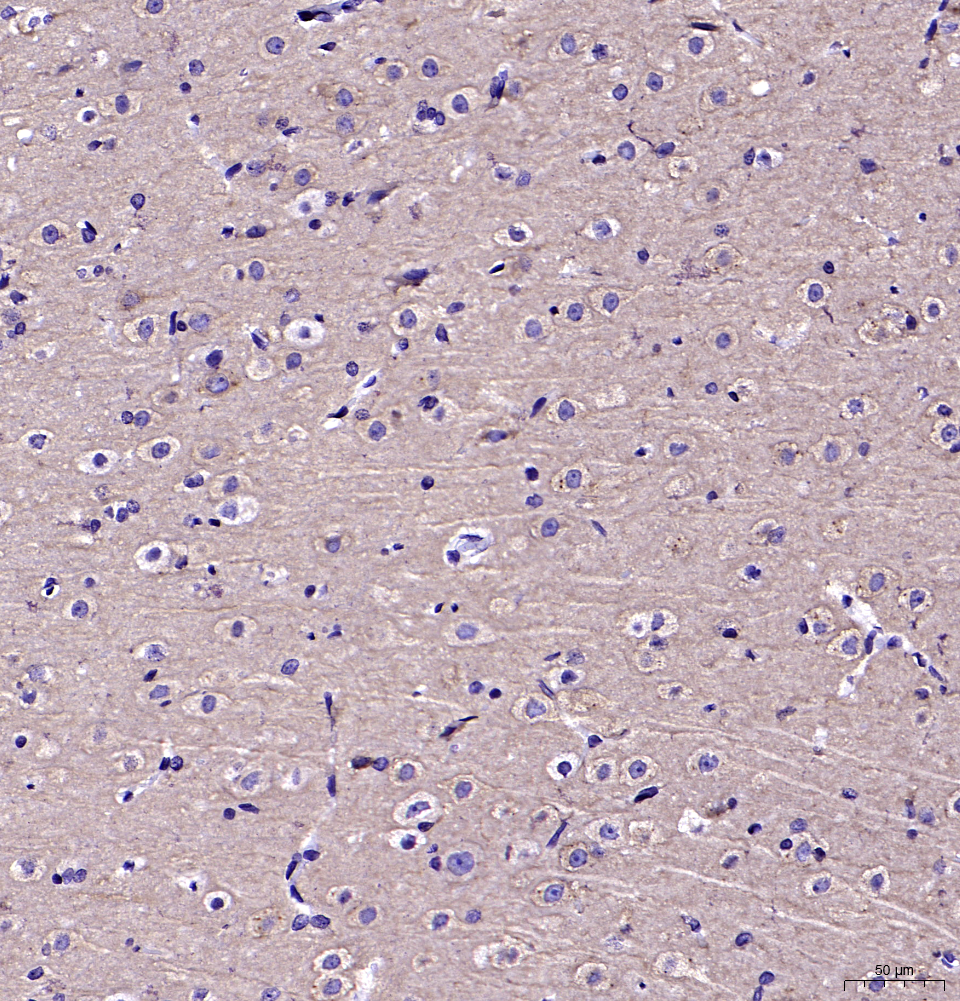

Supplement: Supplementary file 4 [file DataSheet11.ZIP › IHC Raw Image of PKA in PFC (1)/MX18 1-200 PKA_20.0x-Q1.tif]

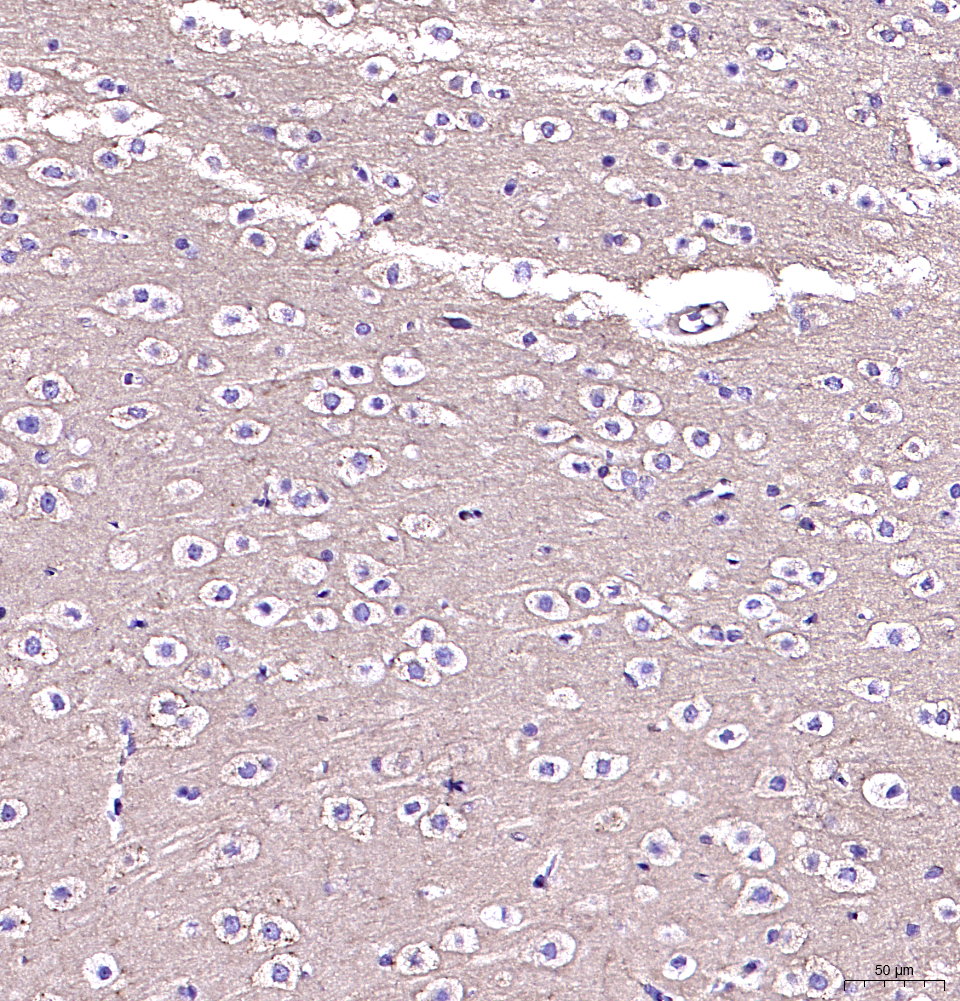

Supplement: Supplementary file 4 [file DataSheet11.ZIP › IHC Raw Image of PKA in PFC (1)/MX21 1-200 PKA_20.0x-Q1.tif]

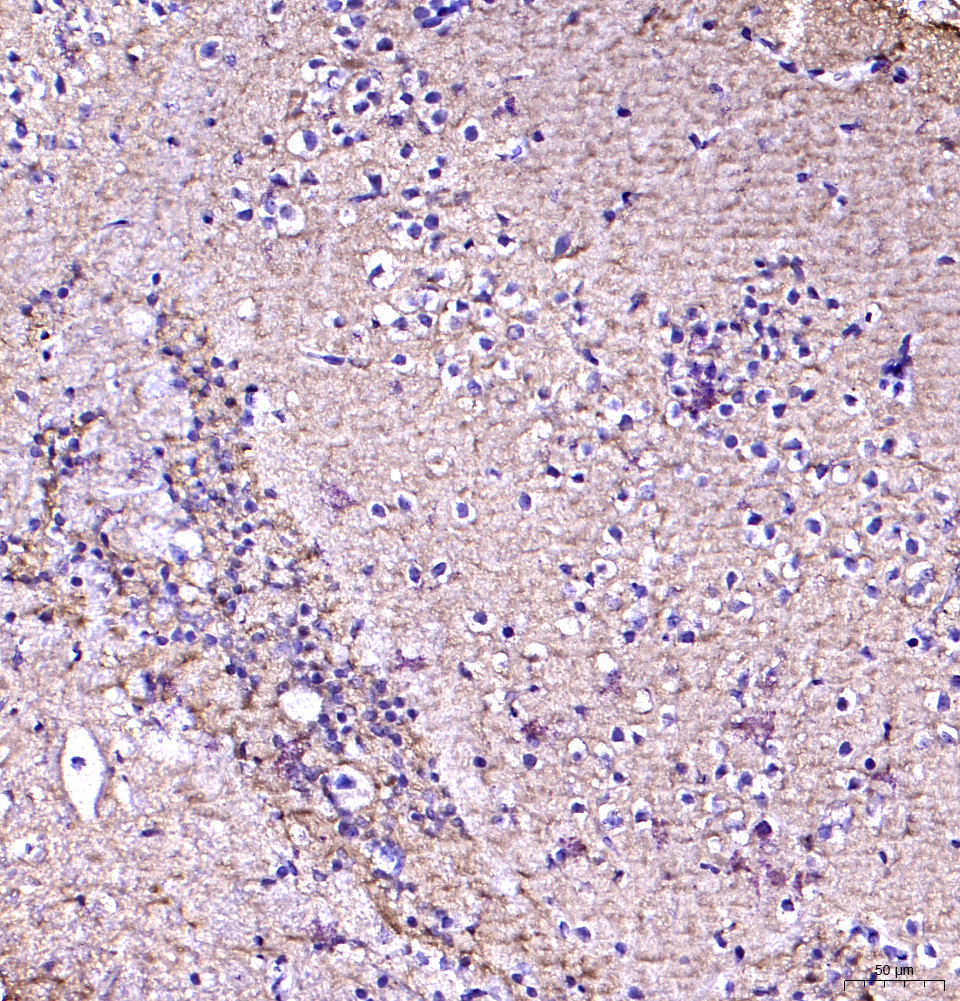

Supplement: Supplementary file 4 [file DataSheet11.ZIP › IHC Raw Image of PKA in PFC (1)/Z23 1-200 PKA_20.0x-Q3.tif]

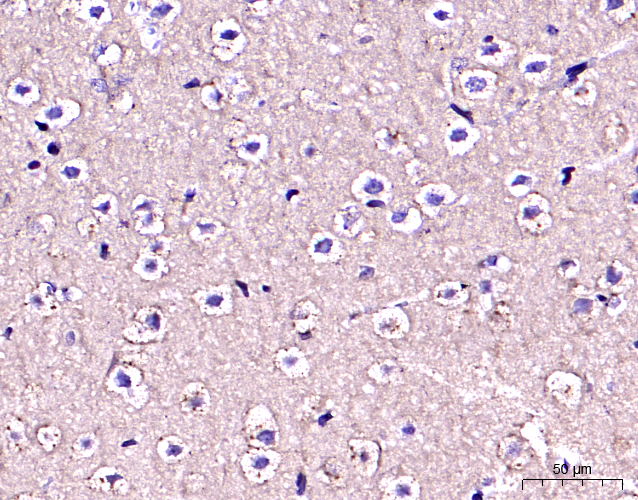

Supplement: Supplementary file 4 [file DataSheet11.ZIP › IHC Raw Image of PKA in PFC (1)/Z46 1-200 PKA_20.0x.tif]

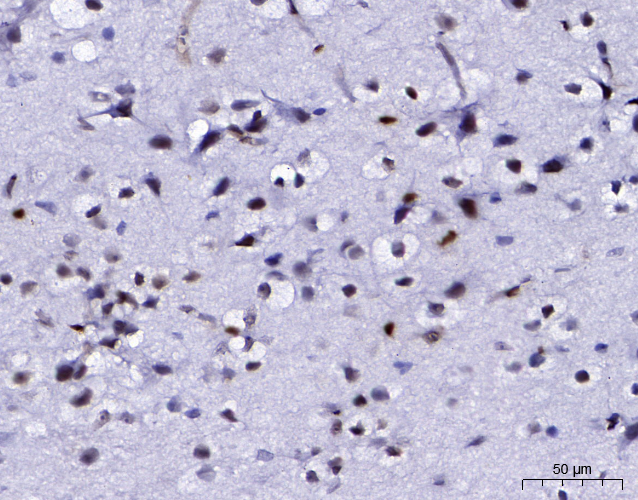

Supplement: Supplementary file 6 [file DataSheet8.ZIP › IHC Raw Image of p-CREB in striatum (1)/K61 1-200 PCREB_20.0x.tif-W1.tif]

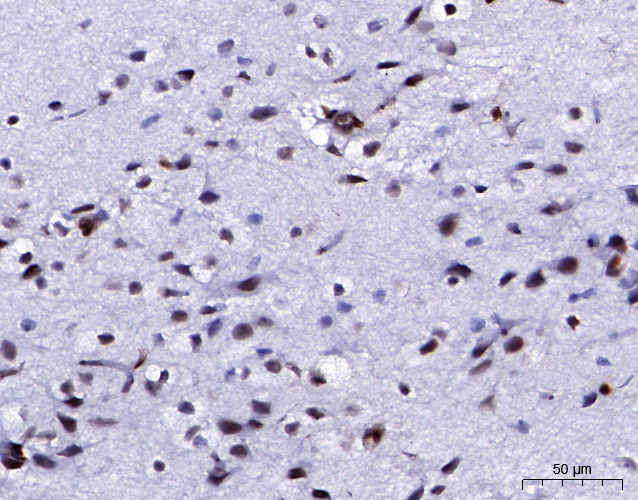

Supplement: Supplementary file 6 [file DataSheet8.ZIP › IHC Raw Image of p-CREB in striatum (1)/K61 1-200 PCREB_20.0x.tif-W2.tif]

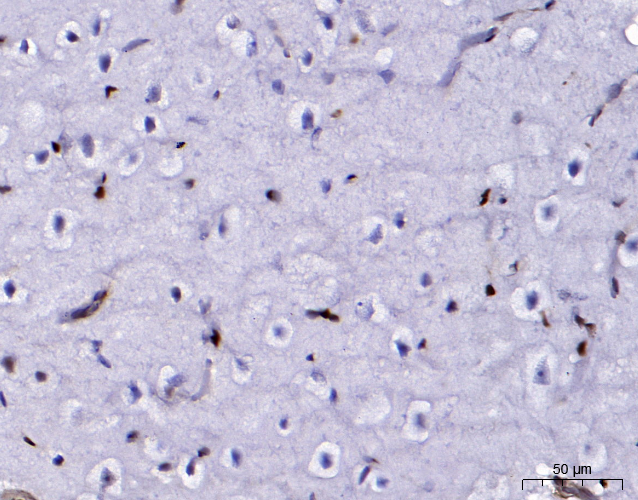

Supplement: Supplementary file 6 [file DataSheet8.ZIP › IHC Raw Image of p-CREB in striatum (1)/K61 1-200 PCREB_20.0x.tif-W3.tif]

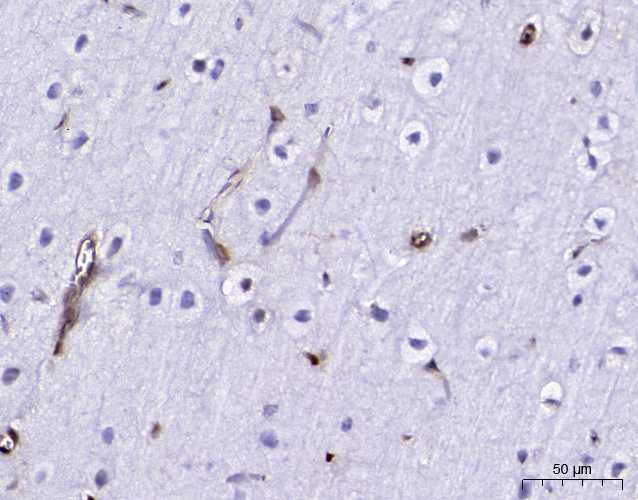

Supplement: Supplementary file 6 [file DataSheet8.ZIP › IHC Raw Image of p-CREB in striatum (1)/K61 1-200 PCREB_20.0x.tif-W4.tif]

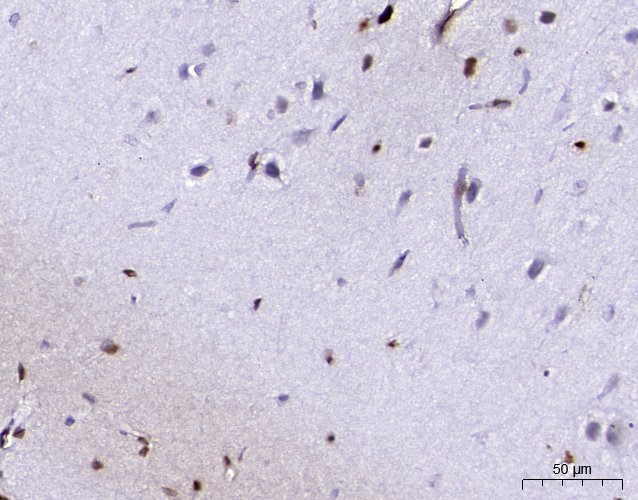

Supplement: Supplementary file 6 [file DataSheet8.ZIP › IHC Raw Image of p-CREB in striatum (1)/K61 1-200 PCREB_20.0x.tif-W5.tif]

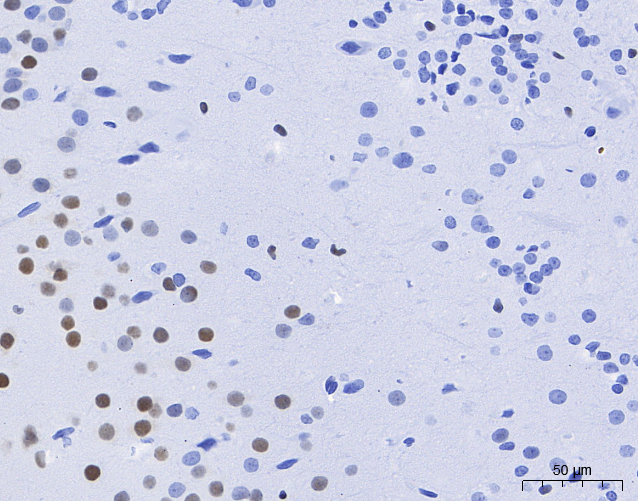

Supplement: Supplementary file 6 [file DataSheet8.ZIP › IHC Raw Image of p-CREB in striatum (1)/K65 CREB_20.0x.tif-W1.tif]

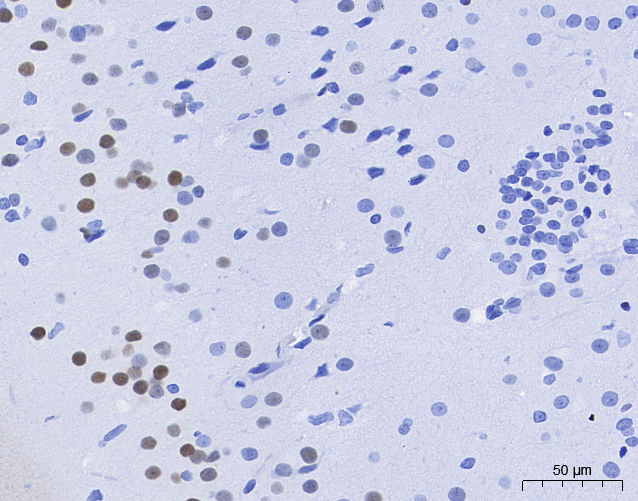

Supplement: Supplementary file 6 [file DataSheet8.ZIP › IHC Raw Image of p-CREB in striatum (1)/K65 CREB_20.0x.tif-W2.tif]

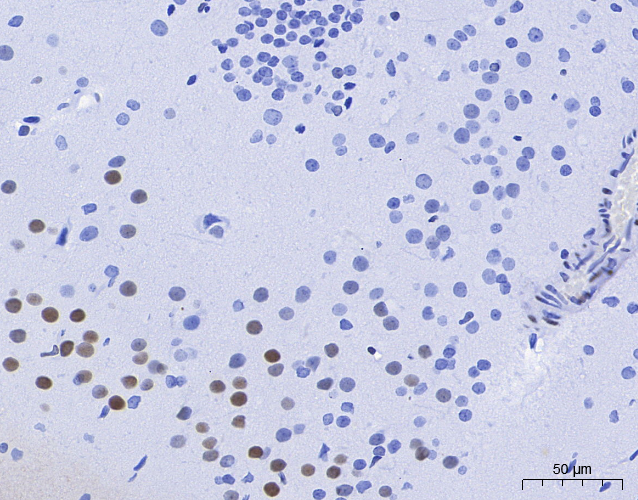

Supplement: Supplementary file 6 [file DataSheet8.ZIP › IHC Raw Image of p-CREB in striatum (1)/K65 CREB_20.0x.tif-W3.tif]

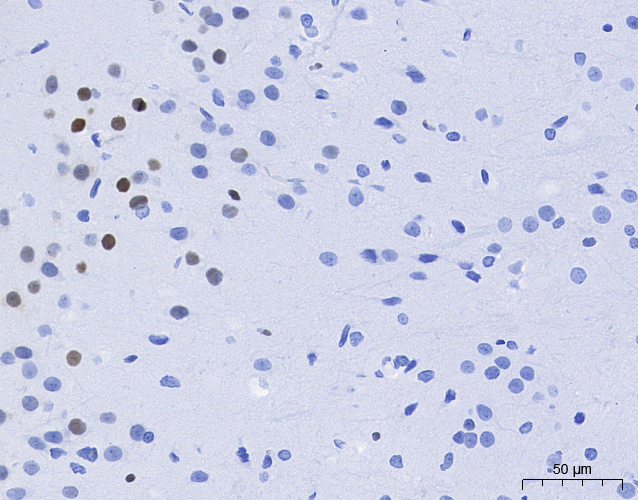

Supplement: Supplementary file 6 [file DataSheet8.ZIP › IHC Raw Image of p-CREB in striatum (1)/K65 CREB_20.0x.tif-W4.tif]

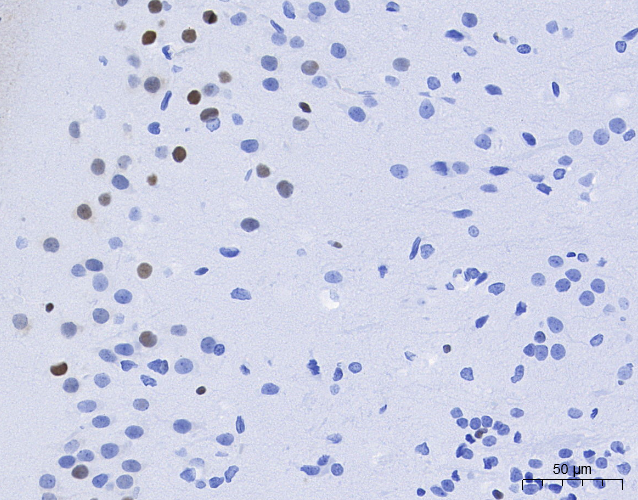

Supplement: Supplementary file 6 [file DataSheet8.ZIP › IHC Raw Image of p-CREB in striatum (1)/K65 CREB_20.0x.tif-W5.tif]

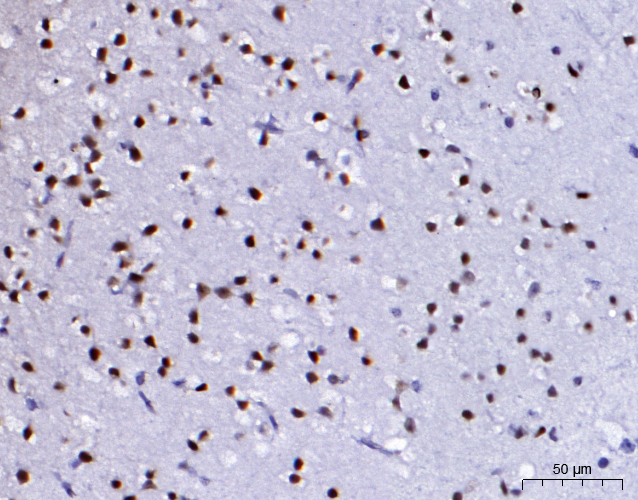

Supplement: Supplementary file 6 [file DataSheet8.ZIP › IHC Raw Image of p-CREB in striatum (1)/K67 1-200 PCREB_20.0x.tif-W1.tif]

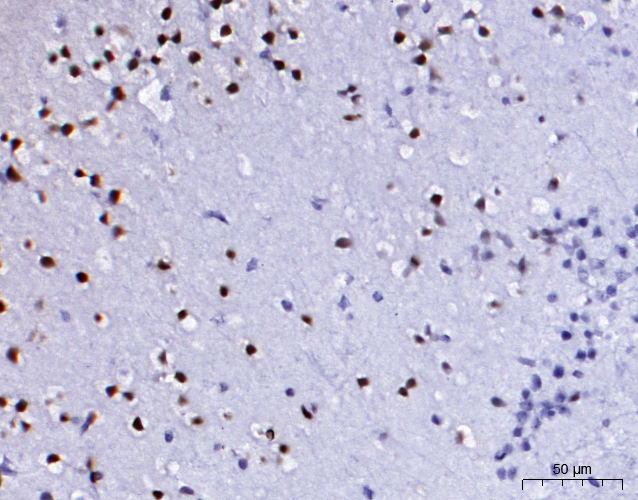

Supplement: Supplementary file 6 [file DataSheet8.ZIP › IHC Raw Image of p-CREB in striatum (1)/K67 1-200 PCREB_20.0x.tif-W2.tif]

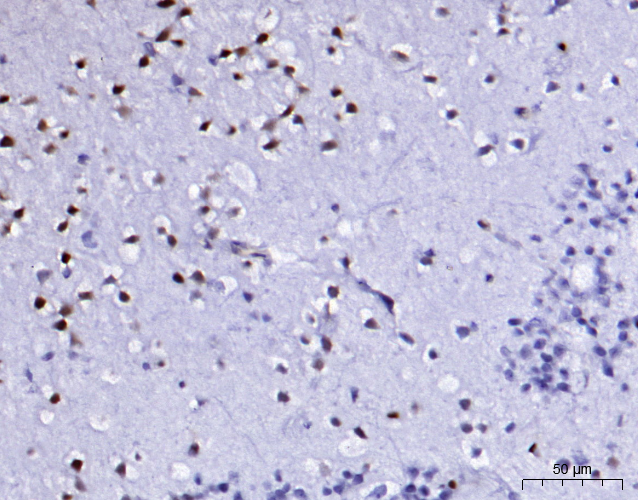

Supplement: Supplementary file 6 [file DataSheet8.ZIP › IHC Raw Image of p-CREB in striatum (1)/K67 1-200 PCREB_20.0x.tif-W3.tif]

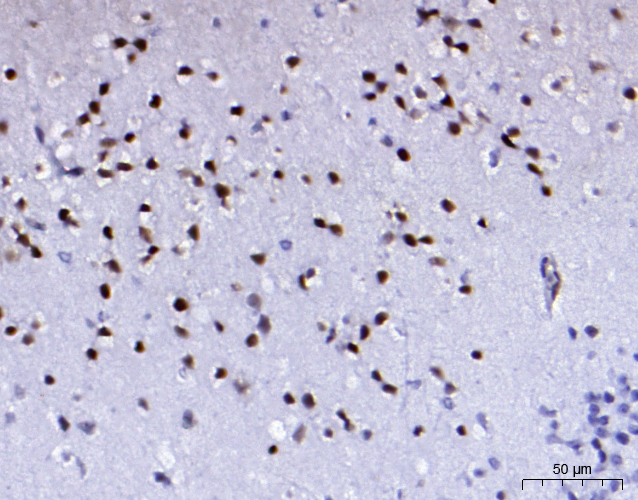

Supplement: Supplementary file 6 [file DataSheet8.ZIP › IHC Raw Image of p-CREB in striatum (1)/K67 1-200 PCREB_20.0x.tif-W4.tif]

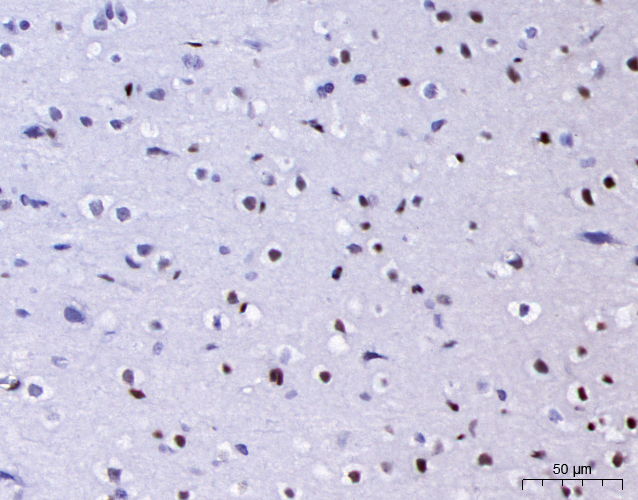

Supplement: Supplementary file 6 [file DataSheet8.ZIP › IHC Raw Image of p-CREB in striatum (1)/K67 1-200 PCREB_20.0x.tif-W5.tif]

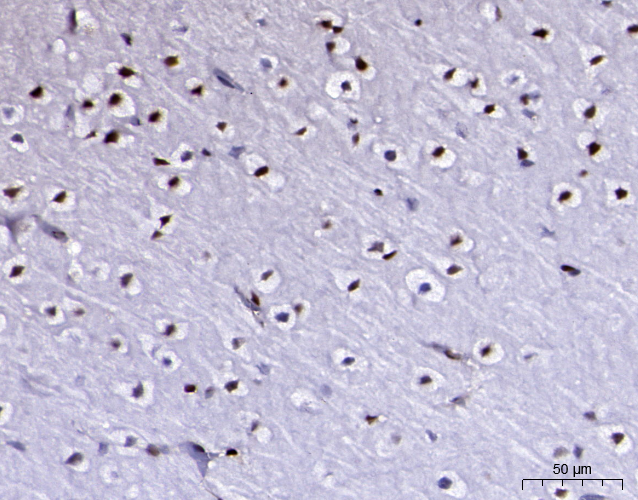

Supplement: Supplementary file 6 [file DataSheet8.ZIP › IHC Raw Image of p-CREB in striatum (1)/K69 1-200 PCREB_20.0x.tif-W1.tif]

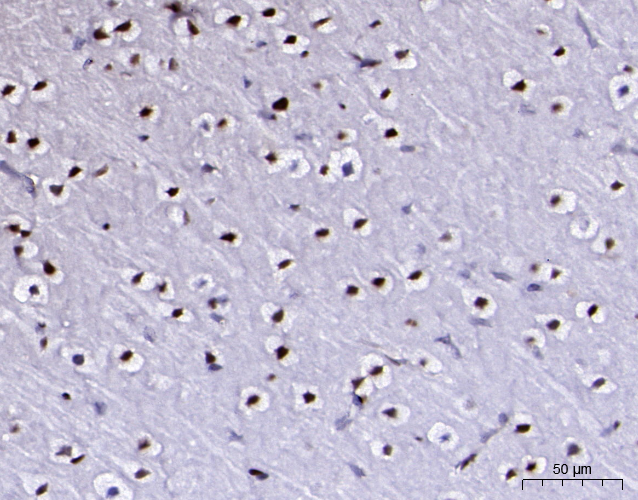

Supplement: Supplementary file 6 [file DataSheet8.ZIP › IHC Raw Image of p-CREB in striatum (1)/K69 1-200 PCREB_20.0x.tif-W2.tif]

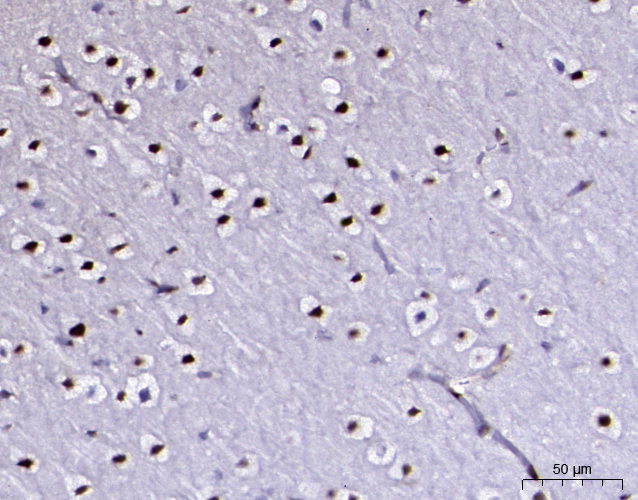

Supplement: Supplementary file 6 [file DataSheet8.ZIP › IHC Raw Image of p-CREB in striatum (1)/K69 1-200 PCREB_20.0x.tif-W3.tif]

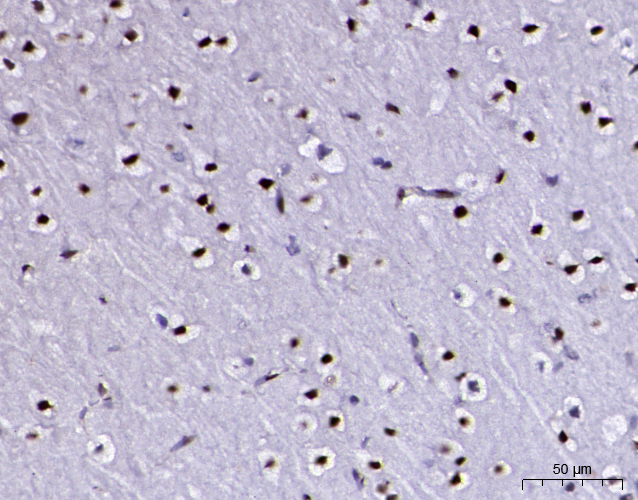

Supplement: Supplementary file 6 [file DataSheet8.ZIP › IHC Raw Image of p-CREB in striatum (1)/K69 1-200 PCREB_20.0x.tif-W4.tif]

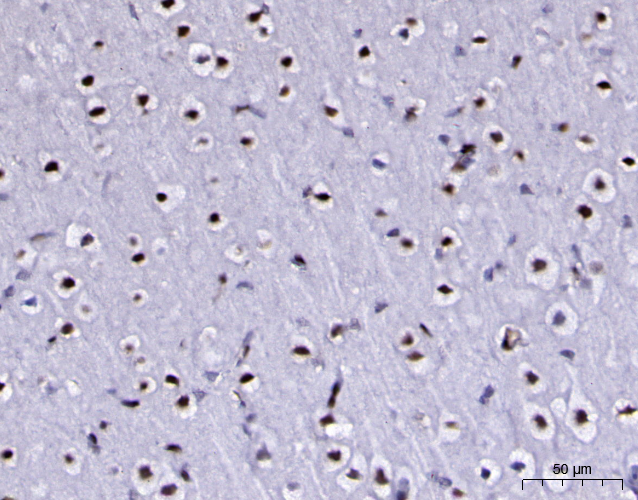

Supplement: Supplementary file 6 [file DataSheet8.ZIP › IHC Raw Image of p-CREB in striatum (1)/K69 1-200 PCREB_20.0x.tif-W5.tif]

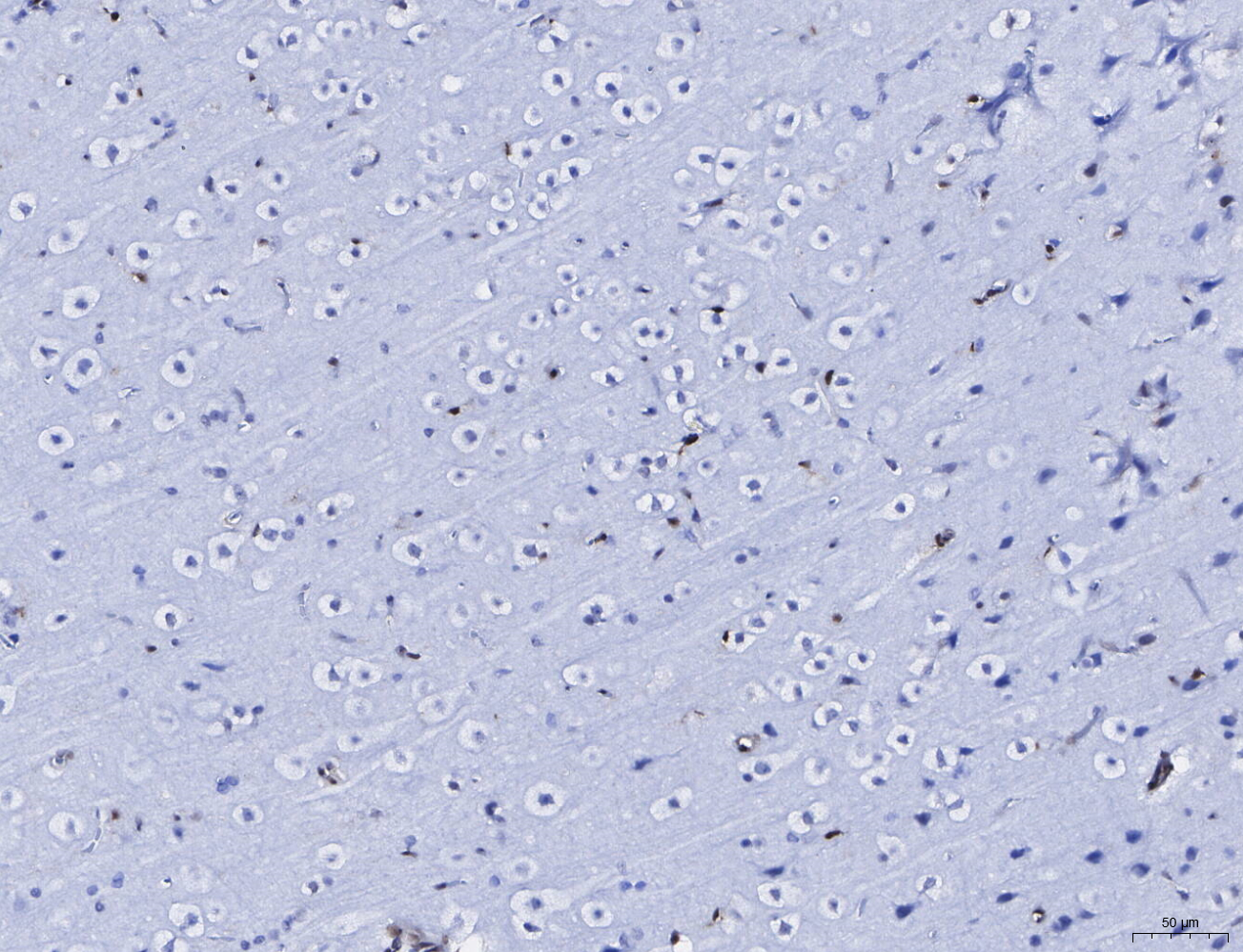

Supplement: Supplementary file 6 [file DataSheet8.ZIP › IHC Raw Image of p-CREB in striatum (1)/L7 pCREB.svs_20.0x-10.tif-W5.tif]

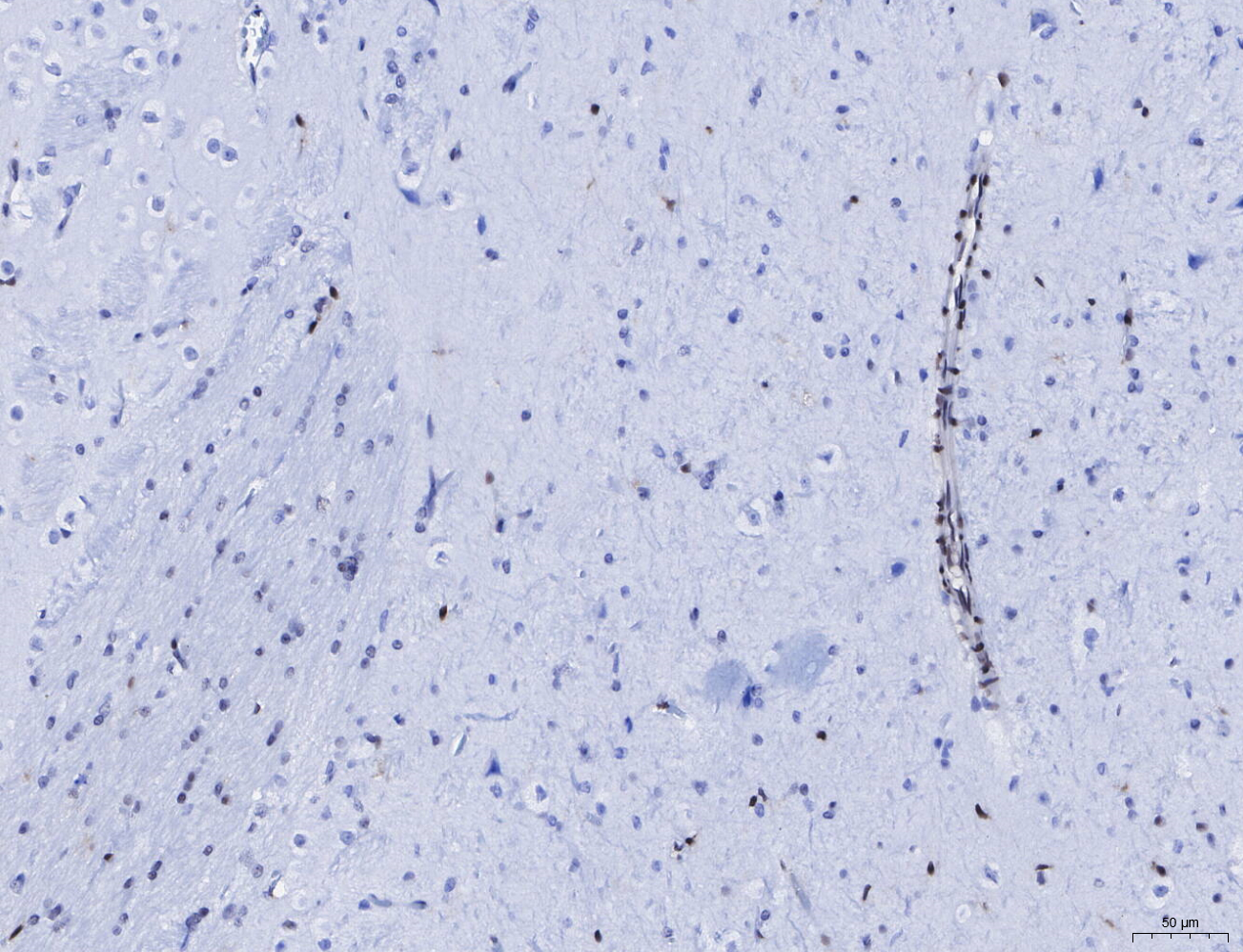

Supplement: Supplementary file 6 [file DataSheet8.ZIP › IHC Raw Image of p-CREB in striatum (1)/L7 pCREB.svs_20.0x-4.tif-W1.tif]

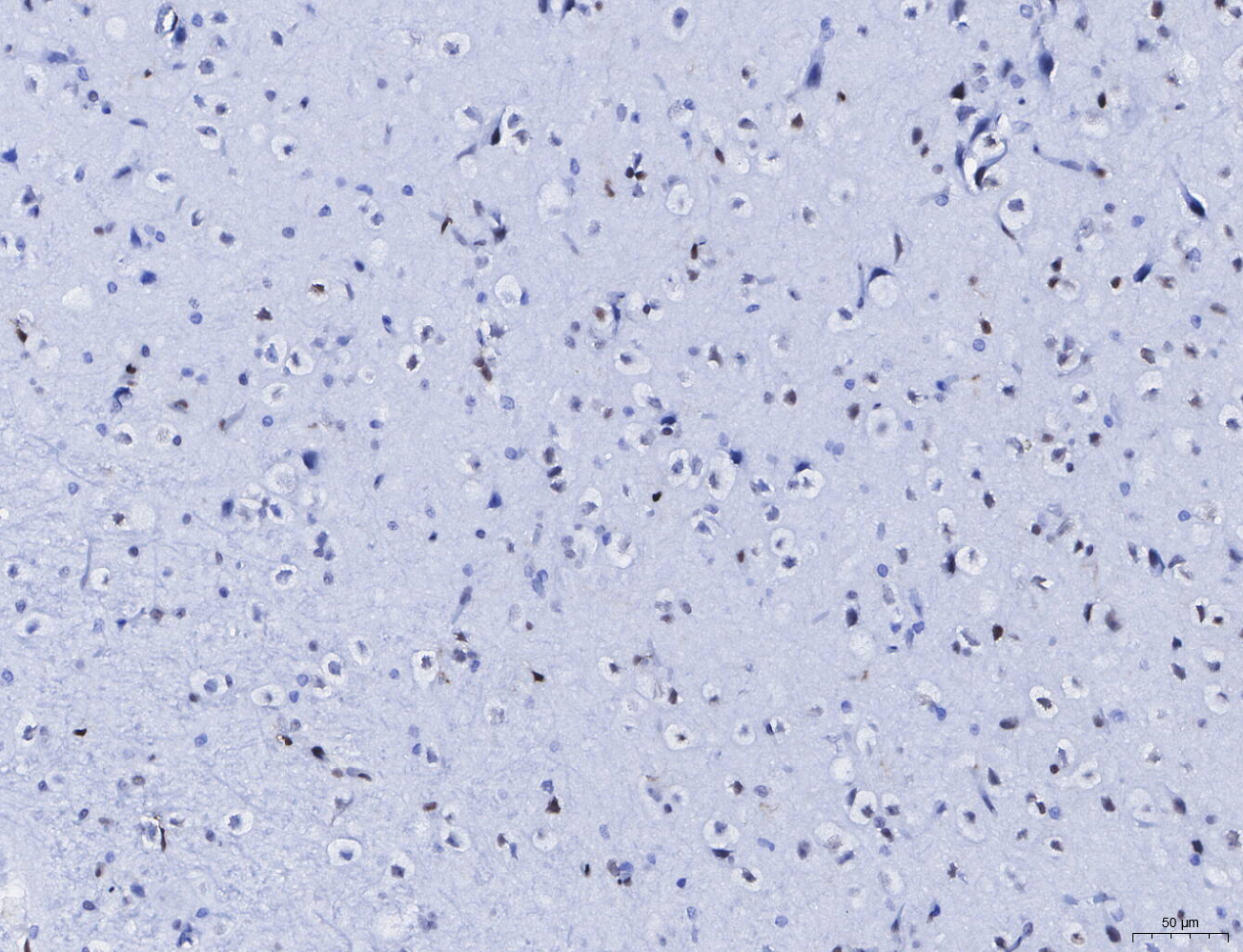

Supplement: Supplementary file 6 [file DataSheet8.ZIP › IHC Raw Image of p-CREB in striatum (1)/L7 pCREB.svs_20.0x-6.tif-W2.tif]

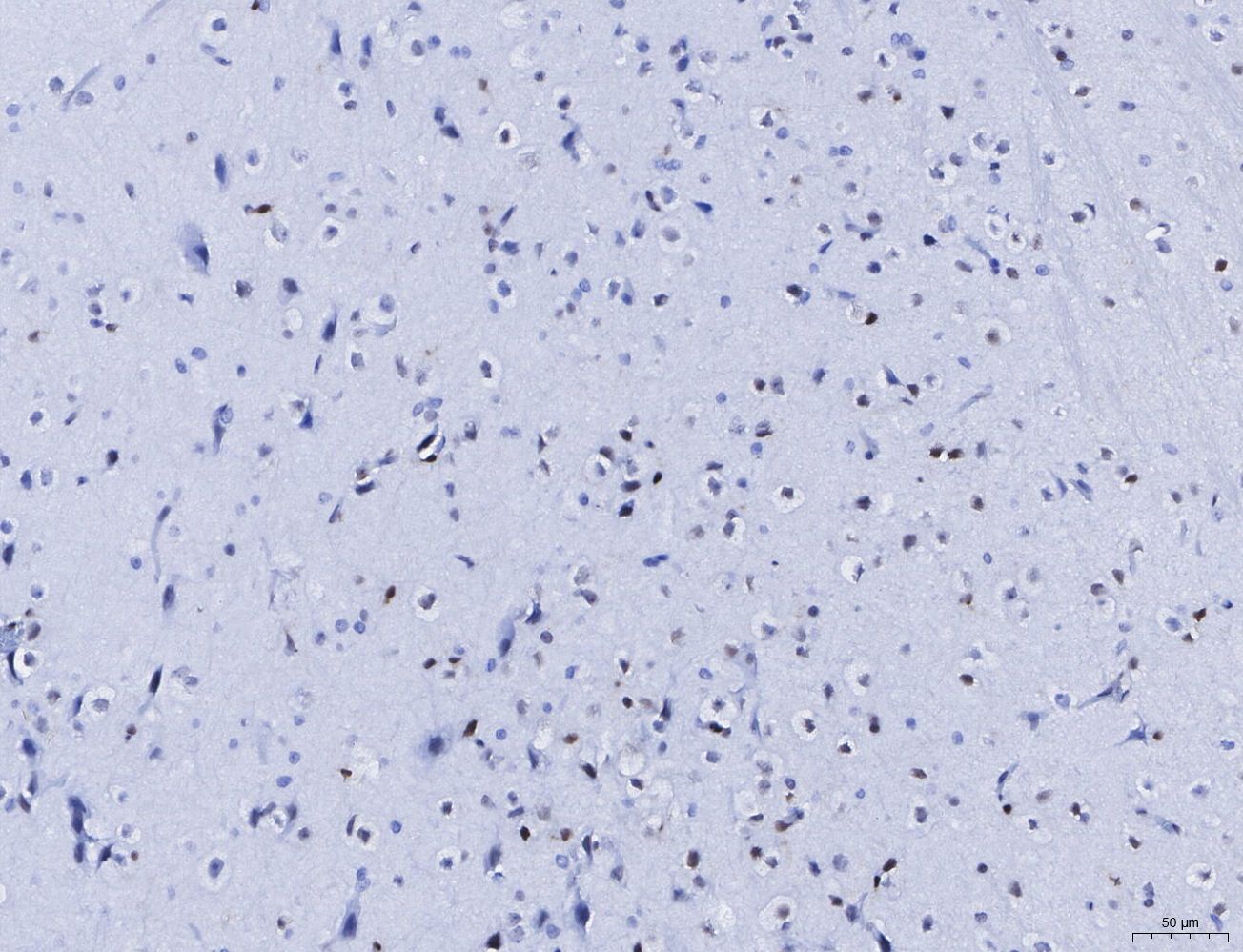

Supplement: Supplementary file 6 [file DataSheet8.ZIP › IHC Raw Image of p-CREB in striatum (1)/L7 pCREB.svs_20.0x-7.tif-W3.tif]

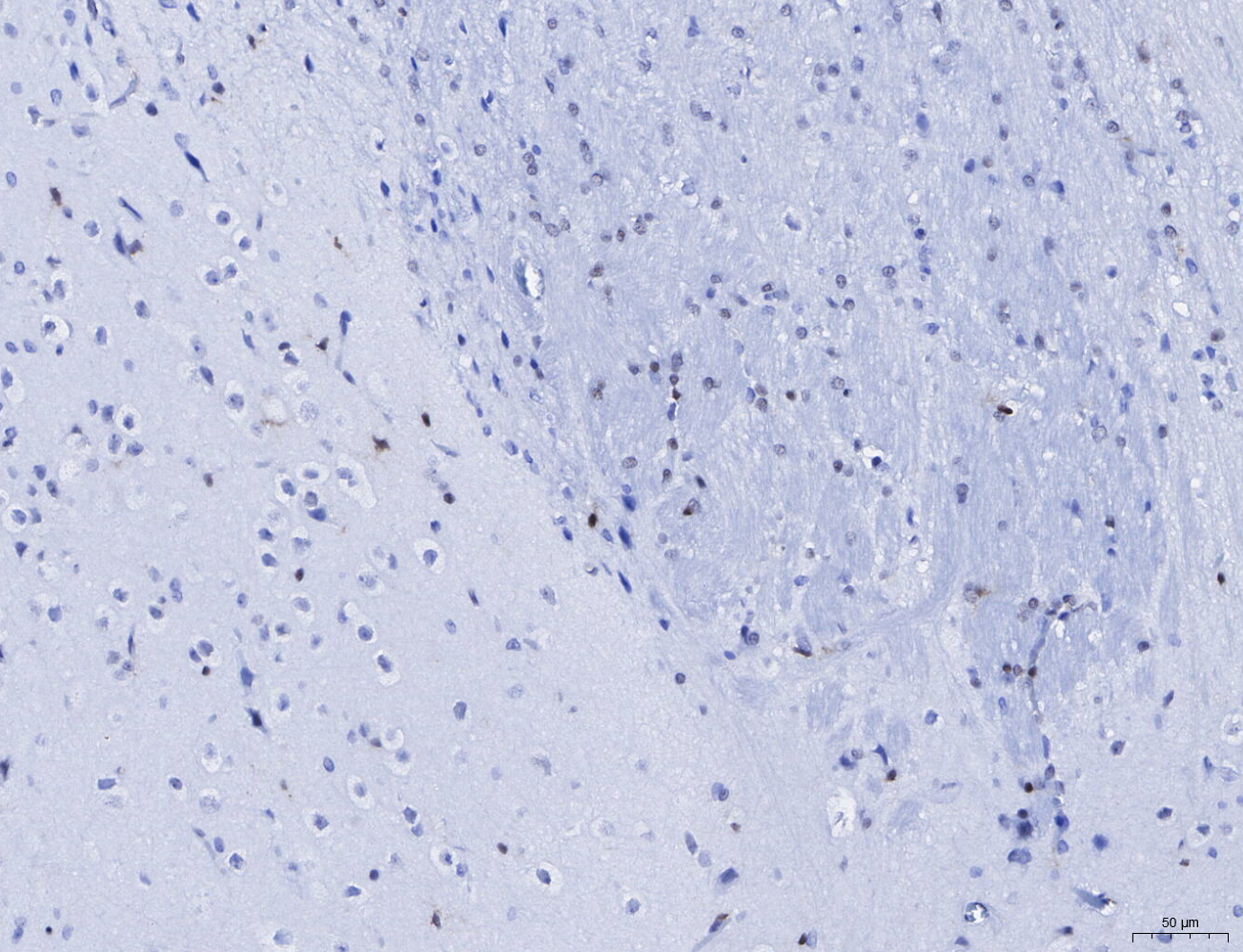

Supplement: Supplementary file 6 [file DataSheet8.ZIP › IHC Raw Image of p-CREB in striatum (1)/L7 pCREB.svs_20.0x-8.tif-W4.tif]

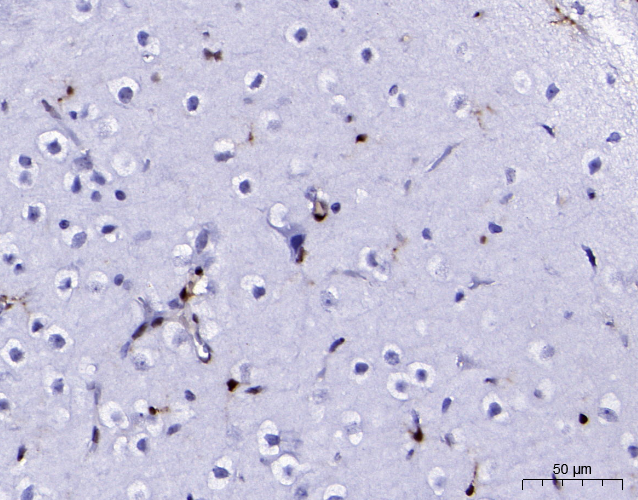

Supplement: Supplementary file 6 [file DataSheet8.ZIP › IHC Raw Image of p-CREB in striatum (1)/L9 1-200 PCREB_20.0x.tif-W1.tif]

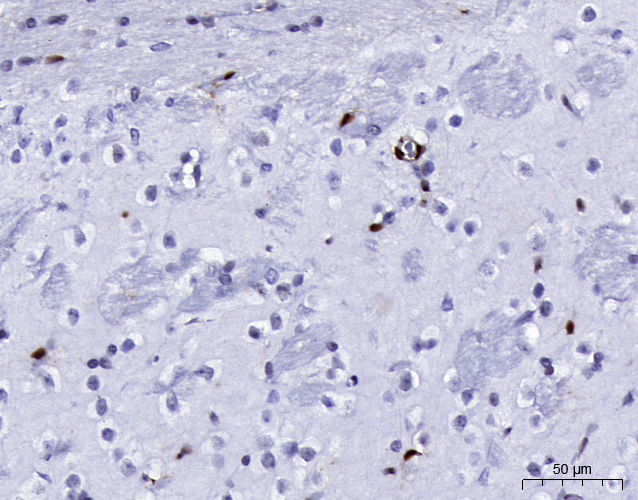

Supplement: Supplementary file 6 [file DataSheet8.ZIP › IHC Raw Image of p-CREB in striatum (1)/L9 1-200 PCREB_20.0x.tif-W2.tif]

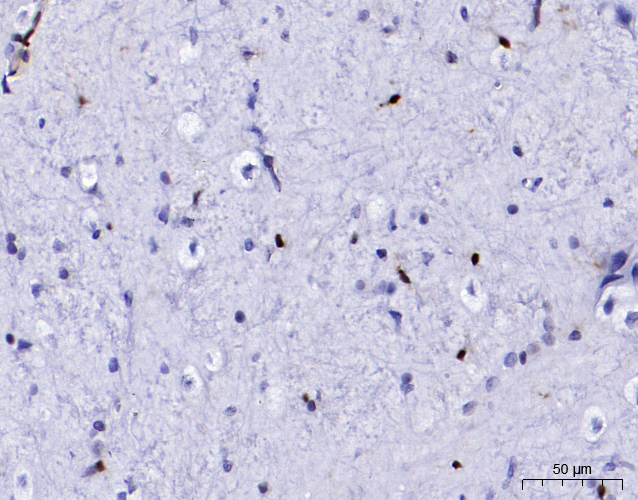

Supplement: Supplementary file 6 [file DataSheet8.ZIP › IHC Raw Image of p-CREB in striatum (1)/L9 1-200 PCREB_20.0x.tif-W3.tif]

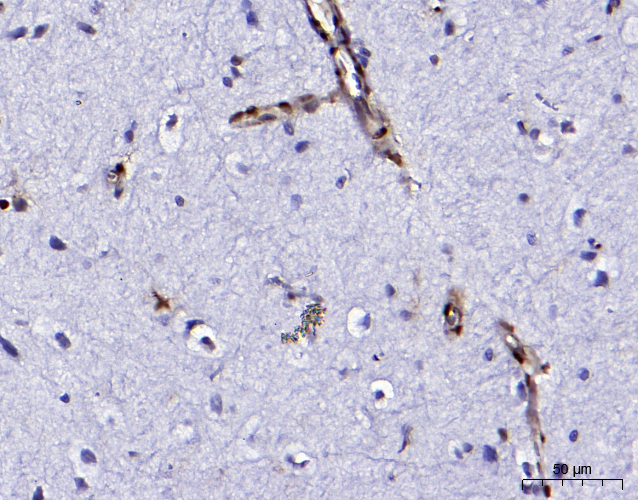

Supplement: Supplementary file 6 [file DataSheet8.ZIP › IHC Raw Image of p-CREB in striatum (1)/L9 1-200 PCREB_20.0x.tif-W4.tif]

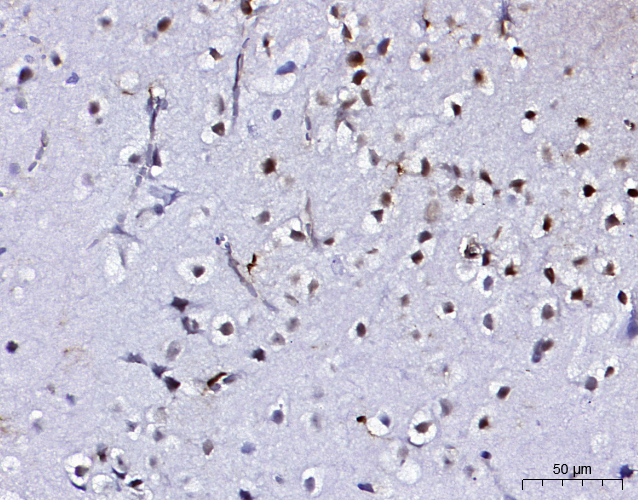

Supplement: Supplementary file 6 [file DataSheet8.ZIP › IHC Raw Image of p-CREB in striatum (1)/L9 1-200 PCREB_20.0x.tif-W5.tif]

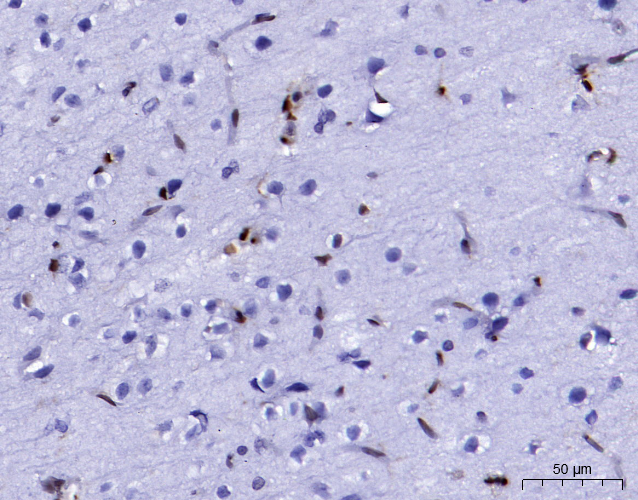

Supplement: Supplementary file 7 [file DataSheet9.ZIP › IHC Raw Image of p-CREB in striatum (2)/MX1 1-200 PCREB_20.0x.tif-W1.tif]

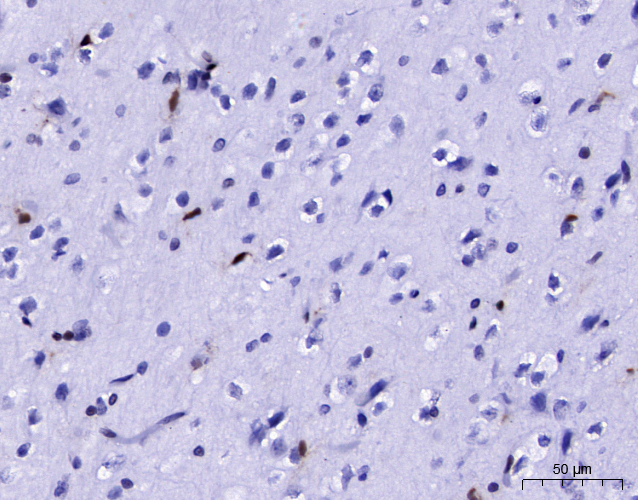

Supplement: Supplementary file 7 [file DataSheet9.ZIP › IHC Raw Image of p-CREB in striatum (2)/MX1 1-200 PCREB_20.0x.tif-W2.tif]

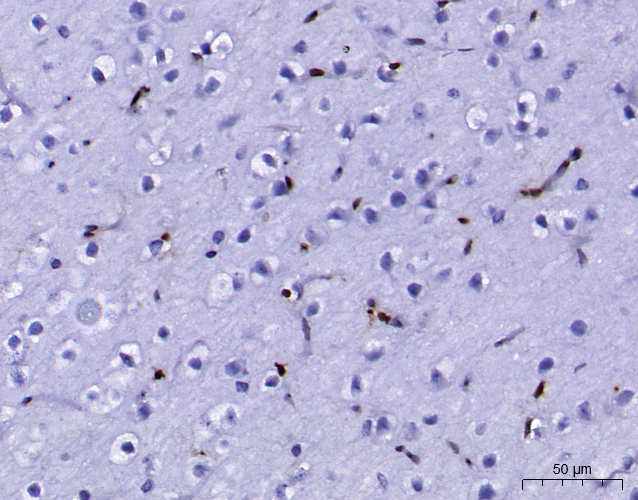

Supplement: Supplementary file 7 [file DataSheet9.ZIP › IHC Raw Image of p-CREB in striatum (2)/MX1 1-200 PCREB_20.0x.tif-W3.tif]

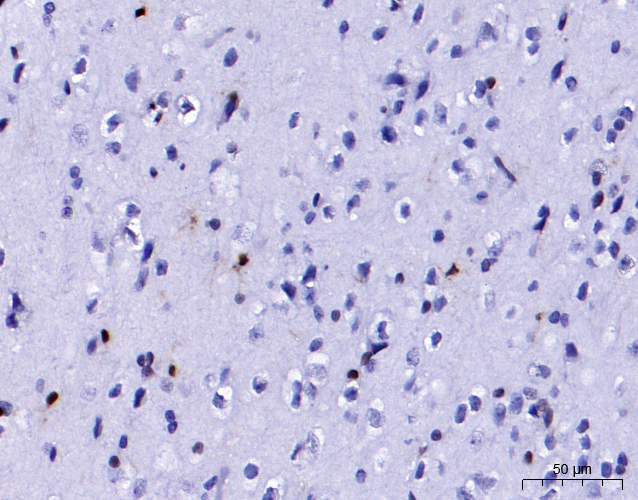

Supplement: Supplementary file 7 [file DataSheet9.ZIP › IHC Raw Image of p-CREB in striatum (2)/MX1 1-200 PCREB_20.0x.tif-W4.tif]
