# Supplementary figures and images for: Long Mu Qing Xin mixture improves behavioral performance in spontaneously hypertensive rats (SHR/NCrl) by upregulating catecholamine neurotransmitters in prefrontal cortex and striatum via DRD1/cAMP/PKA-CREB signaling pathway (part 2 of 4)
Source: Front Pharmacol. 2024 Jul 4;15:1387359. doi: 10.3389/fphar.2024.1387359 (PMC11254830; doi:10.3389/fphar.2024.1387359)

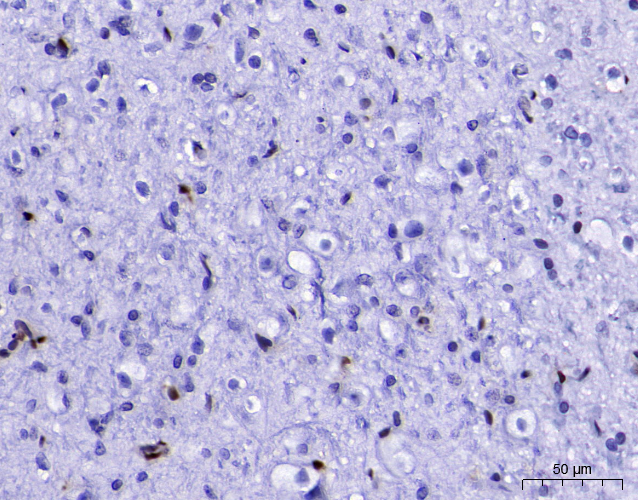

Supplement: Supplementary file 7 [file DataSheet9.ZIP › IHC Raw Image of p-CREB in striatum (2)/MX1 1-200 PCREB_20.0x.tif-W5.tif]

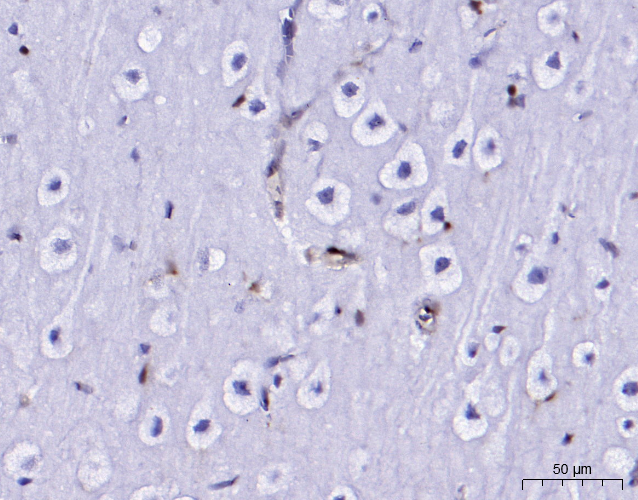

Supplement: Supplementary file 7 [file DataSheet9.ZIP › IHC Raw Image of p-CREB in striatum (2)/MX18 1-200 PCREB_20.0x.tif-W1.tif]

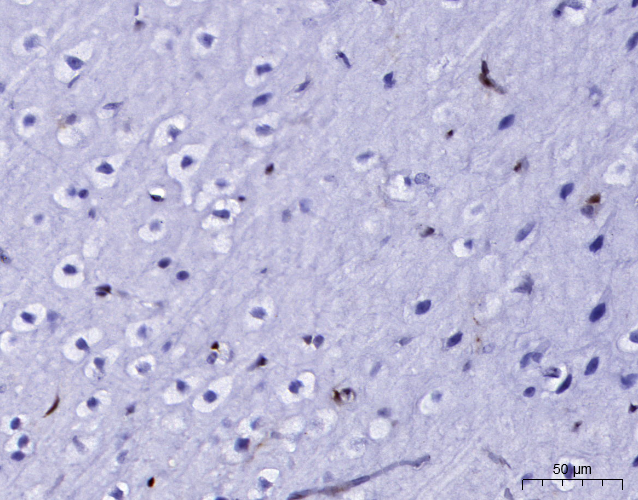

Supplement: Supplementary file 7 [file DataSheet9.ZIP › IHC Raw Image of p-CREB in striatum (2)/MX18 1-200 PCREB_20.0x.tif-W2.tif]

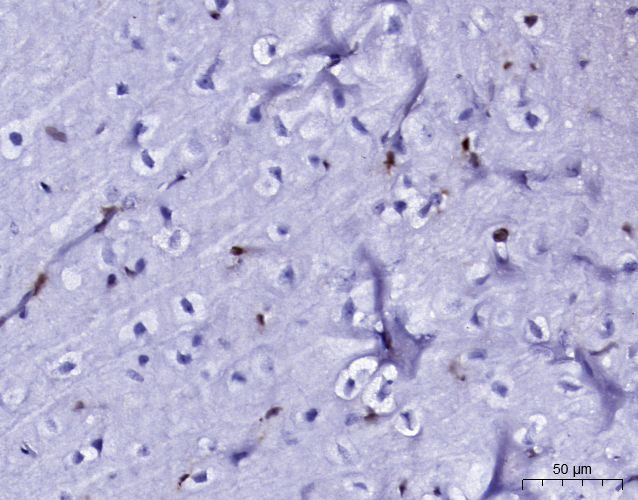

Supplement: Supplementary file 7 [file DataSheet9.ZIP › IHC Raw Image of p-CREB in striatum (2)/MX18 1-200 PCREB_20.0x.tif-W3.tif]

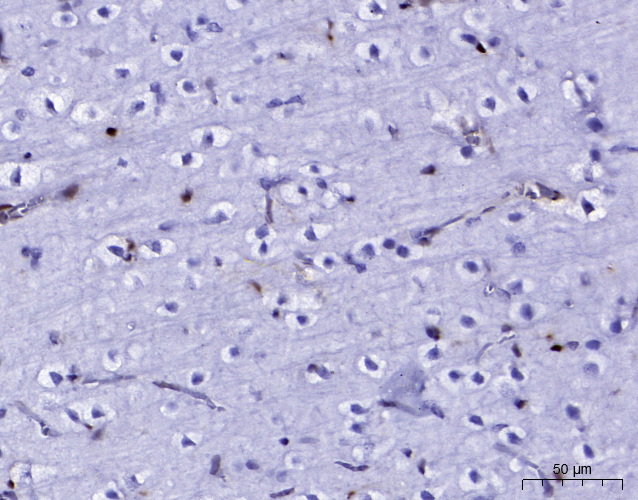

Supplement: Supplementary file 7 [file DataSheet9.ZIP › IHC Raw Image of p-CREB in striatum (2)/MX18 1-200 PCREB_20.0x.tif-W4.tif]

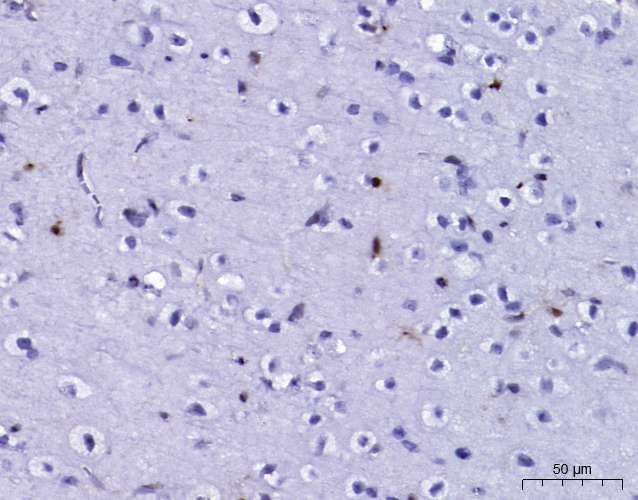

Supplement: Supplementary file 7 [file DataSheet9.ZIP › IHC Raw Image of p-CREB in striatum (2)/MX18 1-200 PCREB_20.0x.tif-W5.tif]

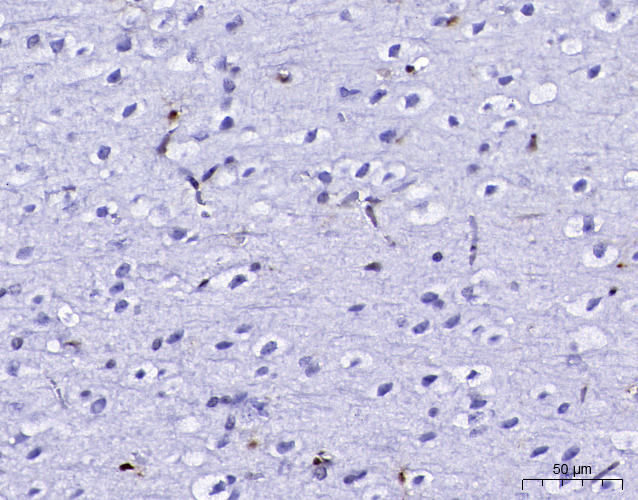

Supplement: Supplementary file 7 [file DataSheet9.ZIP › IHC Raw Image of p-CREB in striatum (2)/MX21 1-200 PCREB_20.0x.tif-W1.tif]

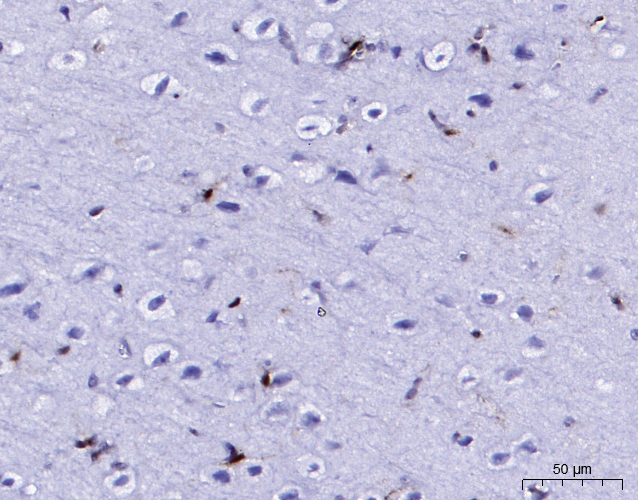

Supplement: Supplementary file 7 [file DataSheet9.ZIP › IHC Raw Image of p-CREB in striatum (2)/MX21 1-200 PCREB_20.0x.tif-W2.tif]

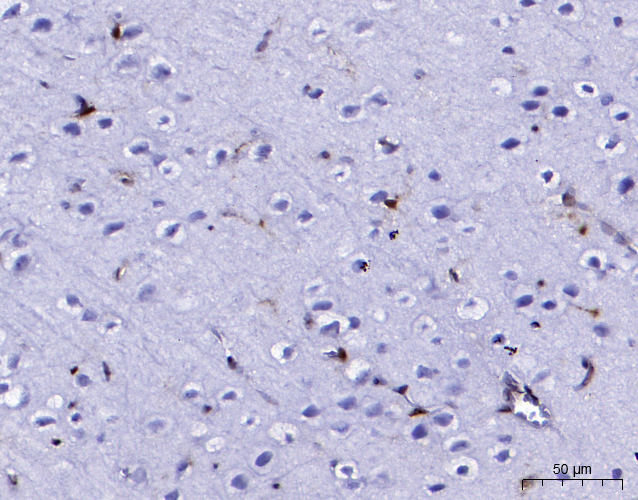

Supplement: Supplementary file 7 [file DataSheet9.ZIP › IHC Raw Image of p-CREB in striatum (2)/MX21 1-200 PCREB_20.0x.tif-W3.tif]

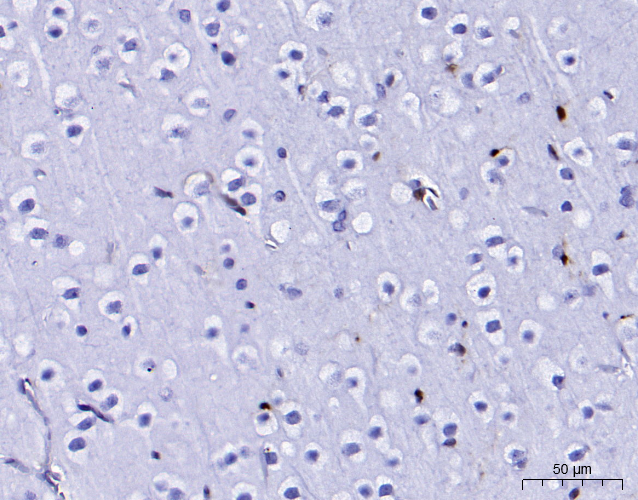

Supplement: Supplementary file 7 [file DataSheet9.ZIP › IHC Raw Image of p-CREB in striatum (2)/MX21 1-200 PCREB_20.0x.tif-W4.tif]

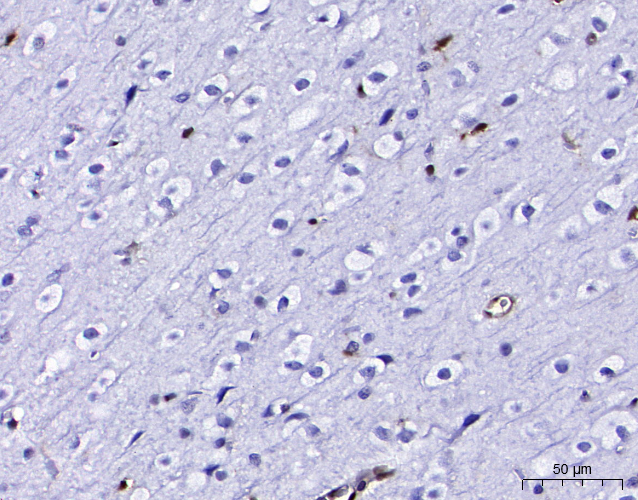

Supplement: Supplementary file 7 [file DataSheet9.ZIP › IHC Raw Image of p-CREB in striatum (2)/MX21 1-200 PCREB_20.0x.tif-W5.tif]

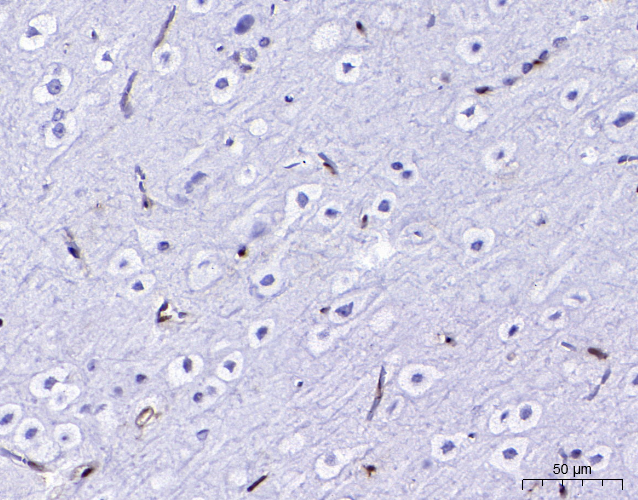

Supplement: Supplementary file 7 [file DataSheet9.ZIP › IHC Raw Image of p-CREB in striatum (2)/MX5 1-200 PCREB_20.0x.tif-W1.tif]

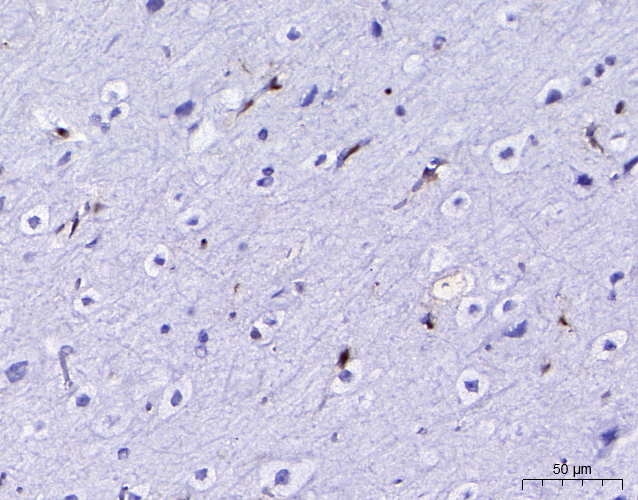

Supplement: Supplementary file 7 [file DataSheet9.ZIP › IHC Raw Image of p-CREB in striatum (2)/MX5 1-200 PCREB_20.0x.tif-W2.tif]

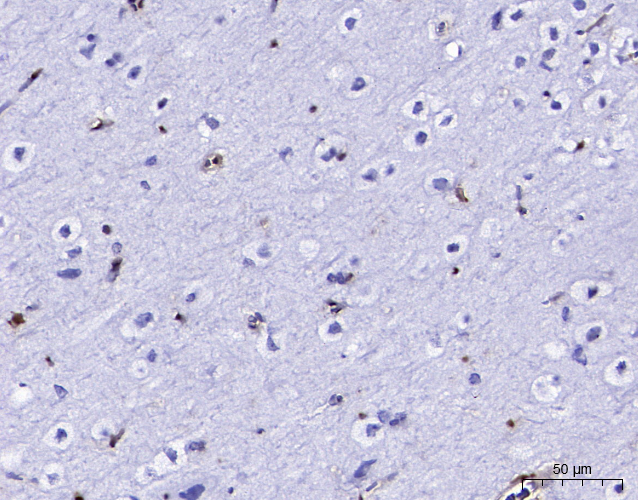

Supplement: Supplementary file 7 [file DataSheet9.ZIP › IHC Raw Image of p-CREB in striatum (2)/MX5 1-200 PCREB_20.0x.tif-W3.tif]

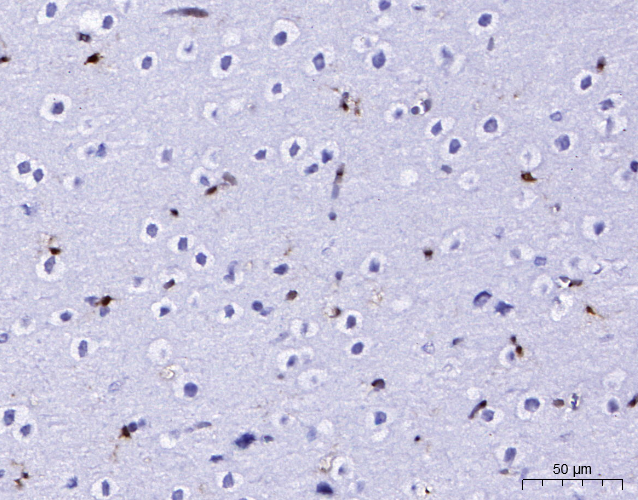

Supplement: Supplementary file 7 [file DataSheet9.ZIP › IHC Raw Image of p-CREB in striatum (2)/MX5 1-200 PCREB_20.0x.tif-W4.tif]

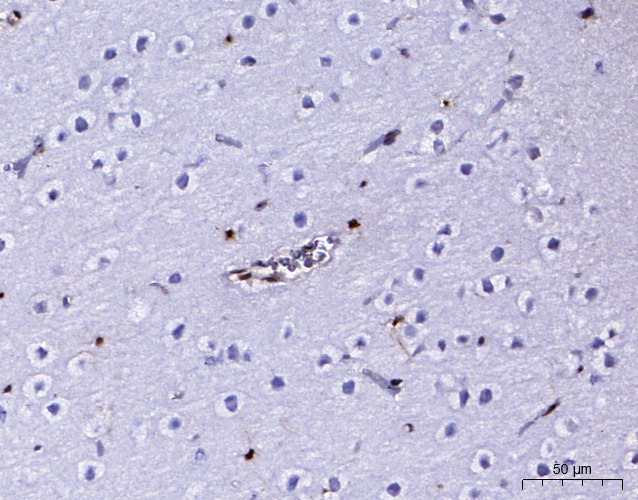

Supplement: Supplementary file 7 [file DataSheet9.ZIP › IHC Raw Image of p-CREB in striatum (2)/MX5 1-200 PCREB_20.0x.tif-W5.tif]

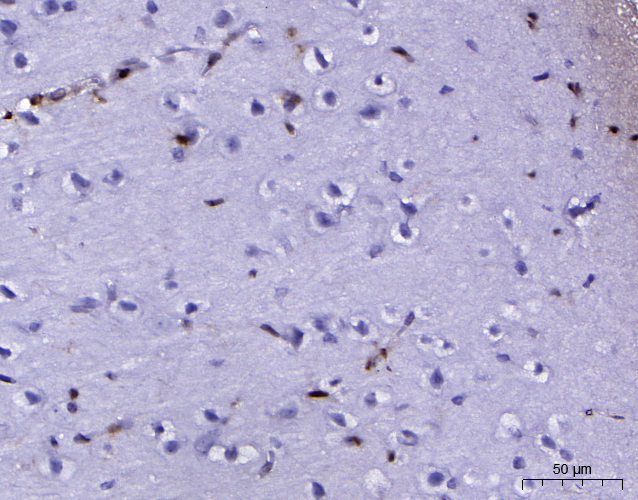

Supplement: Supplementary file 7 [file DataSheet9.ZIP › IHC Raw Image of p-CREB in striatum (2)/Z23 1-200 PCREB_20.0x.tif-W1.tif]

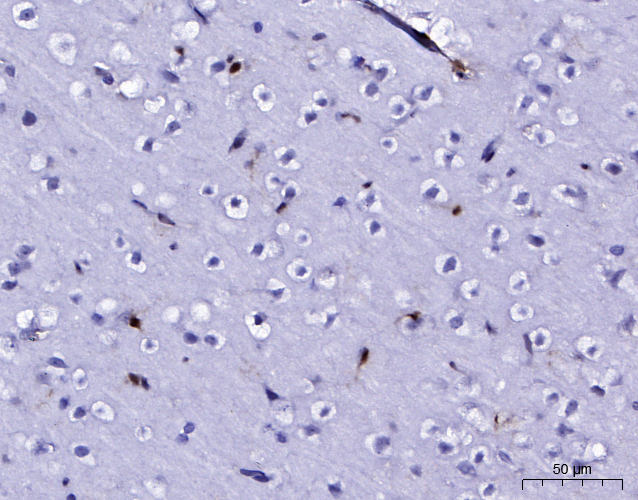

Supplement: Supplementary file 7 [file DataSheet9.ZIP › IHC Raw Image of p-CREB in striatum (2)/Z23 1-200 PCREB_20.0x.tif-W2.tif]

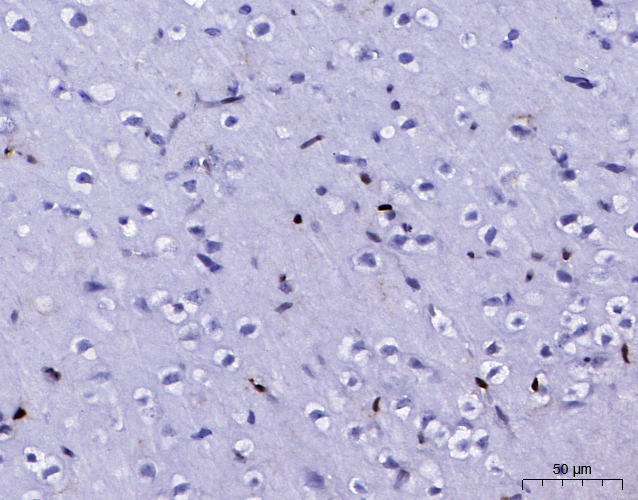

Supplement: Supplementary file 7 [file DataSheet9.ZIP › IHC Raw Image of p-CREB in striatum (2)/Z23 1-200 PCREB_20.0x.tif-W3.tif]

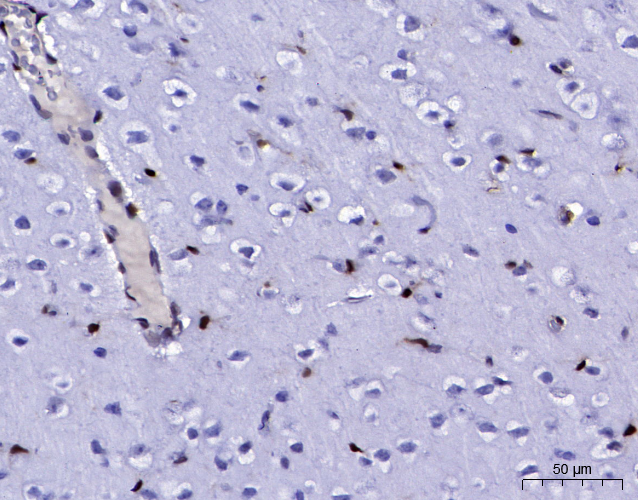

Supplement: Supplementary file 7 [file DataSheet9.ZIP › IHC Raw Image of p-CREB in striatum (2)/Z23 1-200 PCREB_20.0x.tif-W4.tif]

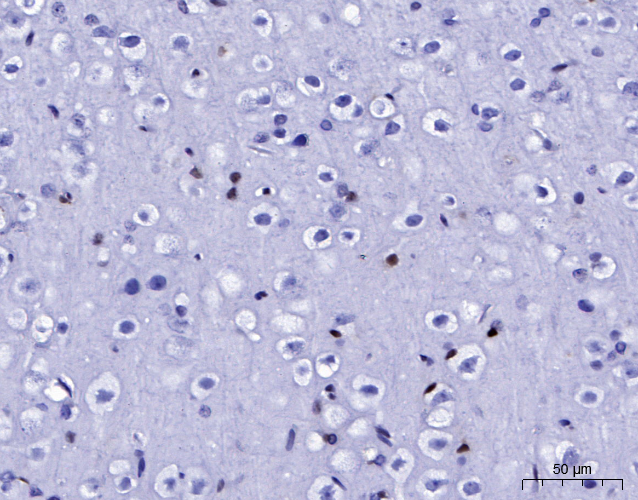

Supplement: Supplementary file 7 [file DataSheet9.ZIP › IHC Raw Image of p-CREB in striatum (2)/Z23 1-200 PCREB_20.0x.tif-W5.tif]

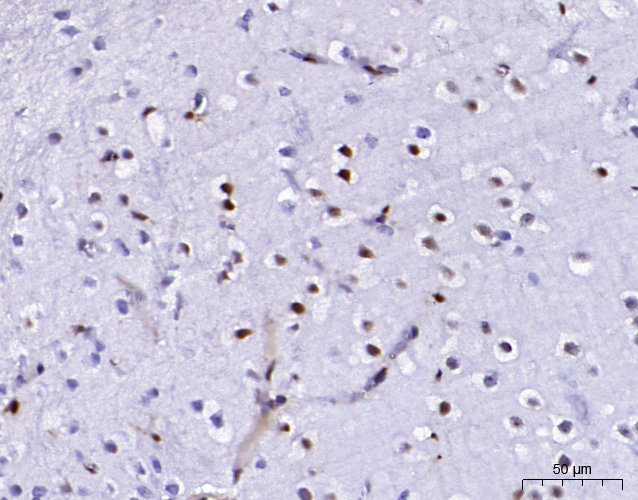

Supplement: Supplementary file 7 [file DataSheet9.ZIP › IHC Raw Image of p-CREB in striatum (2)/Z46 1-200 PCREB_20.0x.tif-W1.tif]

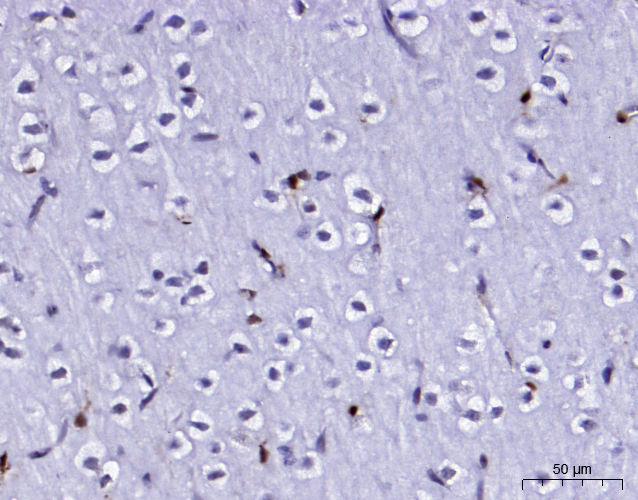

Supplement: Supplementary file 7 [file DataSheet9.ZIP › IHC Raw Image of p-CREB in striatum (2)/Z46 1-200 PCREB_20.0x.tif-W2.tif]

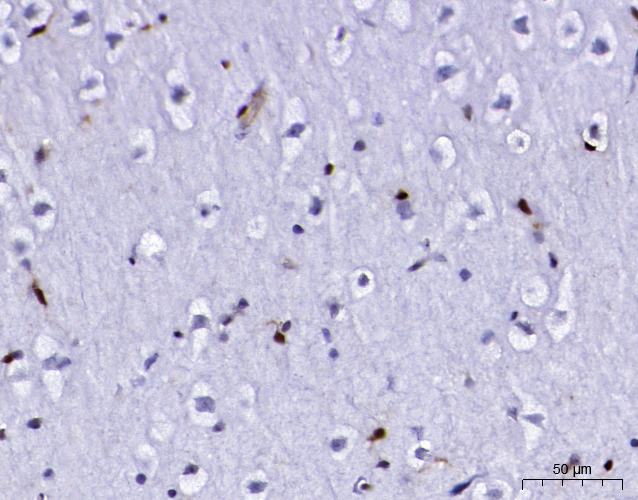

Supplement: Supplementary file 7 [file DataSheet9.ZIP › IHC Raw Image of p-CREB in striatum (2)/Z46 1-200 PCREB_20.0x.tif-W3.tif]

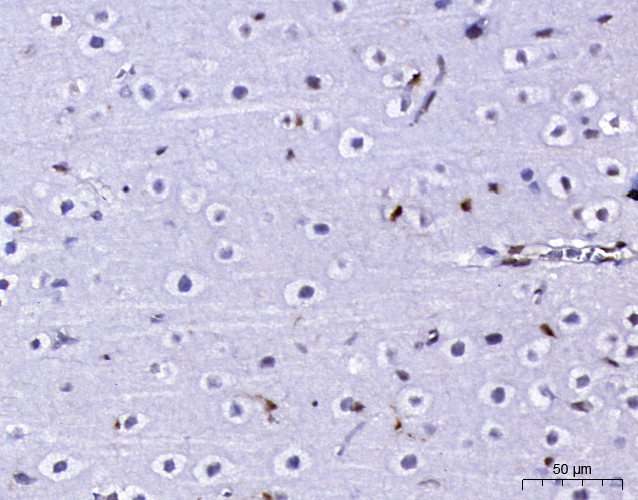

Supplement: Supplementary file 7 [file DataSheet9.ZIP › IHC Raw Image of p-CREB in striatum (2)/Z46 1-200 PCREB_20.0x.tif-W4.tif]

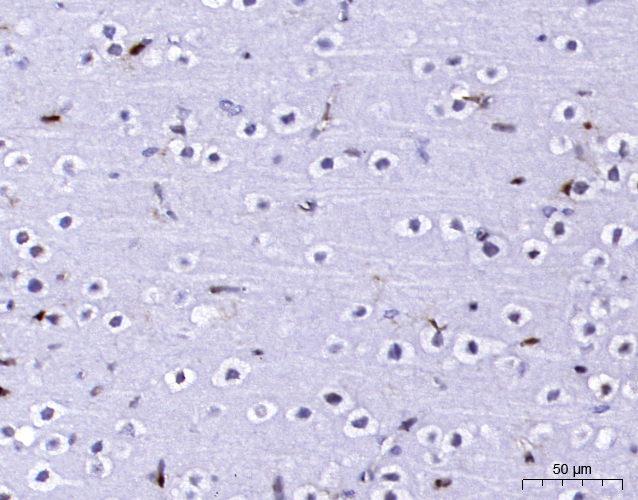

Supplement: Supplementary file 7 [file DataSheet9.ZIP › IHC Raw Image of p-CREB in striatum (2)/Z46 1-200 PCREB_20.0x.tif-W5.tif]

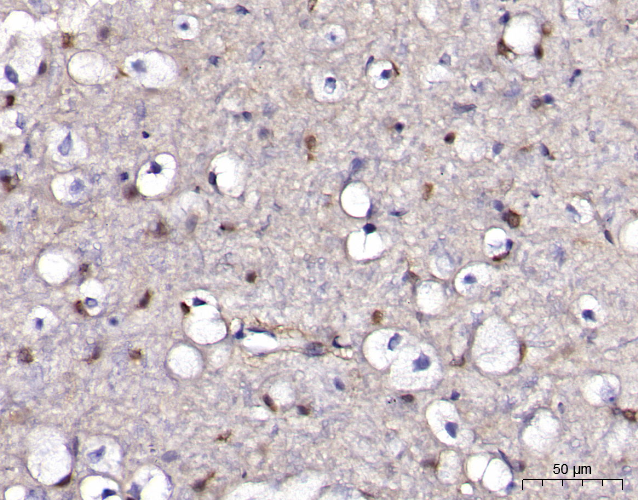

Supplement: Supplementary file 10 [file DataSheet4.ZIP › IHC Raw Image of BDNF in striatum (1)/K61 1-100 BDNF_20.0x.tif-W1.tif]

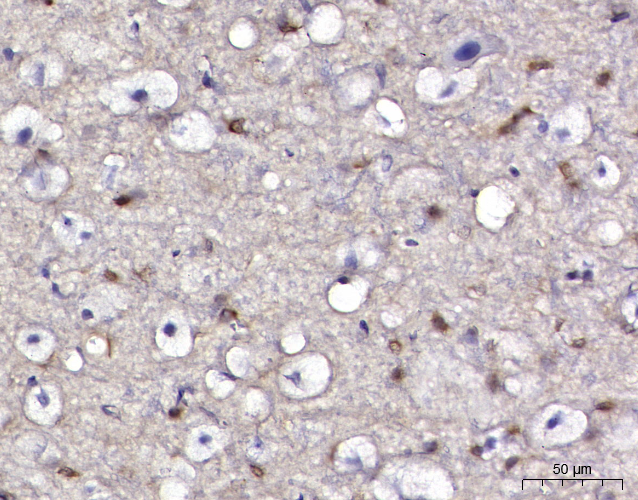

Supplement: Supplementary file 10 [file DataSheet4.ZIP › IHC Raw Image of BDNF in striatum (1)/K61 1-100 BDNF_20.0x.tif-W2.tif]

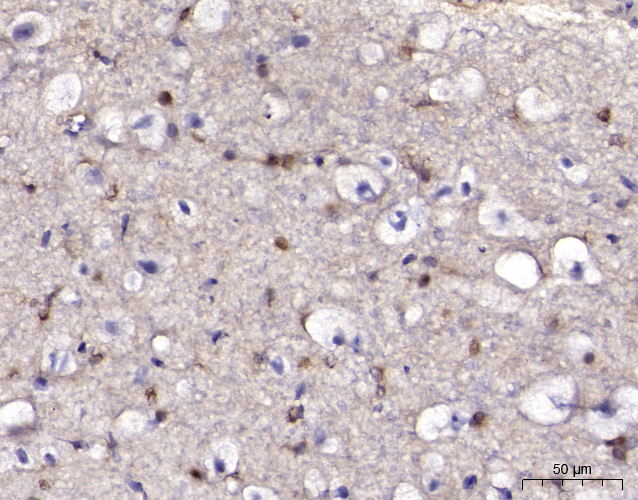

Supplement: Supplementary file 10 [file DataSheet4.ZIP › IHC Raw Image of BDNF in striatum (1)/K61 1-100 BDNF_20.0x.tif-W3.tif]

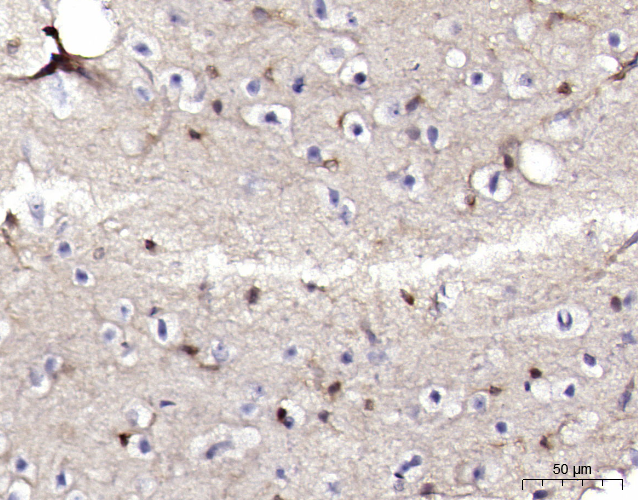

Supplement: Supplementary file 10 [file DataSheet4.ZIP › IHC Raw Image of BDNF in striatum (1)/K61 1-100 BDNF_20.0x.tif-W4.tif]

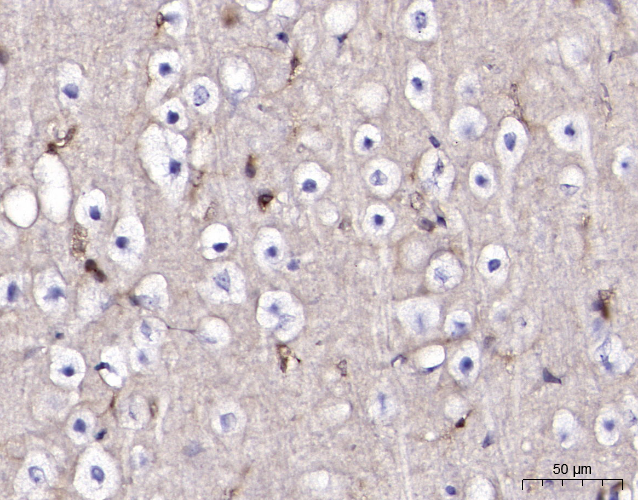

Supplement: Supplementary file 10 [file DataSheet4.ZIP › IHC Raw Image of BDNF in striatum (1)/K61 1-100 BDNF_20.0x.tif-W5.tif]

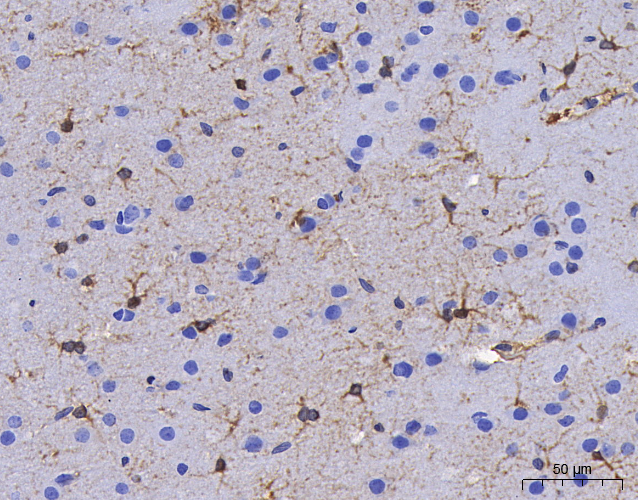

Supplement: Supplementary file 10 [file DataSheet4.ZIP › IHC Raw Image of BDNF in striatum (1)/K65 BDNF_20.0x.tif-W1.tif]

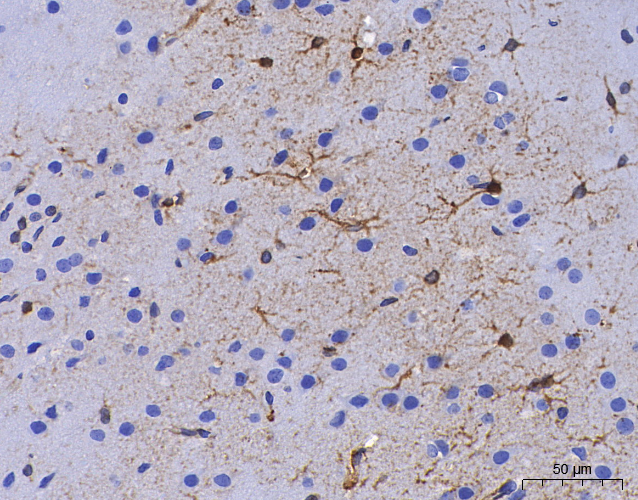

Supplement: Supplementary file 10 [file DataSheet4.ZIP › IHC Raw Image of BDNF in striatum (1)/K65 BDNF_20.0x.tif-W2.tif]

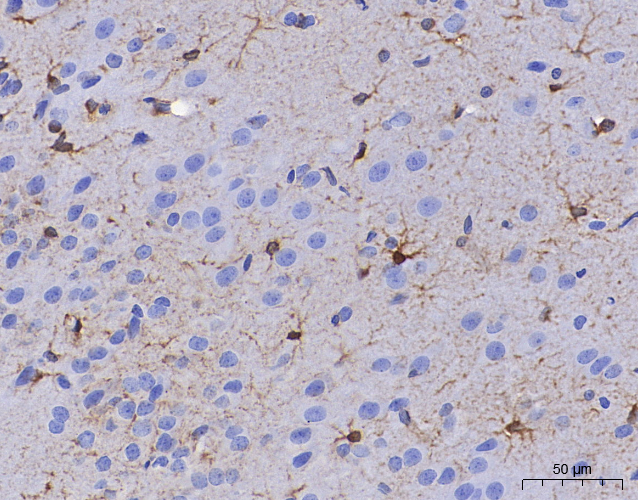

Supplement: Supplementary file 10 [file DataSheet4.ZIP › IHC Raw Image of BDNF in striatum (1)/K65 BDNF_20.0x.tif-W3.tif]

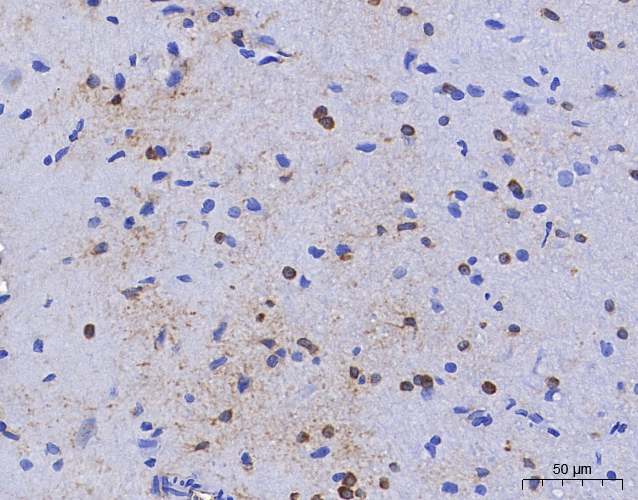

Supplement: Supplementary file 10 [file DataSheet4.ZIP › IHC Raw Image of BDNF in striatum (1)/K65 BDNF_20.0x.tif-W4.tif]

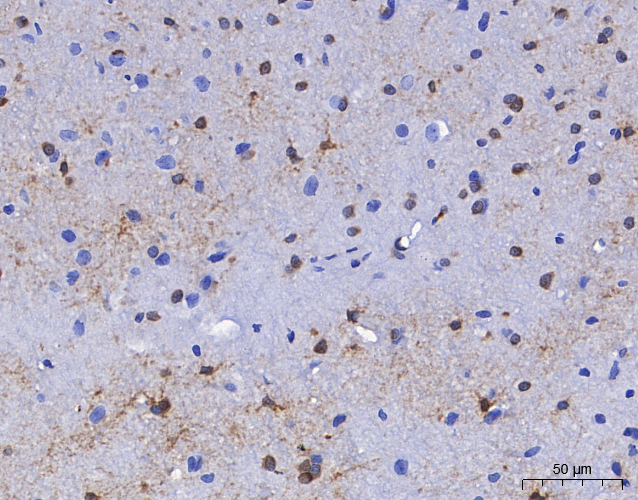

Supplement: Supplementary file 10 [file DataSheet4.ZIP › IHC Raw Image of BDNF in striatum (1)/K65 BDNF_20.0x.tif-W5.tif]

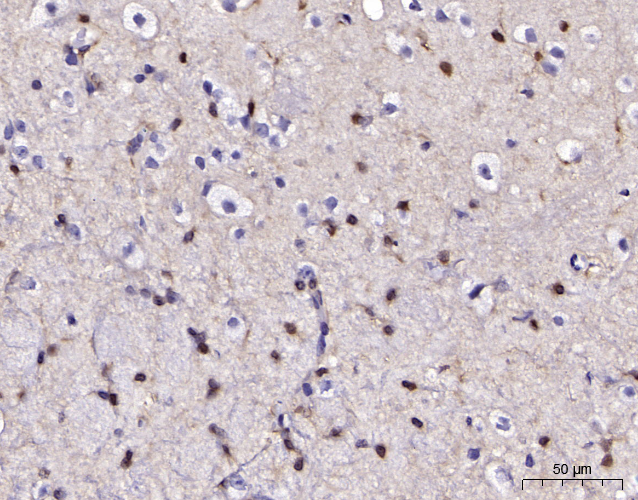

Supplement: Supplementary file 10 [file DataSheet4.ZIP › IHC Raw Image of BDNF in striatum (1)/K67 1-100 BDNF_20.0x.tif-W1.tif]

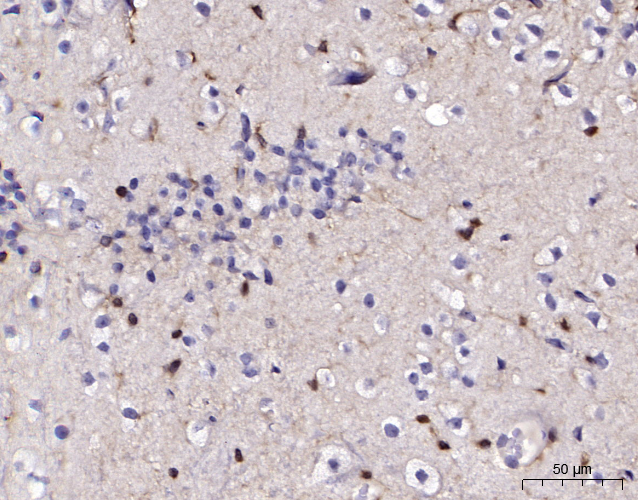

Supplement: Supplementary file 10 [file DataSheet4.ZIP › IHC Raw Image of BDNF in striatum (1)/K67 1-100 BDNF_20.0x.tif-W2.tif]

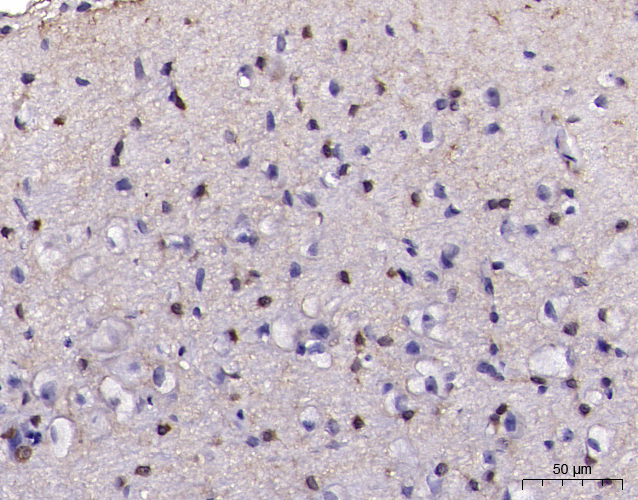

Supplement: Supplementary file 10 [file DataSheet4.ZIP › IHC Raw Image of BDNF in striatum (1)/K67 1-100 BDNF_20.0x.tif-W3.tif]

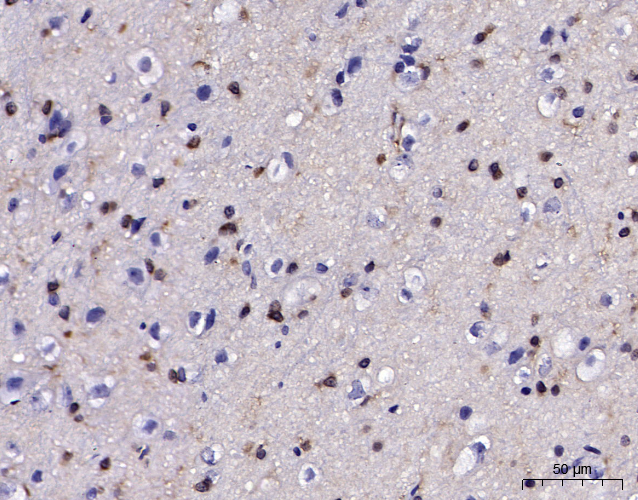

Supplement: Supplementary file 10 [file DataSheet4.ZIP › IHC Raw Image of BDNF in striatum (1)/K67 1-100 BDNF_20.0x.tif-W4.tif]

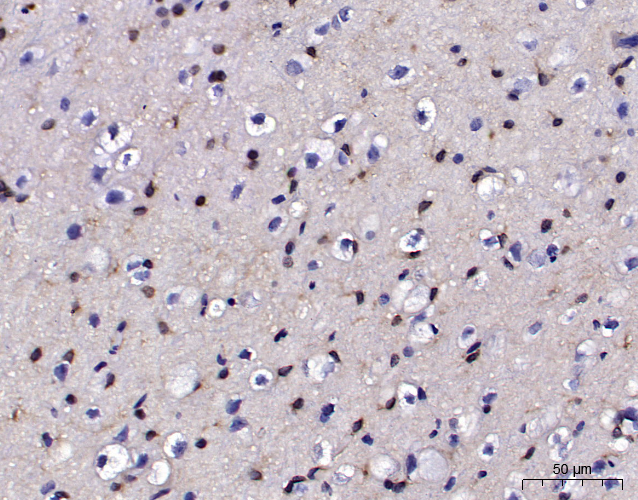

Supplement: Supplementary file 10 [file DataSheet4.ZIP › IHC Raw Image of BDNF in striatum (1)/K67 1-100 BDNF_20.0x.tif-W5.tif]

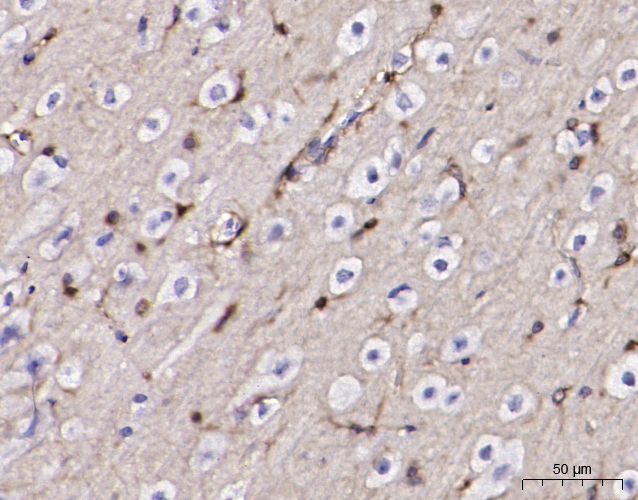

Supplement: Supplementary file 10 [file DataSheet4.ZIP › IHC Raw Image of BDNF in striatum (1)/K69 1-100 BDNF_20.0x.tif-W1.tif]

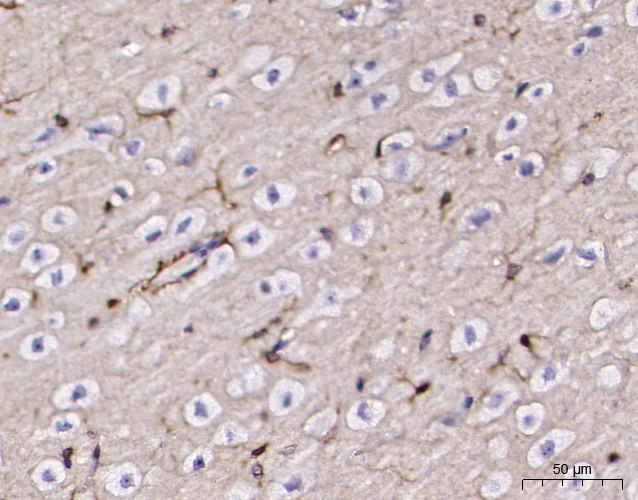

Supplement: Supplementary file 10 [file DataSheet4.ZIP › IHC Raw Image of BDNF in striatum (1)/K69 1-100 BDNF_20.0x.tif-W2.tif]

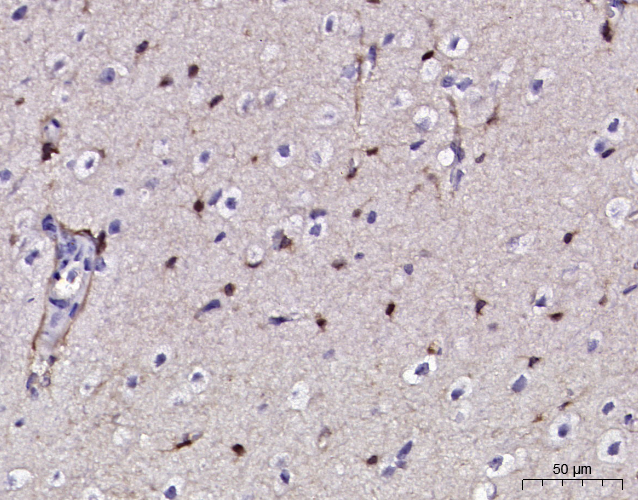

Supplement: Supplementary file 10 [file DataSheet4.ZIP › IHC Raw Image of BDNF in striatum (1)/K69 1-100 BDNF_20.0x.tif-W3.tif]

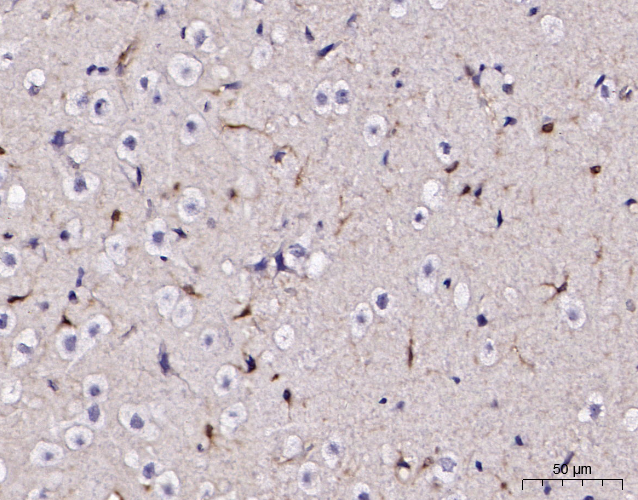

Supplement: Supplementary file 10 [file DataSheet4.ZIP › IHC Raw Image of BDNF in striatum (1)/K69 1-100 BDNF_20.0x.tif-W4.tif]

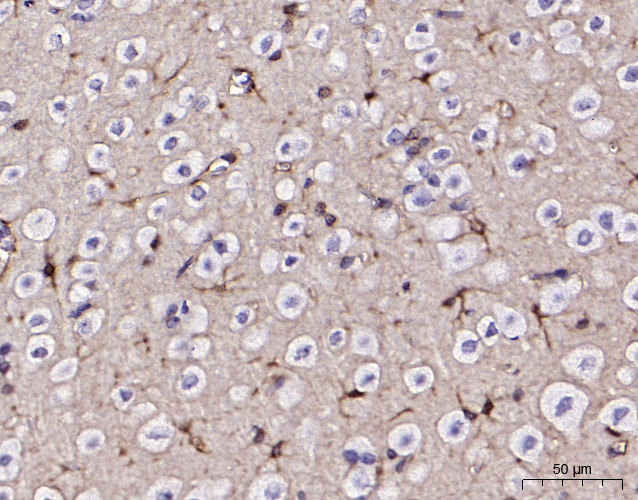

Supplement: Supplementary file 10 [file DataSheet4.ZIP › IHC Raw Image of BDNF in striatum (1)/K69 1-100 BDNF_20.0x.tif-W5.tif]

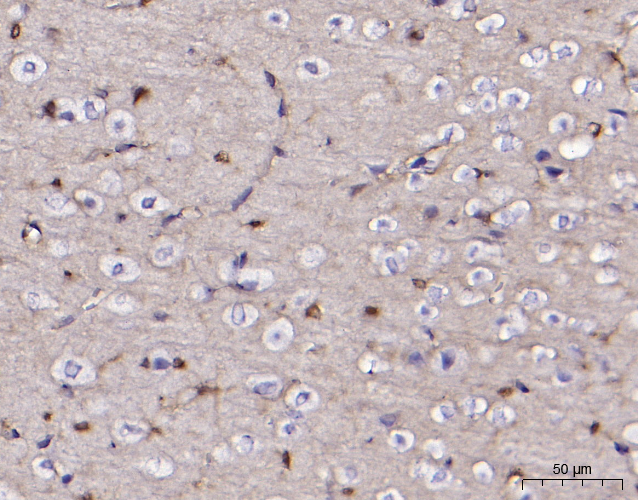

Supplement: Supplementary file 10 [file DataSheet4.ZIP › IHC Raw Image of BDNF in striatum (1)/L7 1-100 BDNF_20.0x.tif-W1.tif]

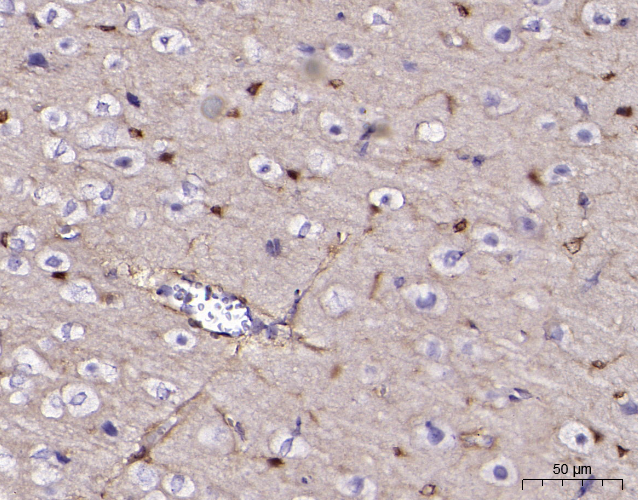

Supplement: Supplementary file 10 [file DataSheet4.ZIP › IHC Raw Image of BDNF in striatum (1)/L7 1-100 BDNF_20.0x.tif-W2.tif]

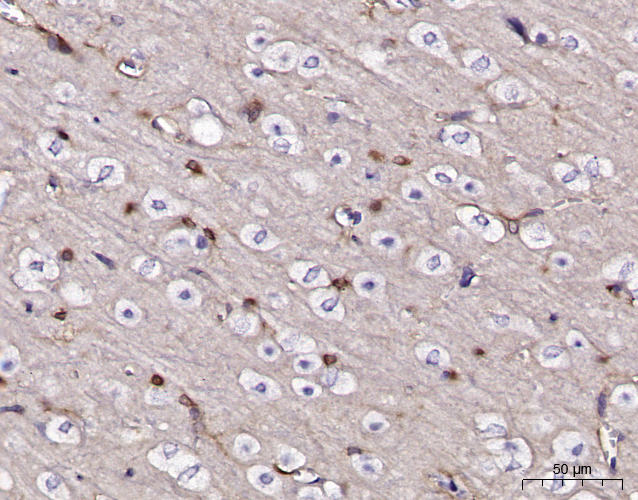

Supplement: Supplementary file 10 [file DataSheet4.ZIP › IHC Raw Image of BDNF in striatum (1)/L7 1-100 BDNF_20.0x.tif-W3.tif]

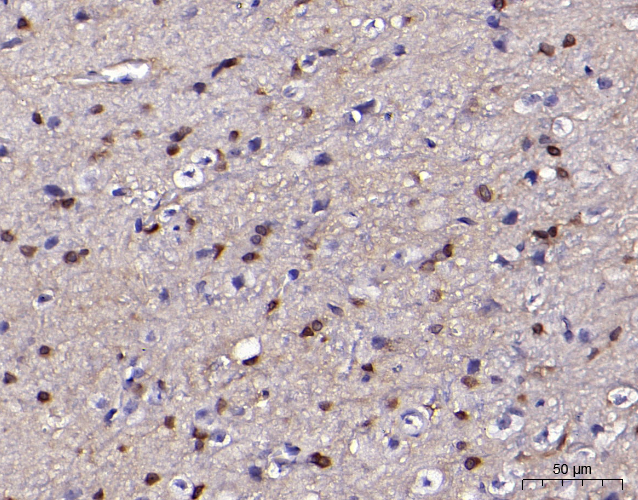

Supplement: Supplementary file 10 [file DataSheet4.ZIP › IHC Raw Image of BDNF in striatum (1)/L7 1-100 BDNF_20.0x.tif-W4.tif]

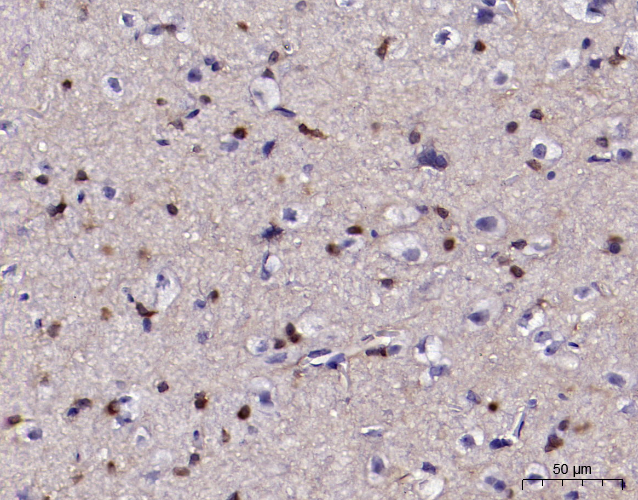

Supplement: Supplementary file 10 [file DataSheet4.ZIP › IHC Raw Image of BDNF in striatum (1)/L7 1-100 BDNF_20.0x.tif-W5.tif]

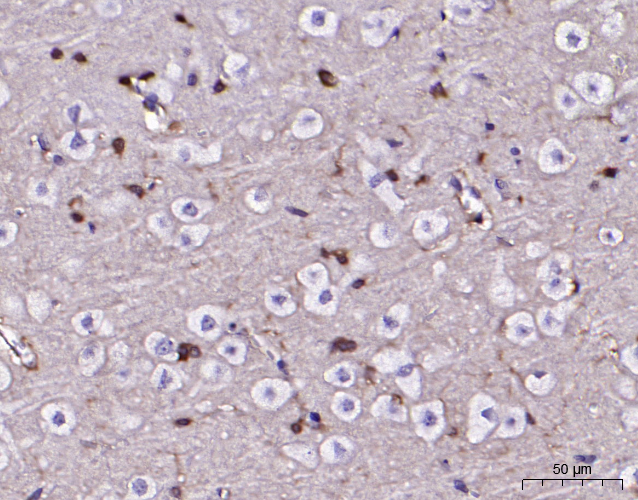

Supplement: Supplementary file 10 [file DataSheet4.ZIP › IHC Raw Image of BDNF in striatum (1)/L9 1-100 BDNF_20.0x.tif-W1.tif]

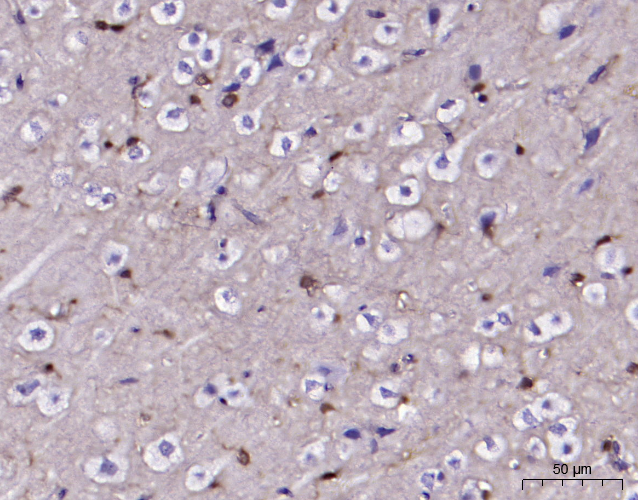

Supplement: Supplementary file 10 [file DataSheet4.ZIP › IHC Raw Image of BDNF in striatum (1)/L9 1-100 BDNF_20.0x.tif-W2.tif]

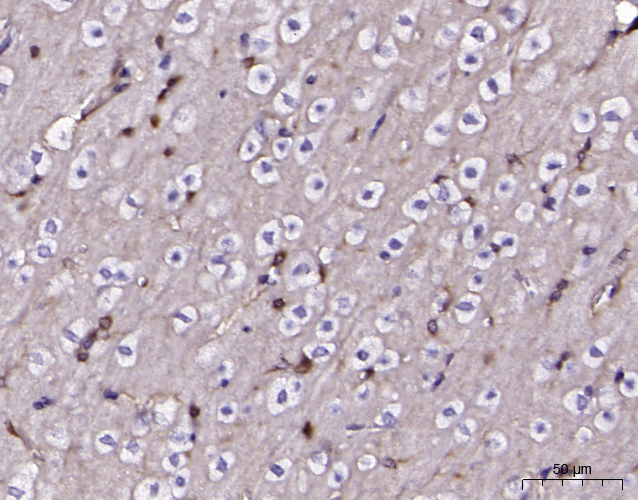

Supplement: Supplementary file 10 [file DataSheet4.ZIP › IHC Raw Image of BDNF in striatum (1)/L9 1-100 BDNF_20.0x.tif-W3.tif]

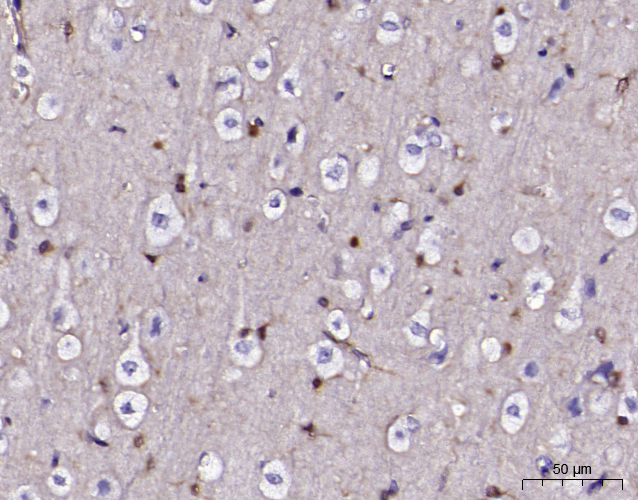

Supplement: Supplementary file 10 [file DataSheet4.ZIP › IHC Raw Image of BDNF in striatum (1)/L9 1-100 BDNF_20.0x.tif-W4.tif]

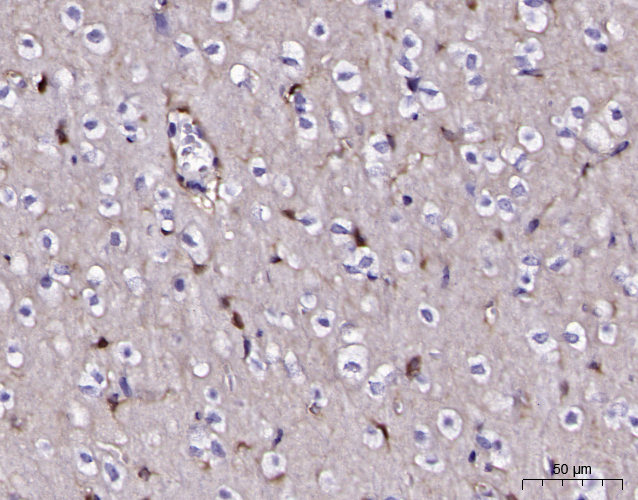

Supplement: Supplementary file 10 [file DataSheet4.ZIP › IHC Raw Image of BDNF in striatum (1)/L9 1-100 BDNF_20.0x.tif-W5.tif]

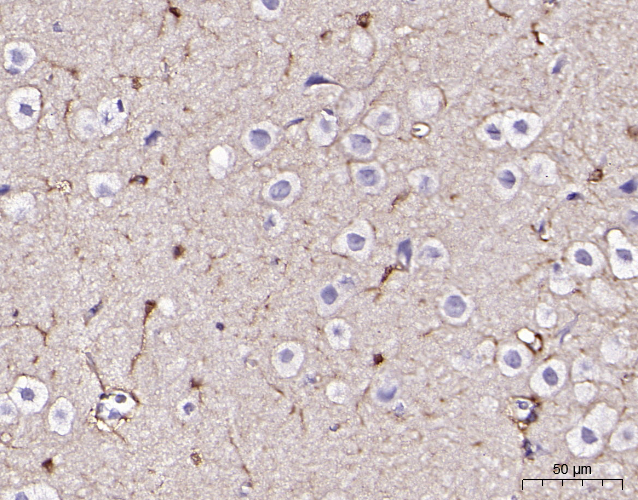

Supplement: Supplementary file 12 [file DataSheet1.ZIP › IHC Raw image of BDNF in PFC(1)/K61 1-100 BDNF_20.0x.tif-Q1.tif]

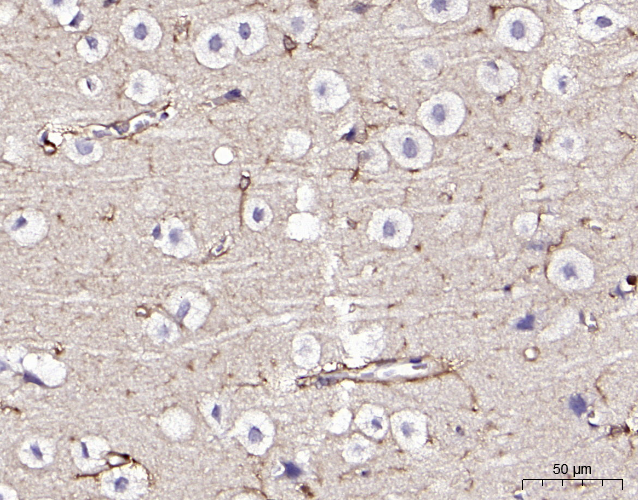

Supplement: Supplementary file 12 [file DataSheet1.ZIP › IHC Raw image of BDNF in PFC(1)/K61 1-100 BDNF_20.0x.tif-Q2.tif]

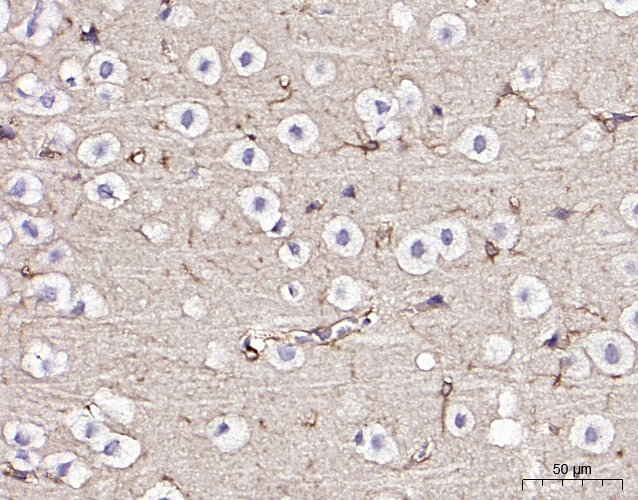

Supplement: Supplementary file 12 [file DataSheet1.ZIP › IHC Raw image of BDNF in PFC(1)/K61 1-100 BDNF_20.0x.tif-Q3.tif]

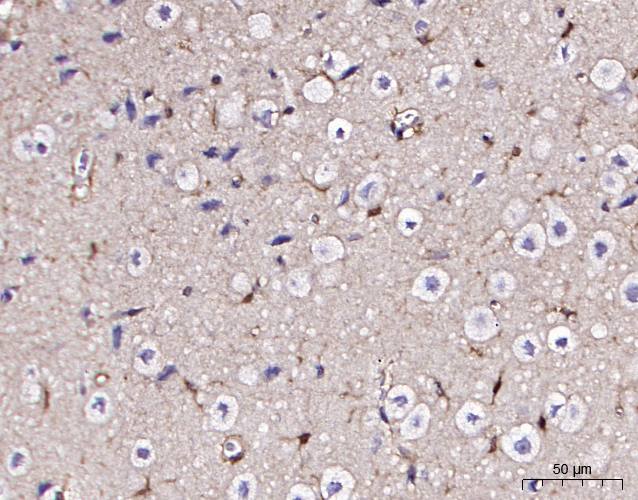

Supplement: Supplementary file 12 [file DataSheet1.ZIP › IHC Raw image of BDNF in PFC(1)/K61 1-100 BDNF_20.0x.tif-Q4.tif]

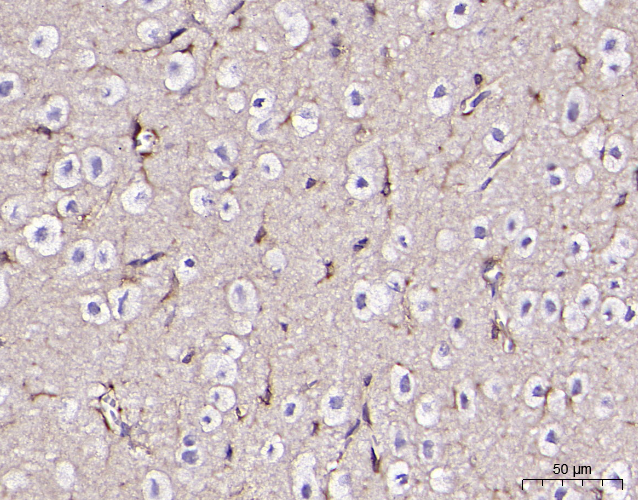

Supplement: Supplementary file 12 [file DataSheet1.ZIP › IHC Raw image of BDNF in PFC(1)/K61 1-100 BDNF_20.0x.tif-Q5.tif]

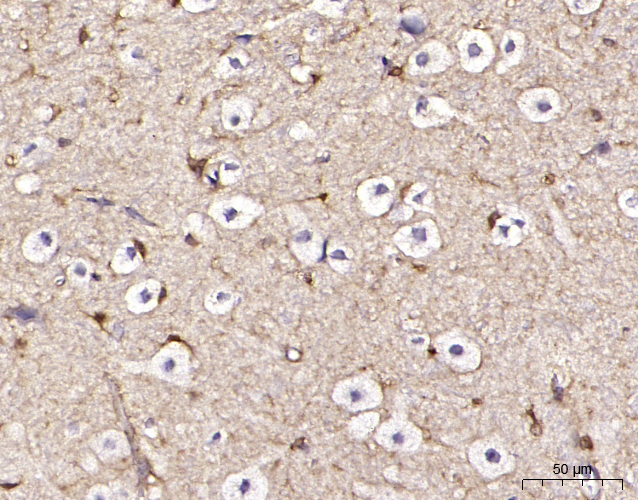

Supplement: Supplementary file 12 [file DataSheet1.ZIP › IHC Raw image of BDNF in PFC(1)/K65 1-100 BDNF_20.0x.tif-Q1.tif]

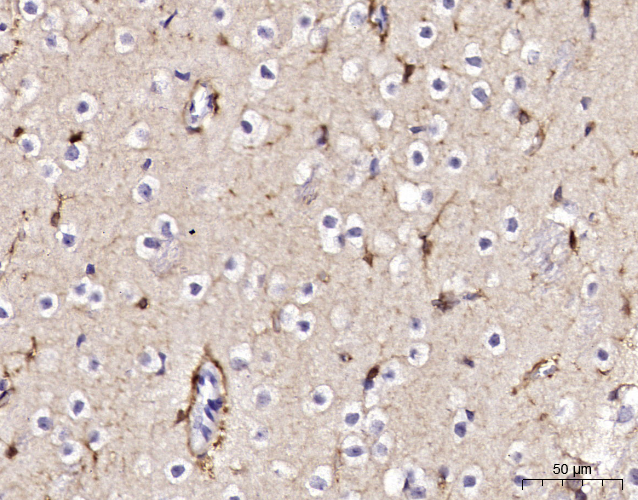

Supplement: Supplementary file 12 [file DataSheet1.ZIP › IHC Raw image of BDNF in PFC(1)/K65 1-100 BDNF_20.0x.tif-Q2.tif]

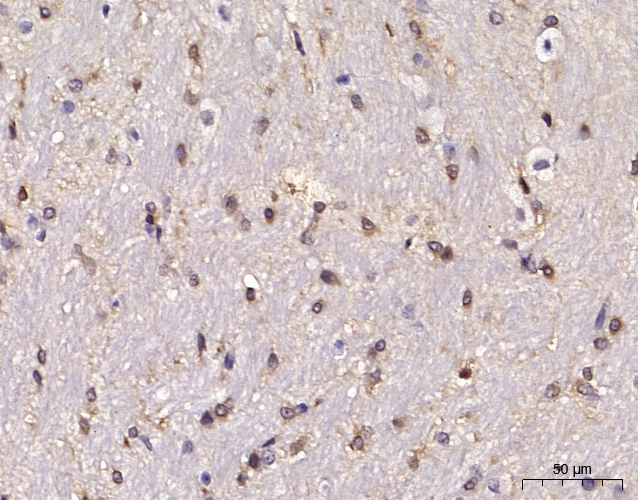

Supplement: Supplementary file 12 [file DataSheet1.ZIP › IHC Raw image of BDNF in PFC(1)/K65 1-100 BDNF_20.0x.tif-Q3.tif]

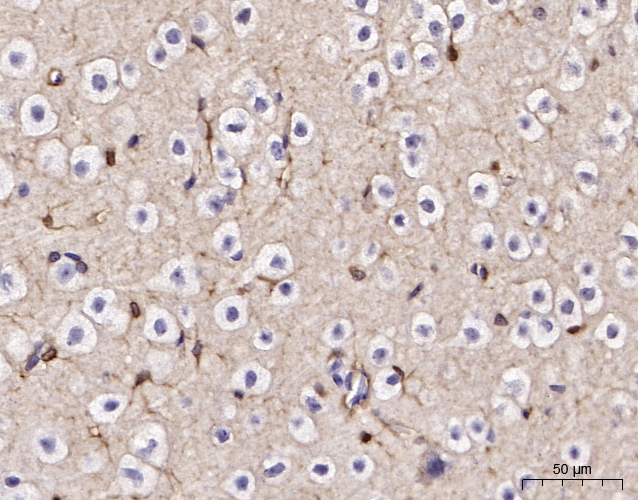

Supplement: Supplementary file 12 [file DataSheet1.ZIP › IHC Raw image of BDNF in PFC(1)/K65 1-100 BDNF_20.0x.tif-Q4.tif]

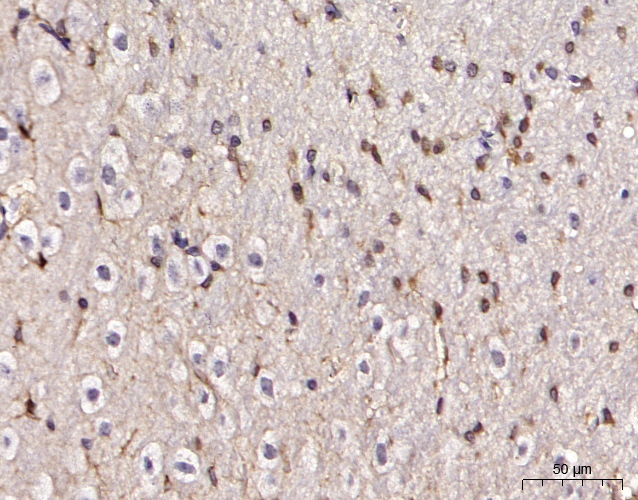

Supplement: Supplementary file 12 [file DataSheet1.ZIP › IHC Raw image of BDNF in PFC(1)/K65 1-100 BDNF_20.0x.tif-Q5.tif]

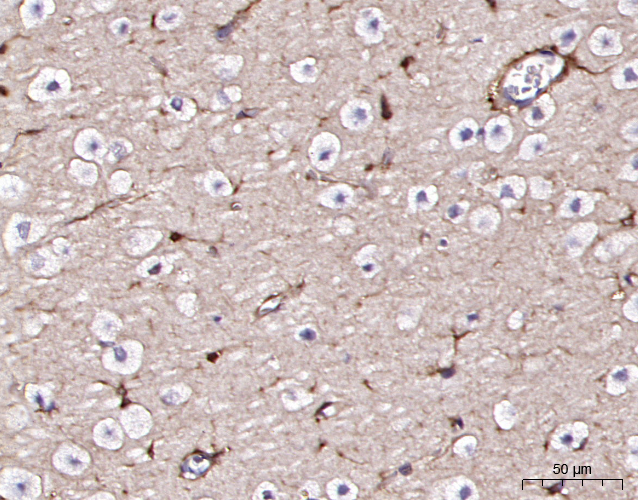

Supplement: Supplementary file 12 [file DataSheet1.ZIP › IHC Raw image of BDNF in PFC(1)/K67 1-100 BDNF_20.0x.tif-Q1.tif]

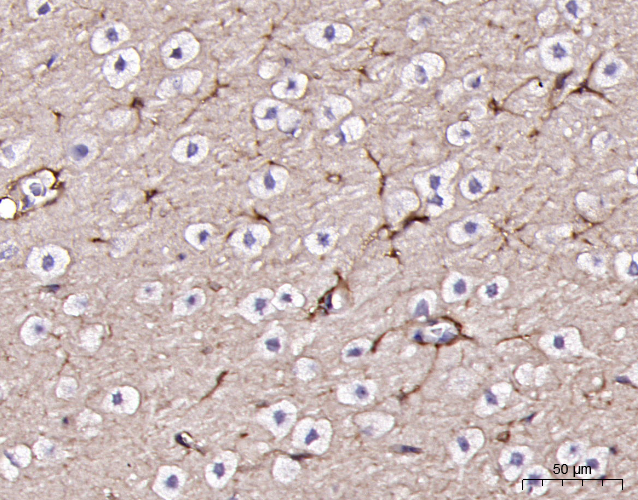

Supplement: Supplementary file 12 [file DataSheet1.ZIP › IHC Raw image of BDNF in PFC(1)/K67 1-100 BDNF_20.0x.tif-Q2.tif]

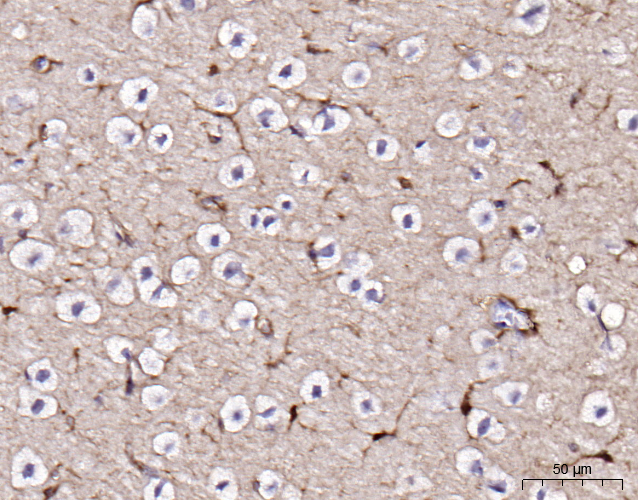

Supplement: Supplementary file 12 [file DataSheet1.ZIP › IHC Raw image of BDNF in PFC(1)/K67 1-100 BDNF_20.0x.tif-Q3.tif]

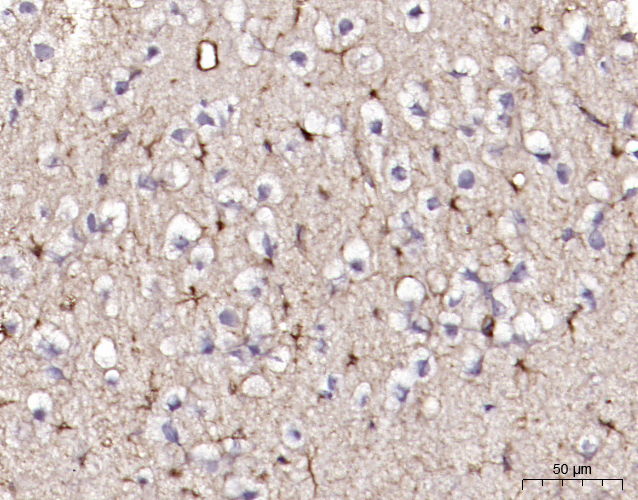

Supplement: Supplementary file 12 [file DataSheet1.ZIP › IHC Raw image of BDNF in PFC(1)/K67 1-100 BDNF_20.0x.tif-Q4.tif]

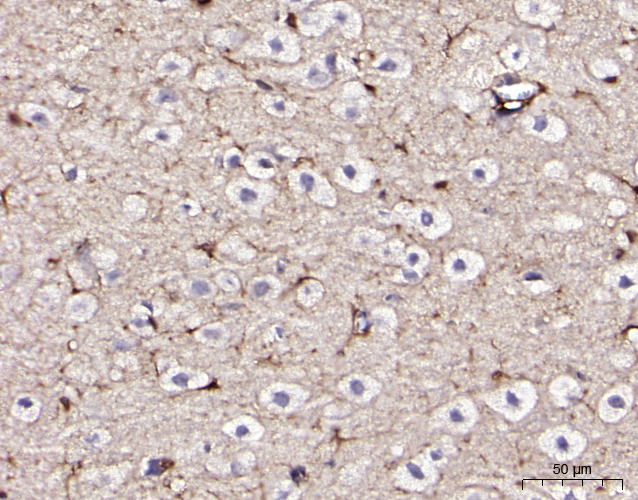

Supplement: Supplementary file 12 [file DataSheet1.ZIP › IHC Raw image of BDNF in PFC(1)/K67 1-100 BDNF_20.0x.tif-Q5.tif]

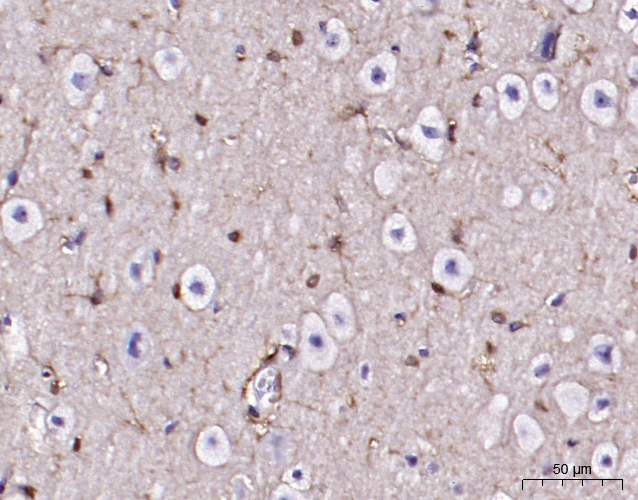

Supplement: Supplementary file 12 [file DataSheet1.ZIP › IHC Raw image of BDNF in PFC(1)/K69 1-100 BDNF_20.0x.tif-Q1.tif]

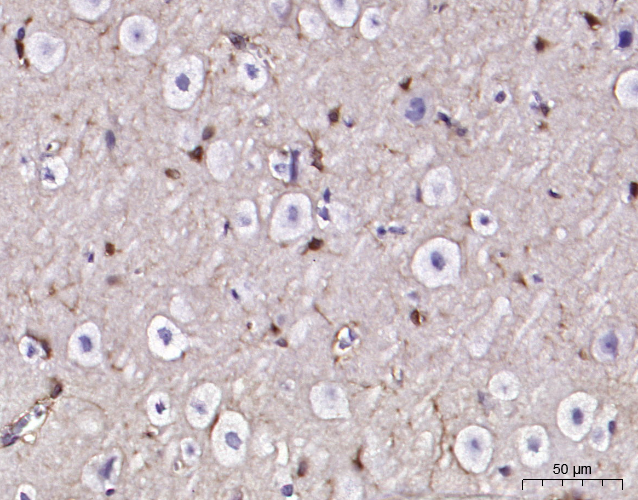

Supplement: Supplementary file 12 [file DataSheet1.ZIP › IHC Raw image of BDNF in PFC(1)/K69 1-100 BDNF_20.0x.tif-Q2.tif]

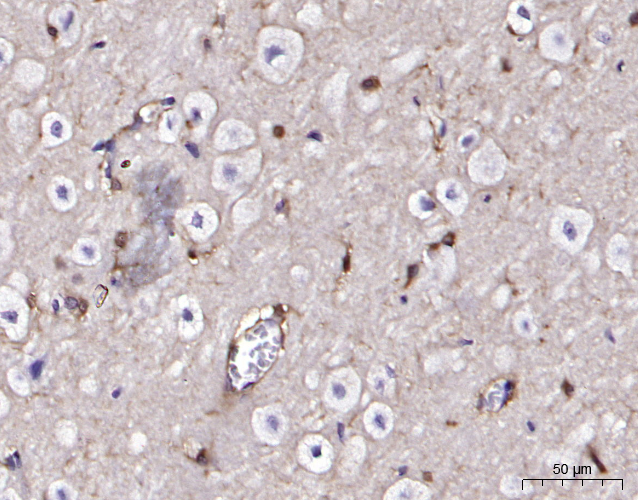

Supplement: Supplementary file 12 [file DataSheet1.ZIP › IHC Raw image of BDNF in PFC(1)/K69 1-100 BDNF_20.0x.tif-Q3.tif]

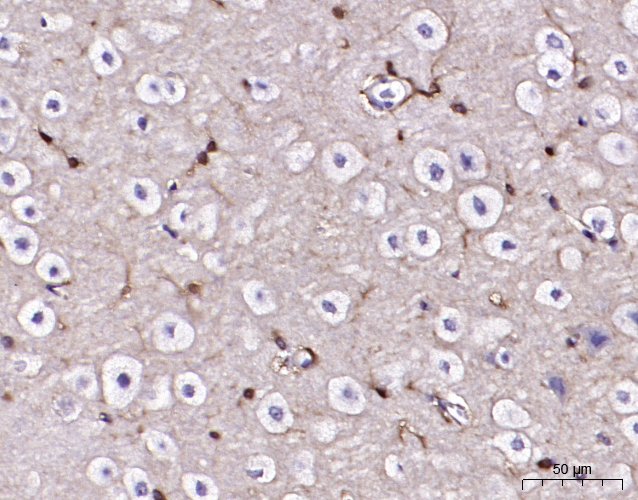

Supplement: Supplementary file 12 [file DataSheet1.ZIP › IHC Raw image of BDNF in PFC(1)/K69 1-100 BDNF_20.0x.tif-Q4.tif]

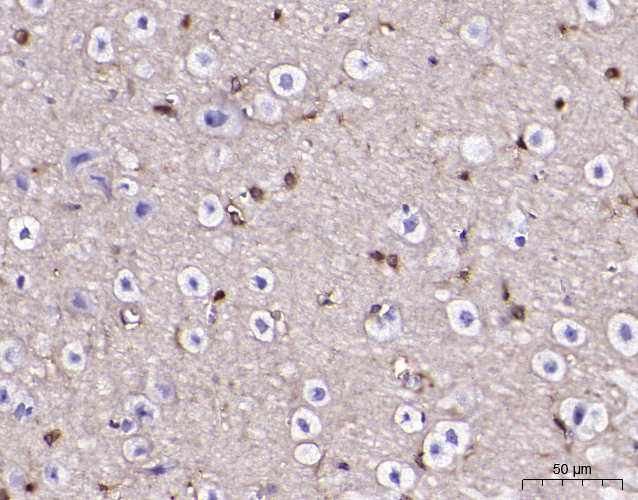

Supplement: Supplementary file 12 [file DataSheet1.ZIP › IHC Raw image of BDNF in PFC(1)/K69 1-100 BDNF_20.0x.tif-Q5.tif]

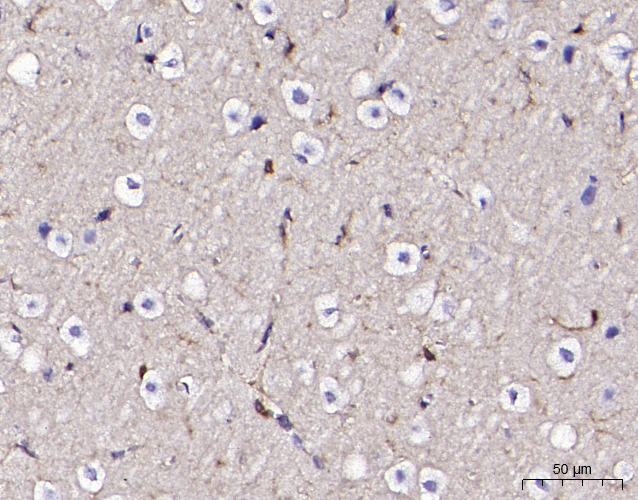

Supplement: Supplementary file 12 [file DataSheet1.ZIP › IHC Raw image of BDNF in PFC(1)/L7 1-100 BDNF_20.0x.tif-Q1.tif]

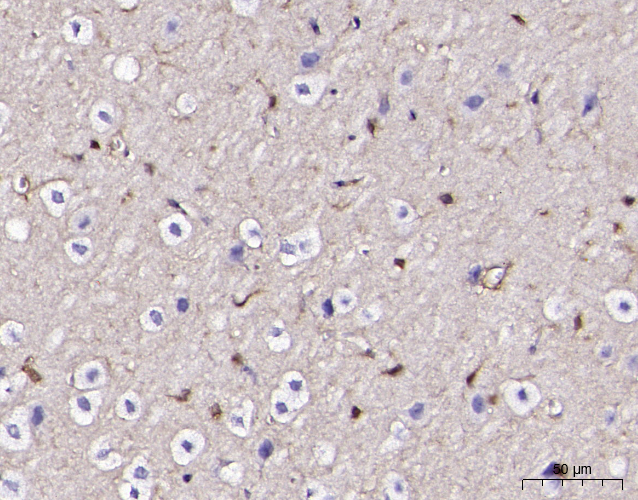

Supplement: Supplementary file 12 [file DataSheet1.ZIP › IHC Raw image of BDNF in PFC(1)/L7 1-100 BDNF_20.0x.tif-Q2.tif]

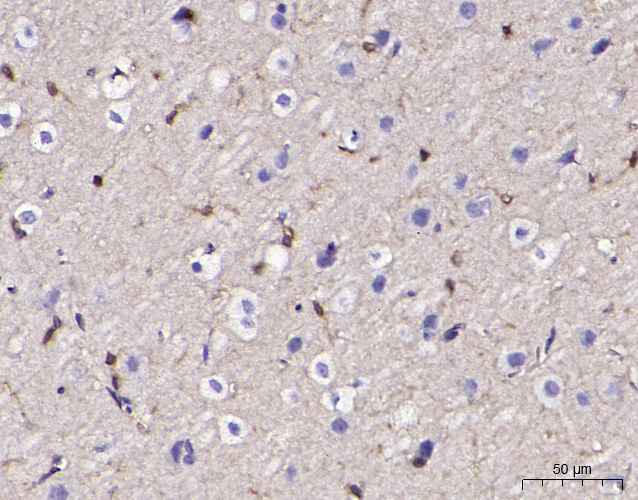

Supplement: Supplementary file 12 [file DataSheet1.ZIP › IHC Raw image of BDNF in PFC(1)/L7 1-100 BDNF_20.0x.tif-Q3.tif]

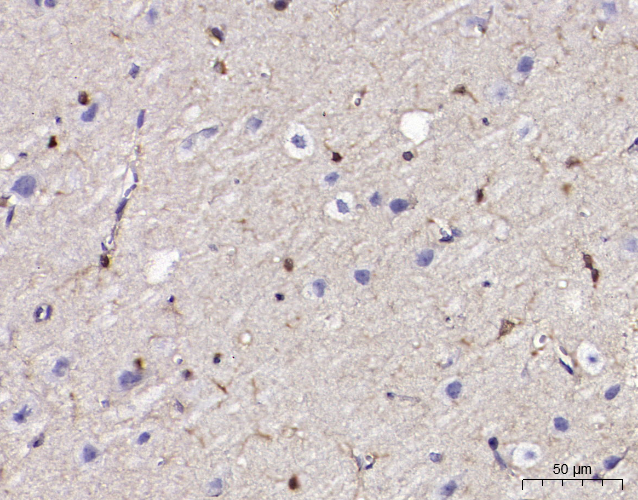

Supplement: Supplementary file 12 [file DataSheet1.ZIP › IHC Raw image of BDNF in PFC(1)/L7 1-100 BDNF_20.0x.tif-Q4.tif]

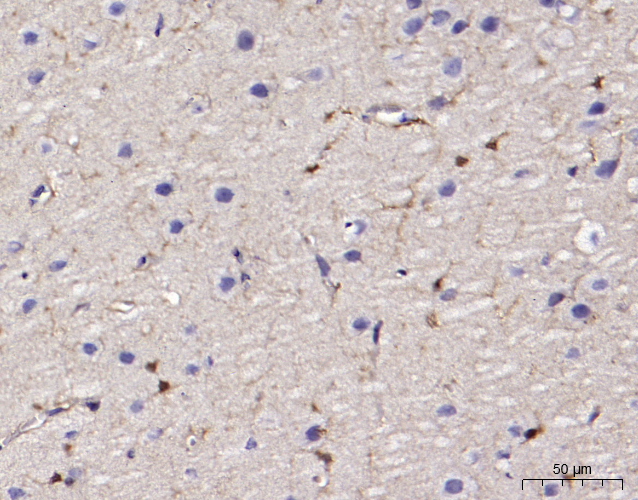

Supplement: Supplementary file 12 [file DataSheet1.ZIP › IHC Raw image of BDNF in PFC(1)/L7 1-100 BDNF_20.0x.tif-Q5.tif]

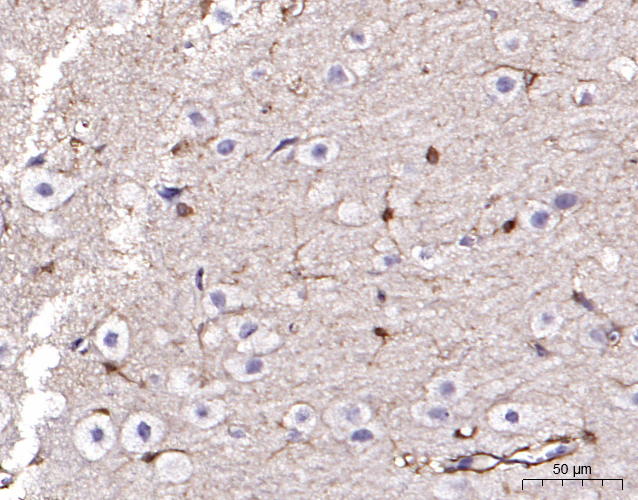

Supplement: Supplementary file 12 [file DataSheet1.ZIP › IHC Raw image of BDNF in PFC(1)/L9 1-100 BDNF_20.0x.tif-Q1.tif]

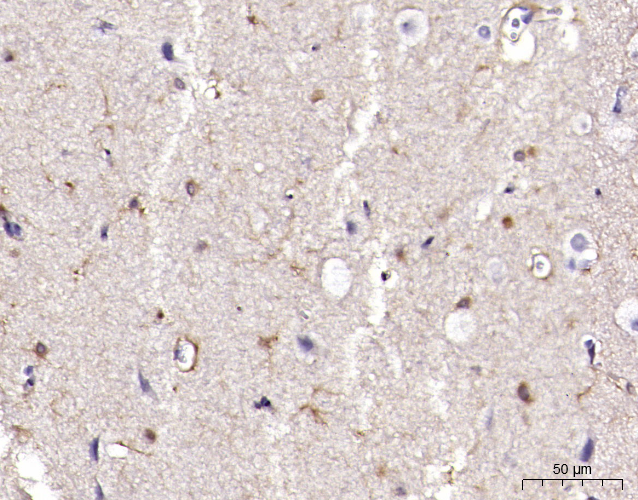

Supplement: Supplementary file 12 [file DataSheet1.ZIP › IHC Raw image of BDNF in PFC(1)/L9 1-100 BDNF_20.0x.tif-Q2.tif]

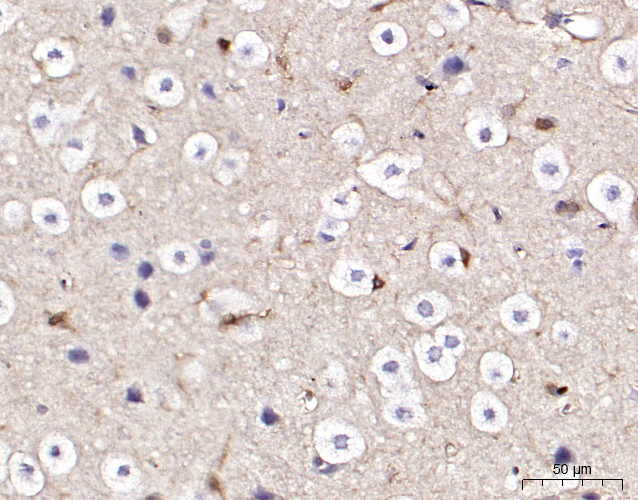

Supplement: Supplementary file 12 [file DataSheet1.ZIP › IHC Raw image of BDNF in PFC(1)/L9 1-100 BDNF_20.0x.tif-Q3.tif]

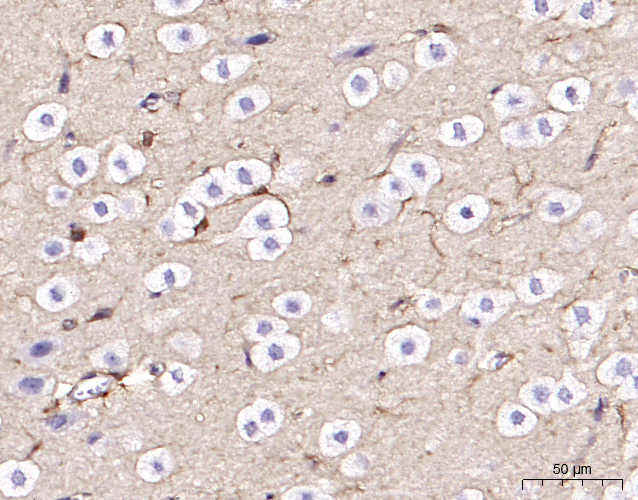

Supplement: Supplementary file 12 [file DataSheet1.ZIP › IHC Raw image of BDNF in PFC(1)/L9 1-100 BDNF_20.0x.tif-Q4.tif]

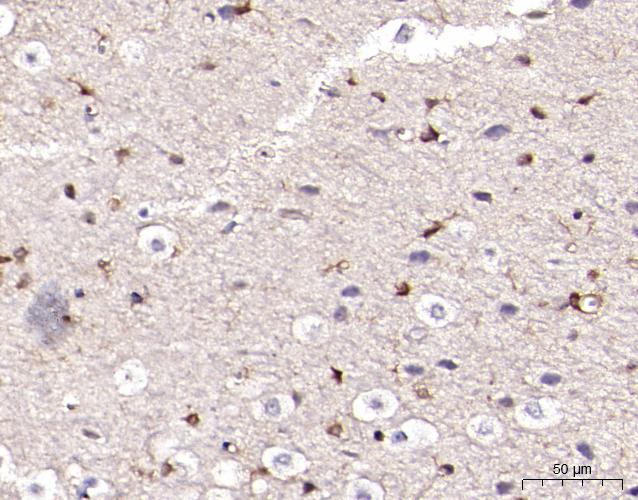

Supplement: Supplementary file 12 [file DataSheet1.ZIP › IHC Raw image of BDNF in PFC(1)/L9 1-100 BDNF_20.0x.tif-Q5.tif]

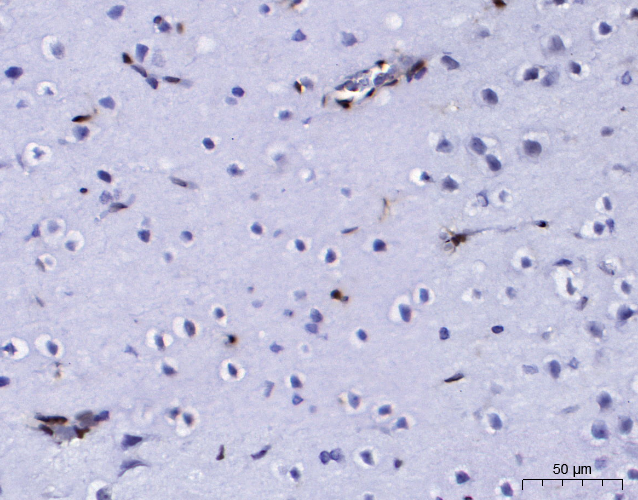

Supplement: Supplementary file 13 [file DataSheet10.ZIP › IHC Raw Image of p-CREB in striatum (3)/H34 1-200 PCREB_20.0x.tif-W1.tif]

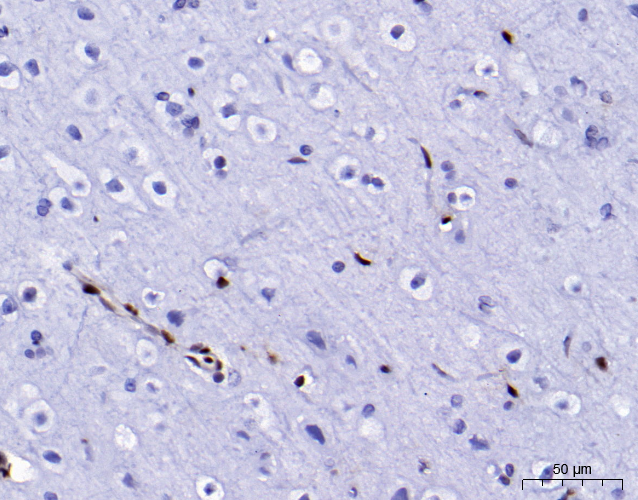

Supplement: Supplementary file 13 [file DataSheet10.ZIP › IHC Raw Image of p-CREB in striatum (3)/H34 1-200 PCREB_20.0x.tif-W2.tif]

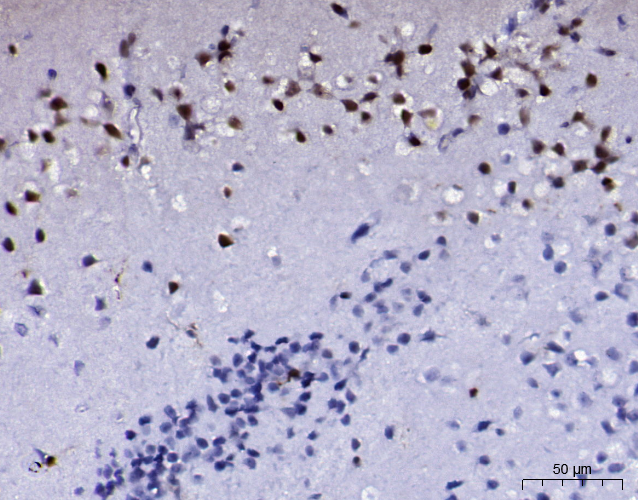

Supplement: Supplementary file 13 [file DataSheet10.ZIP › IHC Raw Image of p-CREB in striatum (3)/H34 1-200 PCREB_20.0x.tif-W3.tif]

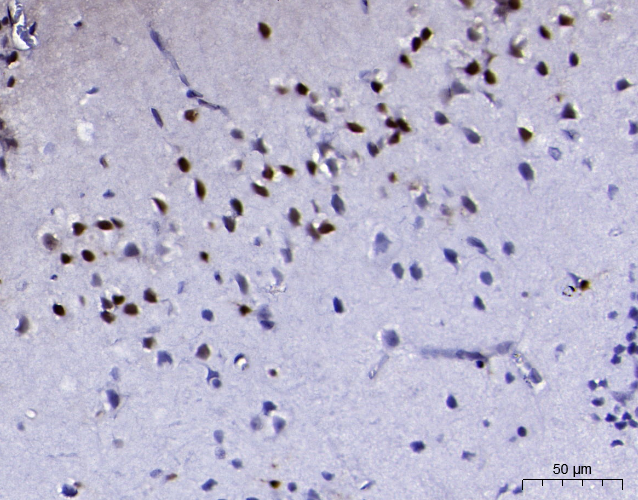

Supplement: Supplementary file 13 [file DataSheet10.ZIP › IHC Raw Image of p-CREB in striatum (3)/H34 1-200 PCREB_20.0x.tif-W4.tif]

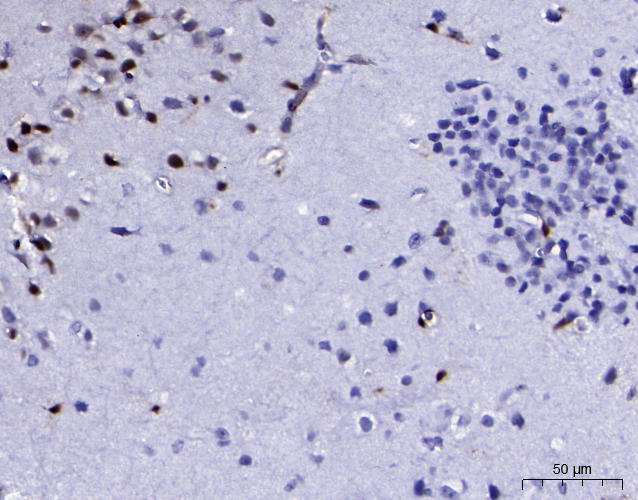

Supplement: Supplementary file 13 [file DataSheet10.ZIP › IHC Raw Image of p-CREB in striatum (3)/H34 1-200 PCREB_20.0x.tif-W5.tif]

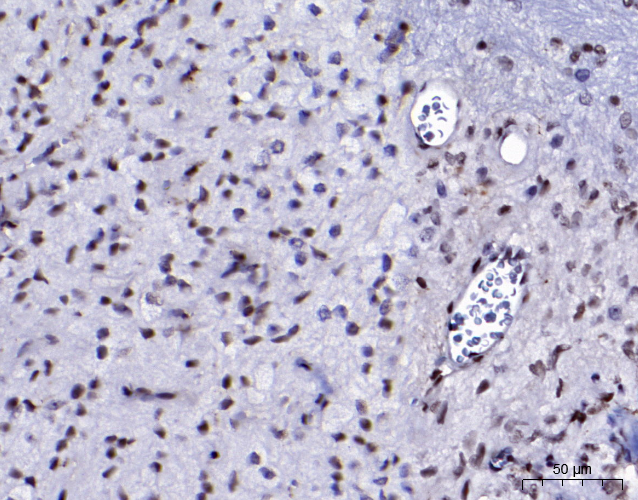

Supplement: Supplementary file 13 [file DataSheet10.ZIP › IHC Raw Image of p-CREB in striatum (3)/H41 1-200 PCREB_20.0x.tif-W1.tif]

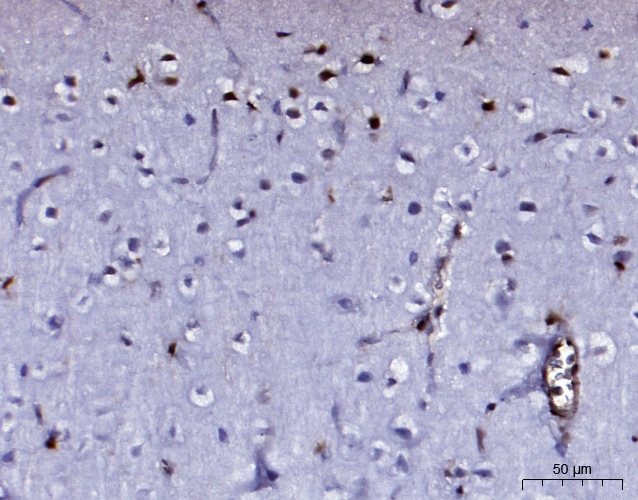

Supplement: Supplementary file 13 [file DataSheet10.ZIP › IHC Raw Image of p-CREB in striatum (3)/H41 1-200 PCREB_20.0x.tif-W2.tif]

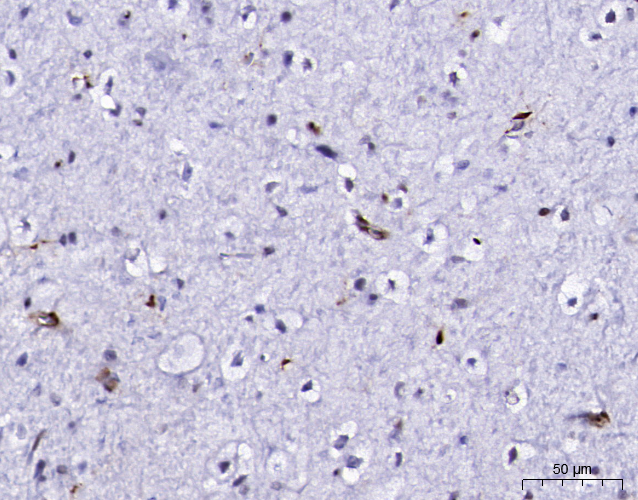

Supplement: Supplementary file 13 [file DataSheet10.ZIP › IHC Raw Image of p-CREB in striatum (3)/H41 1-200 PCREB_20.0x.tif-W3.tif]

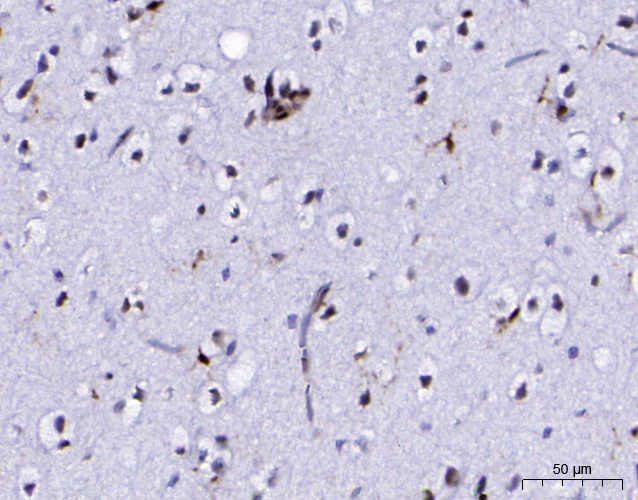

Supplement: Supplementary file 13 [file DataSheet10.ZIP › IHC Raw Image of p-CREB in striatum (3)/H41 1-200 PCREB_20.0x.tif-W4.tif]

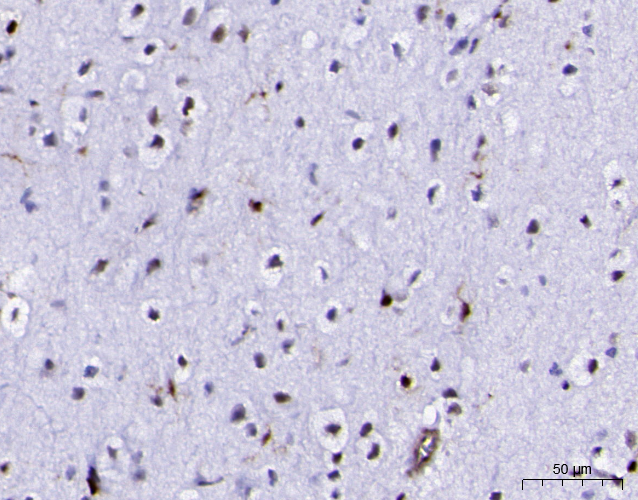

Supplement: Supplementary file 13 [file DataSheet10.ZIP › IHC Raw Image of p-CREB in striatum (3)/H41 1-200 PCREB_20.0x.tif-W5.tif]

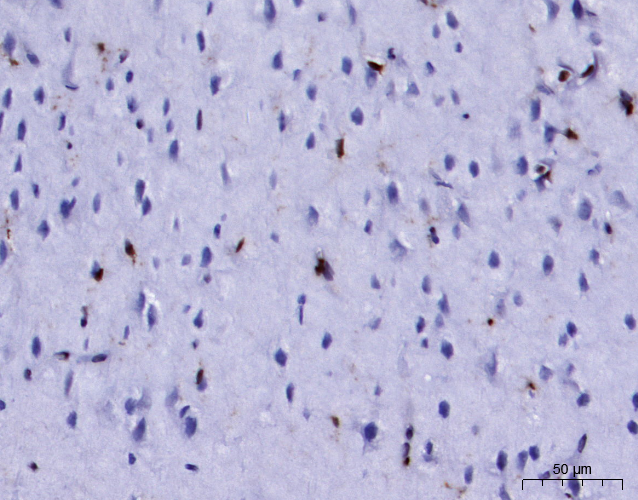

Supplement: Supplementary file 13 [file DataSheet10.ZIP › IHC Raw Image of p-CREB in striatum (3)/H48 1-200 PCREB_20.0x.tif-W1.tif]

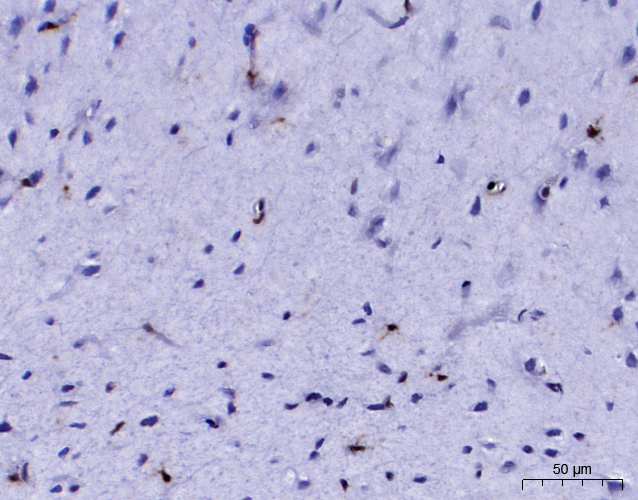

Supplement: Supplementary file 13 [file DataSheet10.ZIP › IHC Raw Image of p-CREB in striatum (3)/H48 1-200 PCREB_20.0x.tif-W2.tif]

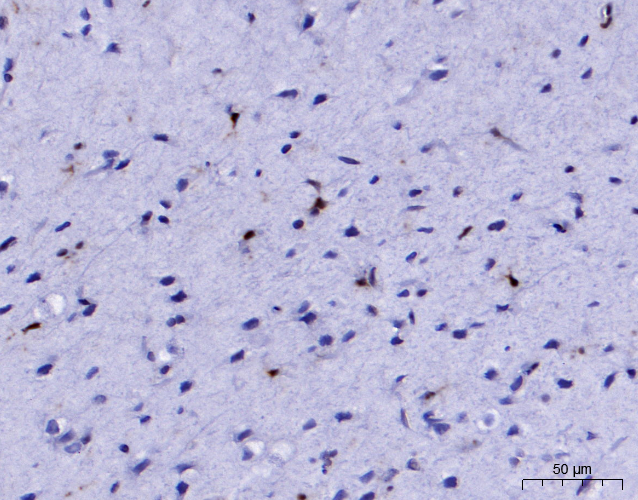

Supplement: Supplementary file 13 [file DataSheet10.ZIP › IHC Raw Image of p-CREB in striatum (3)/H48 1-200 PCREB_20.0x.tif-W3.tif]

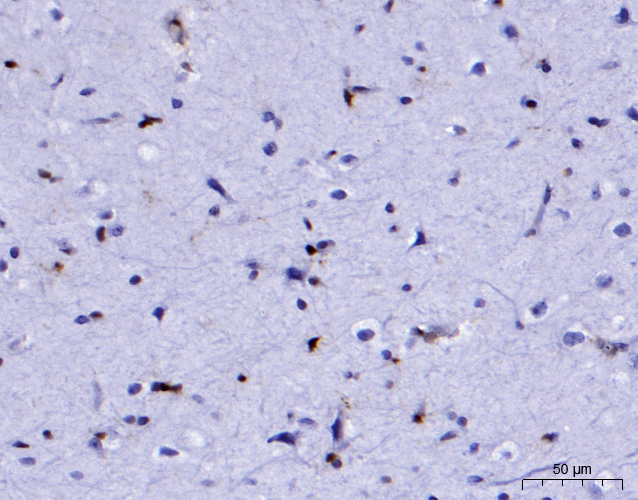

Supplement: Supplementary file 13 [file DataSheet10.ZIP › IHC Raw Image of p-CREB in striatum (3)/H48 1-200 PCREB_20.0x.tif-W4.tif]
