# Supplementary material for: Long Mu Qing Xin mixture improves behavioral performance in spontaneously hypertensive rats (SHR/NCrl) by upregulating catecholamine neurotransmitters in prefrontal cortex and striatum via DRD1/cAMP/PKA-CREB signaling pathway
Source: Front Pharmacol. 2024 Jul 4;15:1387359. doi: 10.3389/fphar.2024.1387359 (PMC11254830; doi:10.3389/fphar.2024.1387359)
Supplement: Supplementary file 8 [file Table1.pdf]

Supplementary Material

Long Mu Qing Xin Mixture improves behavioral performance in spontaneously hypertensive rats by upregulating catecholamine neurotransmitters in prefrontal cortex and Striatum via DRD1/cAMP/PKA-CREB signaling pathway

Xuejun Li, Zhen Xiao*, Zhiyan Jiang*, Wenyan Pu, Xiufeng Chen, Shumin Wang, Anqi Liu, Hongyu Zhang, Zihao Xu

*** Correspondence:** Zhen Xiao: xiaozhen@shutcm.edu.cn; Zhiyan Jiang: lhjzycm@163.com

1 Supplementary Tables

Supplementary Table 1 Qualitative analysis of LMQXM active ingredient.

Serial number	Chemical compound	Molecular formula	MS/MS	Origin
1	Gallic acid	$C_7H_6O_5$	169.0139、125.0244、97.0291、79.0191	Bai Shao
2	Adenosine	$C_{10}H_{13}N_5O_4$	268.1013、136.0616、119.0353	All
3	Cyclicadenosine monophosphate	$C_{10}H_{12}N_5O_6P$	330.0606、136.0615、119.0364	Da Zao
4	Hyemaloside B	$C_{26}H_{24}O_{13}$	543.1174、421.0799、259.0276、121.0297	Bai Shao
5	3-O-Feruloylquinic acid	$C_{17}H_{20}O_9$	367.1030、285.0773、193.0506、134.0376	Dang Gui
6	Phellodendrine	$C_{20}H_{24}NO_4^+$	342.1687、192.1014、177.0780、 148.0751	Huang Bai
7	Albiflorin	$C_{23}H_{28}O_{11}$	525.1596、479.1533、357.1179、283.0801、121.0295	Bai Shao
8	Magnoflorine	$C_{20}H_{24}NO_4^+$	342.1705、297.1126、265.0863、237.0905	Huang Bai 、Da Zao
9	Paeoniflorin	$C_{23}H_{28}O_{11}$	525.1613、479.1555、449.1444、327.1074、165.05556、121.0291	Bai Shao
10	Ferulic acid	$C_{10}H_{10}O_4$	178.0278、134.0377	Dang Gui
11	Liquiritin	$C_{21}H_{22}O_9$	417.1188、255.0659、135.0091、119.0503	Zhi Gan Cao

12	Chrysin 6-C-β-L-arabinoside-8-C-β-D-glucoside	$C_{26}H_{28}O_{13}$	547.1430、529.1329、487.1222、457.1118、367.0803、337.0697	Huang Qin
13	2,3,5,4'-Tetrahydroxystilbene 2-O-glucoside	$C_{20}H_{22}O_9$	405.1179、243.0665、225.0570、173.0614	Shou Wu Teng
14	Galloylpaeoniflorin	$C_{30}H_{32}O_{15}$	631.1667、465.1396、313.0551、169.0137	Bai Shao
15	Chrysin 6-C-β-D-glucoside-8-C-β-L-arabinoside	$C_{26}H_{28}O_{13}$	547.1433、457.1129、427.1018、367.0814、337.0709	Huang Qin
16	Mudanpioside I	$C_{23}H_{28}O_{11}$	525.1610、479.1548、357.1177、327.1063、283.0818、121.0292	Bai Shao
17	Isorhynchophylline	$C_{22}H_{28}N_2O_4$	385.2132、353.1871、241.1341、160.0762	Gou Teng
18	Lactiflorin	$C_{23}H_{26}O_{10}$	507.1502、461.1450、339.1073、177.0558、121.0302	Bai Shao
19	Baicalin	$C_{21}H_{18}O_{11}$	445.0777、269.0456、241.0510、223.0399、195.0447	Huang Qin
20	Rhynchophylline	$C_{22}H_{28}N_2O_4$	385.2117、353.1853、269.1639、215.1174、160.0758	Gou Teng
21	Liquiritigenin	$C_{15}H_{12}O_4$	255.0665、135.0097、119.0505	Zhi Gan Cao
22	Paeonol	$C_9H_{10}O_3$	165.0560、137.0247、93.0350、92.0273	Bai Shao
23	Norwogonin 7-O-glucuronide	$C_{21}H_{18}O_{11}$	445.0786、269.0433、225.0542、197.0597	Huang Qin
24	Berberine	$C_{20}H_{18}NO_4^+$	336.1232、320.0921、304.0976、292.0973、278.0819	Huang Bai
25	Calycosin	$C_{16}H_{12}O_5$	283.0594、268.0354、211.0383、184.0508、135.0075	Huang Qi
26	Oroxylin A 7-O-glucuronide	$C_{22}H_{20}O_{11}$	459.0921、283.0614、268.0370、175.0253、113.0249	Huang Qin
27	Emodin-8-glucoside	$C_{21}H_{20}O_{10}$	431.0954、269.0426、225.0543	Shou Wu Teng
28	Wogonin 7-O-glucuronide	$C_{22}H_{20}O_{11}$	459.0926、283.0609、268.0368、175.0256、113.0255	Huang Qin

29	Yohimbine	$C_{21}H_{26}N_2O_3$	355.2047、337.1968、224.1296、212.1282、144.0818	Gou Teng
30	Benzoylpaeoniflorin	$C_{30}H_{32}O_{12}$	629.1908、583.1853、553.1739、431.1354、121.0303	Bai Shao
31	Baicalein	$C_{15}H_{10}O_5$	269.0440、251.0338、241.0496、223.0385、195.0650、136.9882	Huang Qin
32	Formononetin	$C_{16}H_{12}O_4$	267.0666、252.0428、223.0406、195.0452、132.0224	Huang Qi
33	Licoricesaponin E2	$C_{42}H_{60}O_{16}$	819.3811、351.0555、193.0350	Zhi Gan Cao
34	Licoricesaponin G2	$C_{42}H_{62}O_{17}$	837.3916、351.0560、193.0352、175.0244	Zhi Gan Cao
35	Astragaloside IV	$C_{41}H_{68}O_{14}$	829.4543、783.4487、621.4047、489.3607、445.0764	Huang Qi
36	Obaculactone	$C_{26}H_{30}O_8$	515.1954、469.1886、411.1451、381.2093、306.1264、229.1238	Huang Bai
37	Glycyrrhizic acid	$C_{42}H_{62}O_{16}$	821.3947、351.0557、193.0350、175.0244	Zhi Gan Cao
38	Schisandrol A	$C_{24}H_{32}O_7$	415.2124、400.1894、384.1938、369.1700	Wu Wei Zi
39	(3Z,5aS,6S,9S,9aS)-3-Butylidene-1,3,4,5,5a,6,9,9a-octahydro-1-oxo-9-(1-oxopentyl)-6,9-ethanonaphtho[1,2-c]furan-8-carboxylic acid	$C_{24}H_{30}O_5$	397.2015、353.2116、207.1032、189.0923、163.1131	Dang Gui
40	Schisandrin A	$C_{24}H_{32}O_6$	417.2298、402.2064、347.1493、316.1316、301.1076	Wu Wei Zi

Supplementary Table 2 Effect of LMQXM on total moving distance and average speed in rats.（$\bar{x} \pm s$，n=12）

Group	Total moving distance (cm)		Average speed (cm/s)	
	Pre-administration	Post- administration	Pre-administration	Post- administration
WKY	591.37±207.15	983.87±510.36	1.97±0.69	3.28±1.70
SHR	3527.92±645.60 $^{###}$	3397.31±580.59 $^{###}$	11.76±2.15 $^{###}$	11.33±1.94 $^{###}$
MPH	3052.31±622.54	2070.23±552.07 $^{***,+}$	10.18±2.08	6.89±1.82 $^{***,+}$
LMQXM-LD	3288.92±725.18	2348.21±451.73 $^{**,+}$	10.96±2.42	7.85±1.50 $^{**,+}$

LMQXM-MD	2743.40±1091.42	1578.85±559.46 ***, +++	9.14±3.64	5.28±1.86 ***, +++
LMQXM-HD	3312.78±696.79	2077.91±509.28 ***, +++	11.05±2.32	6.93±1.70 ***, +++

Note: Compared with the WKY group, ###$P < 0.001$; compared with the SHR group, **$P < 0.01$, ***$P < 0.001$. compared with the same group before treatment, +$P < 0.05$, ++$P < 0.01$, +++$P < 0.001$.

Supplementary Table 3 Effect of LMQXM on the number of uprights and time spent in the central area in rats. （$\bar{x} \pm s$，n=12）

Group	The number of uprights (n)		Time spent in the central area (s)	
	Pre-administration	Post- administration	Pre-administration	Post- administration
WKY	3.50±1.51	2.08±1.00	93.95±125.76	68.42±101.26
SHR	39.00±10.96 ###	30.17±9.51 ###	48.77±21.51	110.02±23.24
MPH	29.67±10.00	8.83±5.44 ***, +++	41.35±20.38	94.53±41.84
LMQXM-LD	34.08±10.71	14.17±7.94 ***, +++	47.83±18.25	93.484±38.23
LMQXM-MD	35.33±15.15	8.58±3.80 ***, +++	47.19±31.53	42.19±29.36
LMQXM-HD	30.25±7.56	9.427±4.54 ***, +++	47.83±14.35	65.573±35.89

Note: Compared with the WKY group, ###$P < 0.001$; compared with the SHR group, ***$P < 0.001$. compared with the same group before treatment, +++$P < 0.001$.

Supplementary Table 4 Two- ANOVA of LMQXM on total moving distance in rats.

Table Analyzed	Total moving distance				
Two-way ANOVA	Ordinary				
Alpha	0.05				
Source of Variation	% of total variation	P value	P value summary	Significant?	
Interaction	7.213	<0.0001	****	Yes	
Row Factor	54.56	<0.0001	****	Yes	
Column Factor	9.201	<0.0001	****	Yes	
ANOVA table	SS	DF	MS	F (DFn, DFd)	P value
Interaction	12923476	5	2584695	F (5, 132) = 6.559	P<0.0001
Row Factor	97756219	5	19551244	F (5, 132) = 49.62	P<0.0001
Column Factor	16486192	1	16486192	F (1, 132) = 41.84	P<0.0001
Residual	52014762	132	394051		
Difference between column means					
Mean of 0 week	2753				
Mean of 4 week	2076				
Difference between means	676.7				
SE of difference	104.6				
95% CI of difference	469.8 to 883.7				

Supplementary Table 5 Multiple of comparisons of total moving distance in rats.

Compare cell means regardless of rows and columns					

Number of families	1				
Number of comparisons per family	66				
Alpha	0.05				
Bonferroni's multiple comparisons test	Mean Diff.	95.00% CI of diff.	Significant?	Summary	Adjusted P Value
WKY:0 week vs. WKY:4 week	-392.5	-1276 to 491.2	No	ns	>0.9999
WKY:0 week vs. SHR:0 week	-2937	-3820 to -2053	Yes	****	<0.0001
WKY:0 week vs. SHR:4 week	-2806	-3690 to -1922	Yes	****	<0.0001
WKY:0 week vs. MPH:0 week	-2461	-3345 to -1577	Yes	****	<0.0001
WKY:0 week vs. MPH:4 week	-1479	-2363 to -595.2	Yes	****	<0.0001
WKY:0 week vs. LMQXM-LD:0 week	-2698	-3581 to -1814	Yes	****	<0.0001
WKY:0 week vs. LMQXM-LD:4 week	-1757	-2641 to -873.1	Yes	****	<0.0001
WKY:0 week vs. LMQXM-MD:0 week	-2152	-3036 to -1268	Yes	****	<0.0001
WKY:0 week vs. LMQXM-MD:4 week	-987.5	-1871 to -103.8	Yes	*	0.0120
WKY:0 week vs. LMQXM-HD:0 week	-2721	-3605 to -1838	Yes	****	<0.0001
WKY:0 week vs. LMQXM-HD:4 week	-1487	-2370 to -602.8	Yes	****	<0.0001
WKY:4 week vs. SHR:0 week	-2544	-3428 to -1660	Yes	****	<0.0001
WKY:4 week vs. SHR:4 week	-2413	-3297 to -1530	Yes	****	<0.0001
WKY:4 week vs. MPH:0 week	-2068	-2952 to -1185	Yes	****	<0.0001
WKY:4 week vs. MPH:4 week	-1086	-1970 to -202.7	Yes	**	0.0028
WKY:4 week vs. LMQXM-LD:0 week	-2305	-3189 to -1421	Yes	****	<0.0001
WKY:4 week vs. LMQXM-LD:4 week	-1364	-2248 to -480.7	Yes	****	<0.0001
WKY:4 week vs. LMQXM-MD:0 week	-1760	-2643 to -875.8	Yes	****	<0.0001
WKY:4 week vs. LMQXM-MD:4 week	-595.0	-1479 to 288.7	No	ns	>0.9999
WKY:4 week vs. LMQXM-HD:0 week	-2329	-3213 to -1445	Yes	****	<0.0001
WKY:4 week vs. LMQXM-HD:4 week	-1094	-1978 to -210.3	Yes	**	0.0025
SHR:0 week vs. SHR:4 week	130.6	-753.1 to 1014	No	ns	>0.9999
SHR:0 week vs. MPH:0 week	475.6	-408.1 to 1359	No	ns	>0.9999
SHR:0 week vs. MPH:4 week	1458	574.0 to 2341	Yes	****	<0.0001
SHR:0 week vs. LMQXM-LD:0 week	239.0	-644.7 to 1123	No	ns	>0.9999
SHR:0 week vs. LMQXM-LD:4 week	1180	296.0 to 2063	Yes	***	0.0006
SHR:0 week vs. LMQXM-MD:0 week	784.5	-99.17 to 1668	No	ns	0.1763
SHR:0 week vs. LMQXM-MD:4 week	1949	1065 to 2833	Yes	****	<0.0001
SHR:0 week vs. LMQXM-HD:0 week	215.1	-668.6 to 1099	No	ns	>0.9999
SHR:0 week vs. LMQXM-HD:4 week	1450	566.3 to 2334	Yes	****	<0.0001
SHR:4 week vs. MPH:0 week	345.0	-538.7 to 1229	No	ns	>0.9999
SHR:4 week vs. MPH:4 week	1327	443.4 to 2211	Yes	****	<0.0001
SHR:4 week vs. LMQXM-LD:0 week	108.4	-775.3 to 992.1	No	ns	>0.9999
SHR:4 week vs. LMQXM-LD:4 week	1049	165.4 to 1933	Yes	**	0.0049
SHR:4 week vs. LMQXM-MD:0 week	653.9	-229.8 to 1538	No	ns	0.7828
SHR:4 week vs. LMQXM-MD:4 week	1818	934.8 to 2702	Yes	****	<0.0001
SHR:4 week vs. LMQXM-HD:0 week	84.53	-799.2 to 968.2	No	ns	>0.9999
SHR:4 week vs. LMQXM-HD:4 week	1319	435.7 to 2203	Yes	****	<0.0001
MPH:0 week vs. MPH:4 week	982.1	98.39 to 1866	Yes	*	0.0129
MPH:0 week vs. LMQXM-LD:0 week	-236.6	-1120 to 647.1	No	ns	>0.9999
MPH:0 week vs. LMQXM-LD:4 week	704.1	-179.6 to 1588	No	ns	0.4518
MPH:0 week vs. LMQXM-MD:0 week	308.9	-574.8 to 1193	No	ns	>0.9999
MPH:0 week vs. LMQXM-MD:4 week	1473	589.8 to 2357	Yes	****	<0.0001
MPH:0 week vs. LMQXM-HD:0 week	-260.5	-1144 to 623.2	No	ns	>0.9999
MPH:0 week vs. LMQXM-HD:4 week	974.4	90.71 to 1858	Yes	*	0.0144
MPH:4 week vs. LMQXM-LD:0 week	-1219	-2102 to -335.0	Yes	***	0.0003
MPH:4 week vs. LMQXM-LD:4 week	-278.0	-1162 to 605.7	No	ns	>0.9999
MPH:4 week vs. LMQXM-MD:0 week	-673.2	-1557 to 210.5	No	ns	0.6362
MPH:4 week vs. LMQXM-MD:4 week	491.4	-392.3 to 1375	No	ns	>0.9999
MPH:4 week vs. LMQXM-HD:0 week	-1243	-2126 to -358.9	Yes	***	0.0002
MPH:4 week vs. LMQXM-HD:4 week	-7.678	-891.4 to 876.0	No	ns	>0.9999
LMQXM-LD:0 week vs. LMQXM-LD:4 week	940.7	57.01 to 1824	Yes	*	0.0231

LMQXM-LD:0 week vs. LMQXM-MD:0 week	545.5	-338.2 to 1429	No	ns	>0.9999
LMQXM-LD:0 week vs. LMQXM-MD:4 week	1710	826.4 to 2594	Yes	****	<0.0001
LMQXM-LD:0 week vs. LMQXM-HD:0 week	-23.86	-907.6 to 859.8	No	ns	>0.9999
LMQXM-LD:0 week vs. LMQXM-HD:4 week	1211	327.3 to 2095	Yes	***	0.0004
LMQXM-LD:4 week vs. LMQXM-MD:0 week	-395.2	-1279 to 488.5	No	ns	>0.9999
LMQXM-LD:4 week vs. LMQXM-MD:4 week	769.4	-114.3 to 1653	No	ns	0.2117
LMQXM-LD:4 week vs. LMQXM-HD:0 week	-964.6	-1848 to -80.88	Yes	*	0.0166
LMQXM-LD:4 week vs. LMQXM-HD:4 week	270.3	-613.4 to 1154	No	ns	>0.9999
LMQXM-MD:0 week vs. LMQXM-MD:4 week	1165	280.9 to 2048	Yes	***	0.0008
LMQXM-MD:0 week vs. LMQXM-HD:0 week	-569.4	-1453 to 314.3	No	ns	>0.9999
LMQXM-MD:0 week vs. LMQXM-HD:4 week	665.5	-218.2 to 1549	No	ns	0.6914
LMQXM-MD:4 week vs. LMQXM-HD:0 week	-1734	-2618 to -850.2	Yes	****	<0.0001
LMQXM-MD:4 week vs. LMQXM-HD:4 week	-499.1	-1383 to 384.6	No	ns	>0.9999
LMQXM-HD:0 week vs. LMQXM-HD:4 week	1235	351.2 to 2119	Yes	***	0.0003

Supplementary Table 6 Two- ANOVA of LMQXM on average speed in rats.

Table Analyzed	Average speed				
Two-way ANOVA	Ordinary				
Alpha	0.05				
Source of Variation	% of total variation	P value	P value summary	Significant?	
Interaction	7.215	<0.0001	****	Yes	
Row Factor	54.61	<0.0001	****	Yes	
Column Factor	9.164	<0.0001	****	Yes	
ANOVA table	SS	DF	MS	F (DFn, DFd)	P value
Interaction	143.6	5	28.71	F (5, 132) = 6.565	P<0.0001
Row Factor	1087	5	217.3	F (5, 132) = 49.69	P<0.0001
Column Factor	182.3	1	182.3	F (1, 132) = 41.70	P<0.0001
Residual	577.2	132	4.373		
Difference between column means					
Mean of 0 week	9.177				
Mean of 4 week	6.926				
Difference between means	2.251				
SE of difference	0.3485				
95% CI of difference	1.561 to 2.940				

Supplementary Table 7 Multiple of comparisons of average speed in rats.

Compare cell means regardless of rows and columns					
Number of families	1				
Number of comparisons per family	66				
Alpha	0.05				
Bonferroni's multiple comparisons test	Mean Diff.	95.00% CI of diff.	Significant?	Summary	Adjusted P Value
WKY:0 week vs. WKY:4 week	-1.314	-4.258 to 1.630	No	ns	>0.9999
WKY:0 week vs. SHR:0 week	-9.790	-12.73 to -6.846	Yes	****	<0.0001
WKY:0 week vs. SHR:4 week	-9.356	-12.30 to -6.412	Yes	****	<0.0001
WKY:0 week vs. MPH:0 week	-8.206	-11.15 to -5.262	Yes	****	<0.0001
WKY:0 week vs. MPH:4 week	-4.918	-7.862 to -1.974	Yes	****	<0.0001
WKY:0 week vs. LMQXM-LD:0 week	-8.993	-11.94 to -6.049	Yes	****	<0.0001
WKY:0 week vs. LMQXM-LD:4 week	-5.884	-8.828 to -2.940	Yes	****	<0.0001
WKY:0 week vs. LMQXM-MD:0 week	-7.174	-10.12 to -4.230	Yes	****	<0.0001
WKY:0 week vs. LMQXM-MD:4 week	-3.308	-6.251 to -0.3636	Yes	*	0.0111

WKY:0 week vs. LMQXM-HD:0 week	-9.078	-12.02 to -6.134	Yes	****	<0.0001
WKY:0 week vs. LMQXM-HD:4 week	-4.958	-7.902 to -2.014	Yes	****	<0.0001
WKY:4 week vs. SHR:0 week	-8.476	-11.42 to -5.532	Yes	****	<0.0001
WKY:4 week vs. SHR:4 week	-8.042	-10.99 to -5.098	Yes	****	<0.0001
WKY:4 week vs. MPH:0 week	-6.892	-9.836 to -3.948	Yes	****	<0.0001
WKY:4 week vs. MPH:4 week	-3.604	-6.548 to -0.6603	Yes	**	0.0030
WKY:4 week vs. LMQXM-LD:0 week	-7.679	-10.62 to -4.735	Yes	****	<0.0001
WKY:4 week vs. LMQXM-LD:4 week	-4.570	-7.514 to -1.626	Yes	****	<0.0001
WKY:4 week vs. LMQXM-MD:0 week	-5.860	-8.804 to -2.916	Yes	****	<0.0001
WKY:4 week vs. LMQXM-MD:4 week	-1.993	-4.937 to 0.9505	No	ns	>0.9999
WKY:4 week vs. LMQXM-HD:0 week	-7.764	-10.71 to -4.820	Yes	****	<0.0001
WKY:4 week vs. LMQXM-HD:4 week	-3.644	-6.588 to -0.7003	Yes	**	0.0025
SHR:0 week vs. SHR:4 week	0.4342	-2.510 to 3.378	No	ns	>0.9999
SHR:0 week vs. MPH:0 week	1.584	-1.360 to 4.528	No	ns	>0.9999
SHR:0 week vs. MPH:4 week	4.872	1.928 to 7.816	Yes	****	<0.0001
SHR:0 week vs. LMQXM-LD:0 week	0.7967	-2.147 to 3.741	No	ns	>0.9999
SHR:0 week vs. LMQXM-LD:4 week	3.906	0.9620 to 6.850	Yes	***	0.0007
SHR:0 week vs. LMQXM-MD:0 week	2.616	-0.3280 to 5.560	No	ns	0.1748
SHR:0 week vs. LMQXM-MD:4 week	6.482	3.539 to 9.426	Yes	****	<0.0001
SHR:0 week vs. LMQXM-HD:0 week	0.7117	-2.232 to 3.656	No	ns	>0.9999
SHR:0 week vs. LMQXM-HD:4 week	4.832	1.888 to 7.776	Yes	****	<0.0001
SHR:4 week vs. MPH:0 week	1.150	-1.794 to 4.094	No	ns	>0.9999
SHR:4 week vs. MPH:4 week	4.437	1.494 to 7.381	Yes	****	<0.0001
SHR:4 week vs. LMQXM-LD:0 week	0.3625	-2.581 to 3.306	No	ns	>0.9999
SHR:4 week vs. LMQXM-LD:4 week	3.472	0.5278 to 6.416	Yes	**	0.0054
SHR:4 week vs. LMQXM-MD:0 week	2.182	-0.7622 to 5.126	No	ns	0.7747
SHR:4 week vs. LMQXM-MD:4 week	6.048	3.104 to 8.992	Yes	****	<0.0001
SHR:4 week vs. LMQXM-HD:0 week	0.2775	-2.666 to 3.221	No	ns	>0.9999
SHR:4 week vs. LMQXM-HD:4 week	4.398	1.454 to 7.341	Yes	****	<0.0001
MPH:0 week vs. MPH:4 week	3.288	0.3436 to 6.231	Yes	*	0.0121
MPH:0 week vs. LMQXM-LD:0 week	-0.7875	-3.731 to 2.156	No	ns	>0.9999
MPH:0 week vs. LMQXM-LD:4 week	2.322	-0.6222 to 5.266	No	ns	0.4897
MPH:0 week vs. LMQXM-MD:0 week	1.032	-1.912 to 3.976	No	ns	>0.9999
MPH:0 week vs. LMQXM-MD:4 week	4.898	1.954 to 7.842	Yes	****	<0.0001
MPH:0 week vs. LMQXM-HD:0 week	-0.8725	-3.816 to 2.071	No	ns	>0.9999
MPH:0 week vs. LMQXM-HD:4 week	3.248	0.3036 to 6.191	Yes	*	0.0143
MPH:4 week vs. LMQXM-LD:0 week	-4.075	-7.019 to -1.131	Yes	***	0.0003
MPH:4 week vs. LMQXM-LD:4 week	-0.9658	-3.910 to 1.978	No	ns	>0.9999
MPH:4 week vs. LMQXM-MD:0 week	-2.256	-5.200 to 0.6880	No	ns	0.6091
MPH:4 week vs. LMQXM-MD:4 week	1.611	-1.333 to 4.555	No	ns	>0.9999
MPH:4 week vs. LMQXM-HD:0 week	-4.160	-7.104 to -1.216	Yes	***	0.0002
MPH:4 week vs. LMQXM-HD:4 week	-0.04000	-2.984 to 2.904	No	ns	>0.9999
LMQXM-LD:0 week vs. LMQXM-LD:4 week	3.109	0.1653 to 6.053	Yes	*	0.0256
LMQXM-LD:0 week vs. LMQXM-MD:0 week	1.819	-1.125 to 4.763	No	ns	>0.9999
LMQXM-LD:0 week vs. LMQXM-MD:4 week	5.686	2.742 to 8.630	Yes	****	<0.0001
LMQXM-LD:0 week vs. LMQXM-HD:0 week	-0.08500	-3.029 to 2.859	No	ns	>0.9999
LMQXM-LD:0 week vs. LMQXM-HD:4 week	4.035	1.091 to 6.979	Yes	***	0.0004
LMQXM-LD:4 week vs. LMQXM-MD:0 week	-1.290	-4.234 to 1.654	No	ns	>0.9999
LMQXM-LD:4 week vs. LMQXM-MD:4 week	2.577	-0.3672 to 5.521	No	ns	0.2015
LMQXM-LD:4 week vs. LMQXM-HD:0 week	-3.194	-6.138 to -0.2503	Yes	*	0.0179
LMQXM-LD:4 week vs. LMQXM-HD:4 week	0.9258	-2.018 to 3.870	No	ns	>0.9999
LMQXM-MD:0 week vs. LMQXM-MD:4 week	3.867	0.9228 to 6.811	Yes	***	0.0009
LMQXM-MD:0 week vs. LMQXM-HD:0 week	-1.904	-4.848 to 1.040	No	ns	>0.9999
LMQXM-MD:0 week vs. LMQXM-HD:4 week	2.216	-0.7280 to 5.160	No	ns	0.6940
LMQXM-MD:4 week vs. LMQXM-HD:0 week	-5.771	-8.715 to -2.827	Yes	****	<0.0001

LMQXM-MD:4 week vs. LMQXM-HD:4 week	-1.651	-4.595 to 1.293	No	ns	>0.9999
LMQXM-HD:0 week vs. LMQXM-HD:4 week	4.120	1.176 to 7.064	Yes	***	0.0003

Supplementary Table 8 Two- ANOVA of LMQXM on the number of uprights in rats.

Table Analyzed	The number of uprights				
Two-way ANOVA	Ordinary				
Alpha	0.05				
Source of Variation	% of total variation	P value	P value summary	Significant?	
Interaction	7.698	<0.0001	****	Yes	
Time	36.99	<0.0001	****	Yes	
Column Factor	28.31	<0.0001	****	Yes	
ANOVA table	SS	DF	MS	F (DFn, DFd)	P value
Interaction	2643	5	528.7	F (5, 132) = 7.525	P<0.0001
Time	12700	5	2540	F (5, 132) = 36.16	P<0.0001
Column Factor	9719	1	9719	F (1, 132) = 138.3	P<0.0001
Residual	9273	132	70.25		
Difference between column means					
Mean of 0 week	28.64				
Mean of 4 week	12.21				
Difference between means	16.43				
SE of difference	1.397				
95% CI of difference	13.67 to 19.19				

Supplementary Table 9 Multiple of comparisons of the number of uprights in rats.

Compare cell means regardless of rows and columns					
Number of families	1				
Number of comparisons per family	66				
Alpha	0.05				
Tukey's multiple comparisons test	Mean Diff.	95.00% CI of diff.	Significant?	Summary	Adjusted P Value
WKY:0 week vs. WKY:4 week	1.417	-9.970 to 12.80	No	ns	>0.9999
WKY:0 week vs. SHR:0 week	-35.50	-46.89 to -24.11	Yes	****	<0.0001
WKY:0 week vs. SHR:4 week	-26.67	-38.05 to -15.28	Yes	****	<0.0001
WKY:0 week vs. MPH:0 week	-26.17	-37.55 to -14.78	Yes	****	<0.0001
WKY:0 week vs. MPH:4 week	-5.333	-16.72 to 6.053	No	ns	0.9205
WKY:0 week vs. LMQXM-LD:0 week	-30.58	-41.97 to -19.20	Yes	****	<0.0001
WKY:0 week vs. LMQXM-LD:4 week	-10.67	-22.05 to 0.7196	No	ns	0.0892
WKY:0 week vs. LMQXM-MD:0 week	-31.83	-43.22 to -20.45	Yes	****	<0.0001
WKY:0 week vs. LMQXM-MD:4 week	-5.083	-16.47 to 6.303	No	ns	0.9420
WKY:0 week vs. LMQXM-HD:0 week	-26.75	-38.14 to -15.36	Yes	****	<0.0001
WKY:0 week vs. LMQXM-HD:4 week	-5.917	-17.30 to 5.470	No	ns	0.8515
WKY:4 week vs. SHR:0 week	-36.92	-48.30 to -25.53	Yes	****	<0.0001
WKY:4 week vs. SHR:4 week	-28.08	-39.47 to -16.70	Yes	****	<0.0001
WKY:4 week vs. MPH:0 week	-27.58	-38.97 to -16.20	Yes	****	<0.0001
WKY:4 week vs. MPH:4 week	-6.750	-18.14 to 4.636	No	ns	0.7108
WKY:4 week vs. LMQXM-LD:0 week	-32.00	-43.39 to -20.61	Yes	****	<0.0001
WKY:4 week vs. LMQXM-LD:4 week	-12.08	-23.47 to -0.6970	Yes	*	0.0272

WKY:4 week vs. LMQXM-MD:0 week	-33.25	-44.64 to -21.86	Yes	****	<0.0001
WKY:4 week vs. LMQXM-MD:4 week	-6.500	-17.89 to 4.886	No	ns	0.7574
WKY:4 week vs. LMQXM-HD:0 week	-28.17	-39.55 to -16.78	Yes	****	<0.0001
WKY:4 week vs. LMQXM-HD:4 week	-7.333	-18.72 to 4.053	No	ns	0.5932
SHR:0 week vs. SHR:4 week	8.833	-2.553 to 20.22	No	ns	0.3010
SHR:0 week vs. MPH:0 week	9.333	-2.053 to 20.72	No	ns	0.2248
SHR:0 week vs. MPH:4 week	30.17	18.78 to 41.55	Yes	****	<0.0001
SHR:0 week vs. LMQXM-LD:0 week	4.917	-6.470 to 16.30	No	ns	0.9539
SHR:0 week vs. LMQXM-LD:4 week	24.83	13.45 to 36.22	Yes	****	<0.0001
SHR:0 week vs. LMQXM-MD:0 week	3.667	-7.720 to 15.05	No	ns	0.9954
SHR:0 week vs. LMQXM-MD:4 week	30.42	19.03 to 41.80	Yes	****	<0.0001
SHR:0 week vs. LMQXM-HD:0 week	8.750	-2.636 to 20.14	No	ns	0.3150
SHR:0 week vs. LMQXM-HD:4 week	29.58	18.20 to 40.97	Yes	****	<0.0001
SHR:4 week vs. MPH:0 week	0.5000	-10.89 to 11.89	No	ns	>0.9999
SHR:4 week vs. MPH:4 week	21.33	9.947 to 32.72	Yes	****	<0.0001
SHR:4 week vs. LMQXM-LD:0 week	-3.917	-15.30 to 7.470	No	ns	0.9919
SHR:4 week vs. LMQXM-LD:4 week	16.00	4.614 to 27.39	Yes	***	0.0004
SHR:4 week vs. LMQXM-MD:0 week	-5.167	-16.55 to 6.220	No	ns	0.9354
SHR:4 week vs. LMQXM-MD:4 week	21.58	10.20 to 32.97	Yes	****	<0.0001
SHR:4 week vs. LMQXM-HD:0 week	-0.08333	-11.47 to 11.30	No	ns	>0.9999
SHR:4 week vs. LMQXM-HD:4 week	20.75	9.364 to 32.14	Yes	****	<0.0001
MPH:0 week vs. MPH:4 week	20.83	9.447 to 32.22	Yes	****	<0.0001
MPH:0 week vs. LMQXM-LD:0 week	-4.417	-15.80 to 6.970	No	ns	0.9790
MPH:0 week vs. LMQXM-LD:4 week	15.50	4.114 to 26.89	Yes	***	0.0008
MPH:0 week vs. LMQXM-MD:0 week	-5.667	-17.05 to 5.720	No	ns	0.8843
MPH:0 week vs. LMQXM-MD:4 week	21.08	9.697 to 32.47	Yes	****	<0.0001
MPH:0 week vs. LMQXM-HD:0 week	-0.5833	-11.97 to 10.80	No	ns	>0.9999
MPH:0 week vs. LMQXM-HD:4 week	20.25	8.864 to 31.64	Yes	****	<0.0001
MPH:4 week vs. LMQXM-LD:0 week	-25.25	-36.64 to -13.86	Yes	****	<0.0001
MPH:4 week vs. LMQXM-LD:4 week	-5.333	-16.72 to 6.053	No	ns	0.9205
MPH:4 week vs. LMQXM-MD:0 week	-26.50	-37.89 to -15.11	Yes	****	<0.0001
MPH:4 week vs. LMQXM-MD:4 week	0.2500	-11.14 to 11.64	No	ns	>0.9999
MPH:4 week vs. LMQXM-HD:0 week	-21.42	-32.80 to -10.03	Yes	****	<0.0001
MPH:4 week vs. LMQXM-HD:4 week	-0.5833	-11.97 to 10.80	No	ns	>0.9999
LMQXM-LD:0 week vs. LMQXM-LD:4 week	19.92	8.530 to 31.30	Yes	****	<0.0001
LMQXM-LD:0 week vs. LMQXM-MD:0 week	-1.250	-12.64 to 10.14	No	ns	>0.9999
LMQXM-LD:0 week vs. LMQXM-MD:4 week	25.50	14.11 to 36.89	Yes	****	<0.0001
LMQXM-LD:0 week vs. LMQXM-HD:0 week	3.833	-7.553 to 15.22	No	ns	0.9933
LMQXM-LD:0 week vs. LMQXM-HD:4 week	24.67	13.28 to 36.05	Yes	****	<0.0001
LMQXM-LD:4 week vs. LMQXM-MD:0 week	-21.17	-32.55 to -9.780	Yes	****	<0.0001
LMQXM-LD:4 week vs. LMQXM-MD:4 week	5.583	-5.803 to 16.97	No	ns	0.8942
LMQXM-LD:4 week vs. LMQXM-HD:0 week	-16.08	-27.47 to -4.697	Yes	***	0.0004
LMQXM-LD:4 week vs. LMQXM-HD:4 week	4.750	-6.636 to 16.14	No	ns	0.9639
LMQXM-MD:0 week vs. LMQXM-MD:4 week	26.75	15.36 to 38.14	Yes	****	<0.0001
LMQXM-MD:0 week vs. LMQXM-HD:0 week	5.083	-6.303 to 16.47	No	ns	0.9420
LMQXM-MD:0 week vs. LMQXM-HD:4 week	25.92	14.53 to 37.30	Yes	****	<0.0001
LMQXM-MD:4 week vs. LMQXM-HD:0 week	-21.67	-33.05 to -10.28	Yes	****	<0.0001
LMQXM-MD:4 week vs. LMQXM-HD:4 week	-0.8333	-12.22 to 10.55	No	ns	>0.9999
LMQXM-HD:0 week vs. LMQXM-HD:4 week	20.83	9.447 to 32.22	Yes	****	<0.0001

Supplementary Table 10 Two- ANOVA of LMQXM on time spent in the central area in rats.

Table Analyzed	Time spent in the central area				
Two-way ANOVA	Ordinary				
Alpha	0.05				
Source of Variation	% of total variation	P value	P value summary	Significant?	
Interaction	7.887	0.0320	*	Yes	
Row Factor	5.079	0.1567	ns	No	
Column Factor	4.722	0.0068	**	Yes	
ANOVA table	SS	DF	MS	F (DFn, DFd)	P value
Interaction	36239	5	7248	F (5, 132) = 2.530	P=0.0320
Row Factor	23337	5	4667	F (5, 132) = 1.629	P=0.1567
Column Factor	21695	1	21695	F (1, 132) = 7.572	P=0.0068
Residual	378212	132	2865		
Difference between column means					
Mean of 0 week	54.49				
Mean of 4 week	79.04				
Difference between means	-24.55				
SE of difference	8.921				
95% CI of difference	-42.20 to -6.901				

Supplementary Table 11 Multiple of comparisons of time spent in the central area in rats.

Compare cell means regardless of rows and columns					
Number of families	1				
Number of comparisons per family	66				
Alpha	0.05				
Bonferroni's multiple comparisons test	Mean Diff.	95.00% CI of diff.	Significant?	Summary	Adjusted P Value
WKY:0 week vs. WKY:4 week	25.53	-49.82 to 100.9	No	ns	>0.9999
WKY:0 week vs. SHR:0 week	45.18	-30.18 to 120.5	No	ns	>0.9999
WKY:0 week vs. SHR:4 week	-16.08	-91.43 to 59.28	No	ns	>0.9999
WKY:0 week vs. MPH:0 week	52.60	-22.76 to 128.0	No	ns	>0.9999
WKY:0 week vs. MPH:4 week	-0.5800	-75.93 to 74.77	No	ns	>0.9999
WKY:0 week vs. LMQXM-LD:0 week	46.12	-29.24 to 121.5	No	ns	>0.9999
WKY:0 week vs. LMQXM-LD:4 week	0.4642	-74.89 to 75.82	No	ns	>0.9999
WKY:0 week vs. LMQXM-MD:0 week	46.76	-28.60 to 122.1	No	ns	>0.9999
WKY:0 week vs. LMQXM-MD:4 week	51.76	-23.60 to 127.1	No	ns	>0.9999
WKY:0 week vs. LMQXM-HD:0 week	46.12	-29.24 to 121.5	No	ns	>0.9999
WKY:0 week vs. LMQXM-HD:4 week	28.38	-46.98 to 103.7	No	ns	>0.9999
WKY:4 week vs. SHR:0 week	19.64	-55.71 to 95.00	No	ns	>0.9999
WKY:4 week vs. SHR:4 week	-41.61	-117.0 to 33.75	No	ns	>0.9999
WKY:4 week vs. MPH:0 week	27.06	-48.29 to 102.4	No	ns	>0.9999
WKY:4 week vs. MPH:4 week	-26.11	-101.5 to 49.24	No	ns	>0.9999
WKY:4 week vs. LMQXM-LD:0 week	20.58	-54.77 to 95.94	No	ns	>0.9999
WKY:4 week vs. LMQXM-LD:4 week	-25.07	-100.4 to 50.28	No	ns	>0.9999
WKY:4 week vs. LMQXM-MD:0 week	21.22	-54.13 to 96.58	No	ns	>0.9999
WKY:4 week vs. LMQXM-MD:4 week	26.22	-49.13 to 101.6	No	ns	>0.9999
WKY:4 week vs. LMQXM-HD:0 week	20.59	-54.77 to 95.94	No	ns	>0.9999
WKY:4 week vs. LMQXM-HD:4 week	2.842	-72.51 to 78.20	No	ns	>0.9999
SHR:0 week vs. SHR:4 week	-61.25	-136.6 to 14.10	No	ns	0.3846

SHR:0 week vs. MPH:0 week	7.421	-67.93 to 82.77	No	ns	>0.9999
SHR:0 week vs. MPH:4 week	-45.76	-121.1 to 29.60	No	ns	>0.9999
SHR:0 week vs. LMQXM-LD:0 week	0.9408	-74.41 to 76.29	No	ns	>0.9999
SHR:0 week vs. LMQXM-LD:4 week	-44.71	-120.1 to 30.64	No	ns	>0.9999
SHR:0 week vs. LMQXM-MD:0 week	1.578	-73.78 to 76.93	No	ns	>0.9999
SHR:0 week vs. LMQXM-MD:4 week	6.578	-68.78 to 81.93	No	ns	>0.9999
SHR:0 week vs. LMQXM-HD:0 week	0.9417	-74.41 to 76.30	No	ns	>0.9999
SHR:0 week vs. LMQXM-HD:4 week	-16.80	-92.16 to 58.55	No	ns	>0.9999
SHR:4 week vs. MPH:0 week	68.67	-6.681 to 144.0	No	ns	0.1365
SHR:4 week vs. MPH:4 week	15.50	-59.86 to 90.85	No	ns	>0.9999
SHR:4 week vs. LMQXM-LD:0 week	62.19	-13.16 to 137.5	No	ns	0.3389
SHR:4 week vs. LMQXM-LD:4 week	16.54	-58.81 to 91.89	No	ns	>0.9999
SHR:4 week vs. LMQXM-MD:0 week	62.83	-12.52 to 138.2	No	ns	0.3108
SHR:4 week vs. LMQXM-MD:4 week	67.83	-7.524 to 143.2	No	ns	0.1542
SHR:4 week vs. LMQXM-HD:0 week	62.19	-13.16 to 137.5	No	ns	0.3389
SHR:4 week vs. LMQXM-HD:4 week	44.45	-30.90 to 119.8	No	ns	>0.9999
MPH:0 week vs. MPH:4 week	-53.18	-128.5 to 22.18	No	ns	>0.9999
MPH:0 week vs. LMQXM-LD:0 week	-6.480	-81.83 to 68.87	No	ns	>0.9999
MPH:0 week vs. LMQXM-LD:4 week	-52.13	-127.5 to 23.22	No	ns	>0.9999
MPH:0 week vs. LMQXM-MD:0 week	-5.842	-81.20 to 69.51	No	ns	>0.9999
MPH:0 week vs. LMQXM-MD:4 week	-0.8425	-76.20 to 74.51	No	ns	>0.9999
MPH:0 week vs. LMQXM-HD:0 week	-6.479	-81.83 to 68.87	No	ns	>0.9999
MPH:0 week vs. LMQXM-HD:4 week	-24.22	-99.58 to 51.13	No	ns	>0.9999
MPH:4 week vs. LMQXM-LD:0 week	46.70	-28.66 to 122.1	No	ns	>0.9999
MPH:4 week vs. LMQXM-LD:4 week	1.044	-74.31 to 76.40	No	ns	>0.9999
MPH:4 week vs. LMQXM-MD:0 week	47.34	-28.02 to 122.7	No	ns	>0.9999
MPH:4 week vs. LMQXM-MD:4 week	52.34	-23.02 to 127.7	No	ns	>0.9999
MPH:4 week vs. LMQXM-HD:0 week	46.70	-28.66 to 122.1	No	ns	>0.9999
MPH:4 week vs. LMQXM-HD:4 week	28.96	-46.40 to 104.3	No	ns	>0.9999
LMQXM-LD:0 week vs. LMQXM-LD:4 week	-45.65	-121.0 to 29.70	No	ns	>0.9999
LMQXM-LD:0 week vs. LMQXM-MD:0 week	0.6375	-74.72 to 75.99	No	ns	>0.9999
LMQXM-LD:0 week vs. LMQXM-MD:4 week	5.638	-69.72 to 80.99	No	ns	>0.9999
LMQXM-LD:0 week vs. LMQXM-HD:0 week	0.0008333	-75.35 to 75.35	No	ns	>0.9999
LMQXM-LD:0 week vs. LMQXM-HD:4 week	-17.74	-93.10 to 57.61	No	ns	>0.9999
LMQXM-LD:4 week vs. LMQXM-MD:0 week	46.29	-29.06 to 121.6	No	ns	>0.9999
LMQXM-LD:4 week vs. LMQXM-MD:4 week	51.29	-24.06 to 126.6	No	ns	>0.9999
LMQXM-LD:4 week vs. LMQXM-HD:0 week	45.65	-29.70 to 121.0	No	ns	>0.9999
LMQXM-LD:4 week vs. LMQXM-HD:4 week	27.91	-47.44 to 103.3	No	ns	>0.9999
LMQXM-MD:0 week vs. LMQXM-MD:4 week	5.000	-70.35 to 80.35	No	ns	>0.9999
LMQXM-MD:0 week vs. LMQXM-HD:0 week	-0.6367	-75.99 to 74.72	No	ns	>0.9999
LMQXM-MD:0 week vs. LMQXM-HD:4 week	-18.38	-93.73 to 56.97	No	ns	>0.9999
LMQXM-MD:4 week vs. LMQXM-HD:0 week	-5.637	-80.99 to 69.72	No	ns	>0.9999
LMQXM-MD:4 week vs. LMQXM-HD:4 week	-23.38	-98.73 to 51.97	No	ns	>0.9999
LMQXM-HD:0 week vs. LMQXM-HD:4 week	-17.74	-93.10 to 57.61	No	ns	>0.9999

Supplementary Table 12 Changes in the escape latency of rats. （$\bar{x} \pm s$，n=12）

Group	Day 1（s）	Day 2（s）	Day 3（s）	Day 4（s）	Day 5（s）
WKY	38.79±7.57	41.95±10.68	37.17±13.85	31.21±11.87	31.83±8.54
SHR	38.12±5.01	34.14±6.61	31.62±6.94	26.44±3.80	24.41±4.18
MPH	39.53±5.63	34.25±9.92	24.53±8.45	19.68±5.848*	18.58±5.21*

LMQXM-LD	35.30±7.36	26.04±7.81*	24.99±7.30	20.27±8.97*	21.72±5.36
LMQXM-MD	37.731±4.84	29.08±10.23	22.94±6.76*	20.06±5.24*	17.91±3.49*
LMQXM-HD	35.09±7.95	29.86±4.94	23.41±6.59*	19.54±4.20*	16.25±2.34*

Note: Compared with the SHR group, $^{*}P < 0.05$, $^{**}P < 0.01$.

Supplementary Table 13 Effects of LMQXM on spatial learning and memory in rats （$\bar{x} \pm s$，*n*=12）

Group	Annulus visits	Time spent in the target quadrant（s）	Swimming distance in the target quadrant（cm）
WKY	2.00±0.48	15.65±5.50	226.62±71.06
SHR	1.63±0.86	11.09±2.76#	216.98±73.36
MPH	2.42±0.70*	14.96±4.10*	288.48±83.14*
LMQXM-LD	2.46±1.12*	15.16±3.36*	289.08±67.15*
LMQXM-MD	2.83±1.21**	15.86±5.15**	307.01±113.53*
LMQXM-HD	2.63±1.19*	16.41±3.90**	314.45±93.19*
F -value	2.501	2.452	2.871
P- value	0.039	0.042	0.021

Note: Compared with the WKY group, $^{\#}P < 0.05$; compared with the SHR group, $^{*}P < 0.05$, $^{**}P < 0.01$.

Supplementary Table 14 Analysis of two-way repeated measures ANOVA on the latency of escape in rats

Multivariate test[a]

effect		price	F	Assuming degrees of freedom	Error degrees of freedom	significance
time	Billay trajectory	.806	65.475[b]	4.000	63.000	.000
	Wilke Lambda	.194	65.475[b]	4.000	63.000	.000
	Hortlin Tracks	4.157	65.475[b]	4.000	63.000	.000
	Roy's biggest root	4.157	65.475[b]	4.000	63.000	.000
The time * grouping	Billay trajectory	.456	1.700	20.000	264.000	.033
	Wilke Lambda	.593	1.790	20.000	209.897	.023
	Hortlin Tracks	.605	1.859	20.000	246.000	.016
	Roy's biggest root	.436	5.759[c]	5.000	66.000	.000

Mokhirai sphericity test[a]							
Measurement: escape latency							
Within-subject effect	Motch came to the W	Approximate chi square	free degree	significance	Epsilon[b]		
					Greenhouse-Gaessler	Xin-ferdt	lower limit
time	.640	28.781	9	.001	.857	.978	.250

Within-subject effect test						
Measurement: escape latency						
source		Class III and the sum of the squares	free degree	mean square	F	significance
time	Globular degree is assumed	12531.520	4	3132.880	70.423	.000
	Greenhouse-Gaessler	12531.520	3.429	3654.623	70.423	.000
	Xin-ferdt	12531.520	3.914	3201.961	70.423	.000
	lower limit	12531.520	1.000	12531.520	70.423	.000
The time * grouping	Globular degree is assumed	1603.905	20	80.195	1.803	.020
	Greenhouse-Gaessler	1603.905	17.145	93.551	1.803	.028
	Xin-ferdt	1603.905	19.569	81.964	1.803	.022
	lower limit	1603.905	5.000	320.781	1.803	.124
Error (time)	Globular degree is assumed	11744.399	264	44.486		
	Greenhouse-Gaessler	11744.399	226.311	51.895		
	Xin-ferdt	11744.399	258.304	45.467		
	lower limit	11744.399	66.000	177.945		

Inter-subject effect test						
Measurement: escape latency						
Conversion variables: average						
source	Class III and the sum of the squares	free degree	mean square	F	significance	Partial Eta-square
nodal increment	290650.358	1	290650.358	3077.255	.000	.979
divide into groups	5801.710	5	1160.342	12.285	.000	.482
error	6233.779	66	94.451			

Paired comparison						
Measurement: escape latency						
time	(I) divide	(J) divide	Mean Difference	standard	significance[b]	The 95% confidence interval of the difference[b]

	into groups	into groups	Value (I-J)	error		lower limit	superior limit
1	WKY	SHR	.675	2.661	.801	-4.637	5.987
		MPH	-.739	2.661	.782	-6.051	4.573
		LD	3.494	2.661	.194	-1.818	8.806
		MD	1.059	2.661	.692	-4.253	6.371
		HD	3.700	2.661	.169	-1.612	9.012
	SHR	WKY	-.675	2.661	.801	-5.987	4.637
		MPH	-1.414	2.661	.597	-6.726	3.898
		LD	2.819	2.661	.293	-2.493	8.131
		MD	.384	2.661	.886	-4.928	5.696
		HD	3.025	2.661	.260	-2.287	8.337
	MPH	WKY	.739	2.661	.782	-4.573	6.051
		SHR	1.414	2.661	.597	-3.898	6.726
		LD	4.233	2.661	.116	-1.079	9.546
		MD	1.798	2.661	.501	-3.514	7.111
		HD	4.439	2.661	.100	-.873	9.751
	LD	WKY	-3.494	2.661	.194	-8.806	1.818
		SHR	-2.819	2.661	.293	-8.131	2.493
		MPH	-4.233	2.661	.116	-9.546	1.079
		MD	-2.435	2.661	.363	-7.747	2.877
		HD	.206	2.661	.939	-5.106	5.518
	MD	WKY	-1.059	2.661	.692	-6.371	4.253
		SHR	-.384	2.661	.886	-5.696	4.928
		MPH	-1.798	2.661	.501	-7.111	3.514
		LD	2.435	2.661	.363	-2.877	7.747
		HD	2.641	2.661	.325	-2.671	7.953
	HD	WKY	-3.700	2.661	.169	-9.012	1.612
		SHR	-3.025	2.661	.260	-8.337	2.287
		MPH	-4.439	2.661	.100	-9.751	.873
		LD	-.206	2.661	.939	-5.518	5.106
		MD	-2.641	2.661	.325	-7.953	2.671
2	WKY	SHR	7.806*	3.524	.030	.770	14.842
		MPH	7.697*	3.524	.033	.661	14.733
		LD	15.909*	3.524	.000	8.873	22.945
		MD	12.871*	3.524	.001	5.835	19.907
		HD	12.086*	3.524	.001	5.050	19.122
	SHR	WKY	-7.806*	3.524	.030	-14.842	-.770
		MPH	-.109	3.524	.975	-7.145	6.927

		LD	8.102*	3.524	.025	1.067	15.138
		MD	5.065	3.524	.155	-1.971	12.101
		HD	4.280	3.524	.229	-2.756	11.316
	MPH	WKY	-7.697*	3.524	.033	-14.733	-.661
		SHR	.109	3.524	.975	-6.927	7.145
		LD	8.212*	3.524	.023	1.176	15.248
		MD	5.174	3.524	.147	-1.862	12.210
		HD	4.389	3.524	.217	-2.647	11.425
	LD	WKY	-15.909*	3.524	.000	-22.945	-8.873
		SHR	-8.102*	3.524	.025	-15.138	-1.067
		MPH	-8.212*	3.524	.023	-15.248	-1.176
		MD	-3.037	3.524	.392	-10.073	3.998
		HD	-3.822	3.524	.282	-10.858	3.213
	MD	WKY	-12.871*	3.524	.001	-19.907	-5.835
		SHR	-5.065	3.524	.155	-12.101	1.971
		MPH	-5.174	3.524	.147	-12.210	1.862
		LD	3.037	3.524	.392	-3.998	10.073
		HD	-.785	3.524	.824	-7.821	6.251
	HD	WKY	-12.086*	3.524	.001	-19.122	-5.050
		SHR	-4.280	3.524	.229	-11.316	2.756
		MPH	-4.389	3.524	.217	-11.425	2.647
		LD	3.822	3.524	.282	-3.213	10.858
		MD	.785	3.524	.824	-6.251	7.821
3	WKY	SHR	5.549	3.550	.123	-1.539	12.638
		MPH	12.634*	3.550	.001	5.546	19.723
		LD	12.178*	3.550	.001	5.090	19.267
		MD	14.222*	3.550	.000	7.134	21.311
		HD	13.761*	3.550	.000	6.672	20.849
	SHR	WKY	-5.549	3.550	.123	-12.638	1.539
		MPH	7.085	3.550	.050	-.004	14.174
		LD	6.629	3.550	.066	-.459	13.718
		MD	8.673*	3.550	.017	1.585	15.762
		HD	8.212*	3.550	.024	1.123	15.300
	MPH	WKY	-12.634*	3.550	.001	-19.723	-5.546
		SHR	-7.085	3.550	.050	-14.174	.004
		LD	-.456	3.550	.898	-7.544	6.633
		MD	1.588	3.550	.656	-5.500	8.677
		HD	1.127	3.550	.752	-5.962	8.215
	LD	WKY	-12.178*	3.550	.001	-19.267	-5.090
		SHR	-6.629	3.550	.066	-13.718	.459

		MPH	.456	3.550	.898	-6.633	7.544
		MD	2.044	3.550	.567	-5.044	9.133
		HD	1.582	3.550	.657	-5.506	8.671
	MD	WKY	-14.222*	3.550	.000	-21.311	-7.134
		SHR	-8.673*	3.550	.017	-15.762	-1.585
		MPH	-1.588	3.550	.656	-8.677	5.500
		LD	-2.044	3.550	.567	-9.133	5.044
		HD	-.462	3.550	.897	-7.550	6.627
	HD	WKY	-13.761*	3.550	.000	-20.849	-6.672
		SHR	-8.212*	3.550	.024	-15.300	-1.123
		MPH	-1.127	3.550	.752	-8.215	5.962
		LD	-1.582	3.550	.657	-8.671	5.506
		MD	.462	3.550	.897	-6.627	7.550
4	WKY	SHR	4.770	2.957	.112	-1.134	10.674
		MPH	11.531*	2.957	.000	5.626	17.435
		LD	10.934*	2.957	.000	5.030	16.839
		MD	11.147*	2.957	.000	5.242	17.051
		HD	11.668*	2.957	.000	5.763	17.572
	SHR	WKY	-4.770	2.957	.112	-10.674	1.134
		MPH	6.761*	2.957	.025	.856	12.665
		LD	6.164*	2.957	.041	.260	12.069
		MD	6.377*	2.957	.035	.472	12.281
		HD	6.898*	2.957	.023	.993	12.802
	MPH	WKY	-11.531*	2.957	.000	-17.435	-5.626
		SHR	-6.761*	2.957	.025	-12.665	-.856
		LD	-.597	2.957	.841	-6.501	5.308
		MD	-.384	2.957	.897	-6.289	5.520
		HD	.137	2.957	.963	-5.768	6.041
	LD	WKY	-10.934*	2.957	.000	-16.839	-5.030
		SHR	-6.164*	2.957	.041	-12.069	-.260
		MPH	.597	2.957	.841	-5.308	6.501
		MD	.212	2.957	.943	-5.692	6.117
		HD	.733	2.957	.805	-5.171	6.638
	MD	WKY	-11.147*	2.957	.000	-17.051	-5.242
		SHR	-6.377*	2.957	.035	-12.281	-.472
		MPH	.384	2.957	.897	-5.520	6.289
		LD	-.212	2.957	.943	-6.117	5.692
		HD	.521	2.957	.861	-5.384	6.425
	HD	WKY	-11.668*	2.957	.000	-17.572	-5.763

		SHR	-6.898*	2.957	.023	-12.802	-.993
		MPH	-.137	2.957	.963	-6.041	5.768
		LD	-.733	2.957	.805	-6.638	5.171
		MD	-.521	2.957	.861	-6.425	5.384
5	WKY	SHR	7.416*	2.133	.001	3.157	11.675
		MPH	13.250*	2.133	.000	8.991	17.509
		LD	10.106*	2.133	.000	5.847	14.365
		MD	13.916*	2.133	.000	9.657	18.175
		HD	15.578*	2.133	.000	11.319	19.837
	SHR	WKY	-7.416*	2.133	.001	-11.675	-3.157
		MPH	5.834*	2.133	.008	1.575	10.093
		LD	2.690	2.133	.212	-1.569	6.949
		MD	6.500*	2.133	.003	2.241	10.759
		HD	8.162*	2.133	.000	3.904	12.421
	MPH	WKY	-13.250*	2.133	.000	-17.509	-8.991
		SHR	-5.834*	2.133	.008	-10.093	-1.575
		LD	-3.144	2.133	.145	-7.403	1.115
		MD	.666	2.133	.756	-3.593	4.925
		HD	2.328	2.133	.279	-1.931	6.587
	LD	WKY	-10.106*	2.133	.000	-14.365	-5.847
		SHR	-2.690	2.133	.212	-6.949	1.569
		MPH	3.144	2.133	.145	-1.115	7.403
		MD	3.810	2.133	.079	-.449	8.069
		HD	5.472*	2.133	.013	1.214	9.731
	MD	WKY	-13.916*	2.133	.000	-18.175	-9.657
		SHR	-6.500*	2.133	.003	-10.759	-2.241
		MPH	-.666	2.133	.756	-4.925	3.593
		LD	-3.810	2.133	.079	-8.069	.449
		HD	1.662	2.133	.439	-2.596	5.921
	HD	WKY	-15.578*	2.133	.000	-19.837	-11.319
		SHR	-8.162*	2.133	.000	-12.421	-3.904
		MPH	-2.328	2.133	.279	-6.587	1.931
		LD	-5.472*	2.133	.013	-9.731	-1.214
		MD	-1.662	2.133	.439	-5.921	2.596

Supplementary Table 15 One-ANOVA of annulus visits, time spent in the target quadrant and swimming distance in the target quadrant of rats.

**Homogeneity test of variance**				
	Levin statistics	Degree of freedom 1	Degree of freedom 2	significance

Annulus visits	Based on the mean	2.153	5	66	.070
	Based on the median	1.575	5	66	.179
	Based on the median and with the adjusted degrees of freedom	1.575	5	50.153	.184
	Based on the post-cut average value	2.091	5	66	.078
Time spent in the target quadrant	Based on the mean	1.040	5	66	.402
	Based on the median	.583	5	66	.713
	Based on the median and with the adjusted degrees of freedom	.583	5	41.545	.713
	Based on the post-cut average value	.905	5	66	.483
Target Quadrant swimming distance	Based on the mean	1.321	5	66	.266
	Based on the median	1.309	5	66	.271
	Based on the median and with the adjusted degrees of freedom	1.309	5	58.654	.273
	Based on the post-cut average value	1.329	5	66	.263

ANOVA

				F	significance
Annulus visits	interblock	(assemble)		2.501	.039
		linear term	contrast		.005
			bias in statistics		.412
	Within the group				
	Total				
Time spent in the target quadrant	interblock	(assemble)		2.452	.042
		linear term	contrast		.078
			bias in statistics		.071
	Within the group				
	Total				
Target Quadrant swimming distance	interblock	(assemble)		2.871	.021
		linear term	contrast		.001
			bias in statistics		.660
	Within the group				
	Total				

Multiple comparisons

LSD

dependent variable	(I) divide into groups	(J) divide into groups	Mean Difference Value (I-J)	Standard error	significance	And the 95% confidence interval	
						lower limit	superior limit
Annulus visits	WKY	SHR	.37500	.39388	.345	-.4114	1.1614
		MPH	-.41667	.39388	.294	-1.2031	.3697

		LD	-.45833	.39388	.249	-1.2447	.3281
		MD	-.83333*	.39388	.038	-1.6197	-.0469
		HD	-.62500	.39388	.117	-1.4114	.1614
	SHR	WKY	-.37500	.39388	.345	-1.1614	.4114
		MPH	-.79167*	.39388	.049	-1.5781	-.0053
		LD	-.83333*	.39388	.038	-1.6197	-.0469
		MD	-1.20833*	.39388	.003	-1.9947	-.4219
		HD	-1.00000*	.39388	.013	-1.7864	-.2136
	MPH	WKY	.41667	.39388	.294	-.3697	1.2031
		SHR	.79167*	.39388	.049	.0053	1.5781
		LD	-.04167	.39388	.916	-.8281	.7447
		MD	-.41667	.39388	.294	-1.2031	.3697
		HD	-.20833	.39388	.599	-.9947	.5781
	LD	WKY	.45833	.39388	.249	-.3281	1.2447
		SHR	.83333*	.39388	.038	.0469	1.6197
		MPH	.04167	.39388	.916	-.7447	.8281
		MD	-.37500	.39388	.345	-1.1614	.4114
		HD	-.16667	.39388	.674	-.9531	.6197
	MD	WKY	.83333*	.39388	.038	.0469	1.6197
		SHR	1.20833*	.39388	.003	.4219	1.9947
		MPH	.41667	.39388	.294	-.3697	1.2031
		LD	.37500	.39388	.345	-.4114	1.1614
		HD	.20833	.39388	.599	-.5781	.9947
	HD	WKY	.62500	.39388	.117	-.1614	1.4114
		SHR	1.00000*	.39388	.013	.2136	1.7864
		MPH	.20833	.39388	.599	-.5781	.9947
		LD	.16667	.39388	.674	-.6197	.9531
		MD	-.20833	.39388	.599	-.9947	.5781
Time spent in the target quadrant	WKY	SHR	4.56000*	1.72968	.010	1.1066	8.0134
		MPH	.69000	1.72968	.691	-2.7634	4.1434
		LD	.49167	1.72968	.777	-2.9618	3.9451
		MD	-.20833	1.72968	.904	-3.6618	3.2451
		HD	-.76167	1.72968	.661	-4.2151	2.6918
	SHR	WKY	-4.56000*	1.72968	.010	-8.0134	-1.1066
		MPH	-3.87000*	1.72968	.029	-7.3234	-.4166
		LD	-4.06833*	1.72968	.022	-7.5218	-.6149
		MD	-4.76833*	1.72968	.008	-8.2218	-1.3149
		HD	-5.32167*	1.72968	.003	-8.7751	-1.8682
	MPH	WKY	-.69000	1.72968	.691	-4.1434	2.7634
		SHR	3.87000*	1.72968	.029	.4166	7.3234

		LD	-.19833	1.72968	.909	-3.6518	3.2551
		MD	-.89833	1.72968	.605	-4.3518	2.5551
		HD	-1.45167	1.72968	.404	-4.9051	2.0018
	LD	WKY	-.49167	1.72968	.777	-3.9451	2.9618
		SHR	4.06833*	1.72968	.022	.6149	7.5218
		MPH	.19833	1.72968	.909	-3.2551	3.6518
		MD	-.70000	1.72968	.687	-4.1534	2.7534
		HD	-1.25333	1.72968	.471	-4.7068	2.2001
	MD	WKY	.20833	1.72968	.904	-3.2451	3.6618
		SHR	4.76833*	1.72968	.008	1.3149	8.2218
		MPH	.89833	1.72968	.605	-2.5551	4.3518
		LD	.70000	1.72968	.687	-2.7534	4.1534
		HD	-.55333	1.72968	.750	-4.0068	2.9001
	HD	WKY	.76167	1.72968	.661	-2.6918	4.2151
		SHR	5.32167*	1.72968	.003	1.8682	8.7751
		MPH	1.45167	1.72968	.404	-2.0018	4.9051
		LD	1.25333	1.72968	.471	-2.2001	4.7068
		MD	.55333	1.72968	.750	-2.9001	4.0068
Target Quadrant swimming distance	WKY	SHR	9.64299	34.73201	.782	-59.7017	78.9877
		MPH	-61.85487	34.73201	.080	-131.1996	7.4898
		LD	-62.45093	34.73201	.077	-131.7956	6.8938
		MD	-80.38598*	34.73201	.024	-149.7307	-11.0413
		HD	-87.83011*	34.73201	.014	-157.1748	-18.4854
	SHR	WKY	-9.64299	34.73201	.782	-78.9877	59.7017
		MPH	-71.49786*	34.73201	.043	-140.8425	-2.1532
		LD	-72.09391*	34.73201	.042	-141.4386	-2.7492
		MD	-90.02897*	34.73201	.012	-159.3737	-20.6843
		HD	-97.47310*	34.73201	.007	-166.8178	-28.1284
	MPH	WKY	61.85487	34.73201	.080	-7.4898	131.1996
		SHR	71.49786*	34.73201	.043	2.1532	140.8425
		LD	-.59605	34.73201	.986	-69.9407	68.7486
		MD	-18.53111	34.73201	.595	-87.8758	50.8136
		HD	-25.97524	34.73201	.457	-95.3199	43.3694
	LD	WKY	62.45093	34.73201	.077	-6.8938	131.7956
		SHR	72.09391*	34.73201	.042	2.7492	141.4386
		MPH	.59605	34.73201	.986	-68.7486	69.9407
		MD	-17.93506	34.73201	.607	-87.2797	51.4096
		HD	-25.37919	34.73201	.468	-94.7239	43.9655
	MD	WKY	80.38598*	34.73201	.024	11.0413	149.7307

		SHR	90.02897*	34.73201	.012	20.6843	159.3737
		MPH	18.53111	34.73201	.595	-50.8136	87.8758
		LD	17.93506	34.73201	.607	-51.4096	87.2797
		HD	-7.44413	34.73201	.831	-76.7888	61.9006
	HD	WKY	87.83011*	34.73201	.014	18.4854	157.1748
		SHR	97.47310*	34.73201	.007	28.1284	166.8178
		MPH	25.97524	34.73201	.457	-43.3694	95.3199
		LD	25.37919	34.73201	.468	-43.9655	94.7239
		MD	7.44413	34.73201	.831	-61.9006	76.7888

Supplementary Table 16 Effects of LMQXM on DA and NE levels in rat PFC and Striatum（$\bar{x} \pm s$，n_{DA}=6，n_{NE}=4）

Group	DA（ng/g）		NE（ng/g）	
	PFC	Striatum	PFC	Striatum
WKY	7505.68±2234.068	4005.49±1972.07	99.34±59.10	117.89±50.78
SHR	2460.568±999.99$^{\#\#}$	1057.43±448.23$^{\#\#}$	15.62±5.37$^{\#\#}$	30.20±14.56$^{\#}$
MPH	4965.019±962.11	3220.06±804.68*	99.00±39.25**	125.33±63.51**
LMQXM-LD	5459.04±4484.34*	3237.253±1743.31*	56.50±17.91	75.46±21.54
LMQXM-MD	5651.57±1075.10*	3767.96±2307.14**	97.57±39.53**	124.65±63.35*
LMQXM-HD	3896.99±1522.11	2912.83±1040.71*	60.12±49.62	62.16±39.32
F -value	3.442	2.782	2.902	2.909
P- value	0.014	0.035	0.043	0.043

Note: Compared with the WKY group, $^{\#}P < 0.05$, $^{\#\#}P < 0.01$; compared with the SHR group, $^{*}P < 0.05$, $^{**}P < 0.01$.

Supplementary Table 17 One-ANOVA of DA and NE level.

(1)

Homogeneity test of variance					
		Levin statistics	Degree of freedom 1	Degree of freedom 2	significance
DA of PFC	Based on the mean	2.516	5	30	.051
	Based on the median	1.238	5	30	.316
	Based on the median and with the adjusted degrees of freedom	1.238	5	7.555	.379
	Based on the post-cut average value	2.234	5	30	.077
DA of Striatum	Based on the mean	1.679	5	30	.170
	Based on the median	.572	5	30	.721

	Based on the median and with the adjusted degrees of freedom	.572	5	16.945	.721
	Based on the post-cut average value	1.325	5	30	.280

ANOVA

				quadratic sum	free degree	mean square	F
DA of PFC	interblock	(assemble)		87477963.585	5	17495592.717	3.442
		linear term	contrast	5453402.587	1	5453402.587	1.073
			bias	82024560.998	4	20506140.250	4.034
	Within the group			152493281.372	30	5083109.379	
	Total			239971244.957	35		
DA of Striatum	interblock	(assemble)		32879439.253	5	6575887.851	2.782
		linear term	contrast	618158.860	1	618158.860	.262
			bias	32261280.393	4	8065320.098	3.412
	Within the group			70912969.460	30	2363765.649	
	Total			103792408.713	35		

ANOVA

				significance
DA of PFC	interblock	(assemble)		.014
		linear term	contrast	.309
			bias	.010
	Within the group			
	Total			
DA of Striatum	interblock	(assemble)		.035
		linear term	contrast	.613
			bias	.021
	Within the group			
	Total			

Multiple comparisons

LSD

dependent variable	(I) divide into groups	(J) divide into groups	Mean Difference Value (I-J)	Standard error	significance	And the 95% confidence interval lower limit	superior limit
DA of PFC	WKY	SHR	5045.11429*	1301.67960	.001	2386.7299	7703.4987
		MPH	2540.66817	1301.67960	.060	-117.7162	5199.0526
		LD	2046.63820	1301.67960	.126	-611.7462	4705.0226
		MD	1854.10701	1301.67960	.165	-804.2774	4512.4914
		HD	3608.69128*	1301.67960	.009	950.3069	6267.0757
	SHR	WKY	-5045.11429*	1301.67960	.001	-7703.4987	-2386.7299

		MPH	-2504.44612	1301.67960	.064	-5162.8305	153.9383
		LD	-2998.47609*	1301.67960	.028	-5656.8605	-340.0917
		MD	-3191.00728*	1301.67960	.020	-5849.3917	-532.6229
		HD	-1436.42301	1301.67960	.279	-4094.8074	1221.9614
	MPH	WKY	-2540.66817	1301.67960	.060	-5199.0526	117.7162
		SHR	2504.44612	1301.67960	.064	-153.9383	5162.8305
		LD	-494.02997	1301.67960	.707	-3152.4144	2164.3544
		MD	-686.56117	1301.67960	.602	-3344.9456	1971.8232
		HD	1068.02311	1301.67960	.418	-1590.3613	3726.4075
	LD	WKY	-2046.63820	1301.67960	.126	-4705.0226	611.7462
		SHR	2998.47609*	1301.67960	.028	340.0917	5656.8605
		MPH	494.02997	1301.67960	.707	-2164.3544	3152.4144
		MD	-192.53119	1301.67960	.883	-2850.9156	2465.8532
		HD	1562.05308	1301.67960	.240	-1096.3313	4220.4375
	MD	WKY	-1854.10701	1301.67960	.165	-4512.4914	804.2774
		SHR	3191.00728*	1301.67960	.020	532.6229	5849.3917
		MPH	686.56117	1301.67960	.602	-1971.8232	3344.9456
		LD	192.53119	1301.67960	.883	-2465.8532	2850.9156
		HD	1754.58428	1301.67960	.188	-903.8001	4412.9687
	HD	WKY	-3608.69128*	1301.67960	.009	-6267.0757	-950.3069
		SHR	1436.42301	1301.67960	.279	-1221.9614	4094.8074
		MPH	-1068.02311	1301.67960	.418	-3726.4075	1590.3613
		LD	-1562.05308	1301.67960	.240	-4220.4375	1096.3313
		MD	-1754.58428	1301.67960	.188	-4412.9687	903.8001
DA of Striatum	WKY	SHR	2948.05325*	887.64964	.002	1135.2308	4760.8757
		MPH	785.43080	887.64964	.383	-1027.3916	2598.2532
		LD	768.23244	887.64964	.394	-1044.5900	2581.0548
		MD	237.52826	887.64964	.791	-1575.2942	2050.3507
		HD	1092.65679	887.64964	.228	-720.1656	2905.4792
	SHR	WKY	-2948.05325*	887.64964	.002	-4760.8757	-1135.2308
		MPH	-2162.62246*	887.64964	.021	-3975.4449	-349.8000
		LD	-2179.82082*	887.64964	.020	-3992.6432	-366.9984
		MD	-2710.52499*	887.64964	.005	-4523.3474	-897.7026
		HD	-1855.39647*	887.64964	.045	-3668.2189	-42.5741
	MPH	WKY	-785.43080	887.64964	.383	-2598.2532	1027.3916
		SHR	2162.62246*	887.64964	.021	349.8000	3975.4449
		LD	-17.19836	887.64964	.985	-1830.0208	1795.6241
		MD	-547.90254	887.64964	.542	-2360.7249	1264.9199
		HD	307.22599	887.64964	.732	-1505.5964	2120.0484
	LD	WKY	-768.23244	887.64964	.394	-2581.0548	1044.5900

		SHR	2179.82082*	887.64964	.020	366.9984	3992.6432
		MPH	17.19836	887.64964	.985	-1795.6241	1830.0208
		MD	-530.70418	887.64964	.554	-2343.5266	1282.1182
		HD	324.42435	887.64964	.717	-1488.3981	2137.2468
	MD	WKY	-237.52826	887.64964	.791	-2050.3507	1575.2942
		SHR	2710.52499*	887.64964	.005	897.7026	4523.3474
		MPH	547.90254	887.64964	.542	-1264.9199	2360.7249
		LD	530.70418	887.64964	.554	-1282.1182	2343.5266
		HD	855.12853	887.64964	.343	-957.6939	2667.9509
	HD	WKY	-1092.65679	887.64964	.228	-2905.4792	720.1656
		SHR	1855.39647*	887.64964	.045	42.5741	3668.2189
		MPH	-307.22599	887.64964	.732	-2120.0484	1505.5964
		LD	-324.42435	887.64964	.717	-2137.2468	1488.3981
		MD	-855.12853	887.64964	.343	-2667.9509	957.6939

(2)

Homogeneity test of variance

		Levin statistics	Degree of freedom 1	Degree of freedom 2	significance
prefrontal lobe NE	Based on the mean	2.389	5	18	.079
	Based on the median	1.498	5	18	.240
	Based on the median and with the adjusted degrees of freedom	1.498	5	9.151	.280
	Based on the post-cut average value	2.249	5	18	.094
Striatum NE	Based on the mean	2.755	5	18	.051
	Based on the median	1.637	5	18	.201
	Based on the median and with the adjusted degrees of freedom	1.637	5	10.171	.236
	Based on the post-cut average value	2.558	5	18	.064

ANOVA

				quadratic sum	free degree	mean square	F	significance
prefrontal lobe NE	interblock	(assemble)		22752.230	5	4550.446	2.902	.043
		linear term	contrast	3.011	1	3.011	.002	.966
			bias in statistics	22749.220	4	5687.305	3.627	.025
	Within the group			28222.967	18	1567.943		
	Total			50975.197	23			

Striatum NE	interblock	(assemble)		31144.224	5	6228.845	2.909	.043
		linear term	contrast	116.716	1	116.716	.055	.818
			bias in statistics	31027.508	4	7756.877	3.623	.025
	Within the group			38541.159	18	2141.176		
	Total			69685.383	23			

Multiple comparisons

LSD

dependent variable	(I) divide into groups	(J) divide into groups	Mean Difference Value (I-J)	Standard error	significance	And the 95% confidence interval	
						lower limit	superior limit
prefrontal lobe NE	WKY	SHR	83.72363*	27.99949	.008	24.8989	142.5484
		MPH	.34109	27.99949	.990	-58.4837	59.1658
		LD	42.84074	27.99949	.143	-15.9840	101.6655
		MD	1.77366	27.99949	.950	-57.0511	60.5984
		HD	39.21838	27.99949	.178	-19.6064	98.0431
	SHR	WKY	-83.72363*	27.99949	.008	-142.5484	-24.8989
		MPH	-83.38255*	27.99949	.008	-142.2073	-24.5578
		LD	-40.88289	27.99949	.161	-99.7076	17.9418
		MD	-81.94997*	27.99949	.009	-140.7747	-23.1252
		HD	-44.50525	27.99949	.129	-103.3300	14.3195
	MPH	WKY	-.34109	27.99949	.990	-59.1658	58.4837
		SHR	83.38255*	27.99949	.008	24.5578	142.2073
		LD	42.49965	27.99949	.146	-16.3251	101.3244
		MD	1.43257	27.99949	.960	-57.3922	60.2573
		HD	38.87729	27.99949	.182	-19.9474	97.7020
	LD	WKY	-42.84074	27.99949	.143	-101.6655	15.9840
		SHR	40.88289	27.99949	.161	-17.9418	99.7076
		MPH	-42.49965	27.99949	.146	-101.3244	16.3251
		MD	-41.06708	27.99949	.160	-99.8918	17.7577
		HD	-3.62236	27.99949	.898	-62.4471	55.2024
	MD	WKY	-1.77366	27.99949	.950	-60.5984	57.0511
		SHR	81.94997*	27.99949	.009	23.1252	140.7747
		MPH	-1.43257	27.99949	.960	-60.2573	57.3922
		LD	41.06708	27.99949	.160	-17.7577	99.8918
		HD	37.44472	27.99949	.198	-21.3800	96.2695
	HD	WKY	-39.21838	27.99949	.178	-98.0431	19.6064
		SHR	44.50525	27.99949	.129	-14.3195	103.3300
		MPH	-38.87729	27.99949	.182	-97.7020	19.9474

		LD	3.62236	27.99949	.898	-55.2024	62.4471
		MD	-37.44472	27.99949	.198	-96.2695	21.3800
Striatum NE	WKY	SHR	87.69394*	32.71984	.015	18.9521	156.4358
		MPH	-7.43570	32.71984	.823	-76.1775	61.3061
		LD	42.43147	32.71984	.211	-26.3104	111.1733
		MD	-6.75352	32.71984	.839	-75.4953	61.9883
		HD	55.73393	32.71984	.106	-13.0079	124.4758
	SHR	WKY	-87.69394*	32.71984	.015	-156.4358	-18.9521
		MPH	-95.12964*	32.71984	.009	-163.8715	-26.3878
		LD	-45.26247	32.71984	.183	-114.0043	23.4794
		MD	-94.44746*	32.71984	.010	-163.1893	-25.7056
		HD	-31.96001	32.71984	.342	-100.7018	36.7818
	MPH	WKY	7.43570	32.71984	.823	-61.3061	76.1775
		SHR	95.12964*	32.71984	.009	26.3878	163.8715
		LD	49.86717	32.71984	.145	-18.8747	118.6090
		MD	.68218	32.71984	.984	-68.0596	69.4240
		HD	63.16963	32.71984	.069	-5.5722	131.9115
	LD	WKY	-42.43147	32.71984	.211	-111.1733	26.3104
		SHR	45.26247	32.71984	.183	-23.4794	114.0043
		MPH	-49.86717	32.71984	.145	-118.6090	18.8747
		MD	-49.18499	32.71984	.150	-117.9268	19.5568
		HD	13.30246	32.71984	.689	-55.4394	82.0443
	MD	WKY	6.75352	32.71984	.839	-61.9883	75.4953
		SHR	94.44746*	32.71984	.010	25.7056	163.1893
		MPH	-.68218	32.71984	.984	-69.4240	68.0596
		LD	49.18499	32.71984	.150	-19.5568	117.9268
		HD	62.48745	32.71984	.072	-6.2544	131.2293
	HD	WKY	-55.73393	32.71984	.106	-124.4758	13.0079
		SHR	31.96001	32.71984	.342	-36.7818	100.7018
		MPH	-63.16963	32.71984	.069	-131.9115	5.5722
		LD	-13.30246	32.71984	.689	-82.0443	55.4394
		MD	-62.48745	32.71984	.072	-131.2293	6.2544

Supplementary Table 18 Effects of LMQXM on AC and cAMP levels in rat PFC and Striatum （$\bar{x} \pm s$，n=6）

分组	AC（ng/g）		cAMP（ng/g）	
	PFC	Striatum	PFC	Striatum
WKY	1.15±0.278	0.72±0.21	0.71±0.11	0.40±0.12

SHR	$0.50\pm0.07^{\#\#\#}$	$0.34\pm0.10^{\#\#}$	$0.36\pm0.11^{\#\#}$	$0.24\pm0.06^{\#}$
MPH	$0.98\pm0.18^{**}$	$0.65\pm0.22^{*}$	$0.68\pm0.22^{**}$	$0.40\pm0.12^{*}$
LMQXM-LD	$0.78\pm0.28^{*\wedge}$	$0.69\pm0.30^{*}$	$0.63\pm0.08^{*}$	$0.63\pm0.12^{***}$
LMQXM-MD	$1.13\pm0.14^{***}$	$0.72\pm0.27^{**}$	$0.80\pm0.30^{***}$	$0.45\pm0.15^{**}$
LMQXM-HD	$0.76\pm0.26^{*\wedge}$	0.54 ± 0.21	$0.50\pm0.22^{\wedge}$	$0.42\pm0.16^{*}$
F -value	8.206	2.600	4.127	5.988
P- value	<0.001	0.046	0.006	0.001

Note: Compared with the WKY group, $^{\#}P < 0.05$, $^{\#\#}P < 0.01$, $^{\#\#\#}P < 0.001$; compared with the SHR group, $^{*}P < 0.05$, $^{**}P < 0.01$, $^{***}P < 0.001$; compared with the LMQXM-MD group, $^{\wedge}P < 0.05$.

Supplementary Table 19 One-ANOVA of AC and cAMP levels in rat PFC and Striatum.

Homogeneity test of variance

		Levin statistics	Degree of freedom 1	Degree of freedom 2	significance
AC of PFC	Based on the mean	2.216	5	30	.079
	Based on the median	1.189	5	30	.338
	Based on the median and with the adjusted degrees of freedom	1.189	5	18.378	.353
	Based on the post-cut average value	2.013	5	30	.105
AC of Striatum	Based on the mean	.944	5	30	.467
	Based on the median	.730	5	30	.607
	Based on the median and with the adjusted degrees of freedom	.730	5	20.976	.609
	Based on the post-cut average value	.936	5	30	.472
cAMP of PFC	Based on the mean	2.375	5	30	.063
	Based on the median	1.348	5	30	.271
	Based on the median and with the adjusted degrees of freedom	1.348	5	13.013	.305
	Based on the post-cut average value	2.239	5	30	.076
cAMP of Striatum	Based on the mean	.709	5	30	.621
	Based on the median	.335	5	30	.888
	Based on the median and with the adjusted degrees of freedom	.335	5	25.068	.887
	Based on the post-cut average value	.652	5	30	.662

ANOVA

				quadratic sum	free degree	mean square	F	Significance
AC of PFC	interblock	(assemble)		1.910	5	.382	8.206	.000
		linear	contrast	.007	1	.007	.152	.699

		term	bias in statistics	1.903	4	.476	10.220	.000
	Within the group			1.396	30	.047		
	Total			3.306	35			
AC of Striatum	interblock	(assemble)		.672	5	.134	2.600	.046
		linear term	contrast	.005	1	.005	.106	.747
			bias in statistics	.666	4	.167	3.223	.026
	Within the group			1.551	30	.052		
	Total			2.222	35			
cAMP of PFC	interblock	(assemble)		.743	5	.149	4.127	.006
		linear term	contrast	.004	1	.004	.099	.756
			bias in statistics	.739	4	.185	5.134	.003
	Within the group			1.080	30	.036		
	Total			1.822	35			
cAMP of Striatum	interblock	(assemble)		.489	5	.098	5.988	.001
		linear term	contrast	.083	1	.083	5.075	.032
			bias in statistics	.406	4	.102	6.216	.001
	Within the group			.490	30	.016		
	Total			.979	35			

Multiple comparisons

Dependent variable		(I) divide into groups	(J) divide into groups	Mean Difference Value (I-J)	Standard error	Significance	And the 95% confidence interval	
							lower limit	superior limit
AC of PFC	LSD	WKY	SHR	.6546*	.1246	.000	.400	.909
			MPH	.1755	.1246	.169	-.079	.430
			LMQXM-LD	.3770*	.1246	.005	.123	.631
			LMQXM-MD	.0226	.1246	.857	-.232	.277
			LMQXM-HD	.3964*	.1246	.003	.142	.651
		SHR	WKY	-.6546*	.1246	.000	-.909	-.400
			MPH	-.4790*	.1246	.001	-.733	-.225
			LMQXM-LD	-.2776*	.1246	.033	-.532	-.023
			LMQXM-MD	-.6319*	.1246	.000	-.886	-.378
			LMQXM-HD	-.2582*	.1246	.047	-.513	-.004
		MPH	WKY	-.1755	.1246	.169	-.430	.079
			SHR	.4790*	.1246	.001	.225	.733
			LMQXM-LD	.2015	.1246	.116	-.053	.456

			LMQXM-MD	-.1529	.1246	.229	-.407	.102
			LMQXM-HD	.2209	.1246	.086	-.034	.475
		LMQXM -LD	WKY	-.3770*	.1246	.005	-.631	-.123
			SHR	.2776*	.1246	.033	.023	.532
			MPH	-.2015	.1246	.116	-.456	.053
			LMQXM-MD	-.3543*	.1246	.008	-.609	-.100
			LMQXM-HD	.0194	.1246	.877	-.235	.274
		LMQXM -MD	WKY	-.0226	.1246	.857	-.277	.232
			SHR	.6319*	.1246	.000	.378	.886
			MPH	.1529	.1246	.229	-.102	.407
			LMQXM-LD	.3543*	.1246	.008	.100	.609
			LMQXM-HD	.3737*	.1246	.005	.119	.628
		LMQXM -HD	WKY	-.3964*	.1246	.003	-.651	-.142
			SHR	.2582*	.1246	.047	.004	.513
			MPH	-.2209	.1246	.086	-.475	.034
			LMQXM-LD	-.0194	.1246	.877	-.274	.235
			LMQXM-MD	-.3737*	.1246	.005	-.628	-.119
AC of Striatum	LSD	WKY	SHR	.38121*	.13126	.007	.1131	.6493
			MPH	.06862	.13126	.605	-.1994	.3367
			LMQXM-LD	.03136	.13126	.813	-.2367	.2994
			LMQXM-MD	-.00198	.13126	.988	-.2700	.2661
			LMQXM-HD	.18671	.13126	.165	-.0814	.4548
		SHR	WKY	-.38121*	.13126	.007	-.6493	-.1131
			MPH	-.31259*	.13126	.024	-.5807	-.0445
			LMQXM-LD	-.34985*	.13126	.012	-.6179	-.0818
			LMQXM-MD	-.38319*	.13126	.007	-.6513	-.1151
			LMQXM-HD	-.19450	.13126	.149	-.4626	.0736
		MPH	WKY	-.06862	.13126	.605	-.3367	.1994
			SHR	.31259*	.13126	.024	.0445	.5807
			LMQXM-LD	-.03727	.13126	.778	-.3053	.2308
			LMQXM-MD	-.07061	.13126	.595	-.3387	.1975
			LMQXM-HD	.11809	.13126	.375	-.1500	.3862
		LMQXM -LD	WKY	-.03136	.13126	.813	-.2994	.2367
			SHR	.34985*	.13126	.012	.0818	.6179
			MPH	.03727	.13126	.778	-.2308	.3053
			LMQXM-MD	-.03334	.13126	.801	-.3014	.2347
			LMQXM-HD	.15535	.13126	.246	-.1127	.4234
		LMQXM -MD	WKY	.00198	.13126	.988	-.2661	.2700
			SHR	.38319*	.13126	.007	.1151	.6513
			MPH	.07061	.13126	.595	-.1975	.3387

			LMQXM-LD	.03334	.13126	.801	-.2347	.3014
			LMQXM-HD	.18870	.13126	.161	-.0794	.4568
		LMQXM-HD	WKY	-.18671	.13126	.165	-.4548	.0814
			SHR	.19450	.13126	.149	-.0736	.4626
			MPH	-.11809	.13126	.375	-.3862	.1500
			LMQXM-LD	-.15535	.13126	.246	-.4234	.1127
			LMQXM-MD	-.18870	.13126	.161	-.4568	.0794
cAMP of PFC	LSD	WKY	SHR	.34945*	.10953	.003	.1258	.5731
			MPH	.02591	.10953	.815	-.1978	.2496
			LMQXM-LD	.08550	.10953	.441	-.1382	.3092
			LMQXM-MD	-.08499	.10953	.444	-.3087	.1387
			LMQXM-HD	.20806	.10953	.067	-.0156	.4317
		SHR	WKY	-.34945*	.10953	.003	-.5731	-.1258
			MPH	-.32353*	.10953	.006	-.5472	-.0998
			LMQXM-LD	-.26395*	.10953	.022	-.4876	-.0403
			LMQXM-MD	-.43444*	.10953	.000	-.6581	-.2107
			LMQXM-HD	-.14139	.10953	.207	-.3651	.0823
		MPH	WKY	-.02591	.10953	.815	-.2496	.1978
			SHR	.32353*	.10953	.006	.0998	.5472
			LMQXM-LD	.05959	.10953	.590	-.1641	.2833
			LMQXM-MD	-.11090	.10953	.319	-.3346	.1128
			LMQXM-HD	.18214	.10953	.107	-.0415	.4058
		LMQXM-LD	WKY	-.08550	.10953	.441	-.3092	.1382
			SHR	.26395*	.10953	.022	.0403	.4876
			MPH	-.05959	.10953	.590	-.2833	.1641
			LMQXM-MD	-.17049	.10953	.130	-.3942	.0532
			LMQXM-HD	.12255	.10953	.272	-.1011	.3462
		LMQXM-MD	WKY	.08499	.10953	.444	-.1387	.3087
			SHR	.43444*	.10953	.000	.2107	.6581
			MPH	.11090	.10953	.319	-.1128	.3346
			LMQXM-LD	.17049	.10953	.130	-.0532	.3942
			LMQXM-HD	.29304*	.10953	.012	.0694	.5167
		LMQXM-HD	WKY	-.20806	.10953	.067	-.4317	.0156
			SHR	.14139	.10953	.207	-.0823	.3651
			MPH	-.18214	.10953	.107	-.4058	.0415
			LMQXM-LD	-.12255	.10953	.272	-.3462	.1011
			LMQXM-MD	-.29304*	.10953	.012	-.5167	-.0694
cAMP of Striatum	LSD	WKY	SHR	.16329*	.07379	.035	.0126	.3140
			MPH	.00492	.07379	.947	-.1458	.1556

			LMQXM-LD	-.23411*	.07379	.003	-.3848	-.0834
			LMQXM-MD	-.05344	.07379	.475	-.2041	.0973
			LMQXM-HD	-.01886	.07379	.800	-.1696	.1318
		SHR	WKY	-.16329*	.07379	.035	-.3140	-.0126
			MPH	-.15837*	.07379	.040	-.3091	-.0077
			LMQXM-LD	-.39740*	.07379	.000	-.5481	-.2467
			LMQXM-MD	-.21673*	.07379	.006	-.3674	-.0660
			LMQXM-HD	-.18215*	.07379	.019	-.3329	-.0314
		MPH	WKY	-.00492	.07379	.947	-.1556	.1458
			SHR	.15837*	.07379	.040	.0077	.3091
			LMQXM-LD	-.23902*	.07379	.003	-.3897	-.0883
			LMQXM-MD	-.05835	.07379	.435	-.2091	.0924
			LMQXM-HD	-.02378	.07379	.750	-.1745	.1269
		LMQXM-LD	WKY	.23411*	.07379	.003	.0834	.3848
			SHR	.39740*	.07379	.000	.2467	.5481
			MPH	.23902*	.07379	.003	.0883	.3897
			LMQXM-MD	.18067*	.07379	.020	.0300	.3314
			LMQXM-HD	.21525*	.07379	.007	.0645	.3660
		LMQXM-MD	WKY	.05344	.07379	.475	-.0973	.2041
			SHR	.21673*	.07379	.006	.0660	.3674
			MPH	.05835	.07379	.435	-.0924	.2091
			LMQXM-LD	-.18067*	.07379	.020	-.3314	-.0300
			LMQXM-HD	.03457	.07379	.643	-.1161	.1853
		LMQXM-HD	WKY	.01886	.07379	.800	-.1318	.1696
			SHR	.18215*	.07379	.019	.0314	.3329
			MPH	.02378	.07379	.750	-.1269	.1745
			LMQXM-LD	-.21525*	.07379	.007	-.3660	-.0645
			LMQXM-MD	-.03457	.07379	.643	-.1853	.1161

Supplementary Table 20 One-ANOVA of AOD of PKA, p-CREB, and BDNF in rat PFC and Striatum.

Description										
						The 95% confidence interval of the mean value				
		The number of cases	average value	standard deviations	Standard error	lower limit	superior limit	least value	crest value	Variance between components
PKA of PFC	WKY	4	.3650	.03284	.01642	.3128	.4173	.32	.39	
	SHR	4	.2023	.02669	.01335	.1599	.2448	.18	.24	
	MPH	4	.3464	.02040	.01020	.3139	.3788	.33	.38	

	LMQXM-LD		4	.3724	.05128	.02564	.2908	.4540	.31	.44	
	LMQXM-MD		4	.3920	.02532	.01266	.3517	.4322	.36	.41	
	LMQXM-HD		4	.3664	.02191	.01095	.3315	.4013	.34	.39	
	Total		24	.3407	.07044	.01438	.3110	.3705	.18	.44	
	model	fixed effect			.03151	.00643	.3272	.3543			
		stochastic effect				.02832	.2679	.4135			.00456
PKA of Striatum	WKY		4	.3326	.05691	.02845	.2421	.4232	.26	.40	
	SHR		4	.2496	.04981	.02491	.1704	.3289	.19	.31	
	MPH		4	.3613	.02616	.01308	.3196	.4029	.34	.39	
	LMQXM-LD		4	.3300	.02583	.01291	.2889	.3711	.31	.35	
	LMQXM-MD		4	.3924	.06097	.03049	.2954	.4894	.34	.47	
	LMQXM-HD		4	.3532	.01427	.00713	.3305	.3759	.34	.37	
	Total		24	.3365	.05877	.01200	.3117	.3613	.19	.47	
	model	fixed effect			.04280	.00874	.3182	.3549			
		stochastic effect				.01968	.2859	.3871			.00187
p-CREB of PFC	WKY		4	.6747	.05981	.02990	.5795	.7698	.60	.75	
	SHR		4	.5225	.05773	.02887	.4307	.6144	.47	.60	
	MPH		4	.6324	.08151	.04076	.5027	.7621	.57	.75	
	LMQXM-LD		4	.5689	.02527	.01264	.5287	.6091	.55	.61	
	LMQXM-MD		4	.6990	.08268	.04134	.5675	.8306	.63	.82	
	LMQXM-HD		4	.5674	.08452	.04226	.4329	.7019	.50	.69	
	Total		24	.6108	.08835	.01804	.5735	.6481	.47	.82	
	model	fixed effect			.06852	.01399	.5814	.6402			
		stochastic effect				.02814	.5385	.6832			.00358
p-CREB of Striatum	WKY		4	.6845	.19798	.09899	.3695	.9996	.48	.94	
	SHR		4	.4394	.01075	.00538	.4223	.4565	.42	.45	
	MPH		4	.7065	.06717	.03358	.5996	.8134	.64	.80	
	LMQXM-LD		4	.6240	.11778	.05889	.4366	.8115	.54	.80	
	LMQXM-MD		4	.7072	.16104	.08052	.4510	.9634	.47	.81	
	LMQXM-HD		4	.6612	.06659	.03329	.5552	.7671	.58	.72	
	Total		24	.6371	.14318	.02923	.5767	.6976	.42	.94	
	model	fixed effect			.12115	.02473	.5852	.6891			

		stochastic effect				.04157	.5303	.7440			.00670
BDNF of PFC	WKY		4	.4865	.05088	.02544	.4055	.5674	.42	.54	
	SHR		4	.3552	.04303	.02152	.2868	.4237	.31	.39	
	MPH		4	.4555	.02668	.01334	.4131	.4980	.43	.48	
	LMQXM-LD		4	.4592	.03796	.01898	.3988	.5196	.42	.51	
	LMQXM-MD		4	.5550	.05874	.02937	.4615	.6485	.50	.62	
	LMQXM-HD		4	.4904	.01607	.00803	.4649	.5160	.47	.51	
	Total		24	.4670	.07112	.01452	.4369	.4970	.31	.62	
	model	fixed effect			.04144	.00846	.4492	.4847			
		stochastic effect				.02668	.3984	.5356			.00384
BDNF of Striatum	WKY		4	.6028	.06162	.03081	.5048	.7009	.54	.66	
	SHR		4	.4525	.06347	.03173	.3515	.5535	.39	.53	
	MPH		4	.6799	.04502	.02251	.6083	.7516	.62	.73	
	LMQXM-LD		4	.5619	.03000	.01500	.5141	.6096	.53	.60	
	LMQXM-MD		4	.6611	.04076	.02038	.5962	.7259	.60	.69	
	LMQXM-HD		4	.6032	.03670	.01835	.5448	.6616	.57	.65	
	Total		24	.5936	.08689	.01774	.5569	.6303	.39	.73	
	model	fixed effect			.04789	.00978	.5730	.6141			
		stochastic effect				.03321	.5082	.6789			.00604

Homogeneity test of variance

		Levin statistics	Degree of freedom 1	Degree of freedom 2	significance
PKA of PFC	Based on the mean	.560	5	18	.729
	Based on the median	.429	5	18	.822
	Based on the median and with the adjusted degrees of freedom	.429	5	9.306	.818
	Based on the post-cut average value	.550	5	18	.736
PKA of Striatum	Based on the mean	1.503	5	18	.238
	Based on the median	1.352	5	18	.288
	Based on the median and with the adjusted degrees of freedom	1.352	5	8.952	.327
	Based on the post-cut average value	1.501	5	18	.239
p-CREB of PFC	Based on the mean	.716	5	18	.619
	Based on the median	.453	5	18	.806

	Based on the median and with the adjusted degrees of freedom	.453	5	13.303	.804
	Based on the post-cut average value	.621	5	18	.686
p-CREB of Striatum	Based on the mean	2.724	5	18	.053
	Based on the median	1.389	5	18	.275
	Based on the median and with the adjusted degrees of freedom	1.389	5	9.692	.309
	Based on the post-cut average value	2.464	5	18	.072
BDNF of PFC	Based on the mean	2.637	5	18	.059
	Based on the median	2.472	5	18	.071
	Based on the median and with the adjusted degrees of freedom	2.472	5	9.614	.108
	Based on the post-cut average value	2.635	5	18	.059
BDNF of Striatum	Based on the mean	1.513	5	18	.235
	Based on the median	1.282	5	18	.315
	Based on the median and with the adjusted degrees of freedom	1.282	5	12.682	.331
	Based on the post-cut average value	1.495	5	18	.240

ANOVA

				quadratic sum	free degree	mean square	F	significance
PKA of PFC	interblock	(assemble)		.096	5	.019	19.386	.000
		linear term	contrast	.021	1	.021	20.835	.000
			bias	.076	4	.019	19.023	.000
	Within the group			.018	18	.001		
	Total			.114	23			
PKA of Striatum	interblock	(assemble)		.046	5	.009	5.073	.004
		linear term	contrast	.014	1	.014	7.792	.012
			bias	.032	4	.008	4.393	.012
	Within the group			.033	18	.002		
	Total			.079	23			
p-CREB of PFC	interblock	(assemble)		.095	5	.019	4.048	.012
		linear term	contrast	.000	1	.000	.060	.809
			bias	.095	4	.024	5.045	.007
	Within the group			.085	18	.005		
	Total			.180	23			
p-CREB of Striatum	interblock	(assemble)		.207	5	.041	2.825	.047
		linear term	contrast	.021	1	.021	1.421	.249
			bias	.186	4	.047	3.176	.039

	Within the group			.264	18	.015		
	Total			.472	23			
BDNF of PFC	interblock	(assemble)		.085	5	.017	9.951	.000
		linear term	contrast	.022	1	.022	12.910	.002
			bias	.063	4	.016	9.211	.000
	Within the group			.031	18	.002		
	Total			.116	23			
BDNF of Striatum	interblock	(assemble)		.132	5	.026	11.542	.000
		linear term	contrast	.015	1	.015	6.465	.020
			bias	.118	4	.029	12.811	.000
	Within the group			.041	18	.002		
	Total			.174	23			

Multiple comparisons

LSD

dependent variable	(I) divide into groups	(J) divide into groups	Mean Difference Value (I-J)	Standard error	significance	And the 95% confidence interval	
						lower limit	superior limit
PKA of PFC	WKY	SHR	.16270*	.02228	.000	.1159	.2095
		MPH	.01866	.02228	.413	-.0281	.0655
		LMQXM-LD	-.00734	.02228	.746	-.0542	.0395
		LMQXM-MD	-.02693	.02228	.243	-.0737	.0199
		LMQXM-HD	-.00136	.02228	.952	-.0482	.0455
	SHR	WKY	-.16270*	.02228	.000	-.2095	-.1159
		MPH	-.14404*	.02228	.000	-.1908	-.0972
		LMQXM-LD	-.17005*	.02228	.000	-.2169	-.1232
		LMQXM-MD	-.18963*	.02228	.000	-.2364	-.1428
		LMQXM-HD	-.16406*	.02228	.000	-.2109	-.1173
	MPH	WKY	-.01866	.02228	.413	-.0655	.0281
		SHR	.14404*	.02228	.000	.0972	.1908
		LMQXM-LD	-.02601	.02228	.258	-.0728	.0208
		LMQXM-MD	-.04559	.02228	.056	-.0924	.0012
		LMQXM-HD	-.02003	.02228	.381	-.0668	.0268
	LMQXM-LD	WKY	.00734	.02228	.746	-.0395	.0542
		SHR	.17005*	.02228	.000	.1232	.2169
		MPH	.02601	.02228	.258	-.0208	.0728
		LMQXM-MD	-.01958	.02228	.391	-.0664	.0272
		LMQXM-HD	.00598	.02228	.791	-.0408	.0528
	LMQXM-MD	WKY	.02693	.02228	.243	-.0199	.0737
		SHR	.18963*	.02228	.000	.1428	.2364
		MPH	.04559	.02228	.056	-.0012	.0924
		LMQXM-LD	.01958	.02228	.391	-.0272	.0664

		LMQXM-HD	.02556	.02228	.266	-.0212	.0724
	LMQXM-HD	WKY	.00136	.02228	.952	-.0455	.0482
		SHR	.16406*	.02228	.000	.1173	.2109
		MPH	.02003	.02228	.381	-.0268	.0668
		LMQXM-LD	-.00598	.02228	.791	-.0528	.0408
		LMQXM-MD	-.02556	.02228	.266	-.0724	.0212
PKA of Striatum	WKY	SHR	.08297*	.03027	.013	.0194	.1466
		MPH	-.02864	.03027	.357	-.0922	.0349
		LMQXM-LD	.00263	.03027	.932	-.0610	.0662
		LMQXM-MD	-.05978	.03027	.064	-.1234	.0038
		LMQXM-HD	-.02057	.03027	.505	-.0842	.0430
	SHR	WKY	-.08297*	.03027	.013	-.1466	-.0194
		MPH	-.11161*	.03027	.002	-.1752	-.0480
		LMQXM-LD	-.08034*	.03027	.016	-.1439	-.0168
		LMQXM-MD	-.14275*	.03027	.000	-.2063	-.0792
		LMQXM-HD	-.10353*	.03027	.003	-.1671	-.0399
	MPH	WKY	.02864	.03027	.357	-.0349	.0922
		SHR	.11161*	.03027	.002	.0480	.1752
		LMQXM-LD	.03127	.03027	.315	-.0323	.0949
		LMQXM-MD	-.03114	.03027	.317	-.0947	.0324
		LMQXM-HD	.00807	.03027	.793	-.0555	.0717
	LMQXM-LD	WKY	-.00263	.03027	.932	-.0662	.0610
		SHR	.08034*	.03027	.016	.0168	.1439
		MPH	-.03127	.03027	.315	-.0949	.0323
		LMQXM-MD	-.06241	.03027	.054	-.1260	.0012
		LMQXM-HD	-.02320	.03027	.453	-.0868	.0404
	LMQXM-MD	WKY	.05978	.03027	.064	-.0038	.1234
		SHR	.14275*	.03027	.000	.0792	.2063
		MPH	.03114	.03027	.317	-.0324	.0947
		LMQXM-LD	.06241	.03027	.054	-.0012	.1260
		LMQXM-HD	.03922	.03027	.211	-.0244	.1028
	LMQXM-HD	WKY	.02057	.03027	.505	-.0430	.0842
		SHR	.10353*	.03027	.003	.0399	.1671
		MPH	-.00807	.03027	.793	-.0717	.0555
		LMQXM-LD	.02320	.03027	.453	-.0404	.0868
		LMQXM-MD	-.03922	.03027	.211	-.1028	.0244
p-CREB of PFC	WKY	SHR	.15211*	.04845	.006	.0503	.2539
		MPH	.04227	.04845	.395	-.0595	.1441
		LMQXM-LD	.10574*	.04845	.043	.0039	.2075

		LMQXM-MD	-.02439	.04845	.621	-.1262	.0774
		LMQXM-HD	.10727*	.04845	.040	.0055	.2091
	SHR	WKY	-.15211*	.04845	.006	-.2539	-.0503
		MPH	-.10984*	.04845	.036	-.2116	-.0080
		LMQXM-LD	-.04637	.04845	.351	-.1482	.0554
		LMQXM-MD	-.17650*	.04845	.002	-.2783	-.0747
		LMQXM-HD	-.04484	.04845	.367	-.1466	.0570
	MPH	WKY	-.04227	.04845	.395	-.1441	.0595
		SHR	.10984*	.04845	.036	.0080	.2116
		LMQXM-LD	.06347	.04845	.207	-.0383	.1653
		LMQXM-MD	-.06666	.04845	.186	-.1685	.0351
		LMQXM-HD	.06500	.04845	.196	-.0368	.1668
	LMQXM-LD	WKY	-.10574*	.04845	.043	-.2075	-.0039
		SHR	.04637	.04845	.351	-.0554	.1482
		MPH	-.06347	.04845	.207	-.1653	.0383
		LMQXM-MD	-.13013*	.04845	.015	-.2319	-.0283
		LMQXM-HD	.00153	.04845	.975	-.1003	.1033
	LMQXM-MD	WKY	.02439	.04845	.621	-.0774	.1262
		SHR	.17650*	.04845	.002	.0747	.2783
		MPH	.06666	.04845	.186	-.0351	.1685
		LMQXM-LD	.13013*	.04845	.015	.0283	.2319
		LMQXM-HD	.13166*	.04845	.014	.0299	.2335
	LMQXM-HD	WKY	-.10727*	.04845	.040	-.2091	-.0055
		SHR	.04484	.04845	.367	-.0570	.1466
		MPH	-.06500	.04845	.196	-.1668	.0368
		LMQXM-LD	-.00153	.04845	.975	-.1033	.1003
		LMQXM-MD	-.13166*	.04845	.014	-.2335	-.0299
p-CREB of Striatum	WKY	SHR	.24517*	.08567	.010	.0652	.4251
		MPH	-.02197	.08567	.800	-.2019	.1580
		LMQXM-LD	.06049	.08567	.489	-.1195	.2405
		LMQXM-MD	-.02266	.08567	.794	-.2026	.1573
		LMQXM-HD	.02336	.08567	.788	-.1566	.2033
	SHR	WKY	-.24517*	.08567	.010	-.4251	-.0652
		MPH	-.26714*	.08567	.006	-.4471	-.0872
		LMQXM-LD	-.18468*	.08567	.045	-.3647	-.0047
		LMQXM-MD	-.26783*	.08567	.006	-.4478	-.0879
		LMQXM-HD	-.22181*	.08567	.019	-.4018	-.0418
	MPH	WKY	.02197	.08567	.800	-.1580	.2019
		SHR	.26714*	.08567	.006	.0872	.4471
		LMQXM-LD	.08246	.08567	.348	-.0975	.2624

		LMQXM-MD	-.00069	.08567	.994	-.1807	.1793
		LMQXM-HD	.04533	.08567	.603	-.1346	.2253
	LMQXM-LD	WKY	-.06049	.08567	.489	-.2405	.1195
		SHR	.18468*	.08567	.045	.0047	.3647
		MPH	-.08246	.08567	.348	-.2624	.0975
		LMQXM-MD	-.08315	.08567	.345	-.2631	.0968
		LMQXM-HD	-.03713	.08567	.670	-.2171	.1428
	LMQXM-MD	WKY	.02266	.08567	.794	-.1573	.2026
		SHR	.26783*	.08567	.006	.0879	.4478
		MPH	.00069	.08567	.994	-.1793	.1807
		LMQXM-LD	.08315	.08567	.345	-.0968	.2631
		LMQXM-HD	.04602	.08567	.598	-.1340	.2260
	LMQXM-HD	WKY	-.02336	.08567	.788	-.2033	.1566
		SHR	.22181*	.08567	.019	.0418	.4018
		MPH	-.04533	.08567	.603	-.2253	.1346
		LMQXM-LD	.03713	.08567	.670	-.1428	.2171
		LMQXM-MD	-.04602	.08567	.598	-.2260	.1340
BDNF of PFC	WKY	SHR	.13122*	.02930	.000	.0697	.1928
		MPH	.03095	.02930	.305	-.0306	.0925
		LMQXM-LD	.02724	.02930	.365	-.0343	.0888
		LMQXM-MD	-.06855*	.02930	.031	-.1301	-.0070
		LMQXM-HD	-.00396	.02930	.894	-.0655	.0576
	SHR	WKY	-.13122*	.02930	.000	-.1928	-.0697
		MPH	-.10027*	.02930	.003	-.1618	-.0387
		LMQXM-LD	-.10398*	.02930	.002	-.1655	-.0424
		LMQXM-MD	-.19977*	.02930	.000	-.2613	-.1382
		LMQXM-HD	-.13518*	.02930	.000	-.1967	-.0736
	MPH	WKY	-.03095	.02930	.305	-.0925	.0306
		SHR	.10027*	.02930	.003	.0387	.1618
		LMQXM-LD	-.00371	.02930	.901	-.0653	.0579
		LMQXM-MD	-.09950*	.02930	.003	-.1611	-.0379
		LMQXM-HD	-.03491	.02930	.249	-.0965	.0266
	LMQXM-LD	WKY	-.02724	.02930	.365	-.0888	.0343
		SHR	.10398*	.02930	.002	.0424	.1655
		MPH	.00371	.02930	.901	-.0579	.0653
		LMQXM-MD	-.09579*	.02930	.004	-.1573	-.0342
		LMQXM-HD	-.03120	.02930	.301	-.0928	.0304
	LMQXM-MD	WKY	.06855*	.02930	.031	.0070	.1301
		SHR	.19977*	.02930	.000	.1382	.2613

		MPH	.09950*	.02930	.003	.0379	.1611
		LMQXM-LD	.09579*	.02930	.004	.0342	.1573
		LMQXM-HD	.06459*	.02930	.041	.0030	.1261
	LMQXM-HD	WKY	.00396	.02930	.894	-.0576	.0655
		SHR	.13518*	.02930	.000	.0736	.1967
		MPH	.03491	.02930	.249	-.0266	.0965
		LMQXM-LD	.03120	.02930	.301	-.0304	.0928
		LMQXM-MD	-.06459*	.02930	.041	-.1261	-.0030
BDNF of Striatum	WKY	SHR	.15029*	.03386	.000	.0791	.2214
		MPH	-.07709*	.03386	.035	-.1482	-.0059
		LMQXM-LD	.04095	.03386	.242	-.0302	.1121
		LMQXM-MD	-.05825	.03386	.103	-.1294	.0129
		LMQXM-HD	-.00036	.03386	.992	-.0715	.0708
	SHR	WKY	-.15029*	.03386	.000	-.2214	-.0791
		MPH	-.22738*	.03386	.000	-.2985	-.1562
		LMQXM-LD	-.10934*	.03386	.005	-.1805	-.0382
		LMQXM-MD	-.20854*	.03386	.000	-.2797	-.1374
		LMQXM-HD	-.15065*	.03386	.000	-.2218	-.0795
	MPH	WKY	.07709*	.03386	.035	.0059	.1482
		SHR	.22738*	.03386	.000	.1562	.2985
		LMQXM-LD	.11804*	.03386	.003	.0469	.1892
		LMQXM-MD	.01884	.03386	.585	-.0523	.0900
		LMQXM-HD	.07673*	.03386	.036	.0056	.1479
	LMQXM-LD	WKY	-.04095	.03386	.242	-.1121	.0302
		SHR	.10934*	.03386	.005	.0382	.1805
		MPH	-.11804*	.03386	.003	-.1892	-.0469
		LMQXM-MD	-.09920*	.03386	.009	-.1703	-.0281
		LMQXM-HD	-.04131	.03386	.238	-.1125	.0298
	LMQXM-MD	WKY	.05825	.03386	.103	-.0129	.1294
		SHR	.20854*	.03386	.000	.1374	.2797
		MPH	-.01884	.03386	.585	-.0900	.0523
		LMQXM-LD	.09920*	.03386	.009	.0281	.1703
		LMQXM-HD	.05789	.03386	.105	-.0133	.1290
	LMQXM-HD	WKY	.00036	.03386	.992	-.0708	.0715
		SHR	.15065*	.03386	.000	.0795	.2218
		MPH	-.07673*	.03386	.036	-.1479	-.0056
		LMQXM-LD	.04131	.03386	.238	-.0298	.1125
		LMQXM-MD	-.05789	.03386	.105	-.1290	.0133

Supplementary Table 21 Effects of LMQXM on the relative expression of DRD1 and PKA proteins in rat PFC and Striatum（$\bar{x} \pm s$，n=3）

Group	DRD1/GAPDH		PKA/GAPDH	
	PFC	Striatum	PFC	Striatum
WKY	0.90±0.088	0.82±0.22	0.89±0.10	0.64±0.12
SHR	0.18±0.05 $^{\#\#\#}$	0.12±0.06 $^{\#\#}$	0.21±0.05 $^{\#\#\#}$	0.27±0.10 $^{\#}$
MPH	0.60±0.07 ***	0.57±0.20 *	0.91±0.10 ***	0.90±0.26 ***
LMQXM-LD	0.40±0.08 $^{**\wedge}$	0.43±0.18 $^{\wedge}$	0.56±0.04 $^{***\wedge}$	0.57±0.15 *
LMQXM-MD	0.73±0.06 ***	0.81±0.24 **	0.72±0.03 ***	0.67±0.17 ***
LMQXM-HD	0.29±0.09 $^{\wedge}$	0.30±0.16 $^{\wedge}$	0.30±0.05 $^{\wedge}$	0.48±0.10
F -value	42.279	6.908	54.377	5.259
P- value	＜0.001	0.003	＜0.001	0.009

Note: Compared with the WKY group, $^{\#}P < 0.05$, $^{\#\#}P < 0.01$, $^{\#\#\#}P < 0.001$; compared with the SHR group, $^{*}P < 0.05$, $^{**}P < 0.01$, $^{***}P < 0.001$; compared with the LMQXM-MD group, $^{\wedge}P < 0.05$.

Supplementary Table 22 Effects of LMQXM on the relative expression of Gαs and Gαolf proteins in rat PFC and Striatum（$\bar{x} \pm s$，n=3）

Group	Gαs/GAPDH		Gαolf/GAPDH	
	PFC	Striatum	PFC	Striatum
WKY	0.84±0.14	0.79±0.06	0.45±0.11	0.54±0.12
SHR	0.17±0.03 $^{\#\#\#}$	0.15±0.05 $^{\#\#\#}$	0.15±0.03 $^{\#\#\#}$	0.20±0.12 $^{\#\#}$
MPH	0.63±0.12 ***	0.53±0.05 ***	0.60±0.11 ***	0.78±0.16 ***
LMQXM-LD	0.30±0.04 $^{\wedge}$	0.44±0.08 *** $^{\wedge}$	0.33±0.01 $^{*\wedge}$	0.45±0.13 *
LMQXM-MD	0.84±0.16 ***	0.65±0.06 ***	0.75±0.12 ***	0.56±0.14 **
LMQXM-HD	0.45±0.07 $^{**\wedge}$	0.33±0.04 $^{**\wedge}$	0.32±0.02 $^{*\wedge}$	0.44±0.14
F -value	21.277	46.477	21.674	5.735
P- value	＜0.001	＜0.001	＜0.001	0.006

Note: Compared with the WKY group, $^{\#}P < 0.05$, $^{\#\#}P < 0.01$, $^{\#\#\#}P < 0.001$; compared with the SHR group, $^{*}P < 0.05$, $^{**}P < 0.01$, $^{***}P < 0.001$; compared with the LMQXM-MD group, $^{\wedge}P < 0.05$.

Supplementary Table 23 Effects of LMQXM on the relative expression of p-CREB and BDNF proteins in rat PFC and Striatum（$\bar{x} \pm s$，n=3）

Group	p-CREB/GAPDH		BDNF/GAPDH	
	PFC	Striatum	PFC	Striatum

WKY	0.20±0.04	0.52±0.16	0.38±0.09	0.38±0.10
SHR	0.04±0.02 $^{\#\#\#}$	0.10±0.04 $^{\#\#}$	0.12±0.05 $^{\#\#}$	0.13±0.04 $^{\#\#}$
MPH	0.38±0.05 ***	0.32±0.11 *	0.51±0.11 ***	0.42±0.16 **
LMQXM-LD	0.13±0.04 $^{**\wedge}$	0.20±0.04 $^{\wedge}$	0.22±0.064 $^{\wedge}$	0.22±0.06 $^{\wedge}$
LMQXM-MD	0.21±0.02 ***	0.42±0.15 **	0.51±0.08 ***	0.50±0.11 **
LMQXM-HD	0.05±0.02 $^{\wedge}$	0.23±0.09	0.22±0.06 $^{\wedge}$	0.32±0.05 $^{*\wedge}$
F -value	39.978	5.737	13.777	5.872
P- value	<0.001	0.006	<0.001	0.006

Note: Compared with the WKY group, $^{\#}P < 0.05$, $^{\#\#}P < 0.01$, $^{\#\#\#}P < 0.001$; compared with the SHR group, $^{*}P < 0.05$, $^{**}P < 0.01$, $^{***}P < 0.001$; compared with the LMQXM-MD group, $^{\wedge}P < 0.05$.

Supplementary Table 24 One-ANOVA of the relative expression of DRD1, Gαs, Gαolf, PKA, p-CREB, and BDNF proteins in rat PFC and Striatum.

**Homogeneity test of variance**					
		Levin statistics	Degree of freedom 1	Degree of freedom 2	significance
DRD1 of PFC	Based on the mean	.401	5	12	.839
	Based on the median	.093	5	12	.992
	Based on the median and with the adjusted degrees of freedom	.093	5	7.729	.991
	Based on the post-cut average value	.371	5	12	.859
G α s of PFC	Based on the mean	3.245	5	12	.044
	Based on the median	.386	5	12	.849
	Based on the median and with the adjusted degrees of freedom	.386	5	6.074	.842
	Based on the post-cut average value	2.778	5	12	.068
G α olf of PFC	Based on the mean	2.730	5	12	.072
	Based on the median	1.205	5	12	.364
	Based on the median and with the adjusted degrees of freedom	1.205	5	6.314	.403
	Based on the post-cut average value	2.611	5	12	.080
PKA OF PFC	Based on the mean	2.287	5	12	.112
	Based on the median	.402	5	12	.839
	Based on the median and with the adjusted degrees of freedom	.402	5	8.027	.835

	Based on the post-cut average value	2.028	5	12	.147
p-CREB of PFC	Based on the mean	2.590	5	12	.082
	Based on the median	.310	5	12	.898
	Based on the median and with the adjusted degrees of freedom	.310	5	6.806	.892
	Based on the post-cut average value	2.227	5	12	.119
BDNF OF PFC	Based on the mean	.998	5	12	.459
	Based on the median	.177	5	12	.966
	Based on the median and with the adjusted degrees of freedom	.177	5	8.724	.964
	Based on the post-cut average value	.885	5	12	.520
DRD1 of Striatum	Based on the mean	.794	5	12	.574
	Based on the median	.376	5	12	.856
	Based on the median and with the adjusted degrees of freedom	.376	5	9.742	.854
	Based on the post-cut average value	.762	5	12	.594
Gαs of Striatum	Based on the mean	.335	5	12	.882
	Based on the median	.089	5	12	.992
	Based on the median and with the adjusted degrees of freedom	.089	5	10.946	.992
	Based on the post-cut average value	.306	5	12	.900
Gαolf of Striatum	Based on the mean	.128	5	12	.983
	Based on the median	.027	5	12	1.000
	Based on the median and with the adjusted degrees of freedom	.027	5	10.495	1.000
	Based on the post-cut average value	.113	5	12	.987
PKA of Striatum	Based on the mean	1.879	5	12	.172
	Based on the median	.152	5	12	.975
	Based on the median and with the adjusted degrees of freedom	.152	5	5.557	.971
	Based on the post-cut average value	1.589	5	12	.237
p-CREB of Striatum	Based on the mean	3.192	5	12	.046
	Based on the median	.321	5	12	.891

	Based on the median and with the adjusted degrees of freedom	.321	5	7.866	.887
	Based on the post-cut average value	2.665	5	12	.076
BDNF of Striatum	Based on the mean	1.581	5	12	.239
	Based on the median	.629	5	12	.681
	Based on the median and with the adjusted degrees of freedom	.629	5	7.381	.684
	Based on the post-cut average value	1.503	5	12	.260

ANOVA

				quadratic sum	free degree	mean square	F	significance
DRD1 OF PFC	interblock	(assemble)		1.141	5	.228	42.279	.000
		linear term	contrast	.109	1	.109	20.224	.001
			bias	1.032	4	.258	47.793	.000
	Within the group			.065	12	.005		
	Total			1.206	17			
G α s of PFC	interblock	(assemble)		1.196	5	.239	21.277	.000
		linear term	contrast	.003	1	.003	.256	.622
			bias	1.193	4	.298	26.532	.000
	Within the group			.135	12	.011		
	Total			1.331	17			
G α olf of PFC	interblock	(assemble)		.699	5	.140	21.674	.000
		linear term	contrast	.027	1	.027	4.146	.064
			bias	.672	4	.168	26.056	.000
	Within the group			.077	12	.006		
	Total			.776	17			
PKA OF PFC	interblock	(assemble)		1.314	5	.263	54.377	.000
		linear term	contrast	.133	1	.133	27.490	.000
			bias	1.181	4	.295	61.098	.000
	Within the group			.058	12	.005		
	Total			1.372	17			
p-CREB of PFC	interblock	(assemble)		.238	5	.048	39.978	.000
		linear term	contrast	.012	1	.012	9.985	.008
			bias	.226	4	.057	47.477	.000
	Within the group			.014	12	.001		
	Total			.253	17			
BDNF OF PFC	interblock	(assemble)		.418	5	.084	13.777	.000
		linear	contrast	.000	1	.000	.020	.889

		term	bias	.417	4	.104	17.216	.000
	Within the group			.073	12	.006		
	Total			.490	17			
DRD1 of Striatum	interblock	(assemble)		1.192	5	.238	6.908	.003
		linear	contrast	.019	1	.019	.552	.472
		term	bias	1.173	4	.293	8.497	.002
	Within the group			.414	12	.035		
	Total			1.606	17			
Gαs of Striatum	interblock	(assemble)		.770	5	.154	46.477	.000
		linear	contrast	.034	1	.034	10.309	.007
		term	bias	.736	4	.184	55.519	.000
	Within the group			.040	12	.003		
	Total			.810	17			
Gαolf of Striatum	interblock	(assemble)		.534	5	.107	5.735	.006
		linear	contrast	.002	1	.002	.100	.757
		term	bias	.532	4	.133	7.143	.003
	Within the group			.224	12	.019		
	Total			.758	17			
PKA of Striatum	interblock	(assemble)		.674	5	.135	5.259	.009
		linear	contrast	.000	1	.000	.010	.921
		term	bias	.674	4	.168	6.572	.005
	Within the group			.308	12	.026		
	Total			.982	17			
p-CREB of Striatum	interblock	(assemble)		.348	5	.070	5.737	.006
		linear	contrast	.013	1	.013	1.092	.317
		term	bias	.335	4	.084	6.899	.004
	Within the group			.145	12	.012		
	Total			.493	17			
BDNF of Striatum	interblock	(assemble)		.275	5	.055	5.872	.006
		linear	contrast	.015	1	.015	1.627	.226
		term	bias	.260	4	.065	6.933	.004
	Within the group			.112	12	.009		
	Total			.387	17			

Multiple comparisons

dependent variable		(I) divide into groups	(J) divide into groups	Mean Difference Value (I-J)	Standard error	significance	And the 95% confidence interval lower limit	superior limit
DRD1 of PFC	LSD	WKY	SHR	.72290*	.05999	.000	.5922	.8536

			MPH	.30093*	.05999	.000	.1702	.4316
			LMQXM-LD	.50012*	.05999	.000	.3694	.6308
			LMQXM-MD	.16953*	.05999	.015	.0388	.3002
			LMQXM-HD	.61141*	.05999	.000	.4807	.7421
		SHR	WKY	-.72290*	.05999	.000	-.8536	-.5922
			MPH	-.42198*	.05999	.000	-.5527	-.2913
			LMQXM-LD	-.22278*	.05999	.003	-.3535	-.0921
			LMQXM-MD	-.55338*	.05999	.000	-.6841	-.4227
			LMQXM-HD	-.11149	.05999	.088	-.2422	.0192
		MPH	WKY	-.30093*	.05999	.000	-.4316	-.1702
			SHR	.42198*	.05999	.000	.2913	.5527
			LMQXM-LD	.19920*	.05999	.006	.0685	.3299
			LMQXM-MD	-.13140*	.05999	.049	-.2621	-.0007
			LMQXM-HD	.31048*	.05999	.000	.1798	.4412
		LMQXM-LD	WKY	-.50012*	.05999	.000	-.6308	-.3694
			SHR	.22278*	.05999	.003	.0921	.3535
			MPH	-.19920*	.05999	.006	-.3299	-.0685
			LMQXM-MD	-.33060*	.05999	.000	-.4613	-.1999
			LMQXM-HD	.11129	.05999	.088	-.0194	.2420
		LMQXM-MD	WKY	-.16953*	.05999	.015	-.3002	-.0388
			SHR	.55338*	.05999	.000	.4227	.6841
			MPH	.13140*	.05999	.049	.0007	.2621
			LMQXM-LD	.33060*	.05999	.000	.1999	.4613
			LMQXM-HD	.44189*	.05999	.000	.3112	.5726
		LMQXM-HD	WKY	-.61141*	.05999	.000	-.7421	-.4807
			SHR	.11149	.05999	.088	-.0192	.2422
			MPH	-.31048*	.05999	.000	-.4412	-.1798
			LMQXM-LD	-.11129	.05999	.088	-.2420	.0194
			LMQXM-MD	-.44189*	.05999	.000	-.5726	-.3112
Gαs of PFC	LSD	WKY	SHR	.67528*	.08657	.000	.4867	.8639
			MPH	.21644*	.08657	.028	.0278	.4051
			LMQXM-LD	.54588*	.08657	.000	.3573	.7345
			LMQXM-MD	-.00304	.08657	.973	-.1917	.1856
			LMQXM-HD	.39298*	.08657	.001	.2044	.5816
		SHR	WKY	-.67528*	.08657	.000	-.8639	-.4867
			MPH	-.45885*	.08657	.000	-.6475	-.2702
			LMQXM-LD	-.12940	.08657	.161	-.3180	.0592
			LMQXM-MD	-.67832*	.08657	.000	-.8669	-.4897
			LMQXM-HD	-.28231*	.08657	.007	-.4709	-.0937
		MPH	WKY	-.21644*	.08657	.028	-.4051	-.0278

			SHR	.45885*	.08657	.000	.2702	.6475
			LMQXM-LD	.32944*	.08657	.003	.1408	.5181
			LMQXM-MD	-.21948*	.08657	.026	-.4081	-.0309
			LMQXM-HD	.17654	.08657	.064	-.0121	.3652
		LMQXM-LD	WKY	-.54588*	.08657	.000	-.7345	-.3573
			SHR	.12940	.08657	.161	-.0592	.3180
			MPH	-.32944*	.08657	.003	-.5181	-.1408
			LMQXM-MD	-.54892*	.08657	.000	-.7375	-.3603
			LMQXM-HD	-.15290	.08657	.103	-.3415	.0357
		LMQXM-MD	WKY	.00304	.08657	.973	-.1856	.1917
			SHR	.67832*	.08657	.000	.4897	.8669
			MPH	.21948*	.08657	.026	.0309	.4081
			LMQXM-LD	.54892*	.08657	.000	.3603	.7375
			LMQXM-HD	.39602*	.08657	.001	.2074	.5846
		LMQXM-HD	WKY	-.39298*	.08657	.001	-.5816	-.2044
			SHR	.28231*	.08657	.007	.0937	.4709
			MPH	-.17654	.08657	.064	-.3652	.0121
			LMQXM-LD	.15290	.08657	.103	-.0357	.3415
			LMQXM-MD	-.39602*	.08657	.001	-.5846	-.2074
Gαolf of PFC	LSD	WKY	SHR	.32041*	.06556	.000	.1776	.4633
			MPH	-.13302	.06556	.065	-.2759	.0098
			LMQXM-LD	.14073	.06556	.053	-.0021	.2836
			LMQXM-MD	-.27863*	.06556	.001	-.4215	-.1358
			LMQXM-HD	.14671*	.06556	.045	.0039	.2896
		SHR	WKY	-.32041*	.06556	.000	-.4633	-.1776
			MPH	-.45343*	.06556	.000	-.5963	-.3106
			LMQXM-LD	-.17968*	.06556	.018	-.3225	-.0368
			LMQXM-MD	-.59904*	.06556	.000	-.7419	-.4562
			LMQXM-HD	-.17370*	.06556	.021	-.3165	-.0309
		MPH	WKY	.13302	.06556	.065	-.0098	.2759
			SHR	.45343*	.06556	.000	.3106	.5963
			LMQXM-LD	.27375*	.06556	.001	.1309	.4166
			LMQXM-MD	-.14561*	.06556	.046	-.2885	-.0028
			LMQXM-HD	.27973*	.06556	.001	.1369	.4226
		LMQXM-LD	WKY	-.14073	.06556	.053	-.2836	.0021
			SHR	.17968*	.06556	.018	.0368	.3225
			MPH	-.27375*	.06556	.001	-.4166	-.1309
			LMQXM-MD	-.41937*	.06556	.000	-.5622	-.2765
			LMQXM-HD	.00598	.06556	.929	-.1369	.1488

		LMQXM-MD	WKY	.27863*	.06556	.001	.1358	.4215
			SHR	.59904*	.06556	.000	.4562	.7419
			MPH	.14561*	.06556	.046	.0028	.2885
			LMQXM-LD	.41937*	.06556	.000	.2765	.5622
			LMQXM-HD	.42534*	.06556	.000	.2825	.5682
		LMQXM-HD	WKY	-.14671*	.06556	.045	-.2896	-.0039
			SHR	.17370*	.06556	.021	.0309	.3165
			MPH	-.27973*	.06556	.001	-.4226	-.1369
			LMQXM-LD	-.00598	.06556	.929	-.1488	.1369
			LMQXM-MD	-.42534*	.06556	.000	-.5682	-.2825
PKA of PFC	LSD	WKY	SHR	.68526*	.05676	.000	.5616	.8089
			MPH	-.01713	.05676	.768	-.1408	.1065
			LMQXM-LD	.33656*	.05676	.000	.2129	.4602
			LMQXM-MD	.17594*	.05676	.009	.0523	.2996
			LMQXM-HD	.58695*	.05676	.000	.4633	.7106
		SHR	WKY	-.68526*	.05676	.000	-.8089	-.5616
			MPH	-.70240*	.05676	.000	-.8261	-.5787
			LMQXM-LD	-.34871*	.05676	.000	-.4724	-.2250
			LMQXM-MD	-.50932*	.05676	.000	-.6330	-.3857
			LMQXM-HD	-.09831	.05676	.109	-.2220	.0253
		MPH	WKY	.01713	.05676	.768	-.1065	.1408
			SHR	.70240*	.05676	.000	.5787	.8261
			LMQXM-LD	.35369*	.05676	.000	.2300	.4773
			LMQXM-MD	.19307*	.05676	.005	.0694	.3167
			LMQXM-HD	.60408*	.05676	.000	.4804	.7277
		LMQXM-LD	WKY	-.33656*	.05676	.000	-.4602	-.2129
			SHR	.34871*	.05676	.000	.2250	.4724
			MPH	-.35369*	.05676	.000	-.4773	-.2300
			LMQXM-MD	-.16062*	.05676	.015	-.2843	-.0370
			LMQXM-HD	.25039*	.05676	.001	.1267	.3741
		LMQXM-MD	WKY	-.17594*	.05676	.009	-.2996	-.0523
			SHR	.50932*	.05676	.000	.3857	.6330
			MPH	-.19307*	.05676	.005	-.3167	-.0694
			LMQXM-LD	.16062*	.05676	.015	.0370	.2843
			LMQXM-HD	.41101*	.05676	.000	.2873	.5347
		LMQXM-HD	WKY	-.58695*	.05676	.000	-.7106	-.4633
			SHR	.09831	.05676	.109	-.0253	.2220
			MPH	-.60408*	.05676	.000	-.7277	-.4804
			LMQXM-LD	-.25039*	.05676	.001	-.3741	-.1267
			LMQXM-MD	-.41101*	.05676	.000	-.5347	-.2873

p-CREB of PFC	LSD	WKY	SHR	.15666*	.02819	.000	.0952	.2181
			MPH	-.18207*	.02819	.000	-.2435	-.1207
			LMQXM-LD	.06947*	.02819	.030	.0080	.1309
			LMQXM-MD	-.00431	.02819	.881	-.0657	.0571
			LMQXM-HD	.15167*	.02819	.000	.0902	.2131
		SHR	WKY	-.15666*	.02819	.000	-.2181	-.0952
			MPH	-.33873*	.02819	.000	-.4002	-.2773
			LMQXM-LD	-.08718*	.02819	.009	-.1486	-.0258
			LMQXM-MD	-.16096*	.02819	.000	-.2224	-.0995
			LMQXM-HD	-.00498	.02819	.863	-.0664	.0564
		MPH	WKY	.18207*	.02819	.000	.1207	.2435
			SHR	.33873*	.02819	.000	.2773	.4002
			LMQXM-LD	.25155*	.02819	.000	.1901	.3130
			LMQXM-MD	.17777*	.02819	.000	.1163	.2392
			LMQXM-HD	.33375*	.02819	.000	.2723	.3952
		LMQXM-LD	WKY	-.06947*	.02819	.030	-.1309	-.0080
			SHR	.08718*	.02819	.009	.0258	.1486
			MPH	-.25155*	.02819	.000	-.3130	-.1901
			LMQXM-MD	-.07378*	.02819	.023	-.1352	-.0124
			LMQXM-HD	.08220*	.02819	.013	.0208	.1436
		LMQXM-MD	WKY	.00431	.02819	.881	-.0571	.0657
			SHR	.16096*	.02819	.000	.0995	.2224
			MPH	-.17777*	.02819	.000	-.2392	-.1163
			LMQXM-LD	.07378*	.02819	.023	.0124	.1352
			LMQXM-HD	.15598*	.02819	.000	.0946	.2174
		LMQXM-HD	WKY	-.15167*	.02819	.000	-.2131	-.0902
			SHR	.00498	.02819	.863	-.0564	.0664
			MPH	-.33375*	.02819	.000	-.3952	-.2723
			LMQXM-LD	-.08220*	.02819	.013	-.1436	-.0208
			LMQXM-MD	-.15598*	.02819	.000	-.2174	-.0946
BDNF OF PFC	LSD	WKY	SHR	.26266*	.06357	.001	.1242	.4012
			MPH	-.13358	.06357	.057	-.2721	.0049
			LMQXM-LD	.16217*	.06357	.025	.0237	.3007
			LMQXM-MD	-.12902	.06357	.065	-.2675	.0095
			LMQXM-HD	.16515*	.06357	.023	.0266	.3037
		SHR	WKY	-.26266*	.06357	.001	-.4012	-.1242
			MPH	-.39625*	.06357	.000	-.5348	-.2577
			LMQXM-LD	-.10050	.06357	.140	-.2390	.0380
			LMQXM-MD	-.39168*	.06357	.000	-.5302	-.2532

			LMQXM-HD	-.09751	.06357	.151	-.2360	.0410
		MPH	WKY	.13358	.06357	.057	-.0049	.2721
			SHR	.39625*	.06357	.000	.2577	.5348
			LMQXM-LD	.29575*	.06357	.001	.1572	.4343
			LMQXM-MD	.00456	.06357	.944	-.1339	.1431
			LMQXM-HD	.29873*	.06357	.001	.1602	.4372
		LMQXM-LD	WKY	-.16217*	.06357	.025	-.3007	-.0237
			SHR	.10050	.06357	.140	-.0380	.2390
			MPH	-.29575*	.06357	.001	-.4343	-.1572
			LMQXM-MD	-.29119*	.06357	.001	-.4297	-.1527
			LMQXM-HD	.00298	.06357	.963	-.1355	.1415
		LMQXM-MD	WKY	.12902	.06357	.065	-.0095	.2675
			SHR	.39168*	.06357	.000	.2532	.5302
			MPH	-.00456	.06357	.944	-.1431	.1339
			LMQXM-LD	.29119*	.06357	.001	.1527	.4297
			LMQXM-HD	.29417*	.06357	.001	.1557	.4327
		LMQXM-HD	WKY	-.16515*	.06357	.023	-.3037	-.0266
			SHR	.09751	.06357	.151	-.0410	.2360
			MPH	-.29873*	.06357	.001	-.4372	-.1602
			LMQXM-LD	-.00298	.06357	.963	-.1415	.1355
			LMQXM-MD	-.29417*	.06357	.001	-.4327	-.1557
DRD1 of Striatum	LSD	WKY	SHR	.70481*	.15170	.001	.3743	1.0353
			MPH	.25237	.15170	.122	-.0782	.5829
			LMQXM-LD	.39616*	.15170	.023	.0656	.7267
			LMQXM-MD	.00993	.15170	.949	-.3206	.3404
			LMQXM-HD	.52155*	.15170	.005	.1910	.8521
		SHR	WKY	-.70481*	.15170	.001	-1.0353	-.3743
			MPH	-.45245*	.15170	.011	-.7830	-.1219
			LMQXM-LD	-.30865	.15170	.065	-.6392	.0219
			LMQXM-MD	-.69489*	.15170	.001	-1.0254	-.3644
			LMQXM-HD	-.18326	.15170	.250	-.5138	.1473
		MPH	WKY	-.25237	.15170	.122	-.5829	.0782
			SHR	.45245*	.15170	.011	.1219	.7830
			LMQXM-LD	.14380	.15170	.362	-.1867	.4743
			LMQXM-MD	-.24244	.15170	.136	-.5730	.0881
			LMQXM-HD	.26919	.15170	.101	-.0613	.5997
		LMQXM-LD	WKY	-.39616*	.15170	.023	-.7267	-.0656
			SHR	.30865	.15170	.065	-.0219	.6392
			MPH	-.14380	.15170	.362	-.4743	.1867
			LMQXM-MD	-.38624*	.15170	.026	-.7168	-.0557

			LMQXM-HD	.12539	.15170	.425	-.2051	.4559
		LMQXM-MD	WKY	-.00993	.15170	.949	-.3404	.3206
			SHR	.69489*	.15170	.001	.3644	1.0254
			MPH	.24244	.15170	.136	-.0881	.5730
			LMQXM-LD	.38624*	.15170	.026	.0557	.7168
			LMQXM-HD	.51162*	.15170	.006	.1811	.8421
		LMQXM-HD	WKY	-.52155*	.15170	.005	-.8521	-.1910
			SHR	.18326	.15170	.250	-.1473	.5138
			MPH	-.26919	.15170	.101	-.5997	.0613
			LMQXM-LD	-.12539	.15170	.425	-.4559	.2051
			LMQXM-MD	-.51162*	.15170	.006	-.8421	-.1811
Gαs of Striatum	LSD	WKY	SHR	.63536*	.04700	.000	.5330	.7378
			MPH	.25672*	.04700	.000	.1543	.3591
			LMQXM-LD	.34770*	.04700	.000	.2453	.4501
			LMQXM-MD	.14025*	.04700	.011	.0378	.2427
			LMQXM-HD	.45742*	.04700	.000	.3550	.5598
		SHR	WKY	-.63536*	.04700	.000	-.7378	-.5330
			MPH	-.37864*	.04700	.000	-.4810	-.2762
			LMQXM-LD	-.28766*	.04700	.000	-.3901	-.1852
			LMQXM-MD	-.49510*	.04700	.000	-.5975	-.3927
			LMQXM-HD	-.17793*	.04700	.003	-.2803	-.0755
		MPH	WKY	-.25672*	.04700	.000	-.3591	-.1543
			SHR	.37864*	.04700	.000	.2762	.4810
			LMQXM-LD	.09098	.04700	.077	-.0114	.1934
			LMQXM-MD	-.11647*	.04700	.029	-.2189	-.0141
			LMQXM-HD	.20070*	.04700	.001	.0983	.3031
		LMQXM-LD	WKY	-.34770*	.04700	.000	-.4501	-.2453
			SHR	.28766*	.04700	.000	.1852	.3901
			MPH	-.09098	.04700	.077	-.1934	.0114
			LMQXM-MD	-.20745*	.04700	.001	-.3099	-.1050
			LMQXM-HD	.10972*	.04700	.038	.0073	.2121
		LMQXM-MD	WKY	-.14025*	.04700	.011	-.2427	-.0378
			SHR	.49510*	.04700	.000	.3927	.5975
			MPH	.11647*	.04700	.029	.0141	.2189
			LMQXM-LD	.20745*	.04700	.001	.1050	.3099
			LMQXM-HD	.31717*	.04700	.000	.2148	.4196
		LMQXM-HD	WKY	-.45742*	.04700	.000	-.5598	-.3550
			SHR	.17793*	.04700	.003	.0755	.2803
			MPH	-.20070*	.04700	.001	-.3031	-.0983

			LMQXM-LD	-.10972*	.04700	.038	-.2121	-.0073
			LMQXM-MD	-.31717*	.04700	.000	-.4196	-.2148
Gαolf of Striatum	LSD	WKY	SHR	.34412*	.11146	.009	.1013	.5870
			MPH	-.23252	.11146	.059	-.4754	.0103
			LMQXM-LD	.09589	.11146	.406	-.1470	.3387
			LMQXM-MD	-.01283	.11146	.910	-.2557	.2300
			LMQXM-HD	.10681	.11146	.357	-.1360	.3497
		SHR	WKY	-.34412*	.11146	.009	-.5870	-.1013
			MPH	-.57664*	.11146	.000	-.8195	-.3338
			LMQXM-LD	-.24824*	.11146	.046	-.4911	-.0054
			LMQXM-MD	-.35696*	.11146	.008	-.5998	-.1141
			LMQXM-HD	-.23732	.11146	.055	-.4802	.0055
		MPH	WKY	.23252	.11146	.059	-.0103	.4754
			SHR	.57664*	.11146	.000	.3338	.8195
			LMQXM-LD	.32840*	.11146	.012	.0856	.5713
			LMQXM-MD	.21968	.11146	.072	-.0232	.4625
			LMQXM-HD	.33932*	.11146	.010	.0965	.5822
		LMQXM-LD	WKY	-.09589	.11146	.406	-.3387	.1470
			SHR	.24824*	.11146	.046	.0054	.4911
			MPH	-.32840*	.11146	.012	-.5713	-.0856
			LMQXM-MD	-.10872	.11146	.349	-.3516	.1341
			LMQXM-HD	.01092	.11146	.924	-.2319	.2538
		LMQXM-MD	WKY	.01283	.11146	.910	-.2300	.2557
			SHR	.35696*	.11146	.008	.1141	.5998
			MPH	-.21968	.11146	.072	-.4625	.0232
			LMQXM-LD	.10872	.11146	.349	-.1341	.3516
			LMQXM-HD	.11964	.11146	.304	-.1232	.3625
		LMQXM-HD	WKY	-.10681	.11146	.357	-.3497	.1360
			SHR	.23732	.11146	.055	-.0055	.4802
			MPH	-.33932*	.11146	.010	-.5822	-.0965
			LMQXM-LD	-.01092	.11146	.924	-.2538	.2319
			LMQXM-MD	-.11964	.11146	.304	-.3625	.1232
PKA of Striatum	LSD	WKY	SHR	.36914*	.13072	.015	.0843	.6540
			MPH	-.26730	.13072	.063	-.5521	.0175
			LMQXM-LD	.06471	.13072	.629	-.2201	.3495
			LMQXM-MD	-.03508	.13072	.793	-.3199	.2497
			LMQXM-HD	.16039	.13072	.243	-.1244	.4452
		SHR	WKY	-.36914*	.13072	.015	-.6540	-.0843
			MPH	-.63645*	.13072	.000	-.9213	-.3516
			LMQXM-LD	-.30443*	.13072	.038	-.5892	-.0196

			LMQXM-MD	-.40422*	.13072	.009	-.6890	-.1194
			LMQXM-HD	-.20875	.13072	.136	-.4936	.0761
		MPH	WKY	.26730	.13072	.063	-.0175	.5521
			SHR	.63645*	.13072	.000	.3516	.9213
			LMQXM-LD	.33202*	.13072	.026	.0472	.6168
			LMQXM-MD	.23223	.13072	.101	-.0526	.5170
			LMQXM-HD	.42770*	.13072	.007	.1429	.7125
		LMQXM-LD	WKY	-.06471	.13072	.629	-.3495	.2201
			SHR	.30443*	.13072	.038	.0196	.5892
			MPH	-.33202*	.13072	.026	-.6168	-.0472
			LMQXM-MD	-.09979	.13072	.460	-.3846	.1850
			LMQXM-HD	.09568	.13072	.478	-.1891	.3805
		LMQXM-MD	WKY	.03508	.13072	.793	-.2497	.3199
			SHR	.40422*	.13072	.009	.1194	.6890
			MPH	-.23223	.13072	.101	-.5170	.0526
			LMQXM-LD	.09979	.13072	.460	-.1850	.3846
			LMQXM-HD	.19547	.13072	.161	-.0893	.4803
		LMQXM-HD	WKY	-.16039	.13072	.243	-.4452	.1244
			SHR	.20875	.13072	.136	-.0761	.4936
			MPH	-.42770*	.13072	.007	-.7125	-.1429
			LMQXM-LD	-.09568	.13072	.478	-.3805	.1891
			LMQXM-MD	-.19547	.13072	.161	-.4803	.0893
p-CREB of Striatum	LSD	WKY	SHR	.42026*	.08990	.001	.2244	.6161
			MPH	.19905*	.08990	.047	.0032	.3949
			LMQXM-LD	.31237*	.08990	.005	.1165	.5082
			LMQXM-MD	.09877	.08990	.293	-.0971	.2947
			LMQXM-HD	.28136*	.08990	.009	.0855	.4772
		SHR	WKY	-.42026*	.08990	.001	-.6161	-.2244
			MPH	-.22121*	.08990	.030	-.4171	-.0253
			LMQXM-LD	-.10789	.08990	.253	-.3038	.0880
			LMQXM-MD	-.32148*	.08990	.004	-.5174	-.1256
			LMQXM-HD	-.13889	.08990	.148	-.3348	.0570
		MPH	WKY	-.19905*	.08990	.047	-.3949	-.0032
			SHR	.22121*	.08990	.030	.0253	.4171
			LMQXM-LD	.11332	.08990	.231	-.0826	.3092
			LMQXM-MD	-.10027	.08990	.287	-.2961	.0956
			LMQXM-HD	.08231	.08990	.378	-.1136	.2782
		LMQXM-LD	WKY	-.31237*	.08990	.005	-.5082	-.1165
			SHR	.10789	.08990	.253	-.0880	.3038

			MPH	-.11332	.08990	.231	-.3092	.0826
			LMQXM-MD	-.21359*	.08990	.035	-.4095	-.0177
			LMQXM-HD	-.03101	.08990	.736	-.2269	.1649
		LMQXM-MD	WKY	-.09877	.08990	.293	-.2947	.0971
			SHR	.32148*	.08990	.004	.1256	.5174
			MPH	.10027	.08990	.287	-.0956	.2961
			LMQXM-LD	.21359*	.08990	.035	.0177	.4095
			LMQXM-HD	.18259	.08990	.065	-.0133	.3785
		LMQXM-HD	WKY	-.28136*	.08990	.009	-.4772	-.0855
			SHR	.13889	.08990	.148	-.0570	.3348
			MPH	-.08231	.08990	.378	-.2782	.1136
			LMQXM-LD	.03101	.08990	.736	-.1649	.2269
			LMQXM-MD	-.18259	.08990	.065	-.3785	.0133
BDNF of Striatum	LSD	WKY	SHR	.25650*	.07900	.007	.0844	.4286
			MPH	-.03523	.07900	.664	-.2074	.1369
			LMQXM-LD	.15759	.07900	.069	-.0145	.3297
			LMQXM-MD	-.11594	.07900	.168	-.2881	.0562
			LMQXM-HD	.06566	.07900	.422	-.1065	.2378
		SHR	WKY	-.25650*	.07900	.007	-.4286	-.0844
			MPH	-.29173*	.07900	.003	-.4639	-.1196
			LMQXM-LD	-.09891	.07900	.234	-.2710	.0732
			LMQXM-MD	-.37244*	.07900	.001	-.5446	-.2003
			LMQXM-HD	-.19084*	.07900	.033	-.3630	-.0187
		MPH	WKY	.03523	.07900	.664	-.1369	.2074
			SHR	.29173*	.07900	.003	.1196	.4639
			LMQXM-LD	.19281*	.07900	.031	.0207	.3650
			LMQXM-MD	-.08072	.07900	.327	-.2529	.0914
			LMQXM-HD	.10088	.07900	.226	-.0713	.2730
		LMQXM-LD	WKY	-.15759	.07900	.069	-.3297	.0145
			SHR	.09891	.07900	.234	-.0732	.2710
			MPH	-.19281*	.07900	.031	-.3650	-.0207
			LMQXM-MD	-.27353*	.07900	.005	-.4457	-.1014
			LMQXM-HD	-.09193	.07900	.267	-.2641	.0802
		LMQXM-MD	WKY	.11594	.07900	.168	-.0562	.2881
			SHR	.37244*	.07900	.001	.2003	.5446
			MPH	.08072	.07900	.327	-.0914	.2529
			LMQXM-LD	.27353*	.07900	.005	.1014	.4457
			LMQXM-HD	.18160*	.07900	.040	.0095	.3537
		LMQXM-HD	WKY	-.06566	.07900	.422	-.2378	.1065
			SHR	.19084*	.07900	.033	.0187	.3630

			MPH	-.10088	.07900	.226	-.2730	.0713
			LMQXM-LD	.09193	.07900	.267	-.0802	.2641
			LMQXM-MD	-.18160*	.07900	.040	-.3537	-.0095

upplementary Table 25 Effect of LMQXM on the relative expression of DRD1 and PKA mRNA in rat PFC and Striatum（$\bar{x} \pm s$，n=5）

Group	DRD1 mRNA		PKA mRNA	
	PFC	Striatum	PFC	Striatum
WKY	1.18±0.62	1.10±0.49	1.06±0.34	1.12±0.53
SHR	0.34±0.38 $^{\#\#}$	0.25±0.24 $^{\#\#}$	0.44±0.03 $^{\#\#}$	0.26±0.10 $^{\#\#}$
MPH	1.10±0.37 *	0.82±0.22 *	1.29±0.38 ***	0.90±0.38 *
LMQXM-LD	1.01±0.68 *	0.78±0.12 *	0.95±0.23 *	0.79±0.28 *
LMQXM-MD	1.33±0.31 **	0.91±0.38 **	1.31±0.35 ***	1.06±0.39 **
LMQXM-HD	1.08±0.44 *	0.77±0.44 *	0.74±0.43 $^{\wedge\wedge}$	0.75±0.37 *

Note: Compared with the WKY group, $^{\#}P < 0.05$, $^{\#\#}P < 0.01$; compared with the SHR group, $^{*}P < 0.05$, $^{**}P < 0.01$, $^{***}P < 0.001$; compared with the LMQXM-MD group, $^{\wedge\wedge}P < 0.01$.

Supplementary Table 26 Effect of LMQXM on the relative expression of Gαs and Gαolf mRNA in rat PFC and Striatum（$\bar{x} \pm s$，n=5）

Group	Gαs		Gαolf	
	PFC	Striatum	PFC	Striatum
WKY	1.25±0.70	1.10±0.43	1.17±0.54	1.05±0.36
SHR	0.38±0.36 $^{\#\#}$	0.12±0.09 $^{\#}$	0.29±0.19 $^{\#\#}$	0.18±0.15 $^{\#\#}$
MPH	1.03±0.23 *	0.93±1.05 *	1.10±0.60 **	0.74±0.44 *
LMQXM-LD	0.69±0.13	0.37±0.19	0.93±0.48 *	0.70±0.53 *
LMQXM-MD	1.12±0.37 **	0.99±0.55 *	1.11±0.11 **	0.81±0.27 *
LMQXM-HD	0.91±0.39 *	0.39±0.47	0.89±0.45 *	0.47±0.49

Note: Compared with the WKY group, $^{\#}P < 0.05$, $^{\#\#}P < 0.01$; compared with the SHR group, $^{*}P < 0.05$, $^{**}P < 0.01$.

Supplementary Table 27 Effect of LMQXM on the relative expression of CREB and BDNF mRNA in rat PFC and Striatum（$\bar{x} \pm s$，n=5）

Group	CREB		BDNF	
	PFC	Striatum	PFC	Striatum
WKY	1.12±0.44	1.06±0.45	1.14±0.60	1.16±0.55
SHR	0.40±0.09 $^{\#\#}$	0.32±0.16 $^{\#\#}$	0.35±0.17 $^{\#}$	0.25±0.33 $^{\#\#}$
MPH	0.95±0.45 *	0.94±0.67 *	1.14±0.25 *	1.15±0.46 **

LMQXM-LD	0.91±0.19 *	0.55±0.12 ^	0.96±0.49 *	0.99±0.35 *
LMQXM-MD	1.05±0.47 *	1.25±0.46 **	1.21±0.69 **	1.27±0.73 **
LMQXM-HD	0.59±0.44	0.85±0.30 *	0.54±0.21 ^	0.91±0.48 *

Note: Compared with the WKY group, $^{\#\#}P < 0.01$, $^{\#\#\#}P < 0.001$; compared with the SHR group, $^{*}P < 0.05$, $^{**}P < 0.01$; compared with the LMQXM-MD group, $^{\wedge}P < 0.05$.

Supplementary Table 28 One-ANOVA of the relative expression of DRD1, Gαs, Gαolf, PKA, p-CREB, and BDNF mRNA in rat PFC and Striatum.

Homogeneity of variance test					
		Levin statistics	Degree of freedom 1	Degree of freedom 2	significance
BDNF of PFC	Based on the mean	2.244	5	24	.083
	Based on the median	1.375	5	24	.269
	Based on the median and with the adjusted degrees of freedom	1.375	5	12.099	.300
	Based on the post-cut average value	2.150	5	24	.094
DRD1 of PFC	Based on the mean	2.468	5	24	.061
	Based on the median	.977	5	24	.452
	Based on the median and with the adjusted degrees of freedom	.977	5	13.964	.465
	Based on the post-cut average value	2.368	5	24	.070
Gαs of PFC	Based on the mean	1.890	5	24	.134
	Based on the median	.951	5	24	.467
	Based on the median and with the adjusted degrees of freedom	.951	5	13.326	.481
	Based on the post-cut average value	1.821	5	24	.147
Gαolf OF PFC	Based on the mean	1.861	5	24	.139
	Based on the median	.791	5	24	.567
	Based on the median and with the adjusted degrees of freedom	.791	5	16.242	.571
	Based on the post-cut average value	1.736	5	24	.165
PKA of PFC	Based on the mean	1.879	5	24	.136
	Based on the median	.686	5	24	.638
	Based on the median and with the adjusted degrees of freedom	.686	5	16.103	.641
	Based on the post-cut average value	1.745	5	24	.163
CREB of PFC	Based on the mean	2.238	5	24	.083
	Based on the median	.758	5	24	.589
	Based on the median and with the adjusted degrees of freedom	.758	5	15.427	.594

	Based on the post-cut average value	1.997	5	24	.115
BDNF of STRIATUM	Based on the mean	1.121	5	24	.376
	Based on the median	.543	5	24	.742
	Based on the median and with the adjusted degrees of freedom	.543	5	19.793	.742
	Based on the post-cut average value	1.065	5	24	.404
DRD1 of STRIATUM	Based on the mean	1.919	5	24	.128
	Based on the median	1.082	5	24	.395
	Based on the median and with the adjusted degrees of freedom	1.082	5	17.921	.403
	Based on the post-cut average value	1.848	5	24	.141
Gαs of STRIATUM	Based on the mean	2.416	5	24	.066
	Based on the median	.673	5	24	.647
	Based on the median and with the adjusted degrees of freedom	.673	5	9.351	.654
	Based on the post-cut average value	1.879	5	24	.136
Gαolf of striatum	Based on the mean	1.312	5	24	.292
	Based on the median	.823	5	24	.545
	Based on the median and with the adjusted degrees of freedom	.823	5	19.254	.548
	Based on the post-cut average value	1.351	5	24	.277
PKA of striatum	Based on the mean	2.656	5	24	.048
	Based on the median	.867	5	24	.518
	Based on the median and with the adjusted degrees of freedom	.867	5	16.307	.524
	Based on the post-cut average value	2.559	5	24	.054
CREB of striatum	Based on the mean	1.947	5	24	.124
	Based on the median	.590	5	24	.708
	Based on the median and with the adjusted degrees of freedom	.590	5	9.465	.709
	Based on the post-cut average value	1.588	5	24	.201

ANOVA

				quadratic sum	free degree	mean square	F	significance
BDNF OF PFC	interblock	(assemble)		3.228	5	.646	3.212	.023
		linear term	contrast	.024	1	.024	.122	.730
			bias in statistics	3.204	4	.801	3.984	.013
	Within the group			4.824	24	.201		
	total			8.052	29			

DRD1 OF PFC	interblock	(assemble)		2.953	5	.591	2.700	.045
		linear term	contrast	.405	1	.405	1.850	.186
			bias in statistics	2.548	4	.637	2.913	.043
	Within the group			5.249	24	.219		
	total			8.201	29			
Gαs OF PFC	interblock	(assemble)		2.524	5	.505	3.089	.027
		linear term	contrast	.003	1	.003	.016	.899
			bias in statistics	2.522	4	.630	3.857	.015
	Within the group			3.923	24	.163		
	total			6.447	29			
Gαolf OF PFC	interblock	(assemble)		2.643	5	.529	2.803	.039
		linear term	contrast	.056	1	.056	.295	.592
			bias in statistics	2.587	4	.647	3.430	.024
	Within the group			4.526	24	.189		
	total			7.169	29			
PKA OF PFC	interblock	(assemble)		2.782	5	.556	5.332	.002
		linear term	contrast	.033	1	.033	.318	.578
			bias in statistics	2.749	4	.687	6.585	.001
	Within the group			2.504	24	.104		
	total			5.286	29			
CREB OF PFC	interblock	(assemble)		1.963	5	.393	2.755	.042
		linear term	contrast	.038	1	.038	.269	.608
			bias in statistics	1.924	4	.481	3.376	.025
	Within the group			3.419	24	.142		
	total			5.382	29			
BDNF of striatum	interblock	(assemble)		3.390	5	.678	2.686	.046
		linear term	contrast	.195	1	.195	.773	.388
			bias in statistics	3.195	4	.799	3.164	.032
	Within the group			6.059	24	.252		
	total			9.449	29			
DRD1 of striatum	interblock	(assemble)		2.019	5	.404	3.440	.017
		linear term	contrast	.008	1	.008	.065	.801
			bias in statistics	2.011	4	.503	4.284	.009

	Within the group			2.817	24	.117		
	total			4.836	29			
Gαs of striatum	interblock	(assemble)		4.126	5	.825	2.677	.046
		linear term	contrast	.163	1	.163	.528	.474
			bias in statistics	3.964	4	.991	3.215	.030
	Within the group			7.398	24	.308		
	total			11.525	29			
Gαolf of striatum	interblock	(assemble)		2.258	5	.452	2.896	.035
		linear term	contrast	.078	1	.078	.499	.487
			bias in statistics	2.180	4	.545	3.495	.022
	Within the group			3.743	24	.156		
	total			6.001	29			
PKA of striatum	interblock	(assemble)		2.393	5	.479	3.559	.015
		linear term	contrast	.014	1	.014	.107	.746
			bias in statistics	2.379	4	.595	4.422	.008
	Within the group			3.228	24	.135		
	total			5.621	29			
CREB of striatum	interblock	(assemble)		2.928	5	.586	3.526	.016
		linear term	contrast	.131	1	.131	.792	.382
			bias in statistics	2.796	4	.699	4.209	.010
	Within the group			3.986	24	.166		
	total			6.913	29			

Multiple comparisons

LSD

dependent variable	(I) Group	(J) Group	Mean Difference Value (I-J)	Standard error	significance	And the 95% confidence interval	
						lower limit	superior limit
BDNF OF PFC	WKY	SHR	.79201*	.28355	.010	.2068	1.3772
		MPH	.00339	.28355	.991	-.5818	.5886
		LMQXM-LD	.17633	.28355	.540	-.4089	.7615
		LMQXM-MD	-.06850	.28355	.811	-.6537	.5167
		LMQXM-HD	.59876*	.28355	.045	.0135	1.1840
	SHR	WKY	-.79201*	.28355	.010	-1.3772	-.2068
		MPH	-.78862*	.28355	.010	-1.3738	-.2034
		LMQXM-LD	-.61568*	.28355	.040	-1.2009	-.0305

		LMQXM-MD	-.86051*	.28355	.006	-1.4457	-.2753
		LMQXM-HD	-.19325	.28355	.502	-.7785	.3920
	MPH	WKY	-.00339	.28355	.991	-.5886	.5818
		SHR	.78862*	.28355	.010	.2034	1.3738
		LMQXM-LD	.17294	.28355	.548	-.4123	.7582
		LMQXM-MD	-.07188	.28355	.802	-.6571	.5133
		LMQXM-HD	.59537*	.28355	.046	.0101	1.1806
	LMQXM-LD	WKY	-.17633	.28355	.540	-.7615	.4089
		SHR	.61568*	.28355	.040	.0305	1.2009
		MPH	-.17294	.28355	.548	-.7582	.4123
		LMQXM-MD	-.24482	.28355	.396	-.8300	.3404
		LMQXM-HD	.42243	.28355	.149	-.1628	1.0077
	LMQXM-MD	WKY	.06850	.28355	.811	-.5167	.6537
		SHR	.86051*	.28355	.006	.2753	1.4457
		MPH	.07188	.28355	.802	-.5133	.6571
		LMQXM-LD	.24482	.28355	.396	-.3404	.8300
		LMQXM-HD	.66726*	.28355	.027	.0820	1.2525
	LMQXM-HD	WKY	-.59876*	.28355	.045	-1.1840	-.0135
		SHR	.19325	.28355	.502	-.3920	.7785
		MPH	-.59537*	.28355	.046	-1.1806	-.0101
		LMQXM-LD	-.42243	.28355	.149	-1.0077	.1628
		LMQXM-MD	-.66726*	.28355	.027	-1.2525	-.0820
DRD1 OF PFC	WKY	SHR	.83697*	.29577	.009	.2265	1.4474
		MPH	.08111	.29577	.786	-.5293	.6915
		LMQXM-LD	.16898	.29577	.573	-.4415	.7794
		LMQXM-MD	-.15258	.29577	.611	-.7630	.4579
		LMQXM-HD	.10010	.29577	.738	-.5103	.7105
	SHR	WKY	-.83697*	.29577	.009	-1.4474	-.2265
		MPH	-.75586*	.29577	.017	-1.3663	-.1454
		LMQXM-LD	-.66799*	.29577	.033	-1.2784	-.0576
		LMQXM-MD	-.98955*	.29577	.003	-1.6000	-.3791
		LMQXM-HD	-.73687*	.29577	.020	-1.3473	-.1264
	MPH	WKY	-.08111	.29577	.786	-.6915	.5293
		SHR	.75586*	.29577	.017	.1454	1.3663
		LMQXM-LD	.08787	.29577	.769	-.5226	.6983
		LMQXM-MD	-.23369	.29577	.437	-.8441	.3767
		LMQXM-HD	.01899	.29577	.949	-.5914	.6294
	LMQXM-LD	WKY	-.16898	.29577	.573	-.7794	.4415
		SHR	.66799*	.29577	.033	.0576	1.2784
		MPH	-.08787	.29577	.769	-.6983	.5226

		LMQXM-MD	-.32156	.29577	.288	-.9320	.2889
		LMQXM-HD	-.06888	.29577	.818	-.6793	.5416
	LMQXM-MD	WKY	.15258	.29577	.611	-.4579	.7630
		SHR	.98955*	.29577	.003	.3791	1.6000
		MPH	.23369	.29577	.437	-.3767	.8441
		LMQXM-LD	.32156	.29577	.288	-.2889	.9320
		LMQXM-HD	.25268	.29577	.401	-.3578	.8631
	LMQXM-HD	WKY	-.10010	.29577	.738	-.7105	.5103
		SHR	.73687*	.29577	.020	.1264	1.3473
		MPH	-.01899	.29577	.949	-.6294	.5914
		LMQXM-LD	.06888	.29577	.818	-.5416	.6793
		LMQXM-MD	-.25268	.29577	.401	-.8631	.3578
Gαs OF PFC	WKY	SHR	.87205*	.25569	.002	.3443	1.3998
		MPH	.21854	.25569	.401	-.3092	.7463
		LMQXM-LD	.55792*	.25569	.039	.0302	1.0856
		LMQXM-MD	.12810	.25569	.621	-.3996	.6558
		LMQXM-HD	.33985	.25569	.196	-.1879	.8676
	SHR	WKY	-.87205*	.25569	.002	-1.3998	-.3443
		MPH	-.65352*	.25569	.017	-1.1812	-.1258
		LMQXM-LD	-.31413	.25569	.231	-.8419	.2136
		LMQXM-MD	-.74396*	.25569	.008	-1.2717	-.2162
		LMQXM-HD	-.53221*	.25569	.048	-1.0599	-.0045
	MPH	WKY	-.21854	.25569	.401	-.7463	.3092
		SHR	.65352*	.25569	.017	.1258	1.1812
		LMQXM-LD	.33938	.25569	.197	-.1883	.8671
		LMQXM-MD	-.09044	.25569	.727	-.6182	.4373
		LMQXM-HD	.12131	.25569	.639	-.4064	.6490
	LMQXM-LD	WKY	-.55792*	.25569	.039	-1.0856	-.0302
		SHR	.31413	.25569	.231	-.2136	.8419
		MPH	-.33938	.25569	.197	-.8671	.1883
		LMQXM-MD	-.42983	.25569	.106	-.9575	.0979
		LMQXM-HD	-.21808	.25569	.402	-.7458	.3096
	LMQXM-MD	WKY	-.12810	.25569	.621	-.6558	.3996
		SHR	.74396*	.25569	.008	.2162	1.2717
		MPH	.09044	.25569	.727	-.4373	.6182
		LMQXM-LD	.42983	.25569	.106	-.0979	.9575
		LMQXM-HD	.21175	.25569	.416	-.3160	.7395
	LMQXM-HD	WKY	-.33985	.25569	.196	-.8676	.1879
		SHR	.53221*	.25569	.048	.0045	1.0599

		MPH	-.12131	.25569	.639	-.6490	.4064
		LMQXM-LD	.21808	.25569	.402	-.3096	.7458
		LMQXM-MD	-.21175	.25569	.416	-.7395	.3160
Gαolf of PFC	WKY	SHR	.87926*	.27466	.004	.3124	1.4461
		MPH	.07168	.27466	.796	-.4952	.6386
		LMQXM-LD	.23507	.27466	.401	-.3318	.8019
		LMQXM-MD	.05738	.27466	.836	-.5095	.6243
		LMQXM-HD	.28387	.27466	.312	-.2830	.8507
	SHR	WKY	-.87926*	.27466	.004	-1.4461	-.3124
		MPH	-.80758*	.27466	.007	-1.3745	-.2407
		LMQXM-LD	-.64419*	.27466	.028	-1.2111	-.0773
		LMQXM-MD	-.82188*	.27466	.006	-1.3888	-.2550
		LMQXM-HD	-.59539*	.27466	.040	-1.1623	-.0285
	MPH	WKY	-.07168	.27466	.796	-.6386	.4952
		SHR	.80758*	.27466	.007	.2407	1.3745
		LMQXM-LD	.16339	.27466	.557	-.4035	.7303
		LMQXM-MD	-.01430	.27466	.959	-.5812	.5526
		LMQXM-HD	.21219	.27466	.447	-.3547	.7791
	LMQXM-LD	WKY	-.23507	.27466	.401	-.8019	.3318
		SHR	.64419*	.27466	.028	.0773	1.2111
		MPH	-.16339	.27466	.557	-.7303	.4035
		LMQXM-MD	-.17769	.27466	.524	-.7446	.3892
		LMQXM-HD	.04880	.27466	.860	-.5181	.6157
	LMQXM-MD	WKY	-.05738	.27466	.836	-.6243	.5095
		SHR	.82188*	.27466	.006	.2550	1.3888
		MPH	.01430	.27466	.959	-.5526	.5812
		LMQXM-LD	.17769	.27466	.524	-.3892	.7446
		LMQXM-HD	.22649	.27466	.418	-.3404	.7934
	LMQXM-HD	WKY	-.28387	.27466	.312	-.8507	.2830
		SHR	.59539*	.27466	.040	.0285	1.1623
		MPH	-.21219	.27466	.447	-.7791	.3547
		LMQXM-LD	-.04880	.27466	.860	-.6157	.5181
		LMQXM-MD	-.22649	.27466	.418	-.7934	.3404
PKA OF PFC	WKY	SHR	.61078*	.20431	.006	.1891	1.0324
		MPH	-.23129	.20431	.269	-.6530	.1904
		LMQXM-LD	.10326	.20431	.618	-.3184	.5249
		LMQXM-MD	-.25853	.20431	.218	-.6802	.1631
		LMQXM-HD	.31845	.20431	.132	-.1032	.7401
	SHR	WKY	-.61078*	.20431	.006	-1.0324	-.1891
		MPH	-.84207*	.20431	.000	-1.2637	-.4204

		LMQXM-LD	-.50752*	.20431	.020	-.9292	-.0859
		LMQXM-MD	-.86931*	.20431	.000	-1.2910	-.4476
		LMQXM-HD	-.29233	.20431	.165	-.7140	.1293
	MPH	WKY	.23129	.20431	.269	-.1904	.6530
		SHR	.84207*	.20431	.000	.4204	1.2637
		LMQXM-LD	.33455	.20431	.115	-.0871	.7562
		LMQXM-MD	-.02725	.20431	.895	-.4489	.3944
		LMQXM-HD	.54974*	.20431	.013	.1281	.9714
	LMQXM-LD	WKY	-.10326	.20431	.618	-.5249	.3184
		SHR	.50752*	.20431	.020	.0859	.9292
		MPH	-.33455	.20431	.115	-.7562	.0871
		LMQXM-MD	-.36179	.20431	.089	-.7835	.0599
		LMQXM-HD	.21519	.20431	.303	-.2065	.6369
	LMQXM-MD	WKY	.25853	.20431	.218	-.1631	.6802
		SHR	.86931*	.20431	.000	.4476	1.2910
		MPH	.02725	.20431	.895	-.3944	.4489
		LMQXM-LD	.36179	.20431	.089	-.0599	.7835
		LMQXM-HD	.57699*	.20431	.009	.1553	.9987
	LMQXM-HD	WKY	-.31845	.20431	.132	-.7401	.1032
		SHR	.29233	.20431	.165	-.1293	.7140
		MPH	-.54974*	.20431	.013	-.9714	-.1281
		LMQXM-LD	-.21519	.20431	.303	-.6369	.2065
		LMQXM-MD	-.57699*	.20431	.009	-.9987	-.1553
CREB OF PFC	WKY	SHR	.71975*	.23872	.006	.2270	1.2124
		MPH	.17026	.23872	.483	-.3224	.6630
		LMQXM-LD	.21250	.23872	.382	-.2802	.7052
		LMQXM-MD	.07196	.23872	.766	-.4207	.5647
		LMQXM-HD	.52683*	.23872	.037	.0341	1.0195
	SHR	WKY	-.71975*	.23872	.006	-1.2124	-.2270
		MPH	-.54949*	.23872	.030	-1.0422	-.0568
		LMQXM-LD	-.50725*	.23872	.044	-.9999	-.0145
		LMQXM-MD	-.64778*	.23872	.012	-1.1405	-.1551
		LMQXM-HD	-.19291	.23872	.427	-.6856	.2998
	MPH	WKY	-.17026	.23872	.483	-.6630	.3224
		SHR	.54949*	.23872	.030	.0568	1.0422
		LMQXM-LD	.04224	.23872	.861	-.4505	.5349
		LMQXM-MD	-.09830	.23872	.684	-.5910	.3944
		LMQXM-HD	.35657	.23872	.148	-.1361	.8493
	LMQXM-LD	WKY	-.21250	.23872	.382	-.7052	.2802

		SHR	.50725*	.23872	.044	.0145	.9999
		MPH	-.04224	.23872	.861	-.5349	.4505
		LMQXM-MD	-.14054	.23872	.562	-.6332	.3522
		LMQXM-HD	.31433	.23872	.200	-.1784	.8070
	LMQXM-MD	WKY	-.07196	.23872	.766	-.5647	.4207
		SHR	.64778*	.23872	.012	.1551	1.1405
		MPH	.09830	.23872	.684	-.3944	.5910
		LMQXM-LD	.14054	.23872	.562	-.3522	.6332
		LMQXM-HD	.45487	.23872	.069	-.0378	.9476
	LMQXM-HD	WKY	-.52683*	.23872	.037	-1.0195	-.0341
		SHR	.19291	.23872	.427	-.2998	.6856
		MPH	-.35657	.23872	.148	-.8493	.1361
		LMQXM-LD	-.31433	.23872	.200	-.8070	.1784
		LMQXM-MD	-.45487	.23872	.069	-.9476	.0378
BDNF of striatum	WKY	SHR	.91060*	.31778	.009	.2547	1.5665
		MPH	.01428	.31778	.965	-.6416	.6701
		LMQXM-LD	.17192	.31778	.593	-.4839	.8278
		LMQXM-MD	-.10907	.31778	.734	-.7649	.5468
		LMQXM-HD	.24972	.31778	.440	-.4061	.9056
	SHR	WKY	-.91060*	.31778	.009	-1.5665	-.2547
		MPH	-.89632*	.31778	.009	-1.5522	-.2405
		LMQXM-LD	-.73867*	.31778	.029	-1.3945	-.0828
		LMQXM-MD	-1.01967*	.31778	.004	-1.6755	-.3638
		LMQXM-HD	-.66088*	.31778	.048	-1.3167	-.0050
	MPH	WKY	-.01428	.31778	.965	-.6701	.6416
		SHR	.89632*	.31778	.009	.2405	1.5522
		LMQXM-LD	.15764	.31778	.624	-.4982	.8135
		LMQXM-MD	-.12335	.31778	.701	-.7792	.5325
		LMQXM-HD	.23544	.31778	.466	-.4204	.8913
	LMQXM-LD	WKY	-.17192	.31778	.593	-.8278	.4839
		SHR	.73867*	.31778	.029	.0828	1.3945
		MPH	-.15764	.31778	.624	-.8135	.4982
		LMQXM-MD	-.28099	.31778	.385	-.9369	.3749
		LMQXM-HD	.07779	.31778	.809	-.5781	.7337
	LMQXM-MD	WKY	.10907	.31778	.734	-.5468	.7649
		SHR	1.01967*	.31778	.004	.3638	1.6755
		MPH	.12335	.31778	.701	-.5325	.7792
		LMQXM-LD	.28099	.31778	.385	-.3749	.9369
		LMQXM-HD	.35879	.31778	.270	-.2971	1.0146
	LMQXM-HD	WKY	-.24972	.31778	.440	-.9056	.4061

		SHR	.66088*	.31778	.048	.0050	1.3167
		MPH	-.23544	.31778	.466	-.8913	.4204
		LMQXM-LD	-.07779	.31778	.809	-.7337	.5781
		LMQXM-MD	-.35879	.31778	.270	-1.0146	.2971
DRD1 of striatum	WKY	SHR	.85000*	.21668	.001	.4028	1.2972
		MPH	.27346	.21668	.219	-.1737	.7207
		LMQXM-LD	.31272	.21668	.162	-.1345	.7599
		LMQXM-MD	.18459	.21668	.403	-.2626	.6318
		LMQXM-HD	.32610	.21668	.145	-.1211	.7733
	SHR	WKY	-.85000*	.21668	.001	-1.2972	-.4028
		MPH	-.57654*	.21668	.014	-1.0237	-.1293
		LMQXM-LD	-.53728*	.21668	.021	-.9845	-.0901
		LMQXM-MD	-.66541*	.21668	.005	-1.1126	-.2182
		LMQXM-HD	-.52391*	.21668	.024	-.9711	-.0767
	MPH	WKY	-.27346	.21668	.219	-.7207	.1737
		SHR	.57654*	.21668	.014	.1293	1.0237
		LMQXM-LD	.03926	.21668	.858	-.4079	.4865
		LMQXM-MD	-.08887	.21668	.685	-.5361	.3583
		LMQXM-HD	.05263	.21668	.810	-.3946	.4998
	LMQXM-LD	WKY	-.31272	.21668	.162	-.7599	.1345
		SHR	.53728*	.21668	.021	.0901	.9845
		MPH	-.03926	.21668	.858	-.4865	.4079
		LMQXM-MD	-.12813	.21668	.560	-.5753	.3191
		LMQXM-HD	.01337	.21668	.951	-.4338	.4606
	LMQXM-MD	WKY	-.18459	.21668	.403	-.6318	.2626
		SHR	.66541*	.21668	.005	.2182	1.1126
		MPH	.08887	.21668	.685	-.3583	.5361
		LMQXM-LD	.12813	.21668	.560	-.3191	.5753
		LMQXM-HD	.14150	.21668	.520	-.3057	.5887
	LMQXM-HD	WKY	-.32610	.21668	.145	-.7733	.1211
		SHR	.52391*	.21668	.024	.0767	.9711
		MPH	-.05263	.21668	.810	-.4998	.3946
		LMQXM-LD	-.01337	.21668	.951	-.4606	.4338
		LMQXM-MD	-.14150	.21668	.520	-.5887	.3057
GAS of striatum	WKY	SHR	.98085*	.35115	.010	.2561	1.7056
		MPH	.17476	.35115	.623	-.5500	.8995
		LMQXM-LD	.73129*	.35115	.048	.0066	1.4560
		LMQXM-MD	.10871	.35115	.760	-.6160	.8334
		LMQXM-HD	.71393	.35115	.053	-.0108	1.4387

	SHR	WKY	-.98085*	.35115	.010	-1.7056	-.2561
		MPH	-.80608*	.35115	.031	-1.5308	-.0813
		LMQXM-LD	-.24956	.35115	.484	-.9743	.4752
		LMQXM-MD	-.87214*	.35115	.020	-1.5969	-.1474
		LMQXM-HD	-.26692	.35115	.455	-.9917	.4578
	MPH	WKY	-.17476	.35115	.623	-.8995	.5500
		SHR	.80608*	.35115	.031	.0813	1.5308
		LMQXM-LD	.55652	.35115	.126	-.1682	1.2813
		LMQXM-MD	-.06606	.35115	.852	-.7908	.6587
		LMQXM-HD	.53916	.35115	.138	-.1856	1.2639
	LMQXM-LD	WKY	-.73129*	.35115	.048	-1.4560	-.0066
		SHR	.24956	.35115	.484	-.4752	.9743
		MPH	-.55652	.35115	.126	-1.2813	.1682
		LMQXM-MD	-.62258	.35115	.089	-1.3473	.1022
		LMQXM-HD	-.01736	.35115	.961	-.7421	.7074
	LMQXM-MD	WKY	-.10871	.35115	.760	-.8334	.6160
		SHR	.87214*	.35115	.020	.1474	1.5969
		MPH	.06606	.35115	.852	-.6587	.7908
		LMQXM-LD	.62258	.35115	.089	-.1022	1.3473
		LMQXM-HD	.60522	.35115	.098	-.1195	1.3300
	LMQXM-HD	WKY	-.71393	.35115	.053	-1.4387	.0108
		SHR	.26692	.35115	.455	-.4578	.9917
		MPH	-.53916	.35115	.138	-1.2639	.1856
		LMQXM-LD	.01736	.35115	.961	-.7074	.7421
		LMQXM-MD	-.60522	.35115	.098	-1.3300	.1195
GAOLF of striatum	WKY	SHR	.87187*	.24977	.002	.3564	1.3874
		MPH	.30757	.24977	.230	-.2079	.8231
		LMQXM-LD	.35294	.24977	.170	-.1625	.8684
		LMQXM-MD	.23802	.24977	.350	-.2775	.7535
		LMQXM-HD	.57997*	.24977	.029	.0645	1.0955
	SHR	WKY	-.87187*	.24977	.002	-1.3874	-.3564
		MPH	-.56430*	.24977	.033	-1.0798	-.0488
		LMQXM-LD	-.51893*	.24977	.049	-1.0344	-.0034
		LMQXM-MD	-.63386*	.24977	.018	-1.1493	-.1184
		LMQXM-HD	-.29190	.24977	.254	-.8074	.2236
	MPH	WKY	-.30757	.24977	.230	-.8231	.2079
		SHR	.56430*	.24977	.033	.0488	1.0798
		LMQXM-LD	.04537	.24977	.857	-.4701	.5609
		LMQXM-MD	-.06956	.24977	.783	-.5850	.4459
		LMQXM-HD	.27240	.24977	.286	-.2431	.7879

	LMQXM-LD	WKY	-.35294	.24977	.170	-.8684	.1625
		SHR	.51893*	.24977	.049	.0034	1.0344
		MPH	-.04537	.24977	.857	-.5609	.4701
		LMQXM-MD	-.11493	.24977	.650	-.6304	.4006
		LMQXM-HD	.22703	.24977	.372	-.2885	.7425
	LMQXM-MD	WKY	-.23802	.24977	.350	-.7535	.2775
		SHR	.63386*	.24977	.018	.1184	1.1493
		MPH	.06956	.24977	.783	-.4459	.5850
		LMQXM-LD	.11493	.24977	.650	-.4006	.6304
		LMQXM-HD	.34196	.24977	.184	-.1735	.8574
	LMQXM-HD	WKY	-.57997*	.24977	.029	-1.0955	-.0645
		SHR	.29190	.24977	.254	-.2236	.8074
		MPH	-.27240	.24977	.286	-.7879	.2431
		LMQXM-LD	-.22703	.24977	.372	-.7425	.2885
		LMQXM-MD	-.34196	.24977	.184	-.8574	.1735
PKA of striatum	WKY	SHR	.86278*	.23195	.001	.3841	1.3415
		MPH	.21891	.23195	.355	-.2598	.6976
		LMQXM-LD	.32630	.23195	.172	-.1524	.8050
		LMQXM-MD	.05587	.23195	.812	-.4229	.5346
		LMQXM-HD	.37290	.23195	.121	-.1058	.8516
	SHR	WKY	-.86278*	.23195	.001	-1.3415	-.3841
		MPH	-.64388*	.23195	.010	-1.1226	-.1652
		LMQXM-LD	-.53648*	.23195	.030	-1.0152	-.0578
		LMQXM-MD	-.80692*	.23195	.002	-1.2856	-.3282
		LMQXM-HD	-.48988*	.23195	.045	-.9686	-.0112
	MPH	WKY	-.21891	.23195	.355	-.6976	.2598
		SHR	.64388*	.23195	.010	.1652	1.1226
		LMQXM-LD	.10740	.23195	.648	-.3713	.5861
		LMQXM-MD	-.16304	.23195	.489	-.6418	.3157
		LMQXM-HD	.15400	.23195	.513	-.3247	.6327
	LMQXM-LD	WKY	-.32630	.23195	.172	-.8050	.1524
		SHR	.53648*	.23195	.030	.0578	1.0152
		MPH	-.10740	.23195	.648	-.5861	.3713
		LMQXM-MD	-.27044	.23195	.255	-.7492	.2083
		LMQXM-HD	.04660	.23195	.842	-.4321	.5253
	LMQXM-MD	WKY	-.05587	.23195	.812	-.5346	.4229
		SHR	.80692*	.23195	.002	.3282	1.2856
		MPH	.16304	.23195	.489	-.3157	.6418
		LMQXM-LD	.27044	.23195	.255	-.2083	.7492

		LMQXM-HD	.31704	.23195	.184	-.1617	.7958
	LMQXM-HD	WKY	-.37290	.23195	.121	-.8516	.1058
		SHR	.48988*	.23195	.045	.0112	.9686
		MPH	-.15400	.23195	.513	-.6327	.3247
		LMQXM-LD	-.04660	.23195	.842	-.5253	.4321
		LMQXM-MD	-.31704	.23195	.184	-.7958	.1617
CREB of striatum	WKY	SHR	.74696*	.25774	.008	.2150	1.2789
		MPH	.12619	.25774	.629	-.4058	.6581
		LMQXM-LD	.51046	.25774	.059	-.0215	1.0424
		LMQXM-MD	-.18927	.25774	.470	-.7212	.3427
		LMQXM-HD	.21355	.25774	.416	-.3184	.7455
	SHR	WKY	-.74696*	.25774	.008	-1.2789	-.2150
		MPH	-.62077*	.25774	.024	-1.1527	-.0888
		LMQXM-LD	-.23650	.25774	.368	-.7684	.2954
		LMQXM-MD	-.93623*	.25774	.001	-1.4682	-.4043
		LMQXM-HD	-.53341*	.25774	.049	-1.0654	-.0015
	MPH	WKY	-.12619	.25774	.629	-.6581	.4058
		SHR	.62077*	.25774	.024	.0888	1.1527
		LMQXM-LD	.38427	.25774	.149	-.1477	.9162
		LMQXM-MD	-.31546	.25774	.233	-.8474	.2165
		LMQXM-HD	.08736	.25774	.738	-.4446	.6193
	LMQXM-LD	WKY	-.51046	.25774	.059	-1.0424	.0215
		SHR	.23650	.25774	.368	-.2954	.7684
		MPH	-.38427	.25774	.149	-.9162	.1477
		LMQXM-MD	-.69973*	.25774	.012	-1.2317	-.1678
		LMQXM-HD	-.29692	.25774	.261	-.8289	.2350
	LMQXM-MD	WKY	.18927	.25774	.470	-.3427	.7212
		SHR	.93623*	.25774	.001	.4043	1.4682
		MPH	.31546	.25774	.233	-.2165	.8474
		LMQXM-LD	.69973*	.25774	.012	.1678	1.2317
		LMQXM-HD	.40282	.25774	.131	-.1291	.9348
	LMQXM-HD	WKY	-.21355	.25774	.416	-.7455	.3184
		SHR	.53341*	.25774	.049	.0015	1.0654
		MPH	-.08736	.25774	.738	-.6193	.4446
		LMQXM-LD	.29692	.25774	.261	-.2350	.8289
		LMQXM-MD	-.40282	.25774	.131	-.9348	.1291
