# Supplementary figures and images for: Long Mu Qing Xin mixture improves behavioral performance in spontaneously hypertensive rats (SHR/NCrl) by upregulating catecholamine neurotransmitters in prefrontal cortex and striatum via DRD1/cAMP/PKA-CREB signaling pathway (part 3 of 4)
Source: Front Pharmacol. 2024 Jul 4;15:1387359. doi: 10.3389/fphar.2024.1387359 (PMC11254830; doi:10.3389/fphar.2024.1387359)

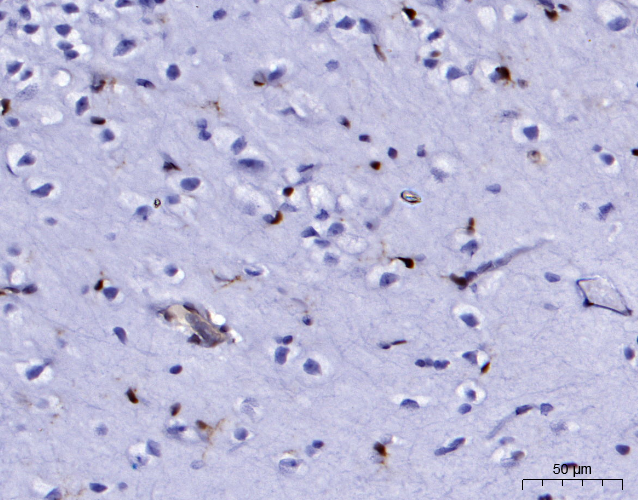

Supplement: Supplementary file 13 [file DataSheet10.ZIP › IHC Raw Image of p-CREB in striatum (3)/H48 1-200 PCREB_20.0x.tif-W5.tif]

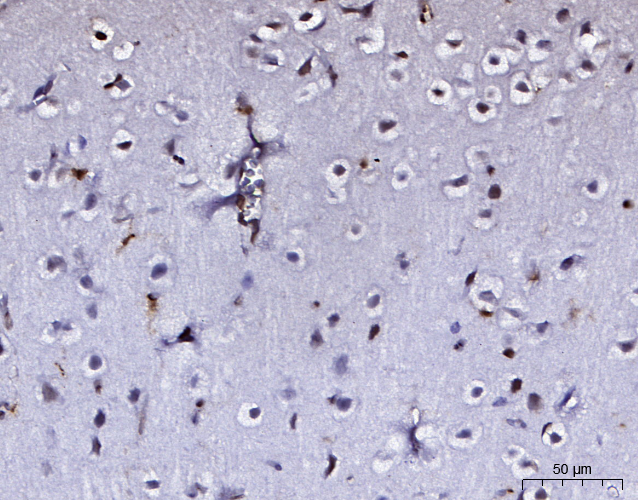

Supplement: Supplementary file 13 [file DataSheet10.ZIP › IHC Raw Image of p-CREB in striatum (3)/H53 1-200 PCREB_20.0x.tif-W1.tif]

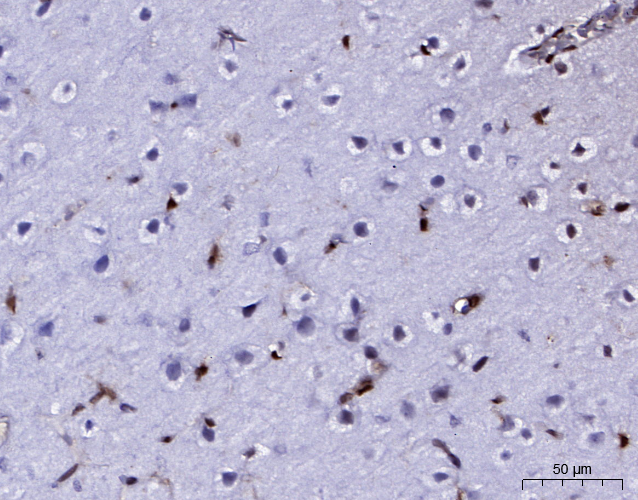

Supplement: Supplementary file 13 [file DataSheet10.ZIP › IHC Raw Image of p-CREB in striatum (3)/H53 1-200 PCREB_20.0x.tif-W2.tif]

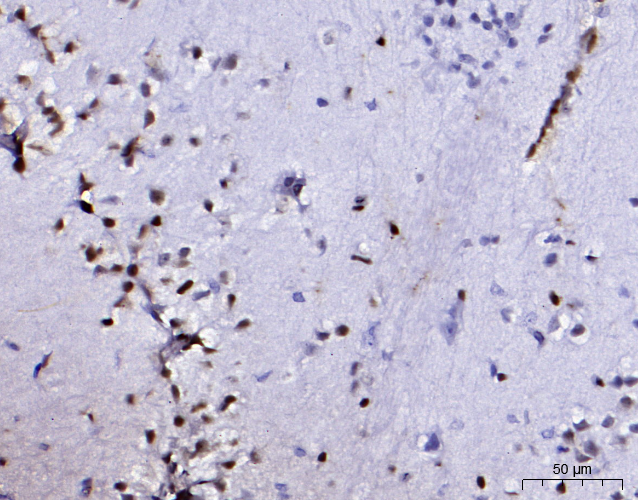

Supplement: Supplementary file 13 [file DataSheet10.ZIP › IHC Raw Image of p-CREB in striatum (3)/H53 1-200 PCREB_20.0x.tif-W3.tif]

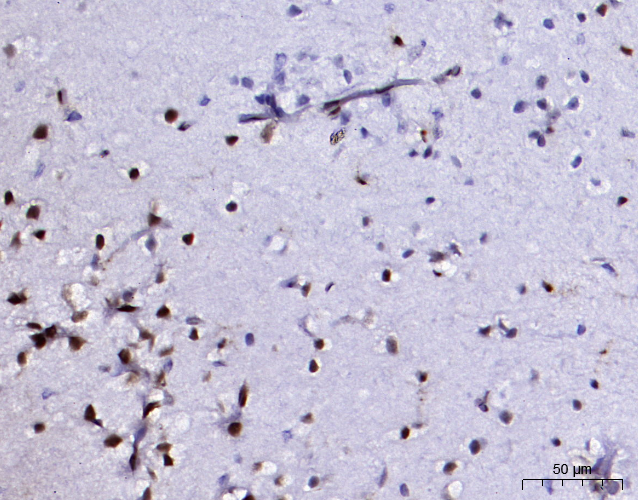

Supplement: Supplementary file 13 [file DataSheet10.ZIP › IHC Raw Image of p-CREB in striatum (3)/H53 1-200 PCREB_20.0x.tif-W4.tif]

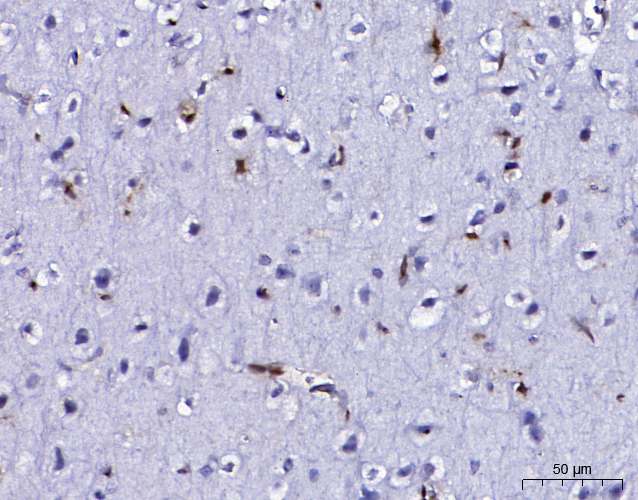

Supplement: Supplementary file 13 [file DataSheet10.ZIP › IHC Raw Image of p-CREB in striatum (3)/H53 1-200 PCREB_20.0x.tif-W5.tif]

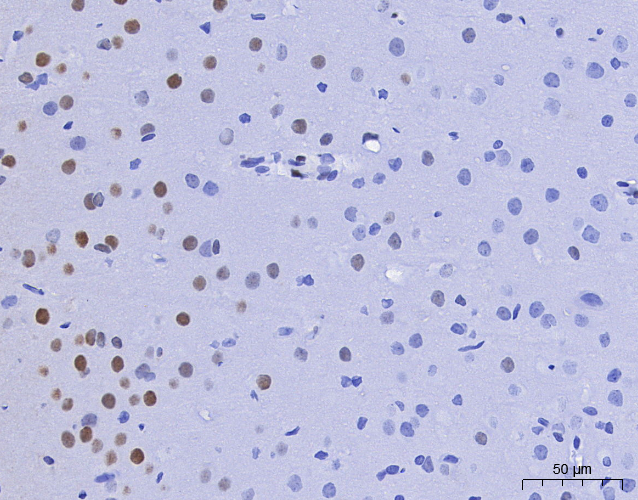

Supplement: Supplementary file 13 [file DataSheet10.ZIP › IHC Raw Image of p-CREB in striatum (3)/M11 CREB_20.0x.tif-W1.tif]

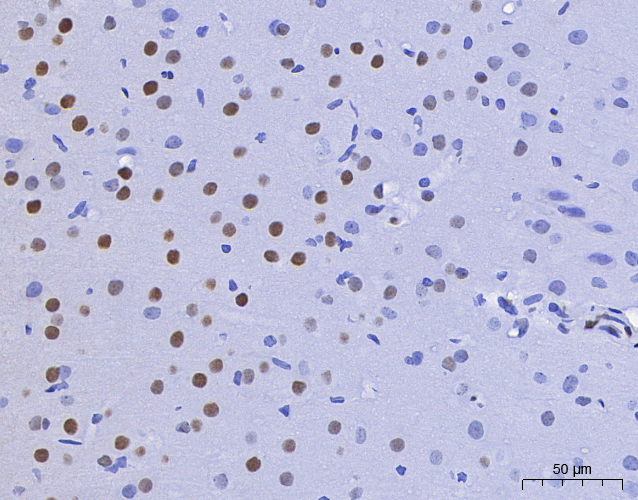

Supplement: Supplementary file 13 [file DataSheet10.ZIP › IHC Raw Image of p-CREB in striatum (3)/M11 CREB_20.0x.tif-W2.tif]

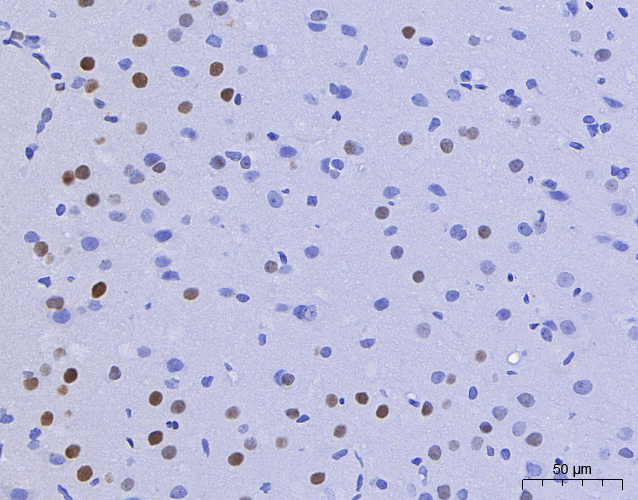

Supplement: Supplementary file 13 [file DataSheet10.ZIP › IHC Raw Image of p-CREB in striatum (3)/M11 CREB_20.0x.tif-W3.tif]

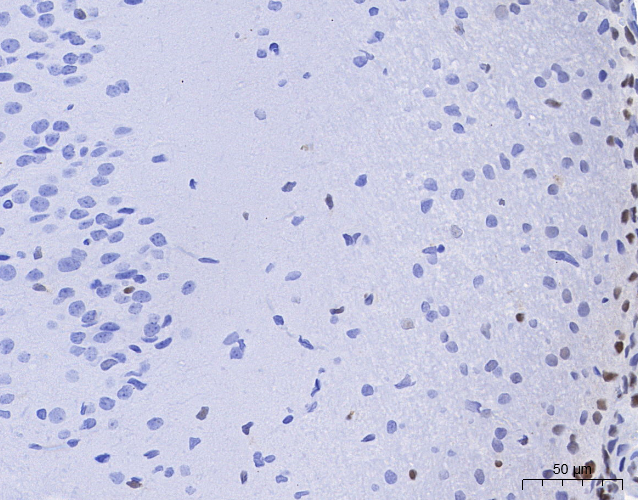

Supplement: Supplementary file 13 [file DataSheet10.ZIP › IHC Raw Image of p-CREB in striatum (3)/M11 CREB_20.0x.tif-W4.tif]

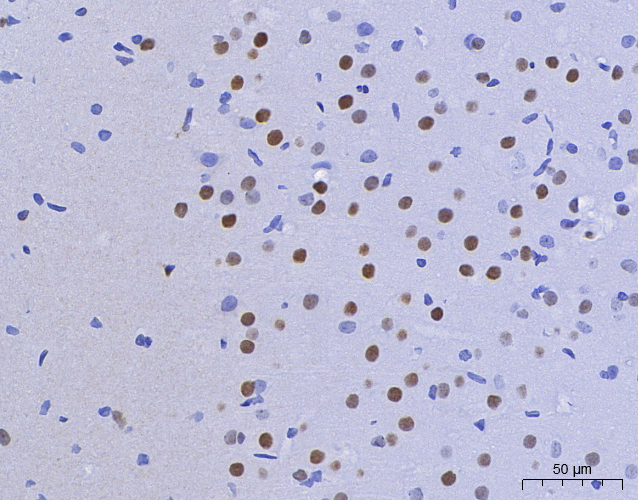

Supplement: Supplementary file 13 [file DataSheet10.ZIP › IHC Raw Image of p-CREB in striatum (3)/M11 CREB_20.0x.tif-W5.tif]

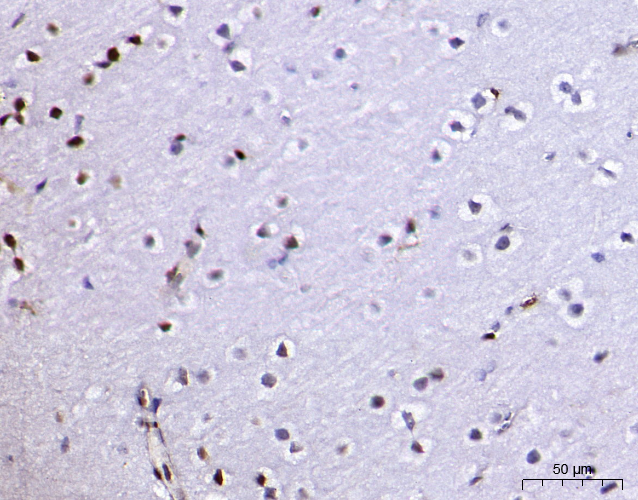

Supplement: Supplementary file 13 [file DataSheet10.ZIP › IHC Raw Image of p-CREB in striatum (3)/M27 1-200 PCREB_20.0x.tif-W1.tif]

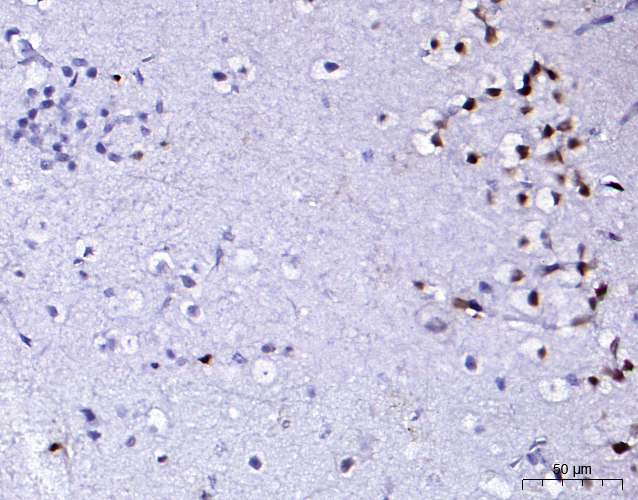

Supplement: Supplementary file 13 [file DataSheet10.ZIP › IHC Raw Image of p-CREB in striatum (3)/M27 1-200 PCREB_20.0x.tif-W2.tif]

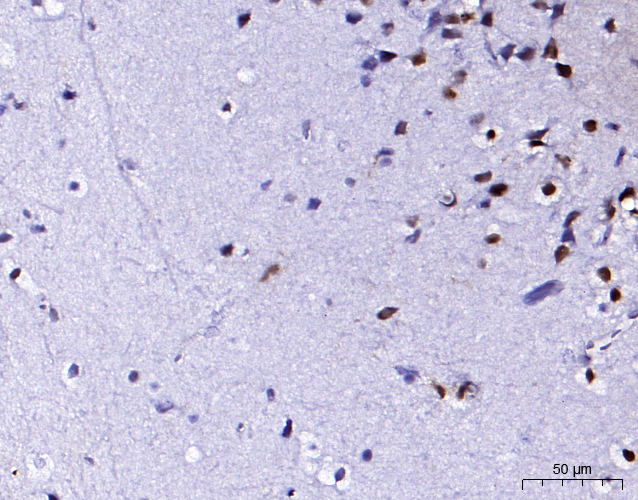

Supplement: Supplementary file 13 [file DataSheet10.ZIP › IHC Raw Image of p-CREB in striatum (3)/M27 1-200 PCREB_20.0x.tif-W3.tif]

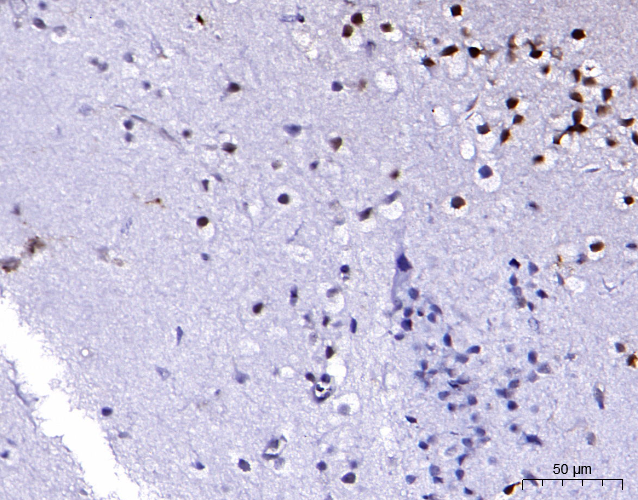

Supplement: Supplementary file 13 [file DataSheet10.ZIP › IHC Raw Image of p-CREB in striatum (3)/M27 1-200 PCREB_20.0x.tif-W4.tif]

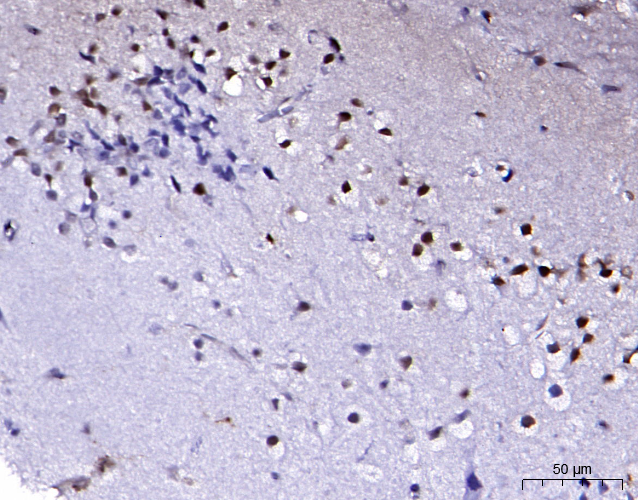

Supplement: Supplementary file 13 [file DataSheet10.ZIP › IHC Raw Image of p-CREB in striatum (3)/M27 1-200 PCREB_20.0x.tif-W5.tif]

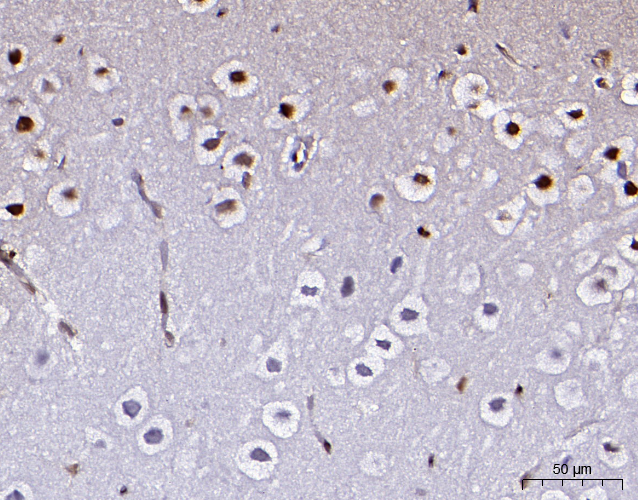

Supplement: Supplementary file 14 [file DataSheet6.ZIP › IHC Raw Image of P-CREB in PFC(1)/K61 1-200 PCREB_20.0x.tif-Q1.tif]

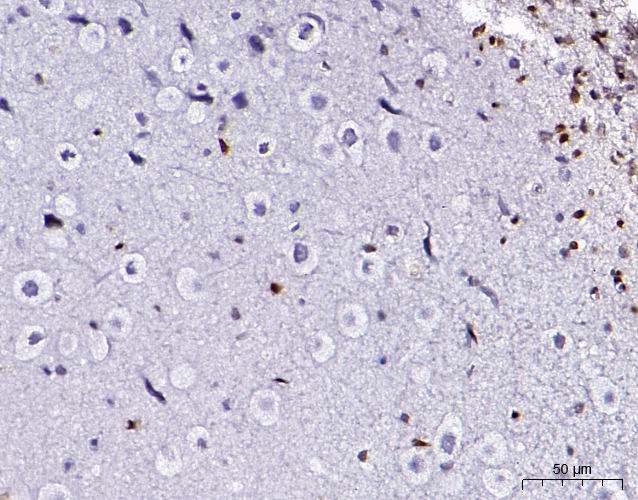

Supplement: Supplementary file 14 [file DataSheet6.ZIP › IHC Raw Image of P-CREB in PFC(1)/K61 1-200 PCREB_20.0x.tif-Q2.tif]

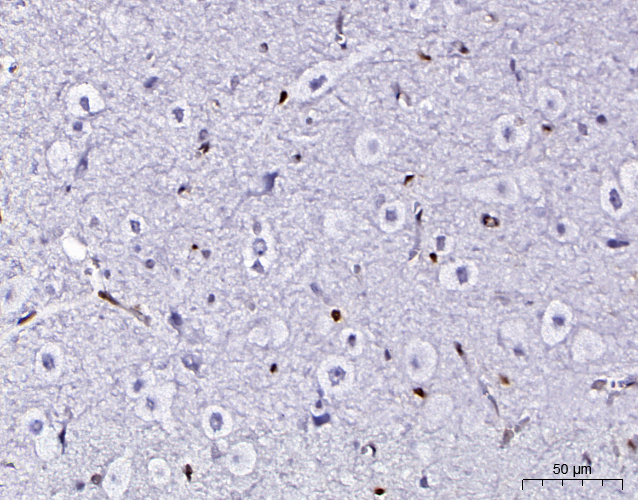

Supplement: Supplementary file 14 [file DataSheet6.ZIP › IHC Raw Image of P-CREB in PFC(1)/K61 1-200 PCREB_20.0x.tif-Q3.tif]

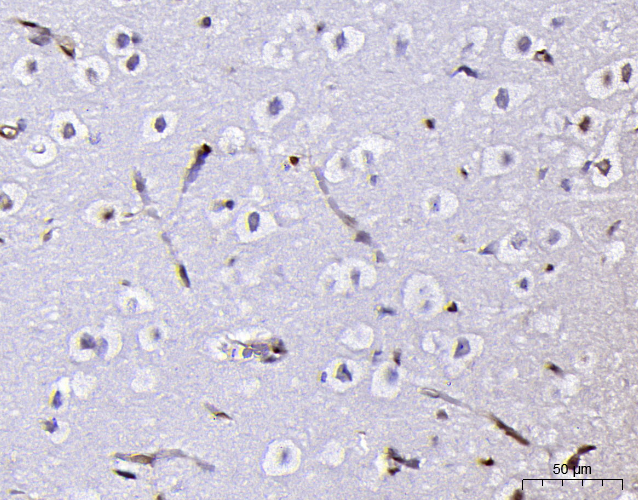

Supplement: Supplementary file 14 [file DataSheet6.ZIP › IHC Raw Image of P-CREB in PFC(1)/K61 1-200 PCREB_20.0x.tif-Q4.tif]

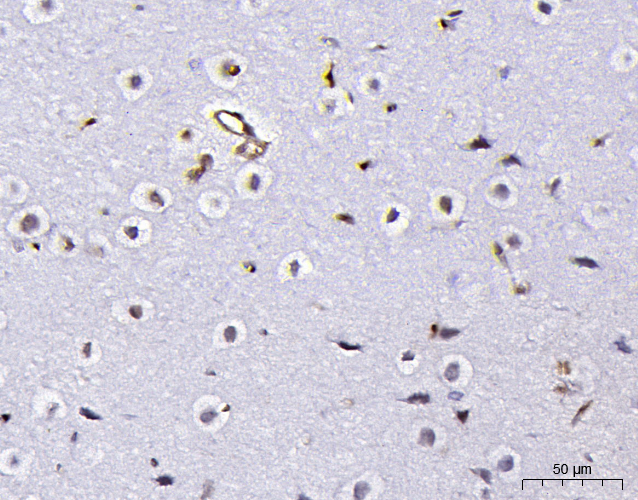

Supplement: Supplementary file 14 [file DataSheet6.ZIP › IHC Raw Image of P-CREB in PFC(1)/K61 1-200 PCREB_20.0x.tif-Q5.tif]

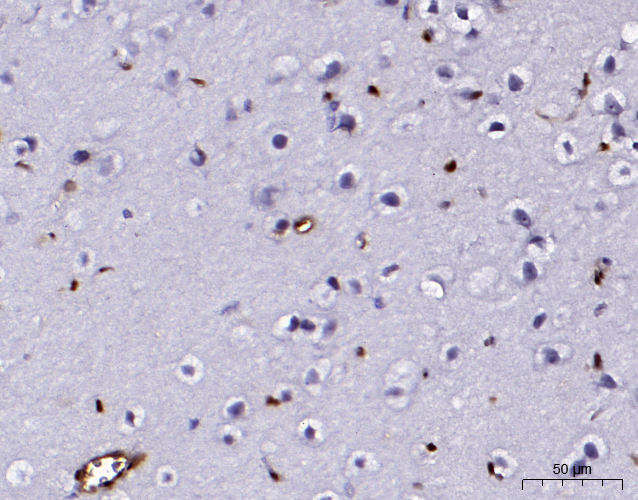

Supplement: Supplementary file 14 [file DataSheet6.ZIP › IHC Raw Image of P-CREB in PFC(1)/K65 1-200 PCREB_20.0x.tif-Q1.tif]

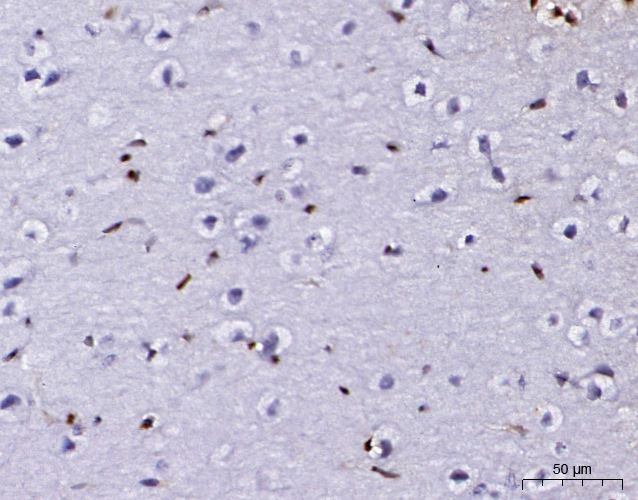

Supplement: Supplementary file 14 [file DataSheet6.ZIP › IHC Raw Image of P-CREB in PFC(1)/K65 1-200 PCREB_20.0x.tif-Q2.tif]

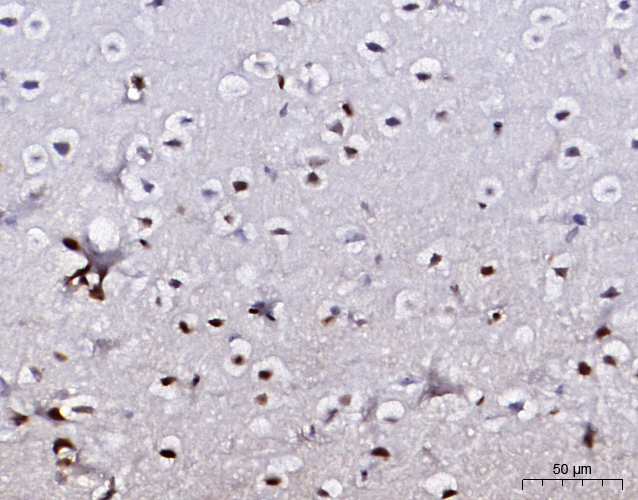

Supplement: Supplementary file 14 [file DataSheet6.ZIP › IHC Raw Image of P-CREB in PFC(1)/K65 1-200 PCREB_20.0x.tif-Q3.tif]

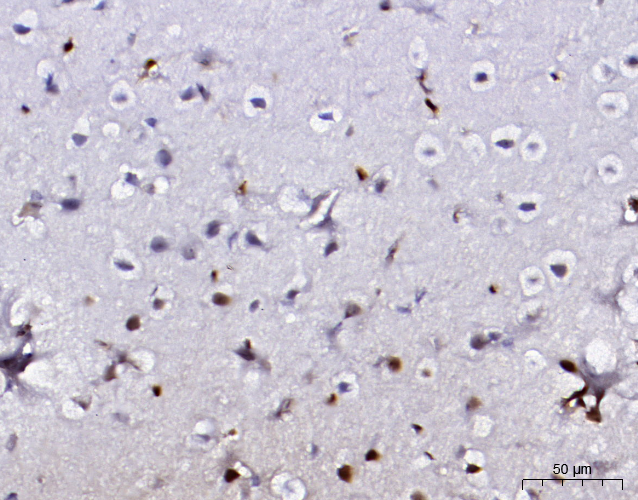

Supplement: Supplementary file 14 [file DataSheet6.ZIP › IHC Raw Image of P-CREB in PFC(1)/K65 1-200 PCREB_20.0x.tif-Q4.tif]

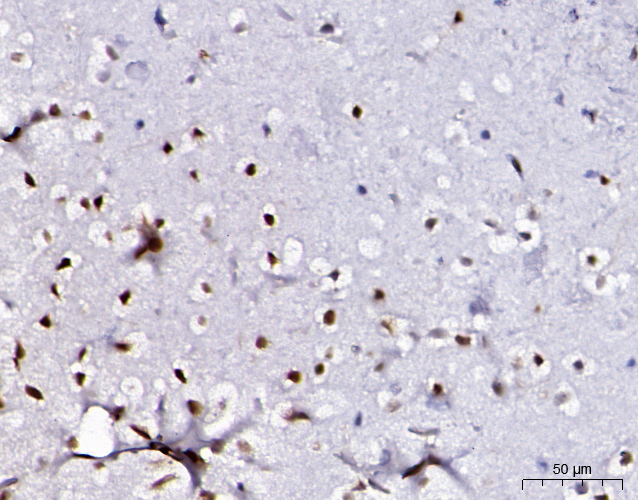

Supplement: Supplementary file 14 [file DataSheet6.ZIP › IHC Raw Image of P-CREB in PFC(1)/K65 1-200 PCREB_20.0x.tif-Q5.tif]

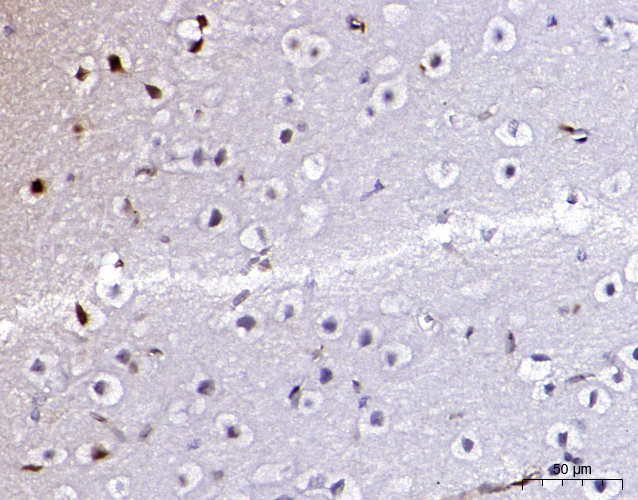

Supplement: Supplementary file 14 [file DataSheet6.ZIP › IHC Raw Image of P-CREB in PFC(1)/K67 1-200 PCREB_20.0x.tif-Q3.tif]

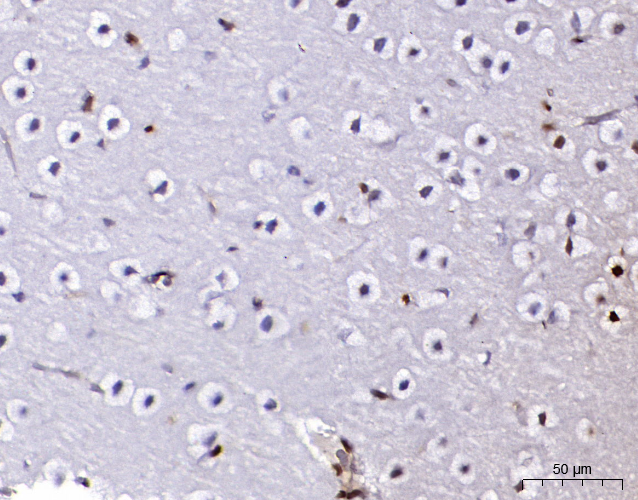

Supplement: Supplementary file 14 [file DataSheet6.ZIP › IHC Raw Image of P-CREB in PFC(1)/K67 1-200 PCREB_20.0x.tif-Q4.tif]

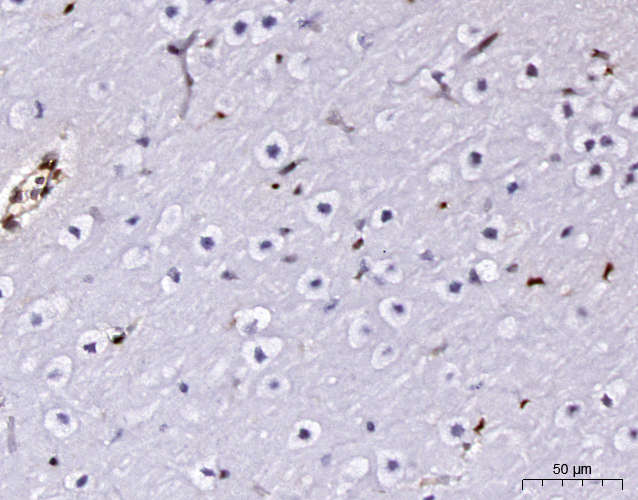

Supplement: Supplementary file 14 [file DataSheet6.ZIP › IHC Raw Image of P-CREB in PFC(1)/K67 1-200 PCREB_20.0x.tif-Q5.tif]

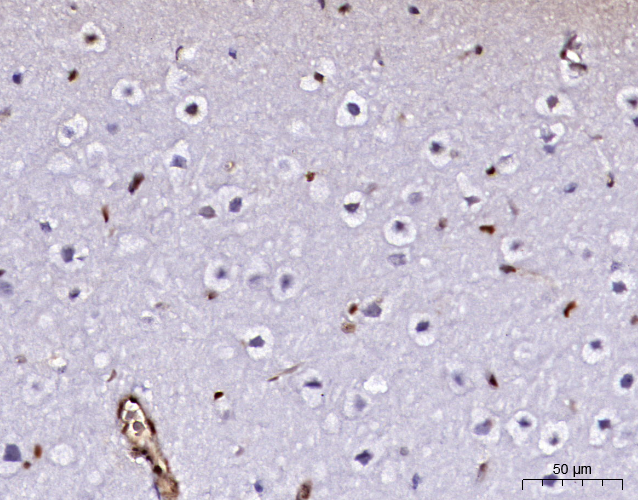

Supplement: Supplementary file 14 [file DataSheet6.ZIP › IHC Raw Image of P-CREB in PFC(1)/K67 1-200 PCREB_20.0x.tif-W1.tif]

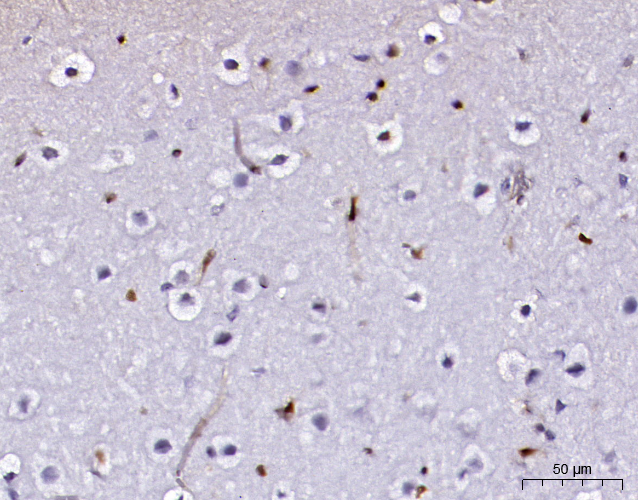

Supplement: Supplementary file 14 [file DataSheet6.ZIP › IHC Raw Image of P-CREB in PFC(1)/K67 1-200 PCREB_20.0x.tif-W2.tif]

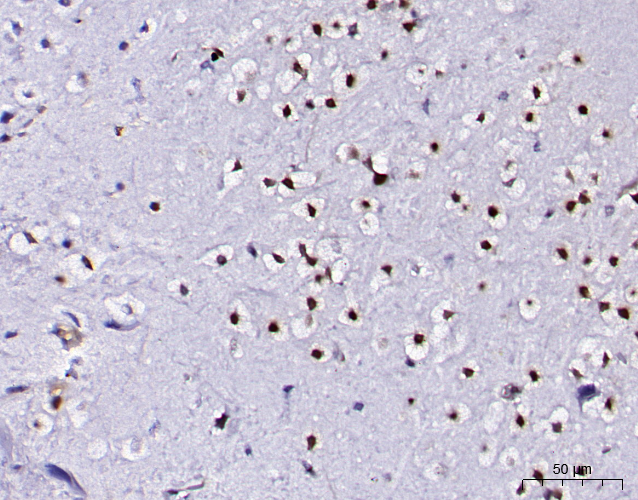

Supplement: Supplementary file 14 [file DataSheet6.ZIP › IHC Raw Image of P-CREB in PFC(1)/K69 1-200 PCREB_20.0x.tif-Q1.tif]

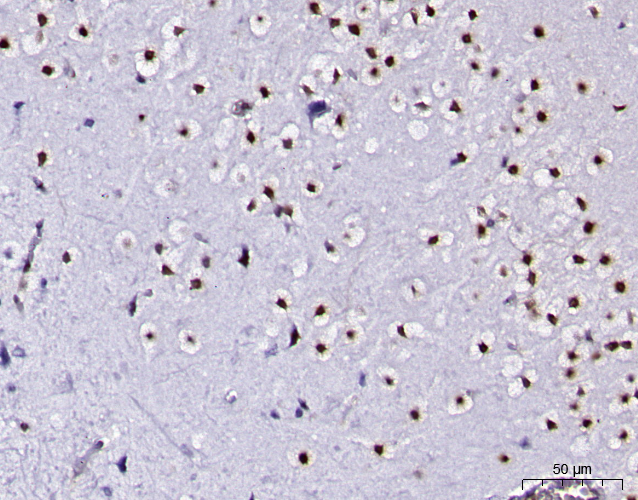

Supplement: Supplementary file 14 [file DataSheet6.ZIP › IHC Raw Image of P-CREB in PFC(1)/K69 1-200 PCREB_20.0x.tif-Q2.tif]

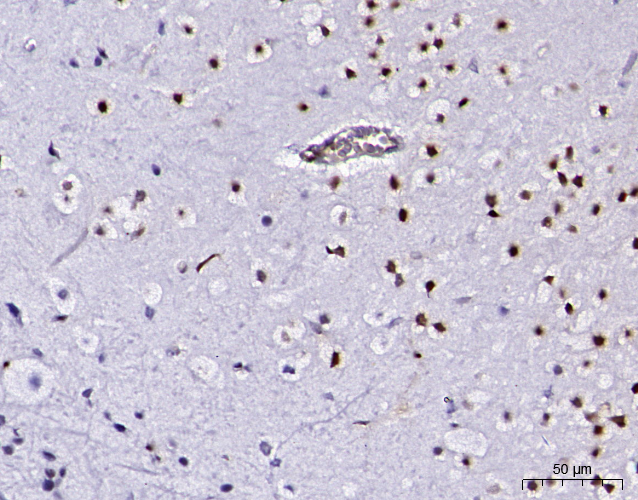

Supplement: Supplementary file 14 [file DataSheet6.ZIP › IHC Raw Image of P-CREB in PFC(1)/K69 1-200 PCREB_20.0x.tif-Q3.tif]

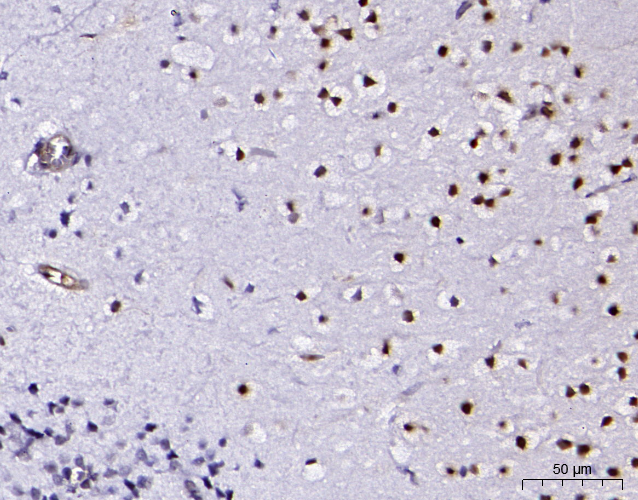

Supplement: Supplementary file 14 [file DataSheet6.ZIP › IHC Raw Image of P-CREB in PFC(1)/K69 1-200 PCREB_20.0x.tif-Q4.tif]

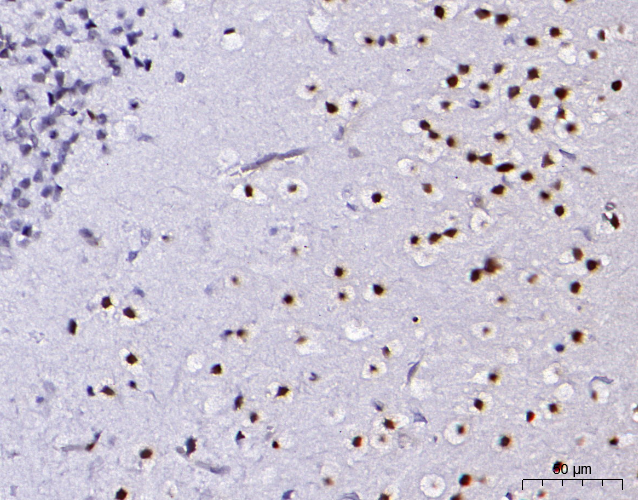

Supplement: Supplementary file 14 [file DataSheet6.ZIP › IHC Raw Image of P-CREB in PFC(1)/K69 1-200 PCREB_20.0x.tif-Q5.tif]

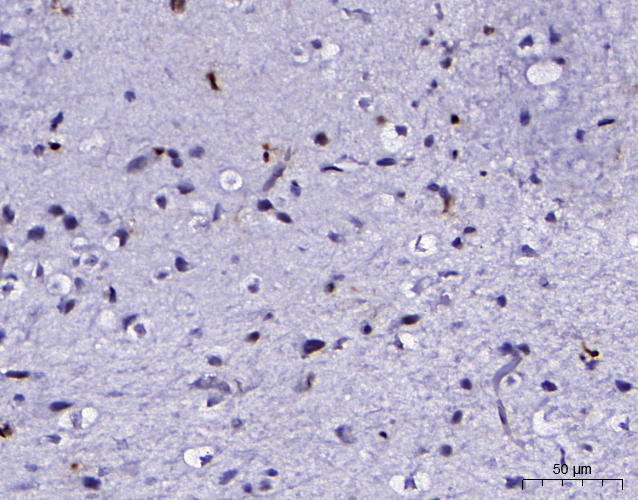

Supplement: Supplementary file 14 [file DataSheet6.ZIP › IHC Raw Image of P-CREB in PFC(1)/L7 1-200 PCREB_20.0x.tif-Q1.tif]

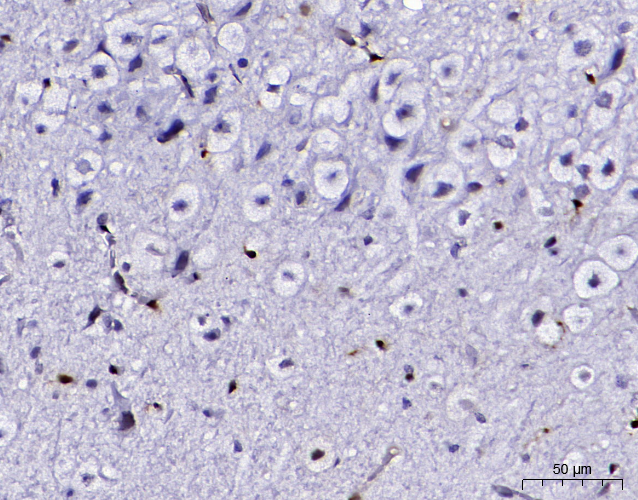

Supplement: Supplementary file 14 [file DataSheet6.ZIP › IHC Raw Image of P-CREB in PFC(1)/L7 1-200 PCREB_20.0x.tif-Q3.tif]

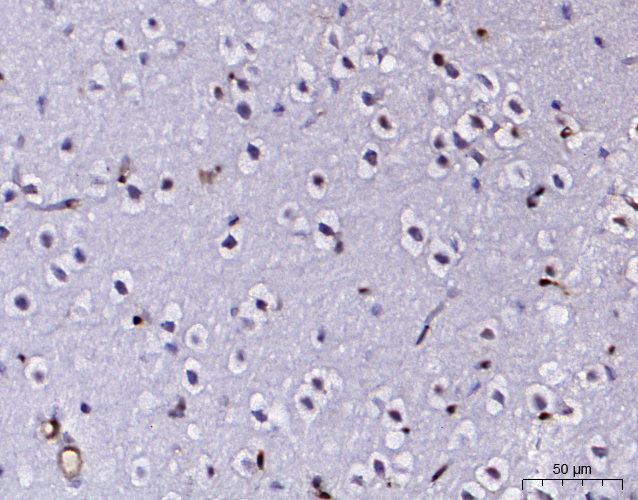

Supplement: Supplementary file 14 [file DataSheet6.ZIP › IHC Raw Image of P-CREB in PFC(1)/L7 1-200 PCREB_20.0x.tif-Q4.tif]

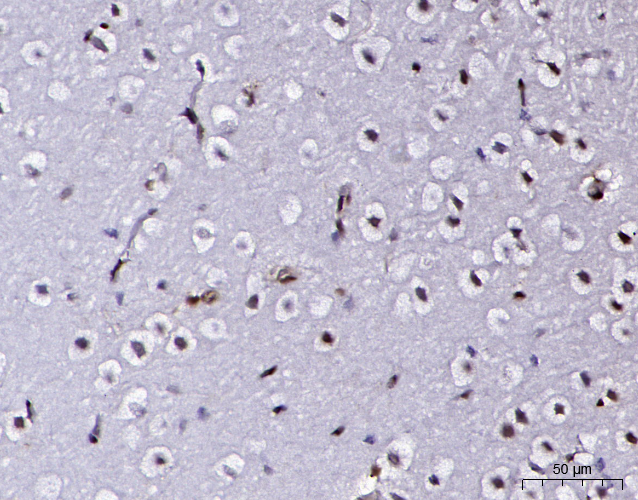

Supplement: Supplementary file 14 [file DataSheet6.ZIP › IHC Raw Image of P-CREB in PFC(1)/L7 1-200 PCREB_20.0x.tif-Q5.tif]

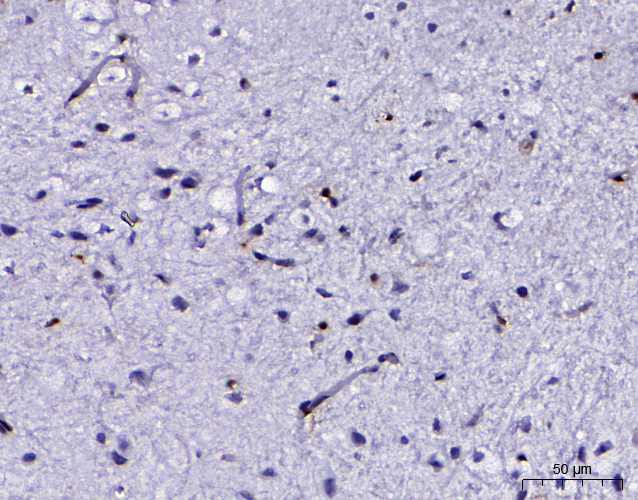

Supplement: Supplementary file 14 [file DataSheet6.ZIP › IHC Raw Image of P-CREB in PFC(1)/L7 1-200 PCREB_20.0x.tif-Q2.tif]

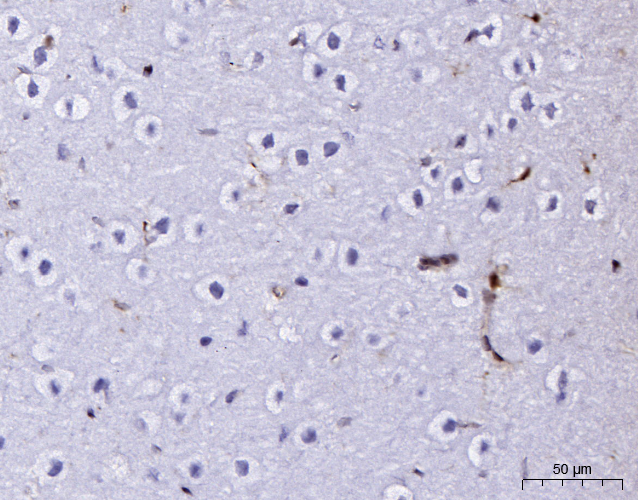

Supplement: Supplementary file 14 [file DataSheet6.ZIP › IHC Raw Image of P-CREB in PFC(1)/L9 1-200 PCREB_20.0x.tif-Q1.tif]

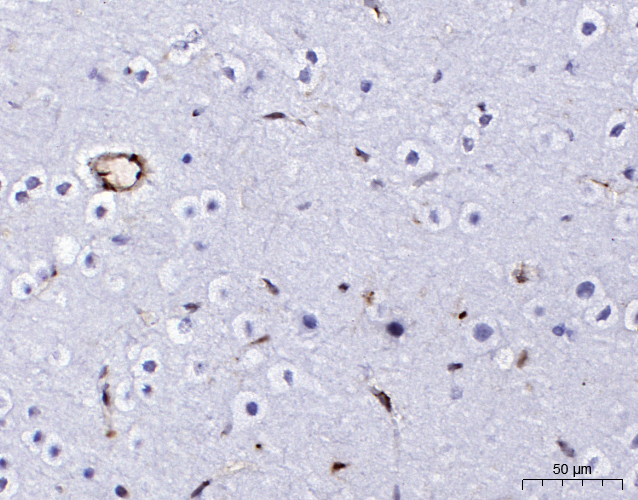

Supplement: Supplementary file 14 [file DataSheet6.ZIP › IHC Raw Image of P-CREB in PFC(1)/L9 1-200 PCREB_20.0x.tif-Q2.tif]

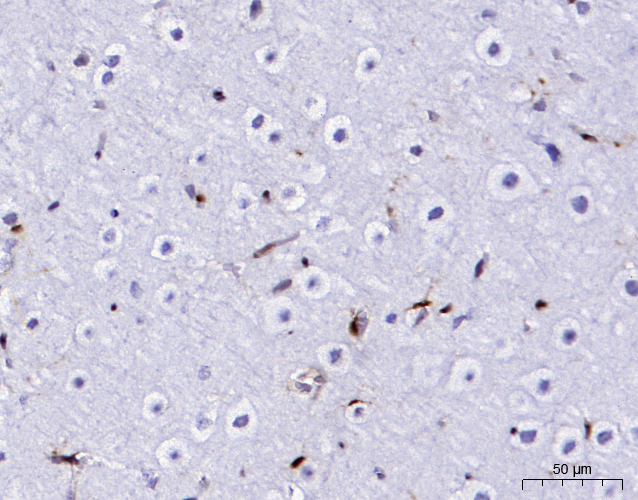

Supplement: Supplementary file 14 [file DataSheet6.ZIP › IHC Raw Image of P-CREB in PFC(1)/L9 1-200 PCREB_20.0x.tif-Q3.tif]

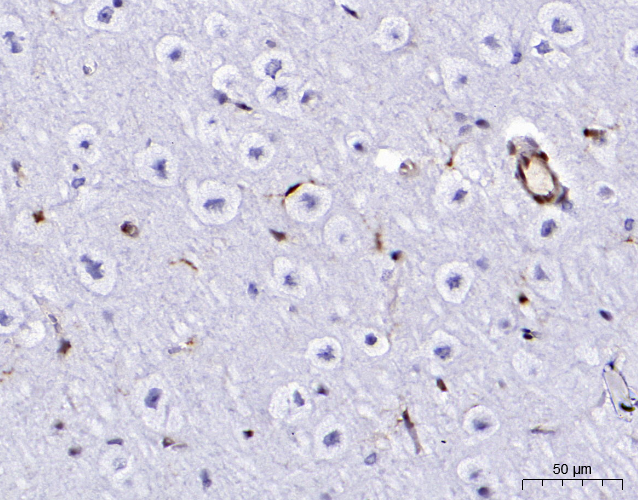

Supplement: Supplementary file 14 [file DataSheet6.ZIP › IHC Raw Image of P-CREB in PFC(1)/L9 1-200 PCREB_20.0x.tif-Q4.tif]

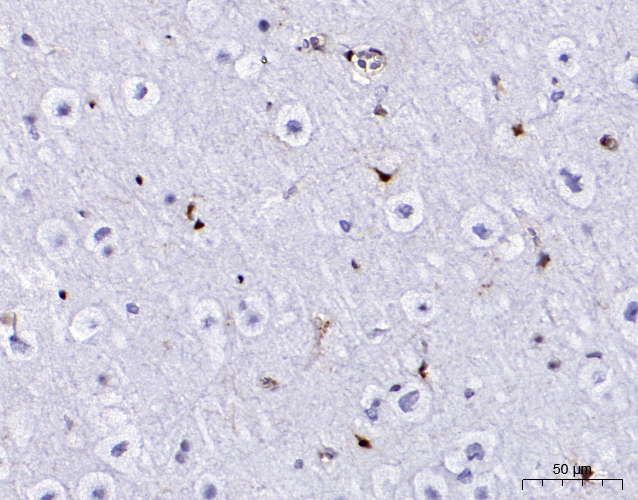

Supplement: Supplementary file 14 [file DataSheet6.ZIP › IHC Raw Image of P-CREB in PFC(1)/L9 1-200 PCREB_20.0x.tif-Q5.tif]

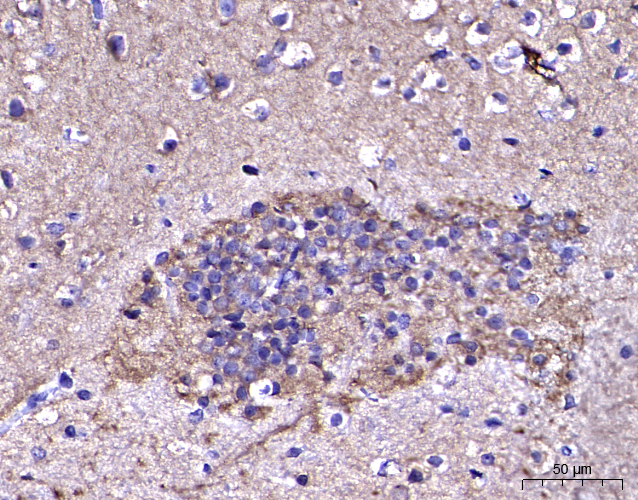

Supplement: Supplementary file 17 [file DataSheet12.ZIP › IHC Raew Image of PKA in striatum/H34 1-200 PKA_20.0x.tif-W3.tif]

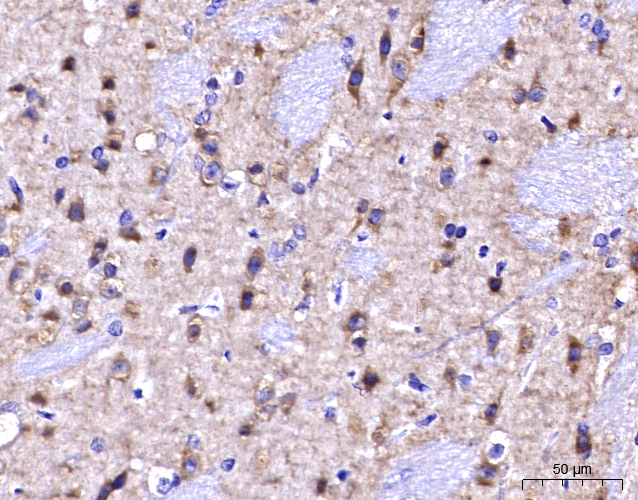

Supplement: Supplementary file 17 [file DataSheet12.ZIP › IHC Raew Image of PKA in striatum/H41 1-200 PKA_20.0x.tif-W1.tif]

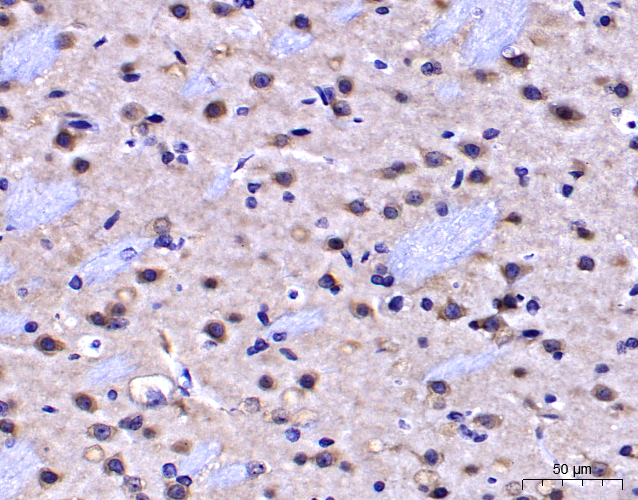

Supplement: Supplementary file 17 [file DataSheet12.ZIP › IHC Raew Image of PKA in striatum/H48 1-200 PKA_20.0x.tif-W2.tif]

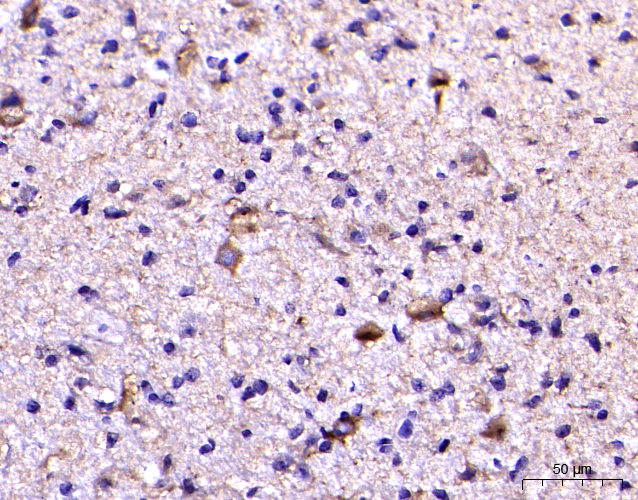

Supplement: Supplementary file 17 [file DataSheet12.ZIP › IHC Raew Image of PKA in striatum/H53 1-200 PKA_20.0x.tif-W2.tif]

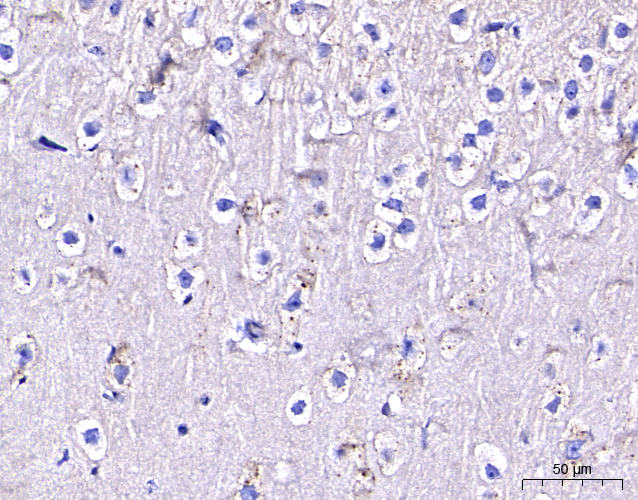

Supplement: Supplementary file 17 [file DataSheet12.ZIP › IHC Raew Image of PKA in striatum/K61 1-200 PKA_20.0x.tif-W1.tif]

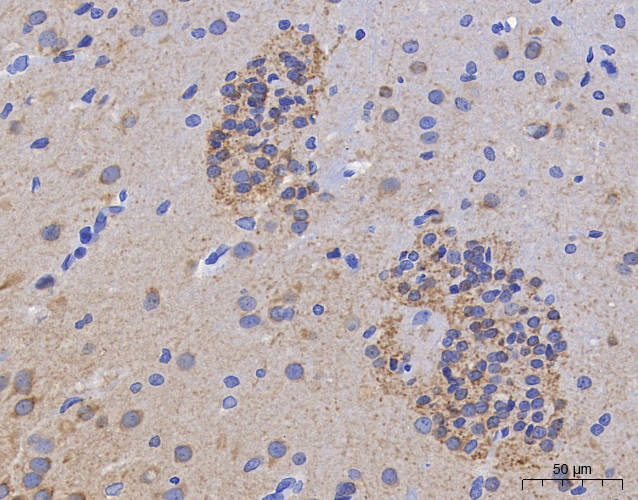

Supplement: Supplementary file 17 [file DataSheet12.ZIP › IHC Raew Image of PKA in striatum/K65 PKA_20.0x.tif-W5.tif]

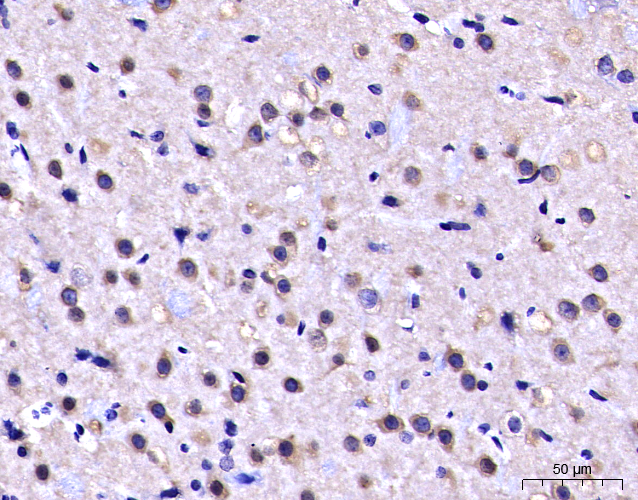

Supplement: Supplementary file 17 [file DataSheet12.ZIP › IHC Raew Image of PKA in striatum/K67 1-200 PKA_20.0x.tif-W1.tif]

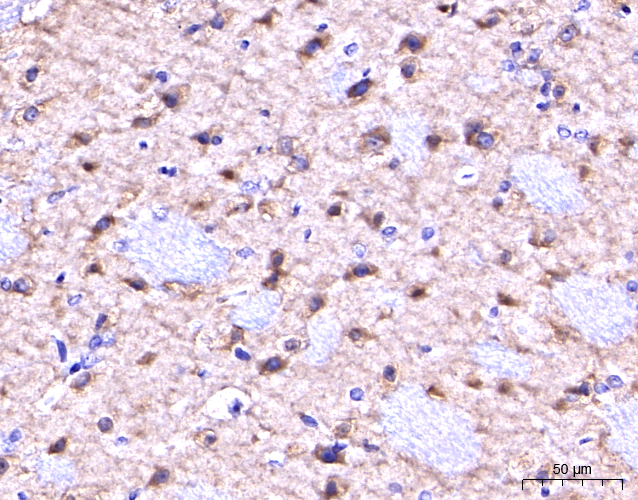

Supplement: Supplementary file 17 [file DataSheet12.ZIP › IHC Raew Image of PKA in striatum/K69 1-200 PKA_20.0x.tif-W5.tif]

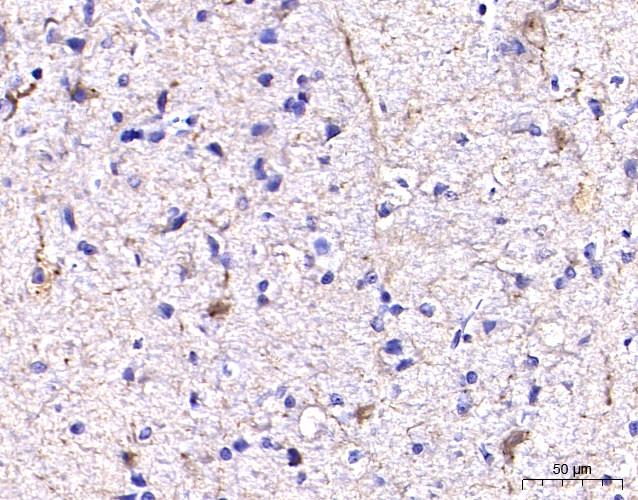

Supplement: Supplementary file 17 [file DataSheet12.ZIP › IHC Raew Image of PKA in striatum/L10 1-200 PKA_20.0x.tif-W4.tif]

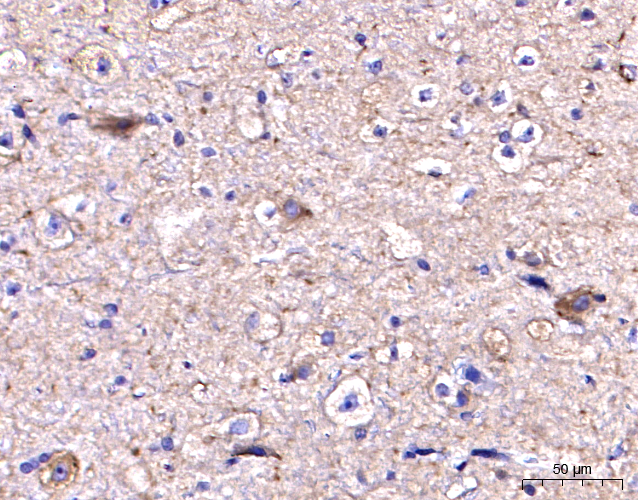

Supplement: Supplementary file 17 [file DataSheet12.ZIP › IHC Raew Image of PKA in striatum/L20 1-200 PKA_20.0x.tif-W1.tif]

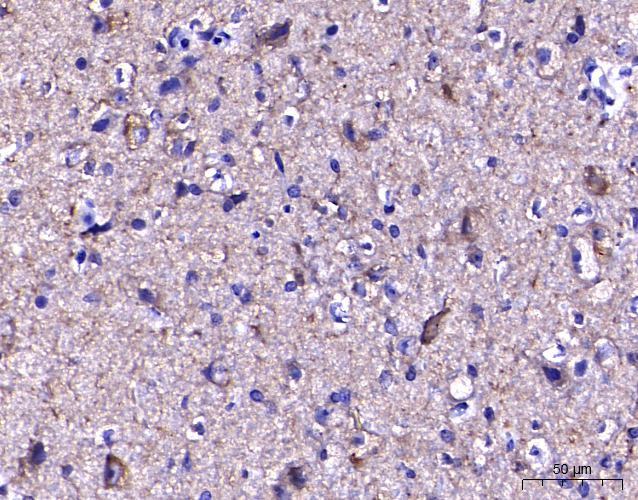

Supplement: Supplementary file 17 [file DataSheet12.ZIP › IHC Raew Image of PKA in striatum/L7 1-200 PKA_20.0x.tif-W5.tif]

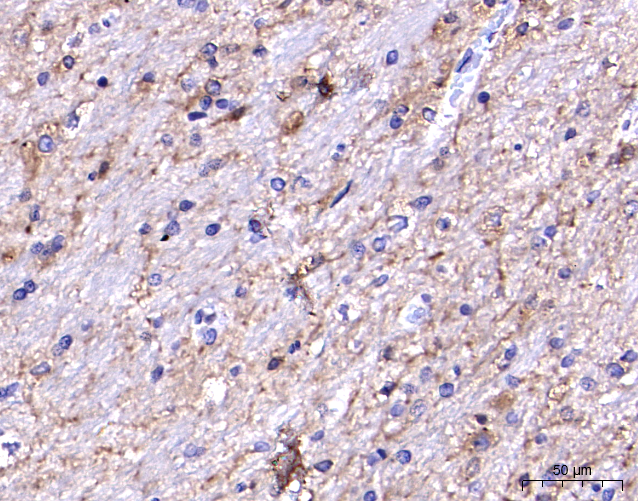

Supplement: Supplementary file 17 [file DataSheet12.ZIP › IHC Raew Image of PKA in striatum/L9 1-200 PKA_20.0x.tif-W5.tif]

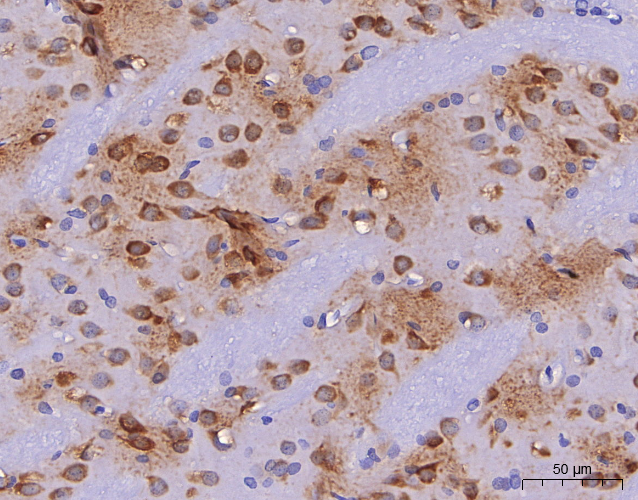

Supplement: Supplementary file 17 [file DataSheet12.ZIP › IHC Raew Image of PKA in striatum/M11 PKA_20.0x.tif-W1.tif]

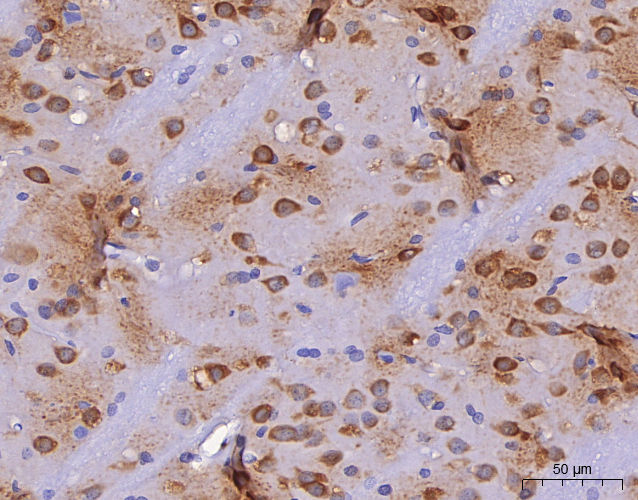

Supplement: Supplementary file 17 [file DataSheet12.ZIP › IHC Raew Image of PKA in striatum/M11 PKA_20.0x.tif-W2.tif]

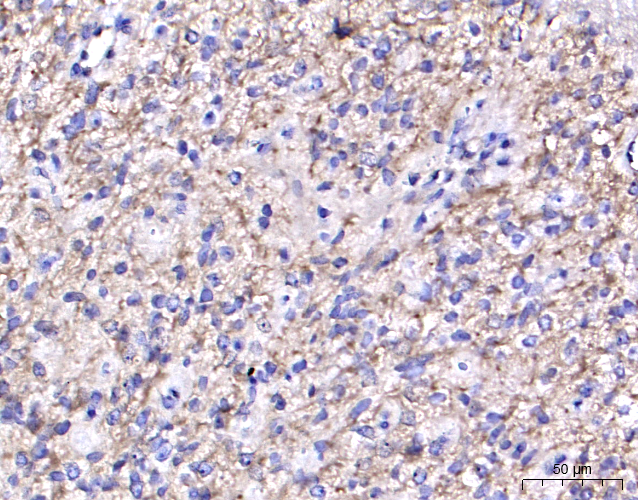

Supplement: Supplementary file 17 [file DataSheet12.ZIP › IHC Raew Image of PKA in striatum/M27 1-200 PKA_20.0x.tif-W5.tif]

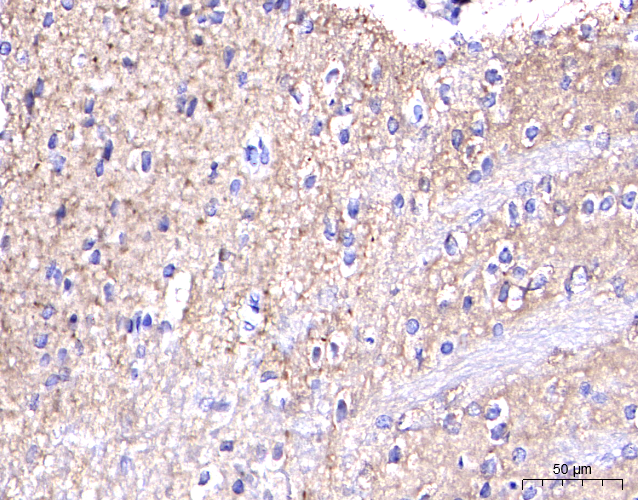

Supplement: Supplementary file 17 [file DataSheet12.ZIP › IHC Raew Image of PKA in striatum/M36 1-200 PKA_20.0x.tif-W4.tif]

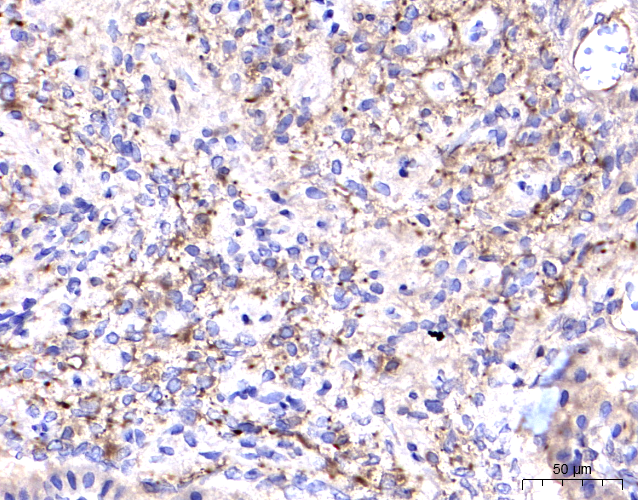

Supplement: Supplementary file 17 [file DataSheet12.ZIP › IHC Raew Image of PKA in striatum/M36 1-200 PKA_20.0x.tif-W5.tif]

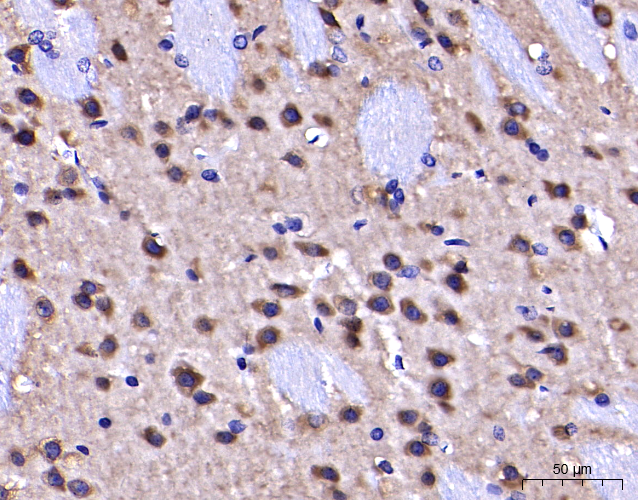

Supplement: Supplementary file 17 [file DataSheet12.ZIP › IHC Raew Image of PKA in striatum/M58 1-200 PKA_20.0x.tif-W1.tif]

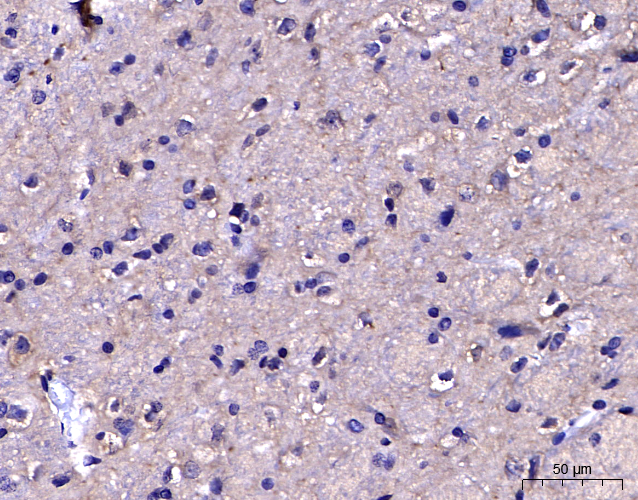

Supplement: Supplementary file 17 [file DataSheet12.ZIP › IHC Raew Image of PKA in striatum/MX1 1-200 PKA_20.0x.tif-W1.tif]

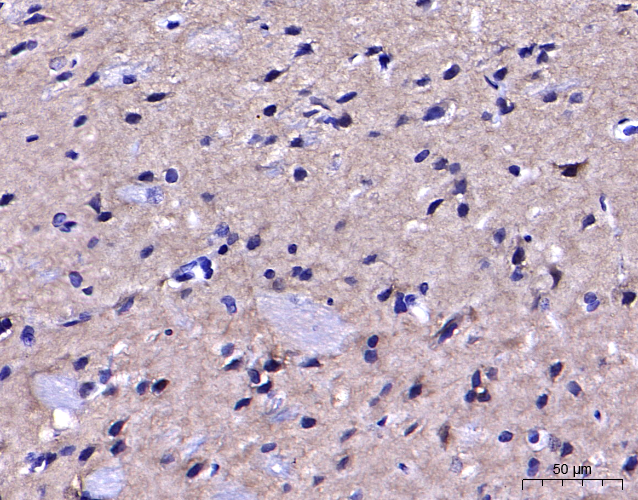

Supplement: Supplementary file 17 [file DataSheet12.ZIP › IHC Raew Image of PKA in striatum/MX1 1-200 PKA_20.0x.tif-W2.tif]

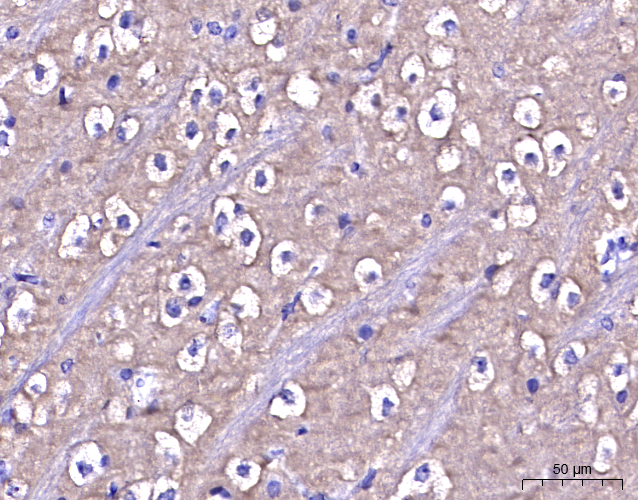

Supplement: Supplementary file 17 [file DataSheet12.ZIP › IHC Raew Image of PKA in striatum/MX18 1-200 PKA_20.0x.tif-W1.tif]

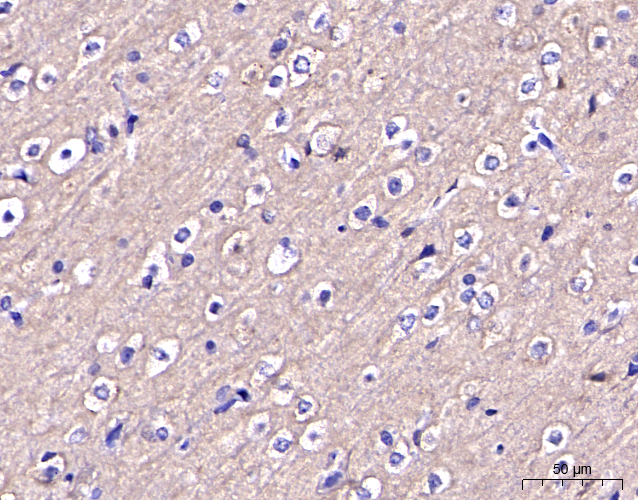

Supplement: Supplementary file 17 [file DataSheet12.ZIP › IHC Raew Image of PKA in striatum/MX18 1-200 PKA_20.0x.tif-W2.tif]

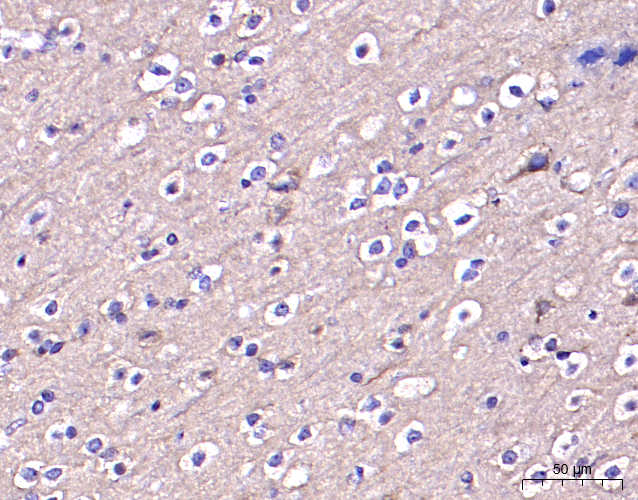

Supplement: Supplementary file 17 [file DataSheet12.ZIP › IHC Raew Image of PKA in striatum/MX21 1-200 PKA_20.0x.tif-W1.tif]

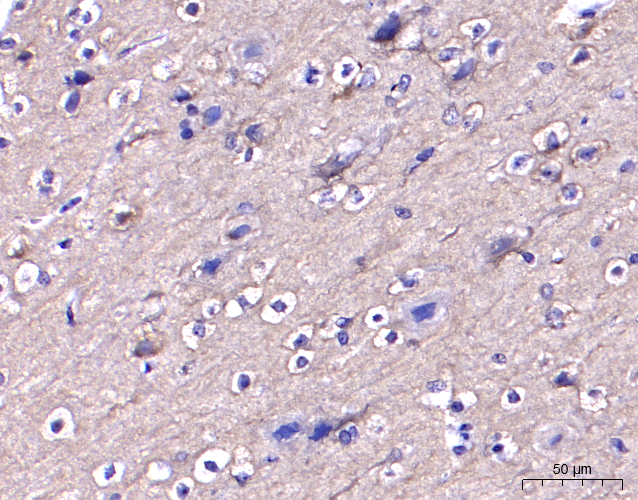

Supplement: Supplementary file 17 [file DataSheet12.ZIP › IHC Raew Image of PKA in striatum/MX21 1-200 PKA_20.0x.tif-W2.tif]

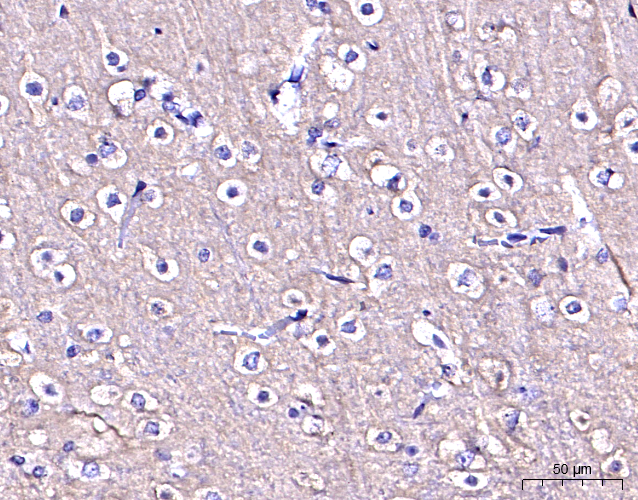

Supplement: Supplementary file 17 [file DataSheet12.ZIP › IHC Raew Image of PKA in striatum/MX5 ╬╞╫┤╠σ1-200 PKA_20.0x.tif-W2.tif]

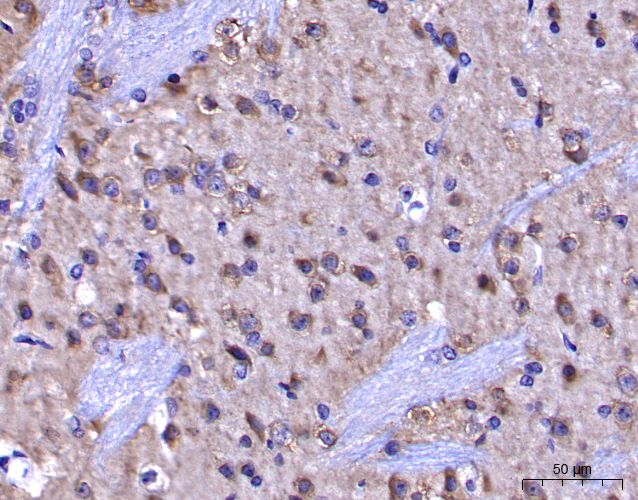

Supplement: Supplementary file 17 [file DataSheet12.ZIP › IHC Raew Image of PKA in striatum/Z23 1-200 PKA_20.0x.tif-W1.tif]

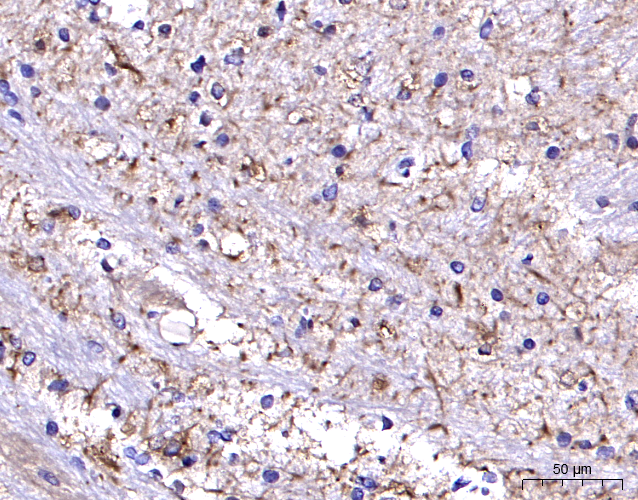

Supplement: Supplementary file 17 [file DataSheet12.ZIP › IHC Raew Image of PKA in striatum/Z46 1-200 PKA_20.0x.tif-W1.tif]

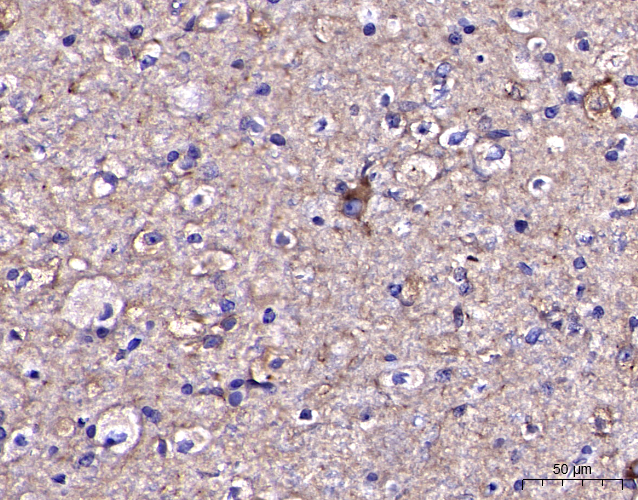

Supplement: Supplementary file 17 [file DataSheet12.ZIP › IHC Raew Image of PKA in striatum/Z46 1-200 PKA_20.0x.tif-W3.tif]

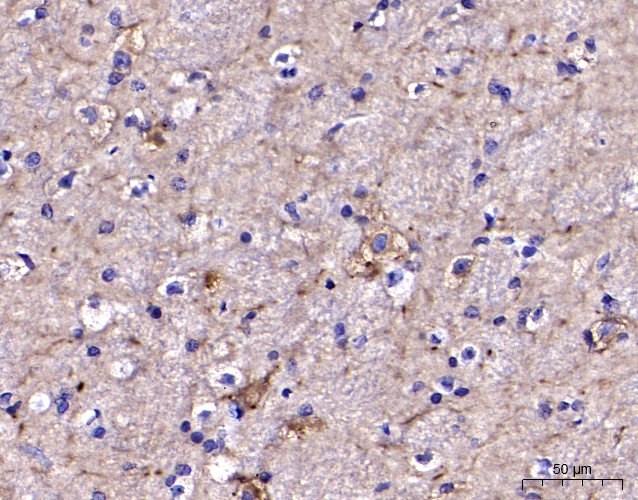

Supplement: Supplementary file 17 [file DataSheet12.ZIP › IHC Raew Image of PKA in striatum/Z46 1-200 PKA_20.0x.tif-W5.tif]

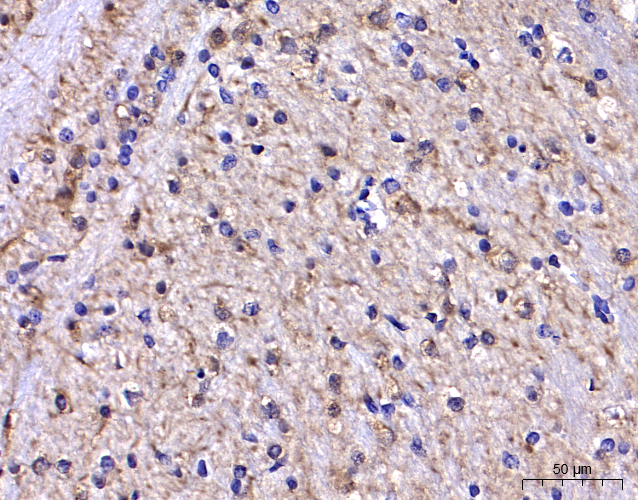

Supplement: Supplementary file 17 [file DataSheet12.ZIP › IHC Raew Image of PKA in striatum/Z47 1-200 PKA_20.0x.tif-W1.tif]

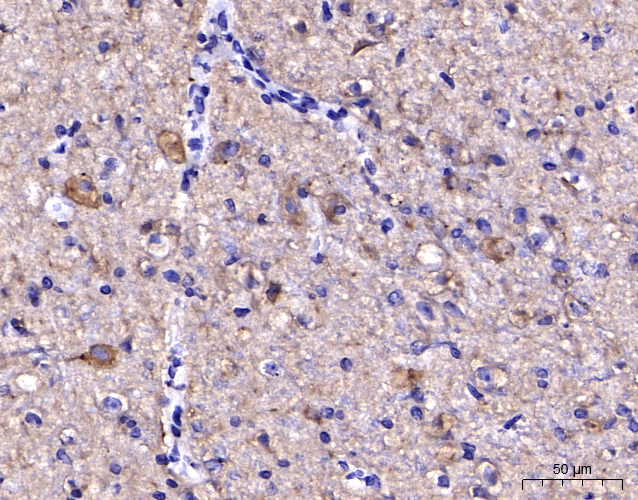

Supplement: Supplementary file 17 [file DataSheet12.ZIP › IHC Raew Image of PKA in striatum/Z47 1-200 PKA_20.0x.tif-W5.tif]

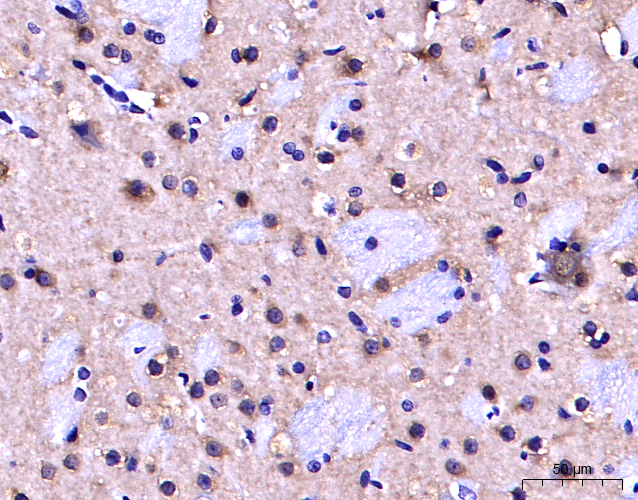

Supplement: Supplementary file 17 [file DataSheet12.ZIP › IHC Raew Image of PKA in striatum/Z56 1-200 PKA_20.0x.tif-W2.tif]

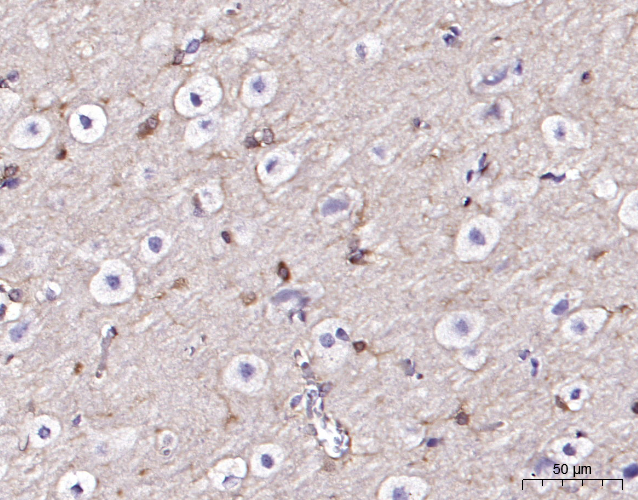

Supplement: Supplementary file 18 [file DataSheet2.ZIP › IHC Raw image of BDNF in PFC(2)/MX1 1-100 BDNF_20.0x.tif-Q1.tif]

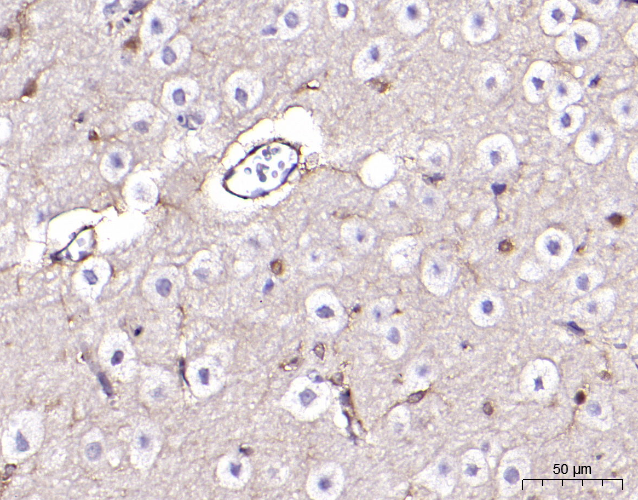

Supplement: Supplementary file 18 [file DataSheet2.ZIP › IHC Raw image of BDNF in PFC(2)/MX1 1-100 BDNF_20.0x.tif-Q2.tif]

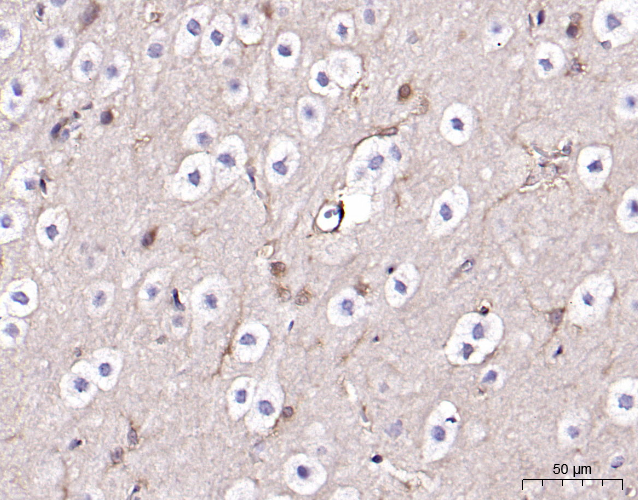

Supplement: Supplementary file 18 [file DataSheet2.ZIP › IHC Raw image of BDNF in PFC(2)/MX1 1-100 BDNF_20.0x.tif-Q3.tif]

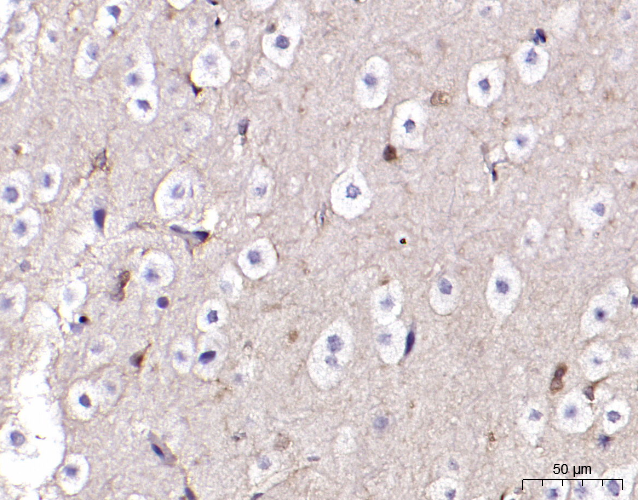

Supplement: Supplementary file 18 [file DataSheet2.ZIP › IHC Raw image of BDNF in PFC(2)/MX1 1-100 BDNF_20.0x.tif-Q4.tif]

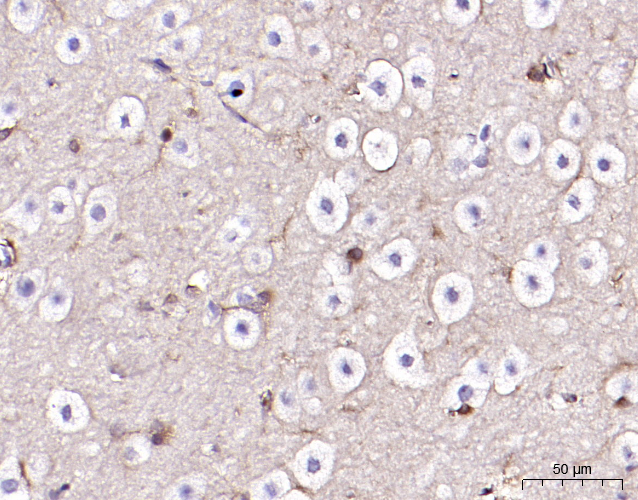

Supplement: Supplementary file 18 [file DataSheet2.ZIP › IHC Raw image of BDNF in PFC(2)/MX1 1-100 BDNF_20.0x.tif-Q5.tif]

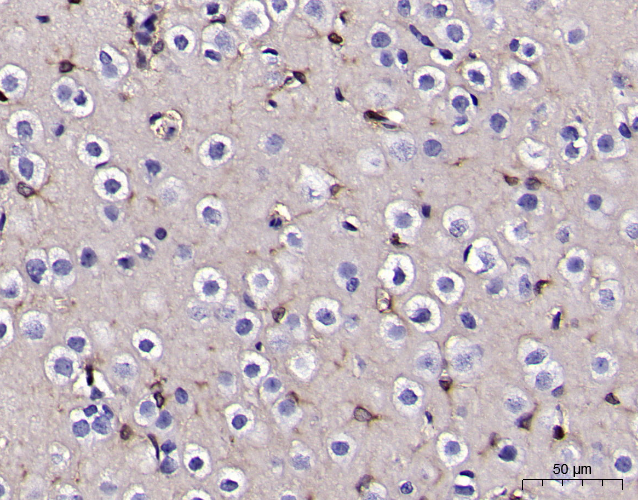

Supplement: Supplementary file 18 [file DataSheet2.ZIP › IHC Raw image of BDNF in PFC(2)/MX18 1-100 BDNF_20.0x.tif-Q1.tif]

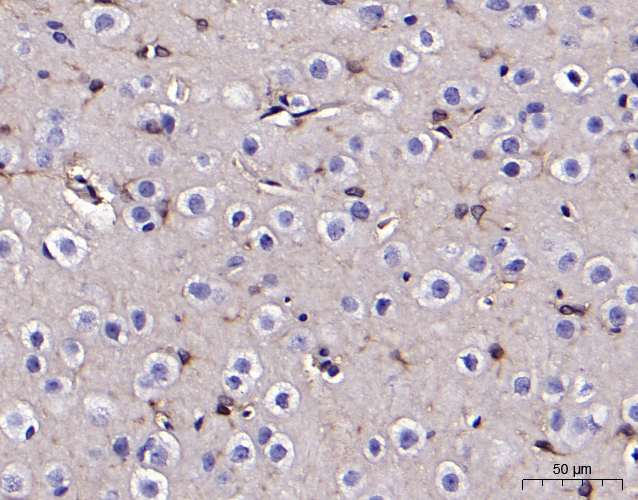

Supplement: Supplementary file 18 [file DataSheet2.ZIP › IHC Raw image of BDNF in PFC(2)/MX18 1-100 BDNF_20.0x.tif-Q2.tif]

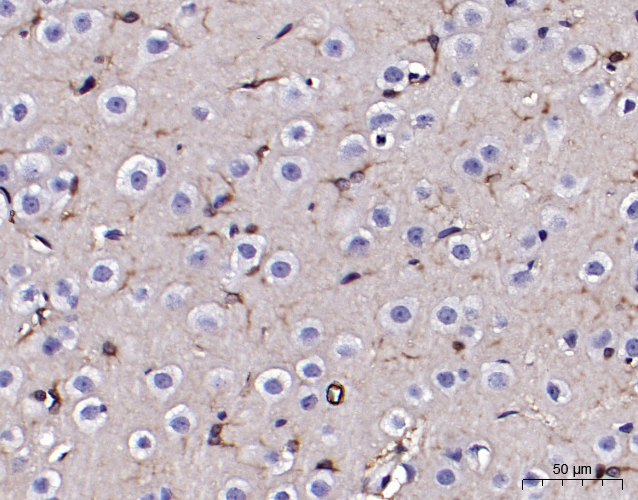

Supplement: Supplementary file 18 [file DataSheet2.ZIP › IHC Raw image of BDNF in PFC(2)/MX18 1-100 BDNF_20.0x.tif-Q3.tif]

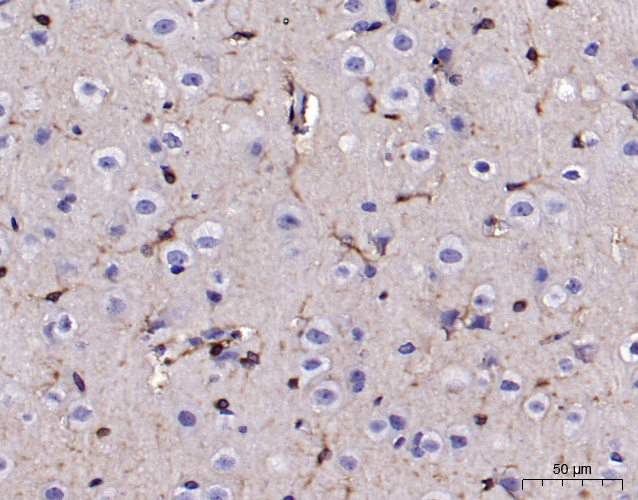

Supplement: Supplementary file 18 [file DataSheet2.ZIP › IHC Raw image of BDNF in PFC(2)/MX18 1-100 BDNF_20.0x.tif-Q4.tif]

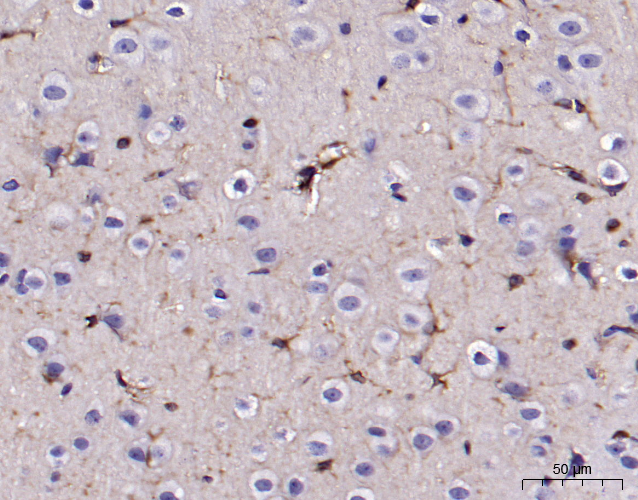

Supplement: Supplementary file 18 [file DataSheet2.ZIP › IHC Raw image of BDNF in PFC(2)/MX18 1-100 BDNF_20.0x.tif-Q5.tif]

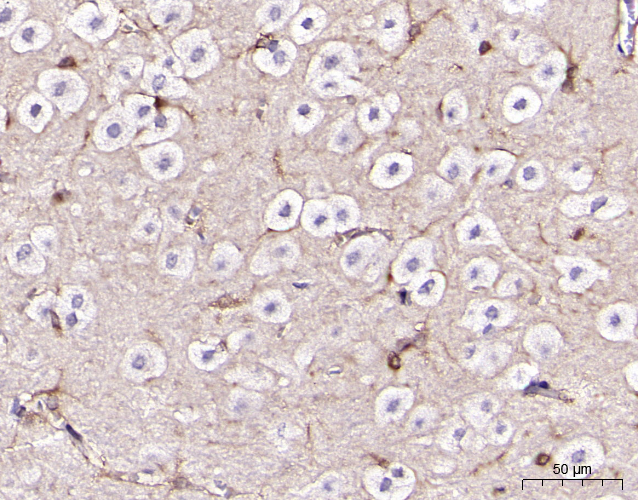

Supplement: Supplementary file 18 [file DataSheet2.ZIP › IHC Raw image of BDNF in PFC(2)/MX21 1-100 BDNF_20.0x.tif-Q1.tif]

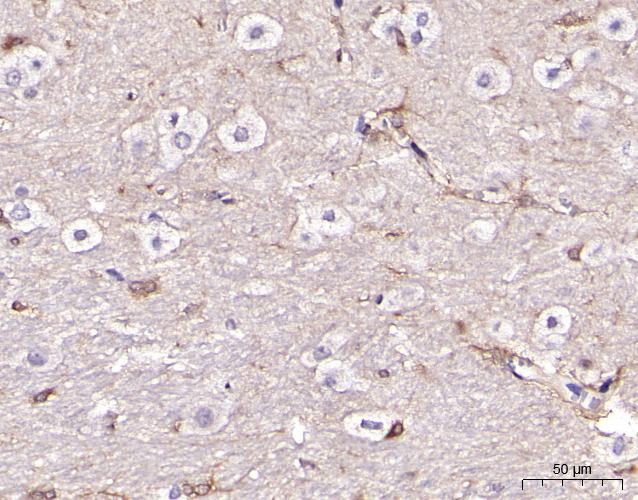

Supplement: Supplementary file 18 [file DataSheet2.ZIP › IHC Raw image of BDNF in PFC(2)/MX21 1-100 BDNF_20.0x.tif-Q2.tif]

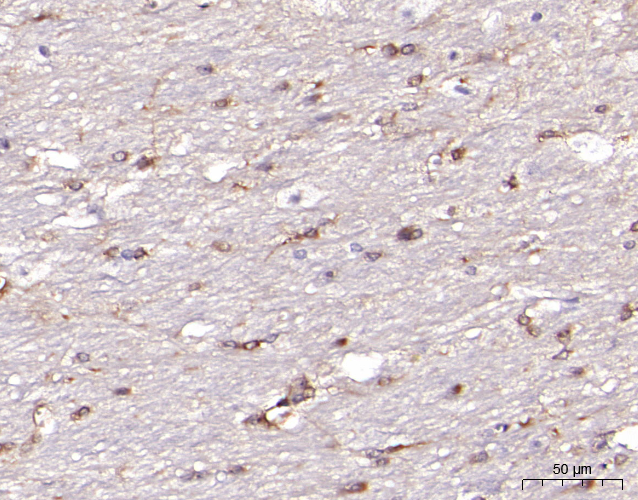

Supplement: Supplementary file 18 [file DataSheet2.ZIP › IHC Raw image of BDNF in PFC(2)/MX21 1-100 BDNF_20.0x.tif-Q3.tif]

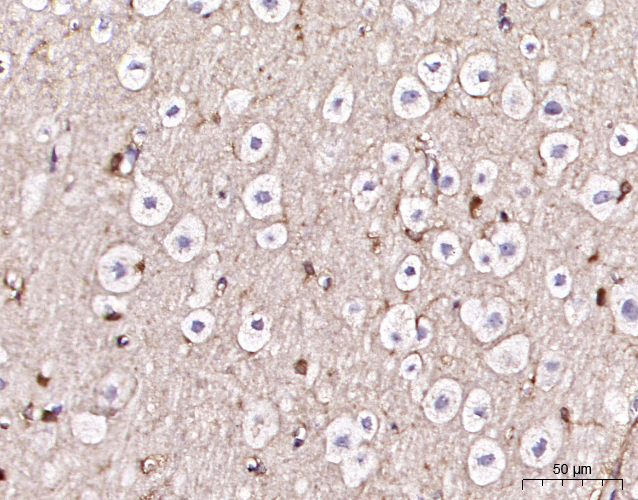

Supplement: Supplementary file 18 [file DataSheet2.ZIP › IHC Raw image of BDNF in PFC(2)/MX21 1-100 BDNF_20.0x.tif-Q4.tif]

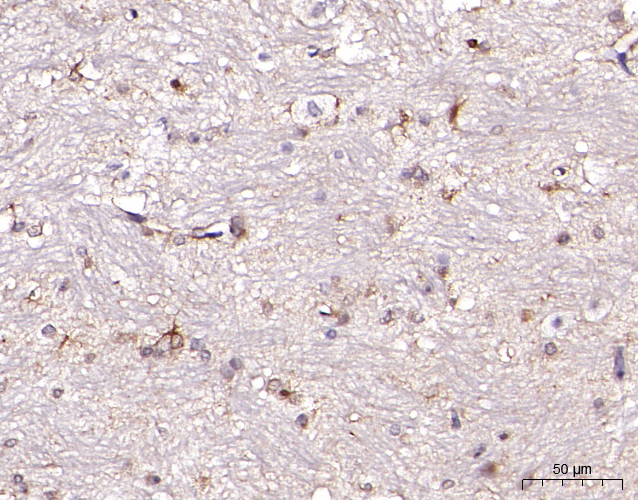

Supplement: Supplementary file 18 [file DataSheet2.ZIP › IHC Raw image of BDNF in PFC(2)/MX21 1-100 BDNF_20.0x.tif-Q5.tif]

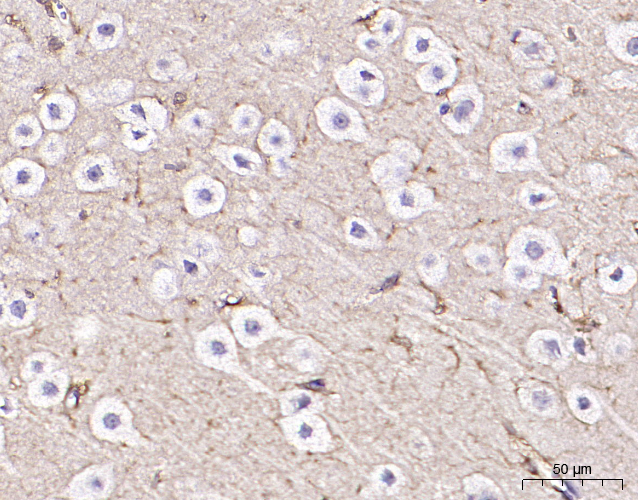

Supplement: Supplementary file 18 [file DataSheet2.ZIP › IHC Raw image of BDNF in PFC(2)/MX5 1-100 BDNF_20.0x.tif-Q1.tif]

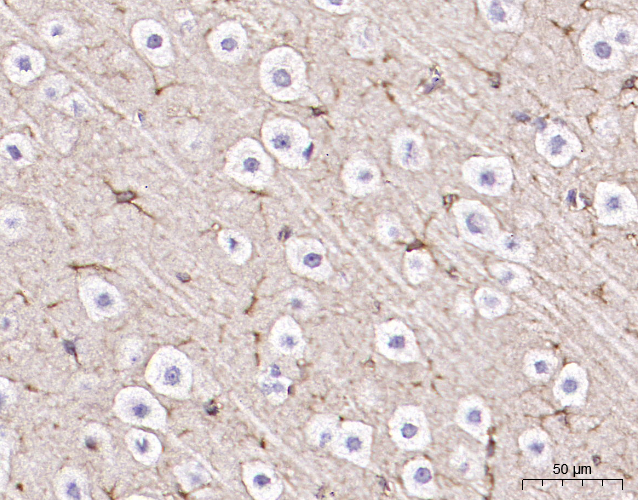

Supplement: Supplementary file 18 [file DataSheet2.ZIP › IHC Raw image of BDNF in PFC(2)/MX5 1-100 BDNF_20.0x.tif-Q2.tif]

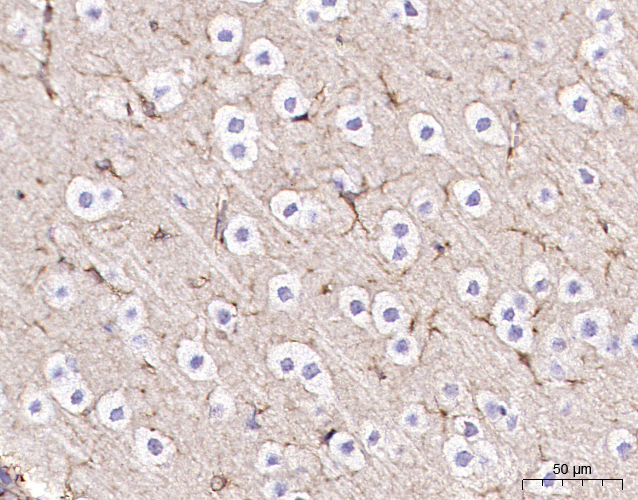

Supplement: Supplementary file 18 [file DataSheet2.ZIP › IHC Raw image of BDNF in PFC(2)/MX5 1-100 BDNF_20.0x.tif-Q3.tif]

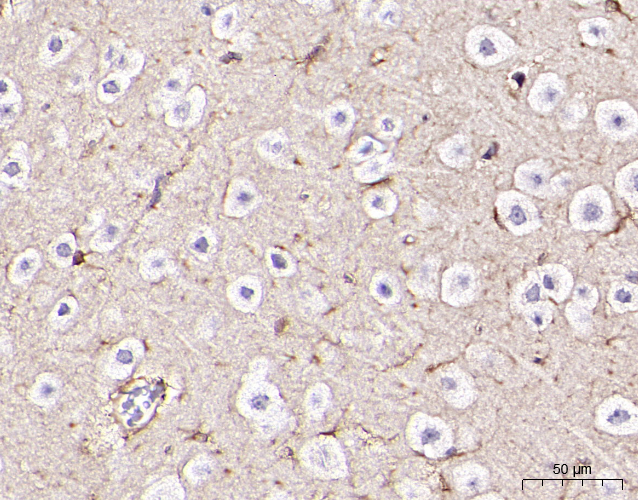

Supplement: Supplementary file 18 [file DataSheet2.ZIP › IHC Raw image of BDNF in PFC(2)/MX5 1-100 BDNF_20.0x.tif-Q4.tif]

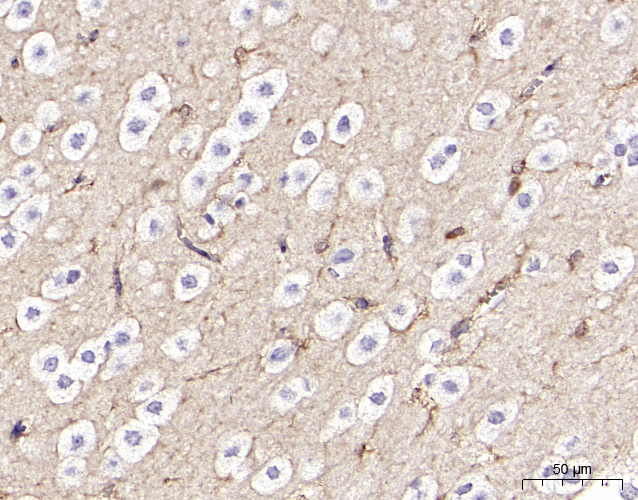

Supplement: Supplementary file 18 [file DataSheet2.ZIP › IHC Raw image of BDNF in PFC(2)/MX5 1-100 BDNF_20.0x.tif-Q5.tif]

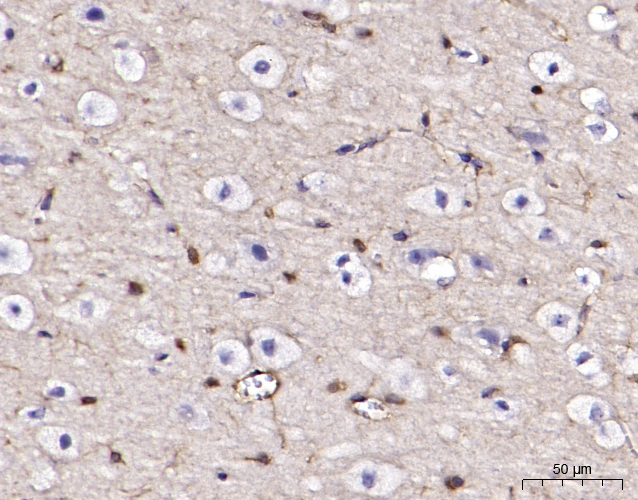

Supplement: Supplementary file 18 [file DataSheet2.ZIP › IHC Raw image of BDNF in PFC(2)/Z23 1-100 BDNF_20.0x.tif-Q1.tif]

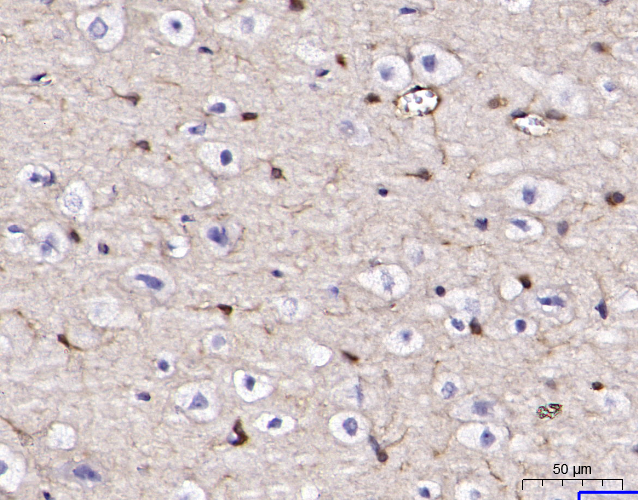

Supplement: Supplementary file 18 [file DataSheet2.ZIP › IHC Raw image of BDNF in PFC(2)/Z23 1-100 BDNF_20.0x.tif-Q2.tif]
