# Supplementary figures and images for: Long Mu Qing Xin mixture improves behavioral performance in spontaneously hypertensive rats (SHR/NCrl) by upregulating catecholamine neurotransmitters in prefrontal cortex and striatum via DRD1/cAMP/PKA-CREB signaling pathway (part 4 of 4)
Source: Front Pharmacol. 2024 Jul 4;15:1387359. doi: 10.3389/fphar.2024.1387359 (PMC11254830; doi:10.3389/fphar.2024.1387359)

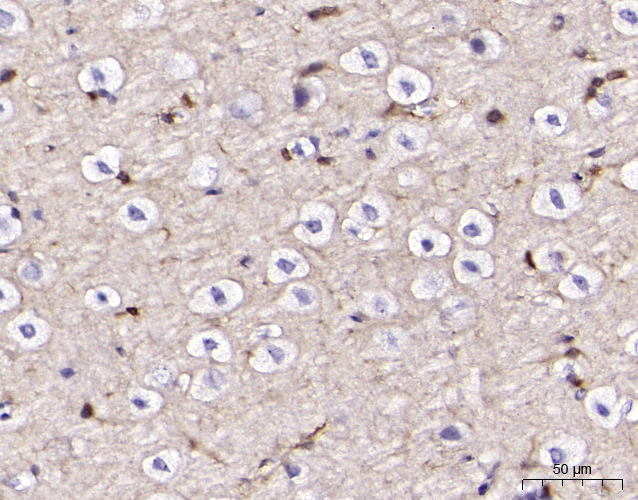

Supplement: Supplementary file 18 [file DataSheet2.ZIP › IHC Raw image of BDNF in PFC(2)/Z23 1-100 BDNF_20.0x.tif-Q3.tif]

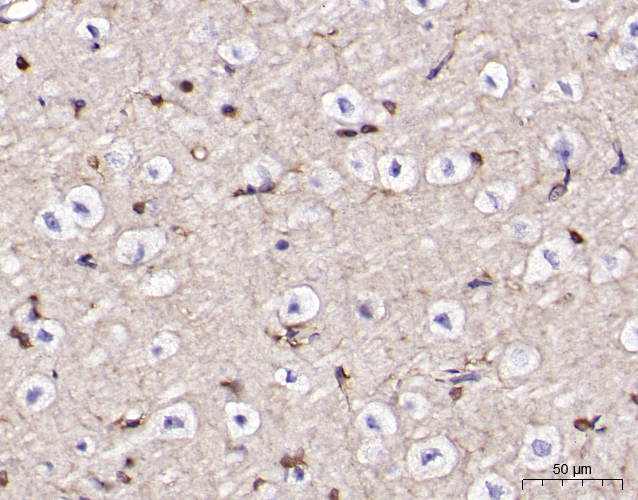

Supplement: Supplementary file 18 [file DataSheet2.ZIP › IHC Raw image of BDNF in PFC(2)/Z23 1-100 BDNF_20.0x.tif-Q4.tif]

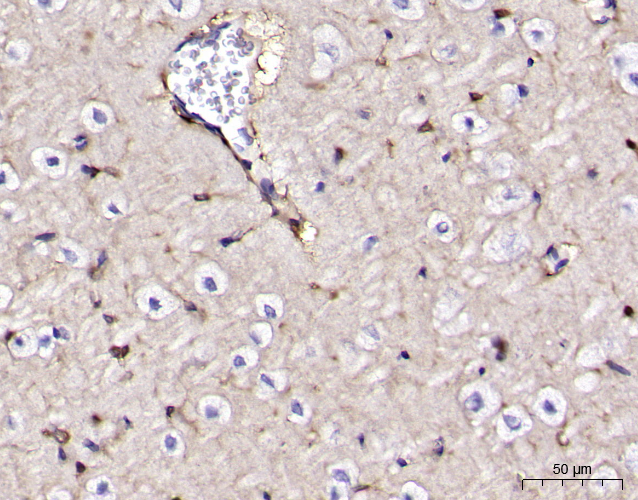

Supplement: Supplementary file 18 [file DataSheet2.ZIP › IHC Raw image of BDNF in PFC(2)/Z23 1-100 BDNF_20.0x.tif-Q5.tif]

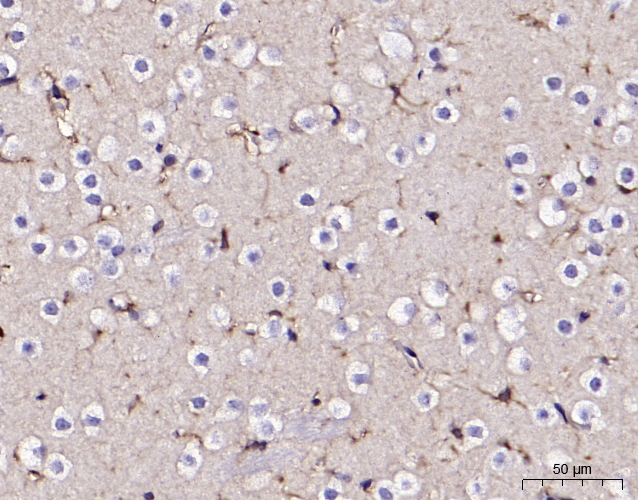

Supplement: Supplementary file 18 [file DataSheet2.ZIP › IHC Raw image of BDNF in PFC(2)/Z46 1-100 BDNF_20.0x.jpg-Q4.jpg]

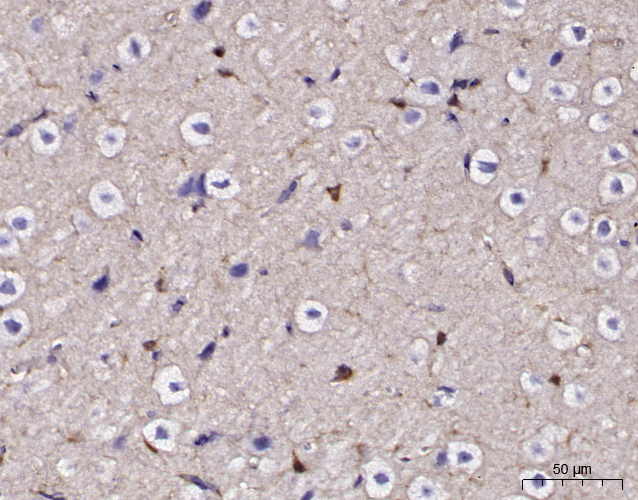

Supplement: Supplementary file 18 [file DataSheet2.ZIP › IHC Raw image of BDNF in PFC(2)/Z46 1-100 BDNF_20.0x.tif-Q1.tif]

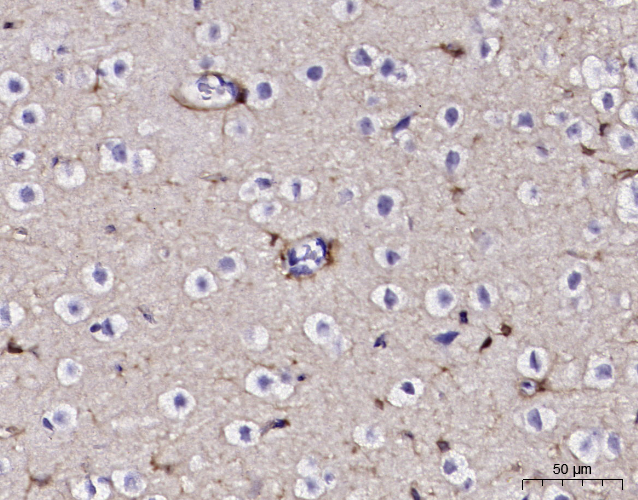

Supplement: Supplementary file 18 [file DataSheet2.ZIP › IHC Raw image of BDNF in PFC(2)/Z46 1-100 BDNF_20.0x.tif-Q2.tif]

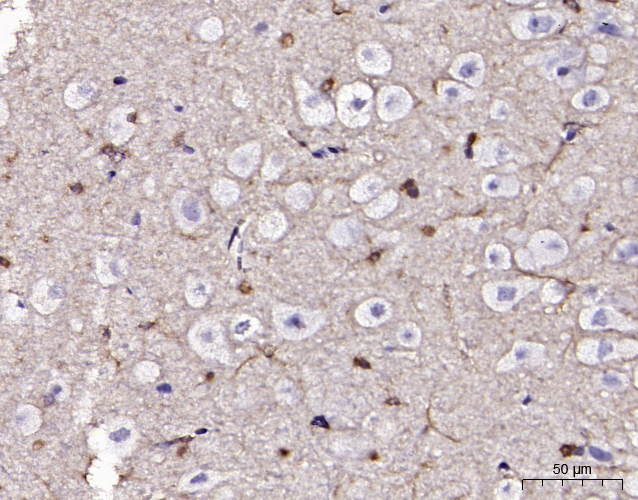

Supplement: Supplementary file 18 [file DataSheet2.ZIP › IHC Raw image of BDNF in PFC(2)/Z46 1-100 BDNF_20.0x.tif-Q3.tif]

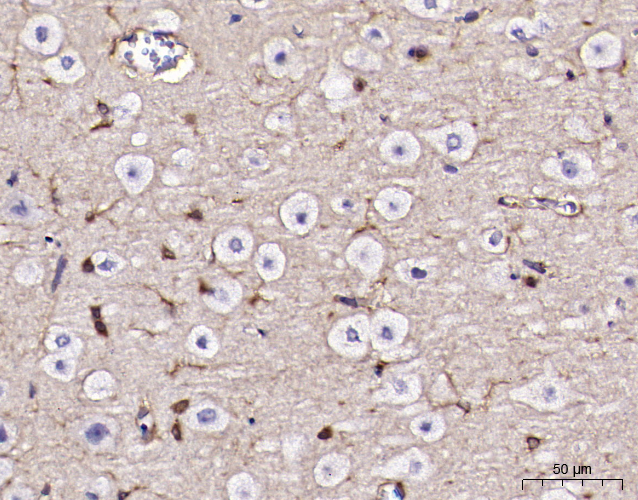

Supplement: Supplementary file 18 [file DataSheet2.ZIP › IHC Raw image of BDNF in PFC(2)/Z46 1-100 BDNF_20.0x.tif-Q4.tif]

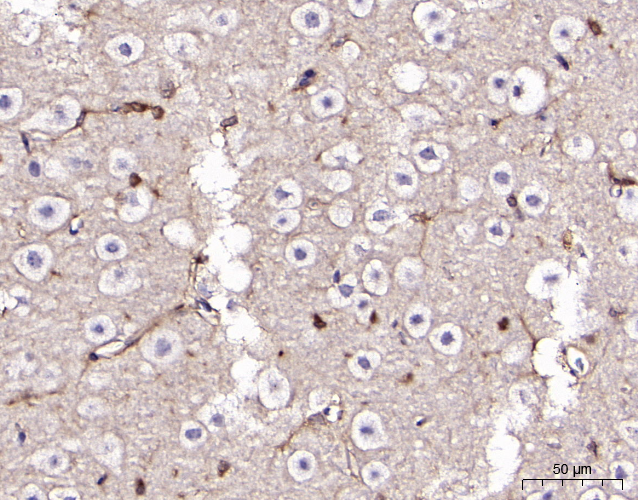

Supplement: Supplementary file 18 [file DataSheet2.ZIP › IHC Raw image of BDNF in PFC(2)/Z46 1-100 BDNF_20.0x.tif-Q5.tif]

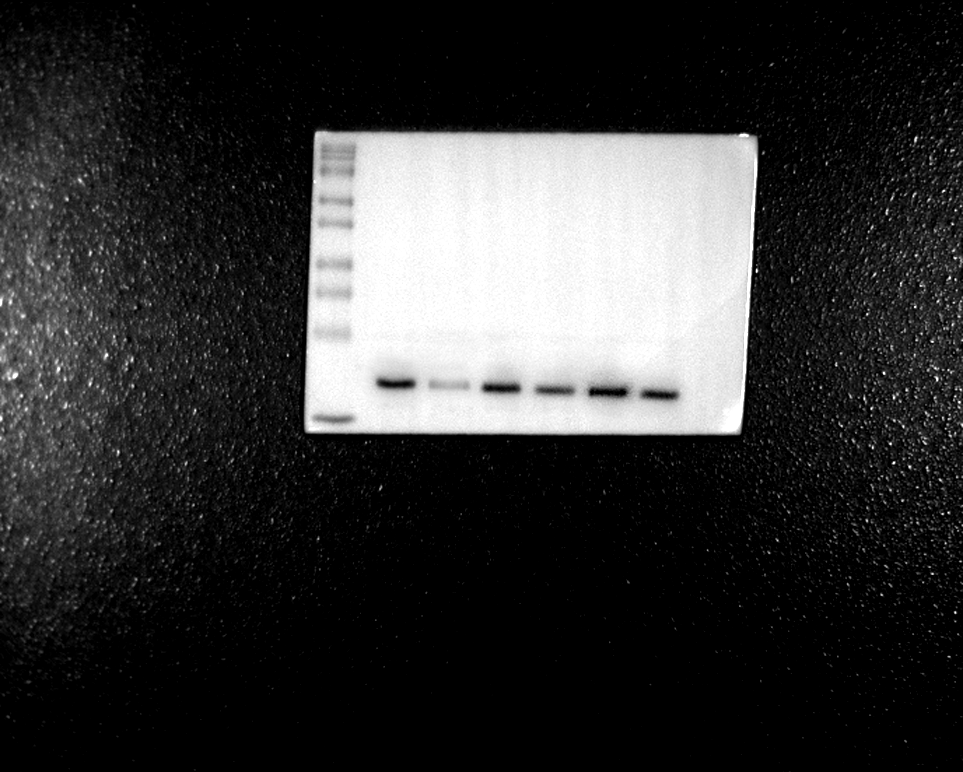

Supplement: Supplementary file 19 [file DataSheet15.ZIP › Raw image of Western blot in Striatum/BDNF (1).tif]

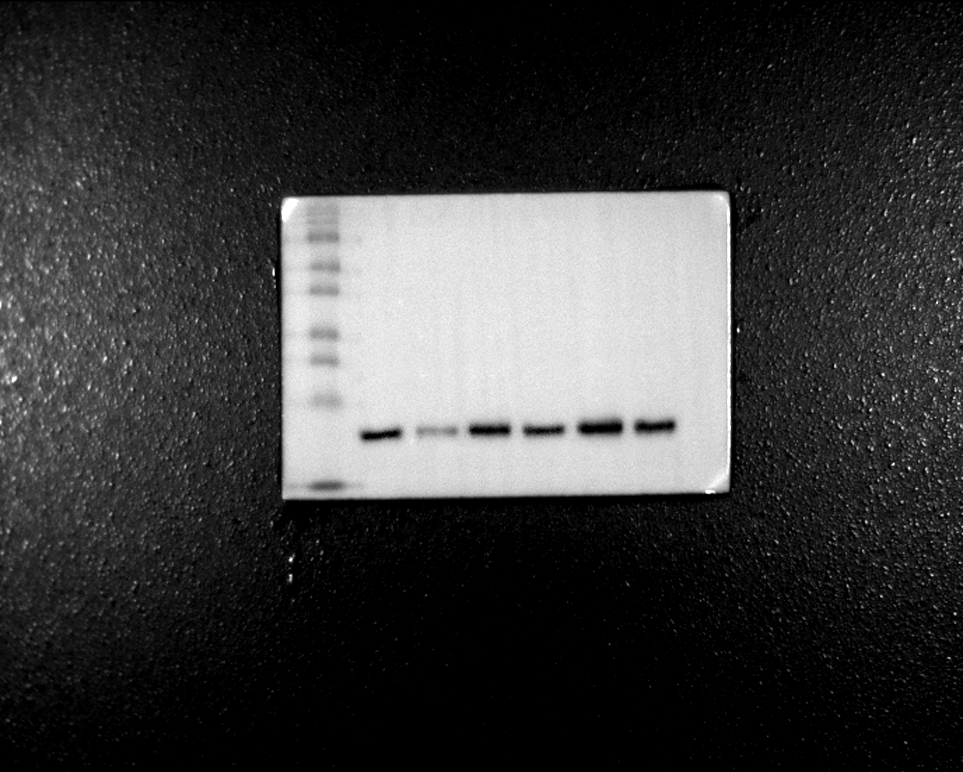

Supplement: Supplementary file 19 [file DataSheet15.ZIP › Raw image of Western blot in Striatum/BDNF (2).tif]

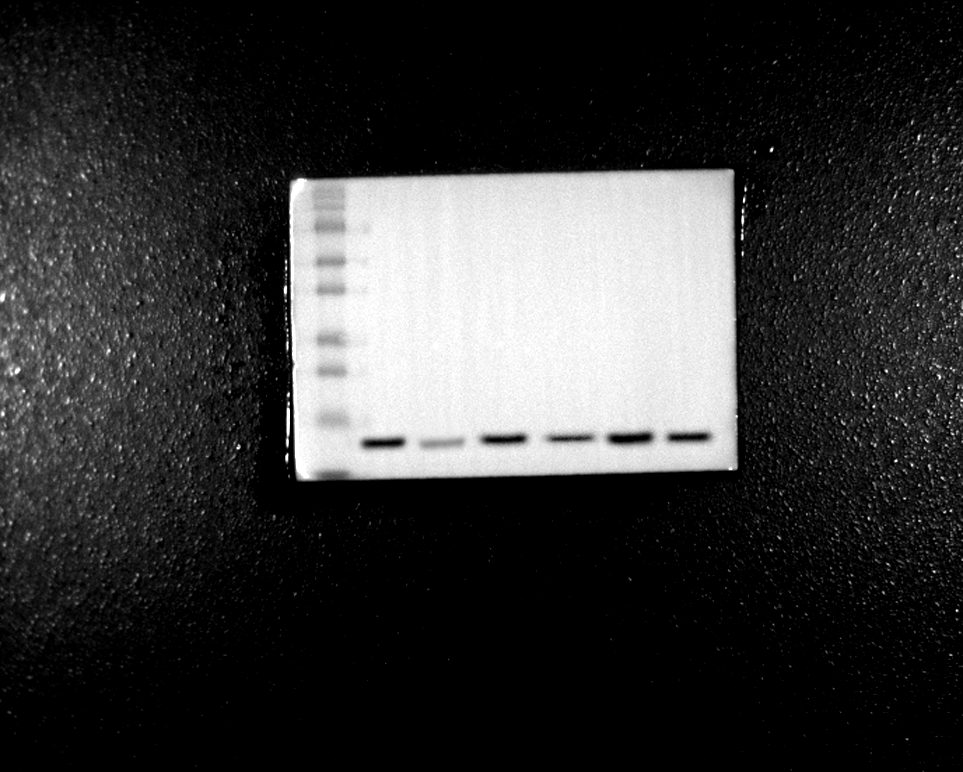

Supplement: Supplementary file 19 [file DataSheet15.ZIP › Raw image of Western blot in Striatum/BDNF (3).tif]

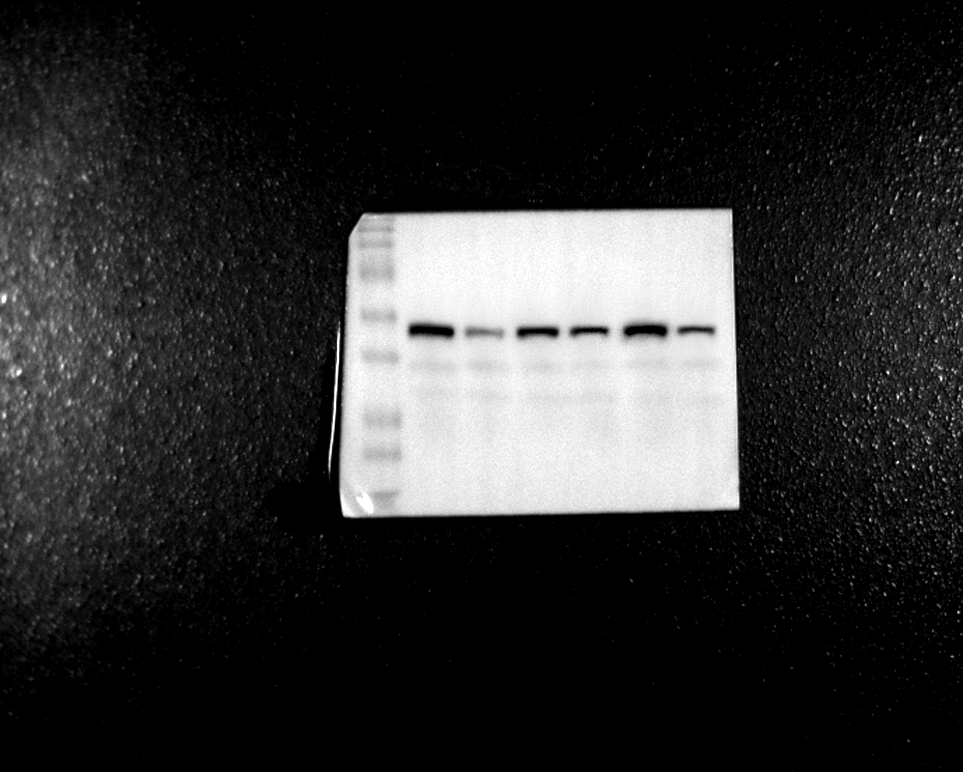

Supplement: Supplementary file 19 [file DataSheet15.ZIP › Raw image of Western blot in Striatum/DRD1 (1).tif]

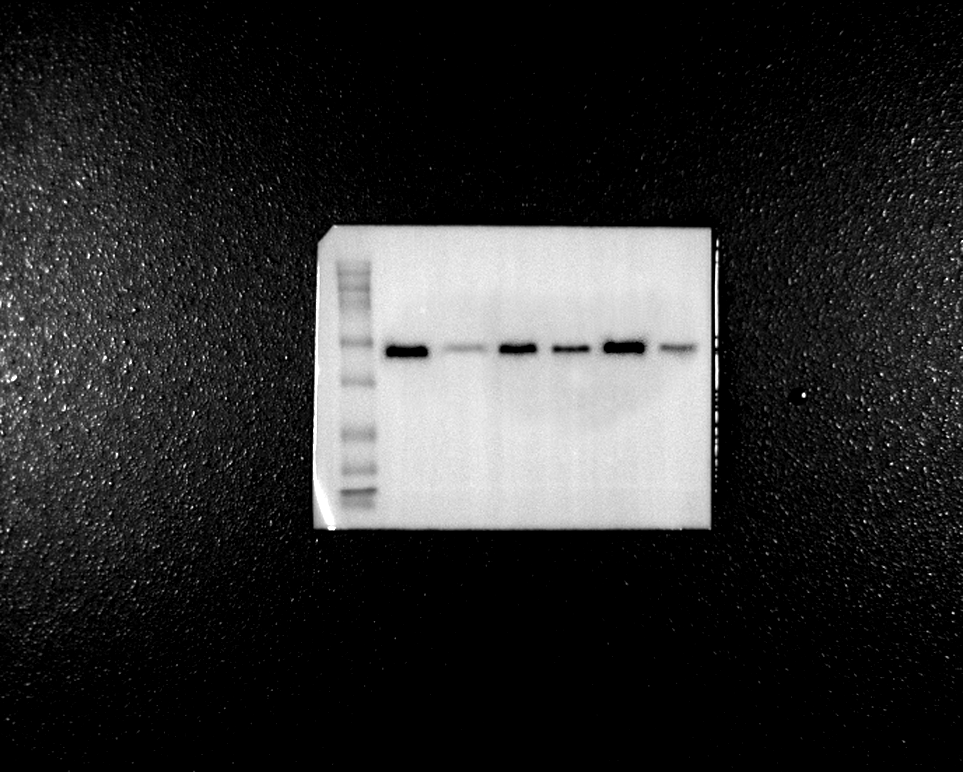

Supplement: Supplementary file 19 [file DataSheet15.ZIP › Raw image of Western blot in Striatum/DRD1 (2).tif]

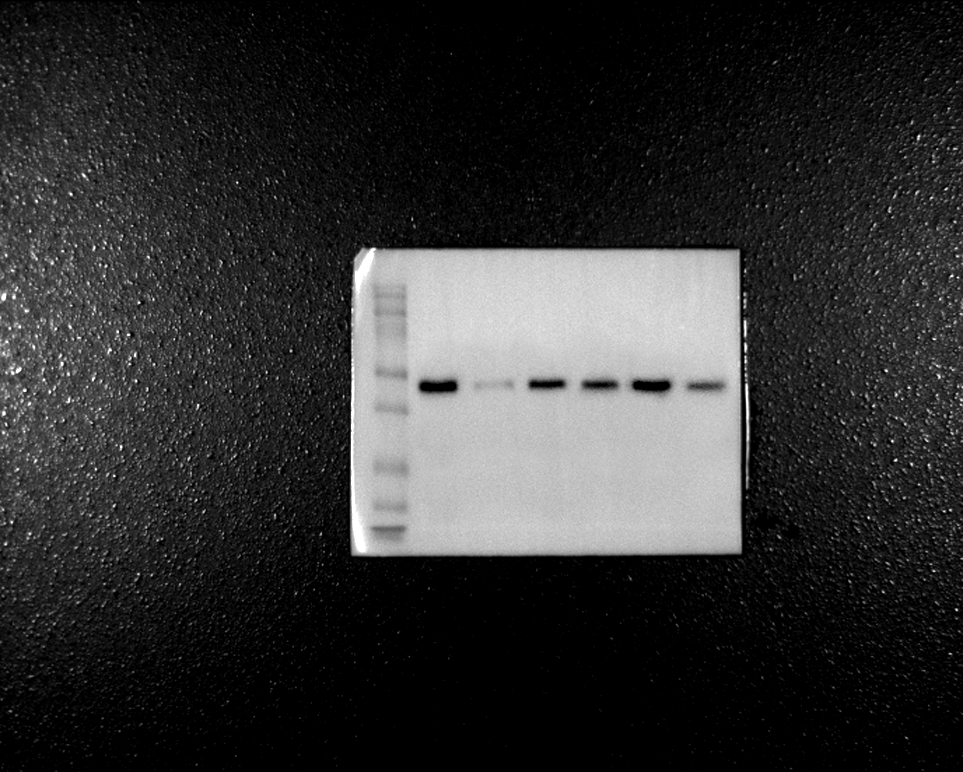

Supplement: Supplementary file 19 [file DataSheet15.ZIP › Raw image of Western blot in Striatum/DRD1 (3).tif]

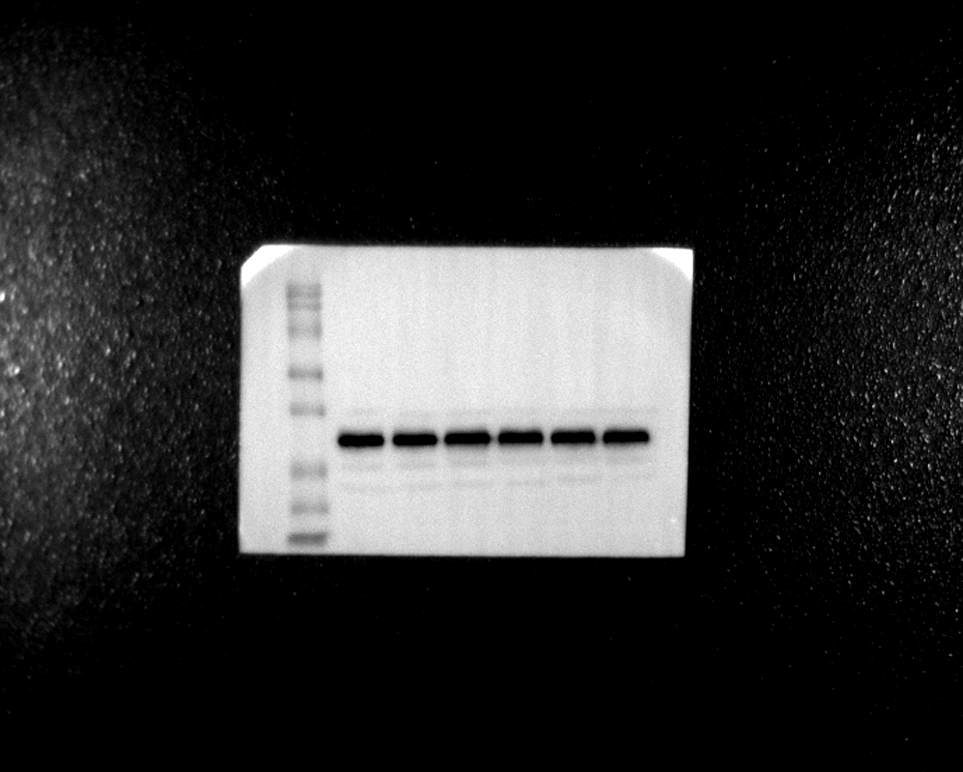

Supplement: Supplementary file 19 [file DataSheet15.ZIP › Raw image of Western blot in Striatum/GAPDH (1).tif]

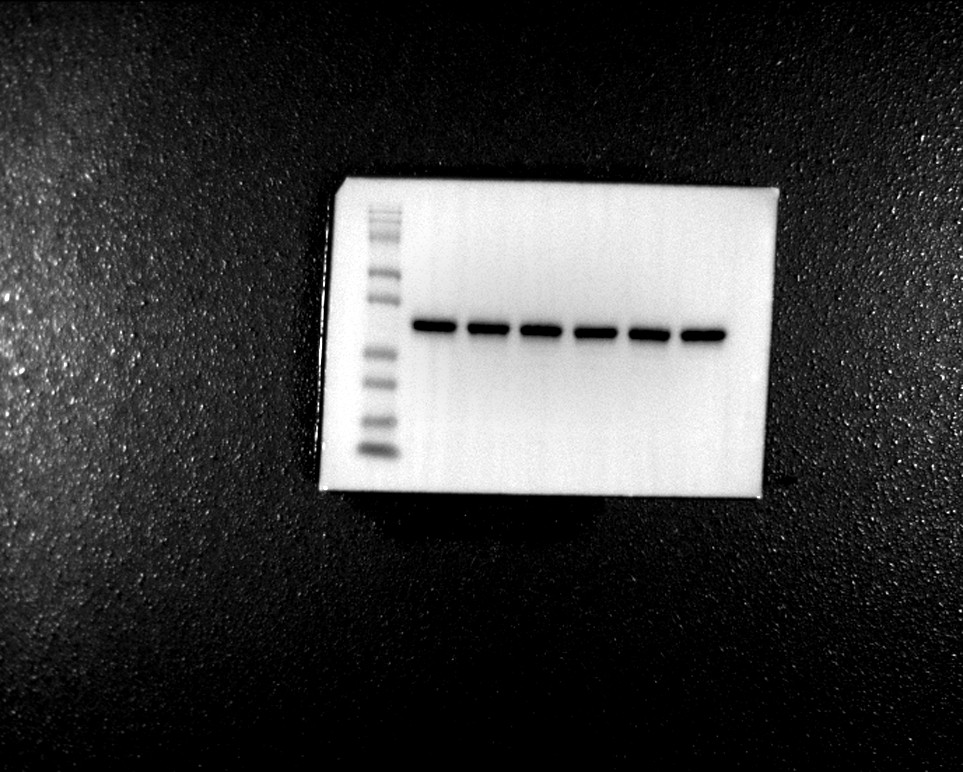

Supplement: Supplementary file 19 [file DataSheet15.ZIP › Raw image of Western blot in Striatum/GAPDH (2).tif]

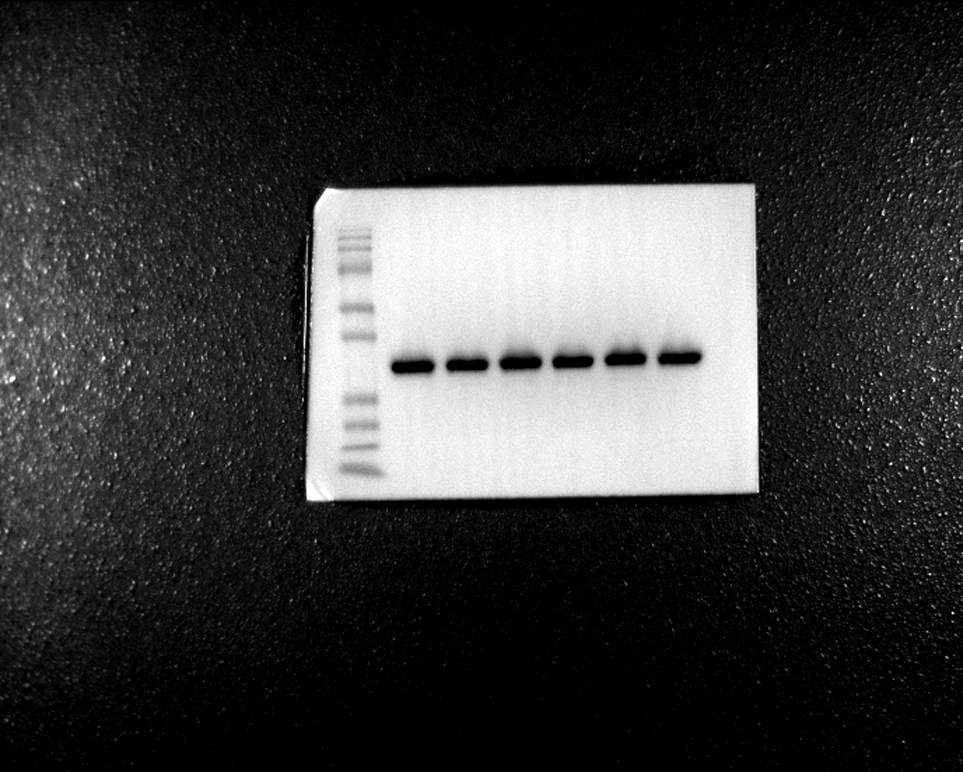

Supplement: Supplementary file 19 [file DataSheet15.ZIP › Raw image of Western blot in Striatum/GAPDH (3).tif]

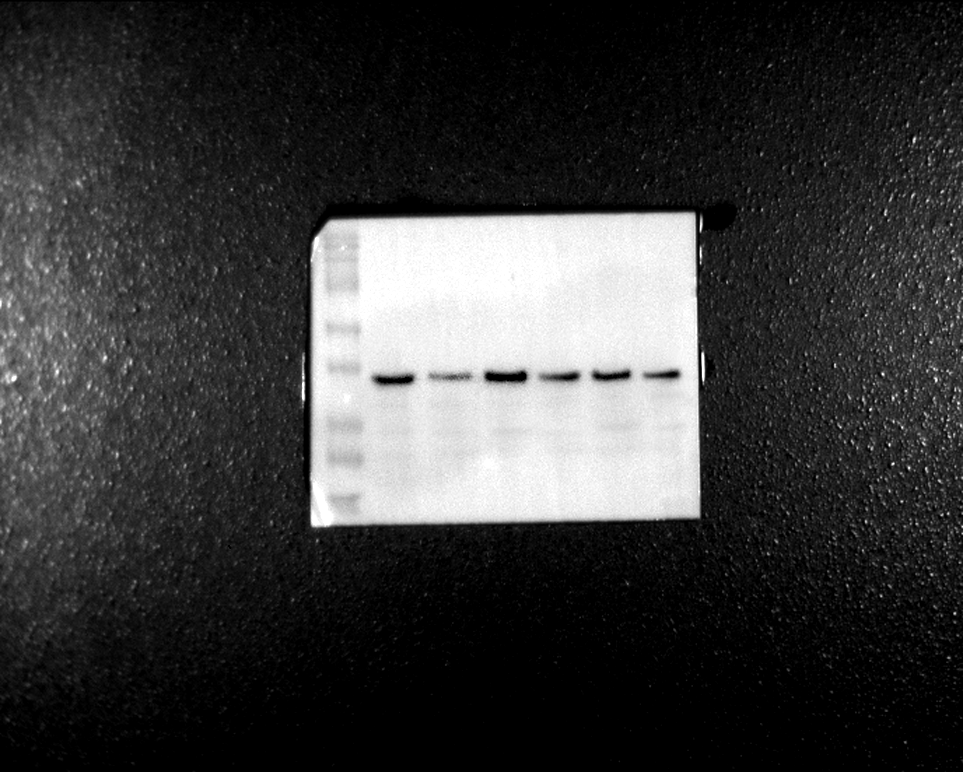

Supplement: Supplementary file 19 [file DataSheet15.ZIP › Raw image of Western blot in Striatum/Ga┴olf (1).tif]

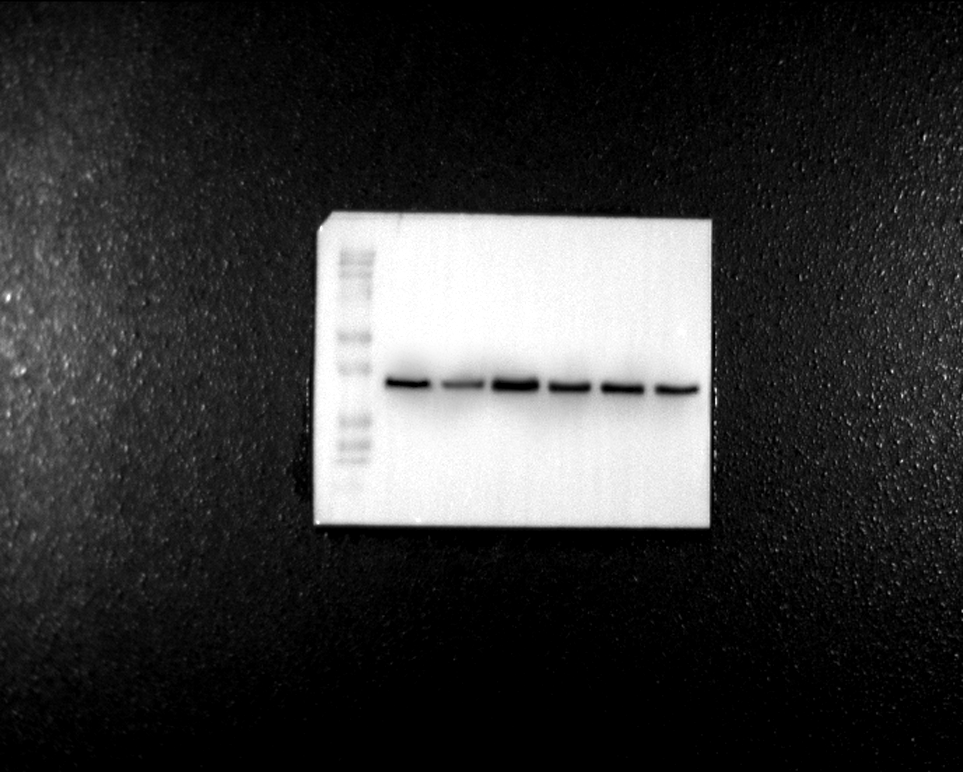

Supplement: Supplementary file 19 [file DataSheet15.ZIP › Raw image of Western blot in Striatum/Ga┴olf (2).tif]

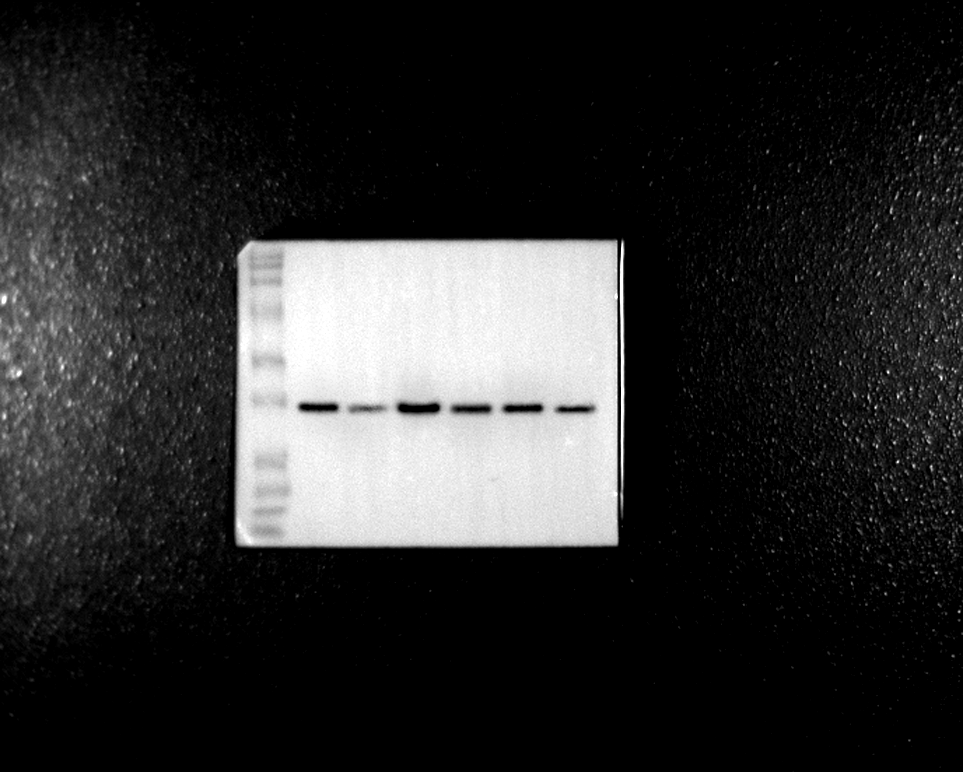

Supplement: Supplementary file 19 [file DataSheet15.ZIP › Raw image of Western blot in Striatum/Ga┴olf (3).tif]

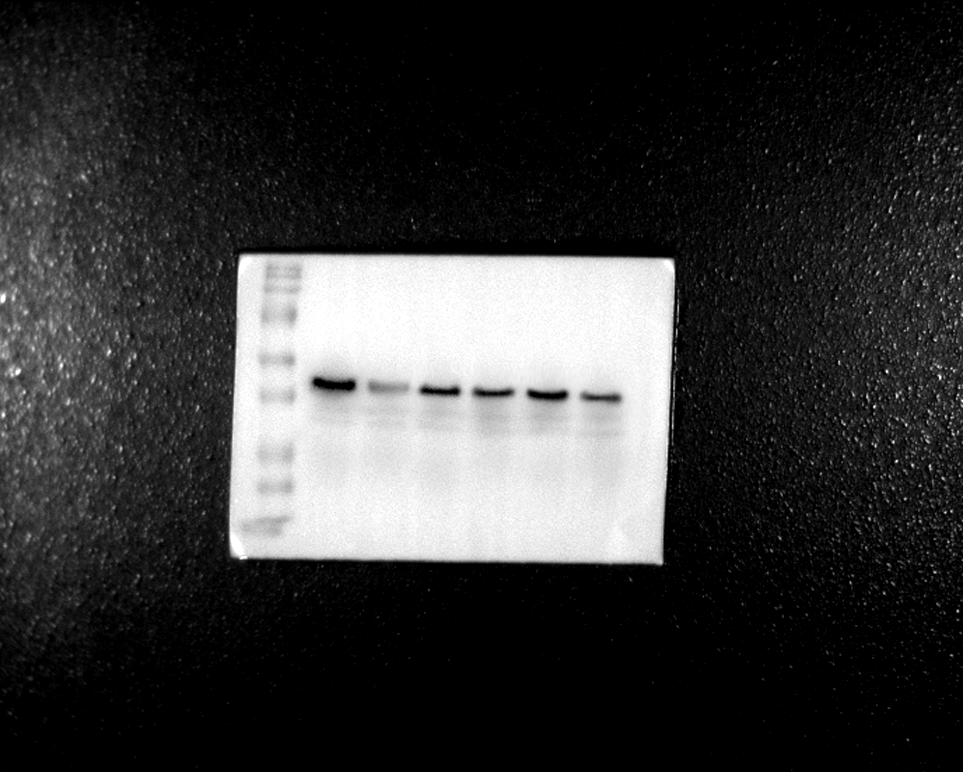

Supplement: Supplementary file 19 [file DataSheet15.ZIP › Raw image of Western blot in Striatum/Ga┴s (1).tif]

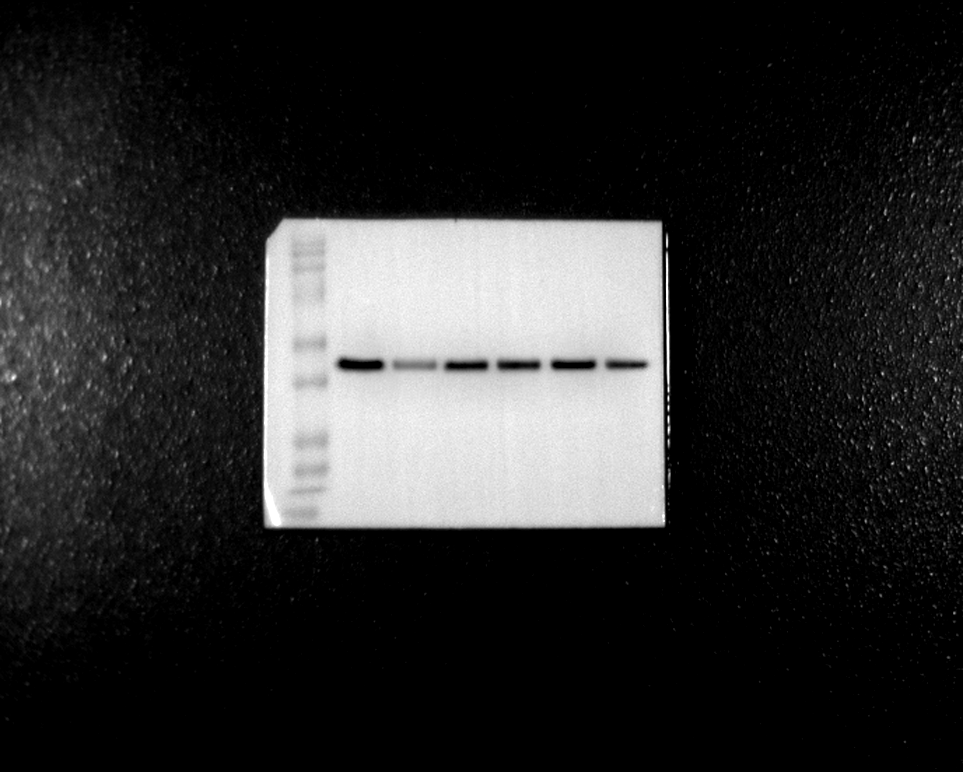

Supplement: Supplementary file 19 [file DataSheet15.ZIP › Raw image of Western blot in Striatum/Ga┴s (2).tif]

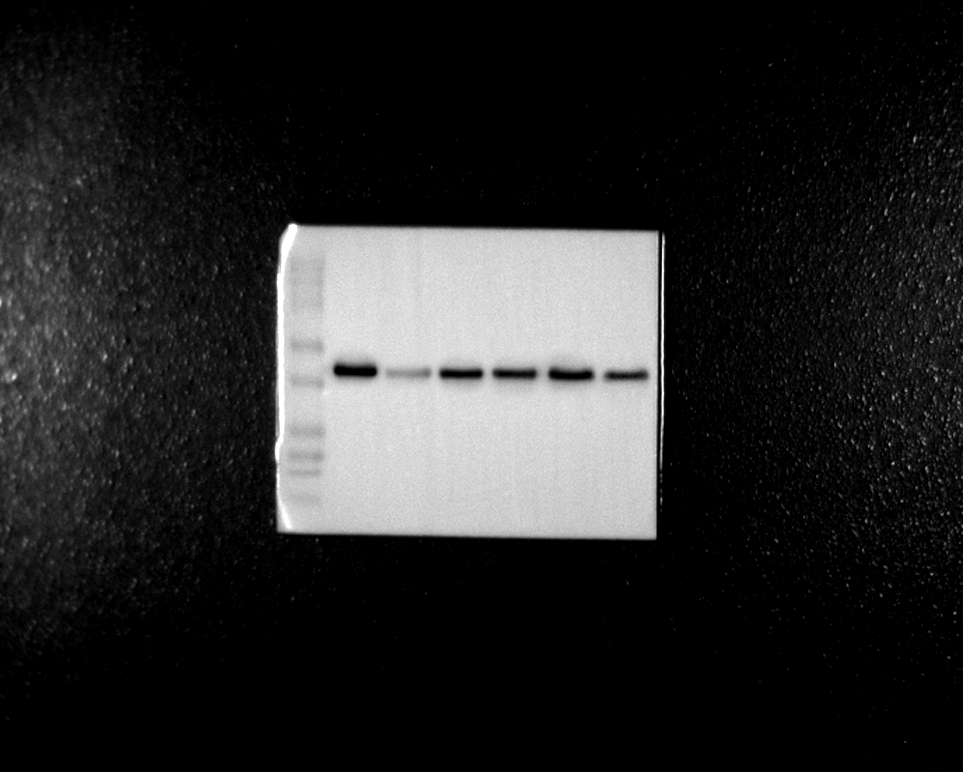

Supplement: Supplementary file 19 [file DataSheet15.ZIP › Raw image of Western blot in Striatum/Ga┴s (3).tif]

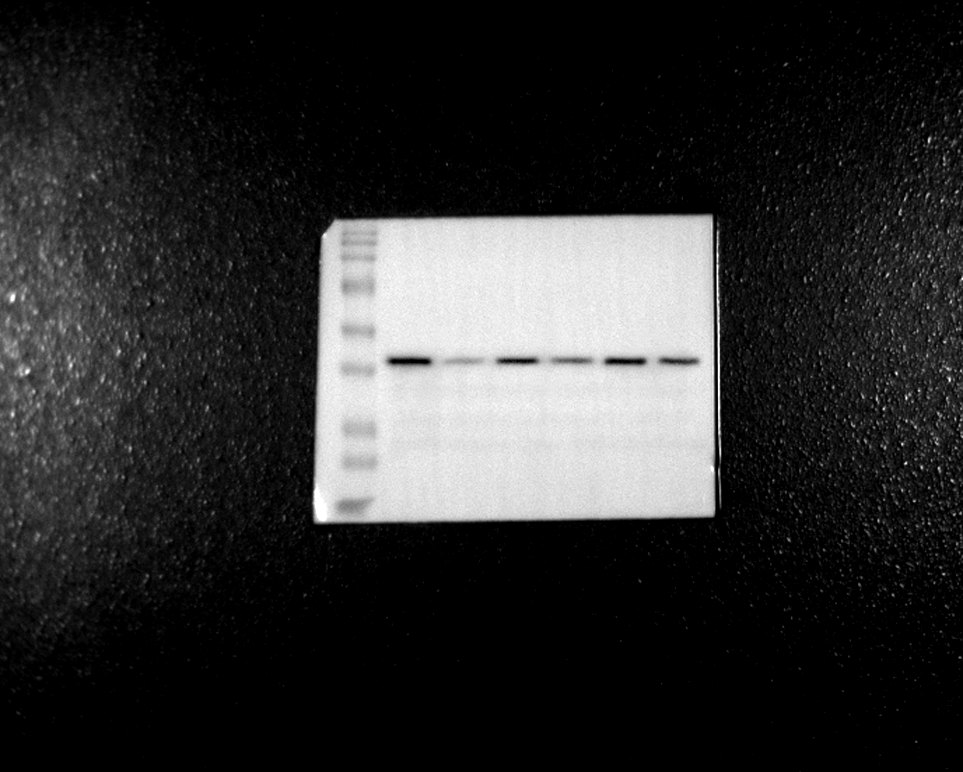

Supplement: Supplementary file 19 [file DataSheet15.ZIP › Raw image of Western blot in Striatum/p-CREB ú¿1ú⌐.tif]

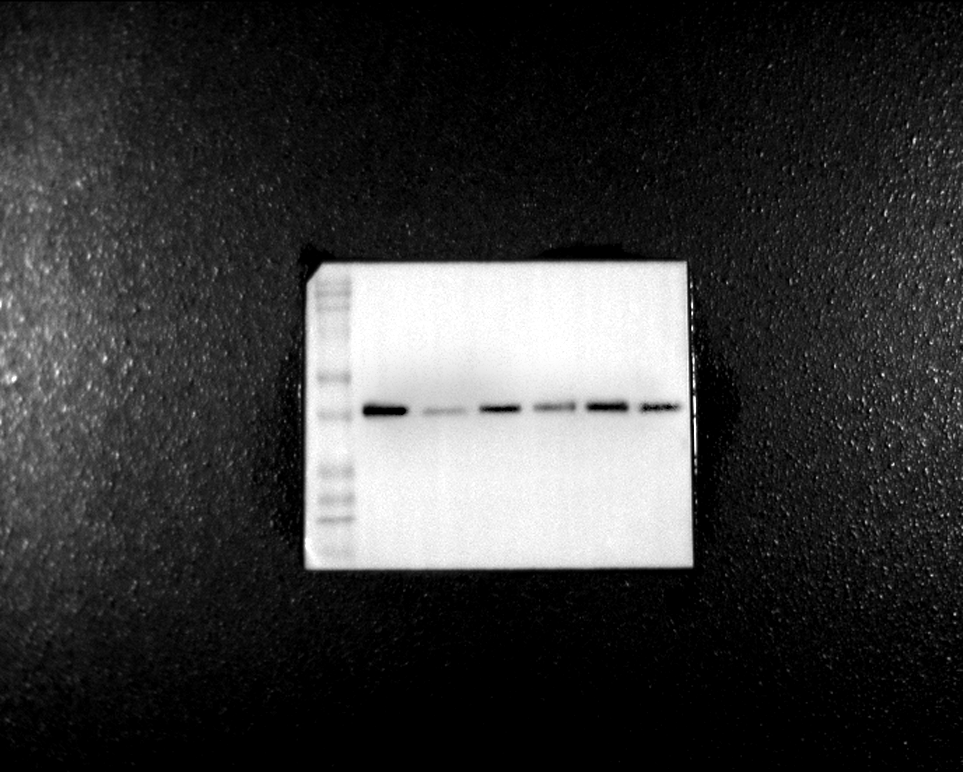

Supplement: Supplementary file 19 [file DataSheet15.ZIP › Raw image of Western blot in Striatum/P-CREB (2).tif]

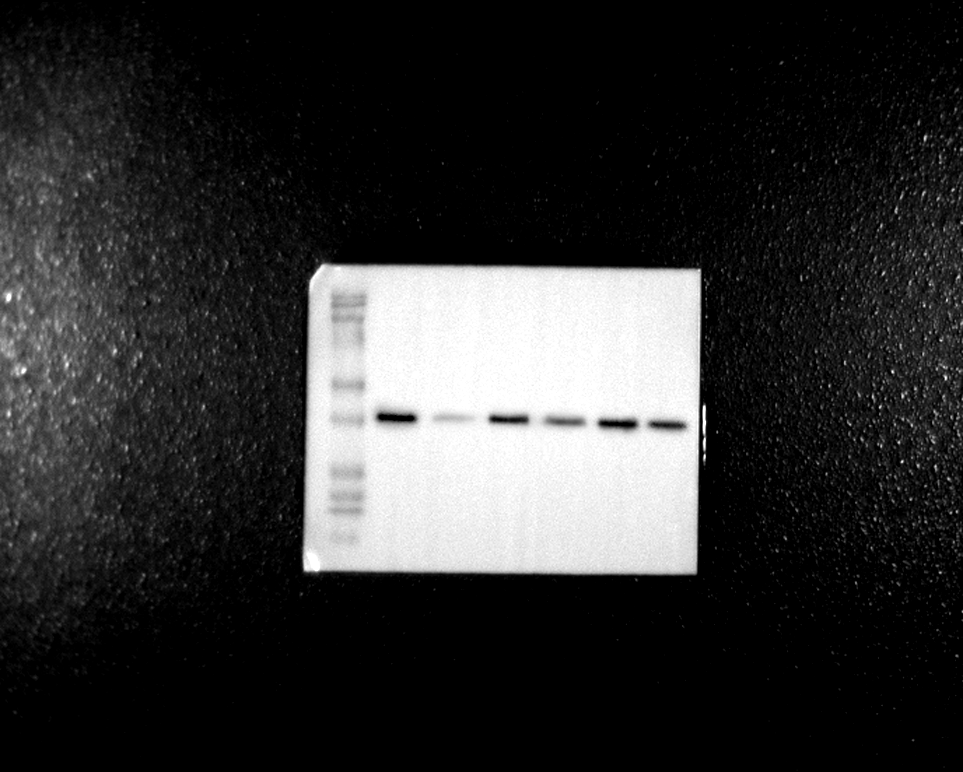

Supplement: Supplementary file 19 [file DataSheet15.ZIP › Raw image of Western blot in Striatum/P-CREB (3).tif]

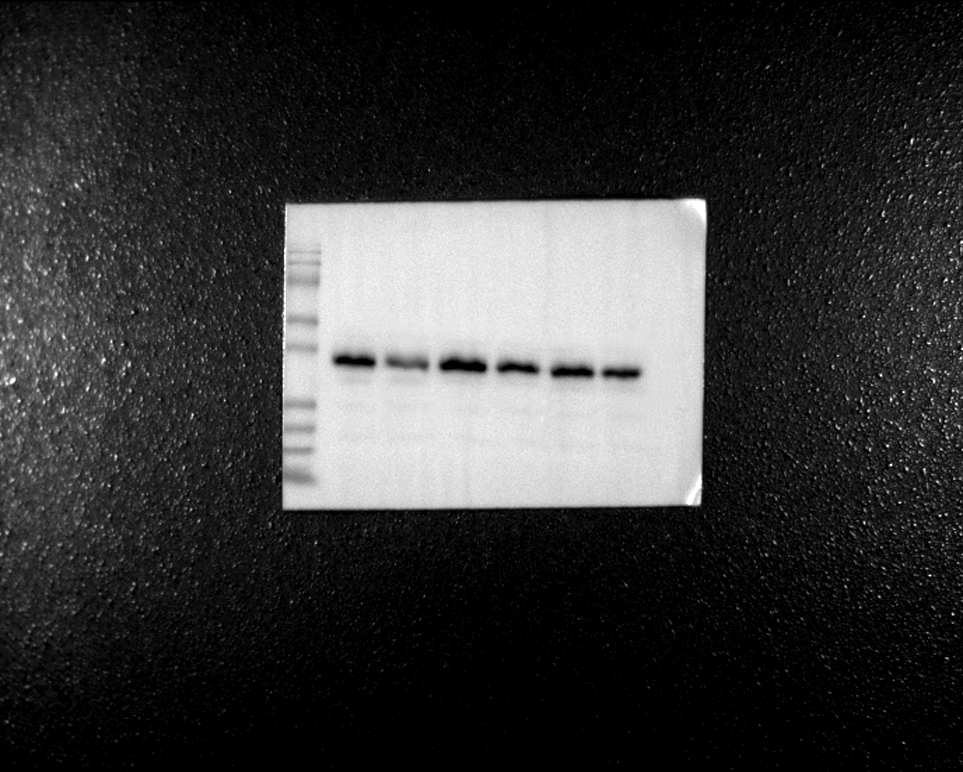

Supplement: Supplementary file 19 [file DataSheet15.ZIP › Raw image of Western blot in Striatum/PKA (1).tif]

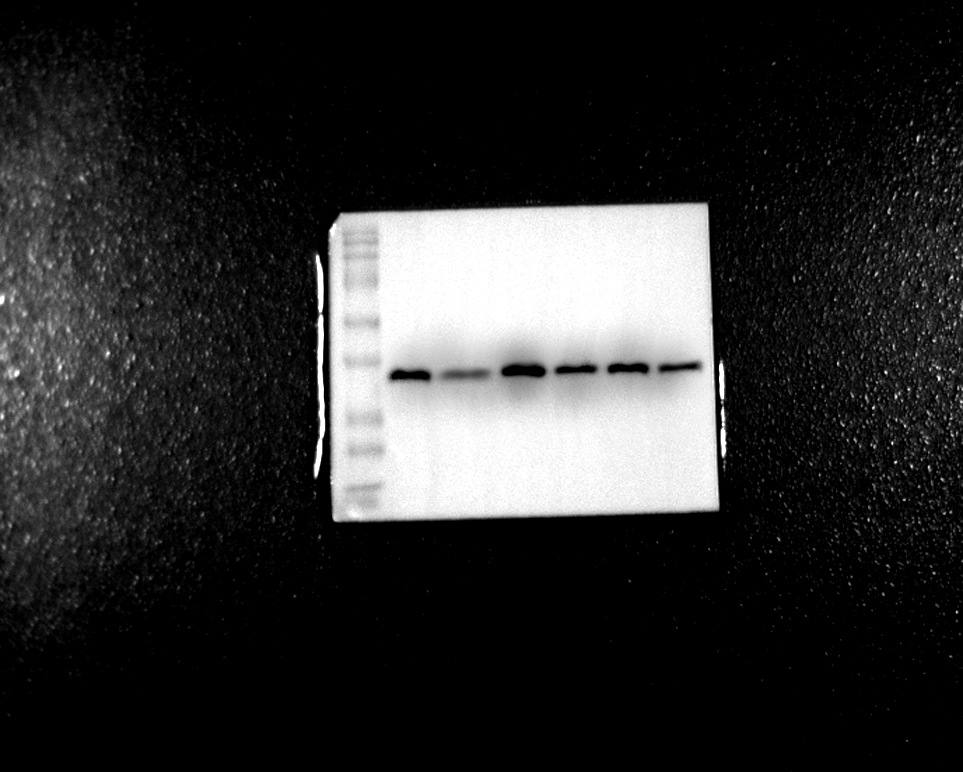

Supplement: Supplementary file 19 [file DataSheet15.ZIP › Raw image of Western blot in Striatum/PKA (2).tif]

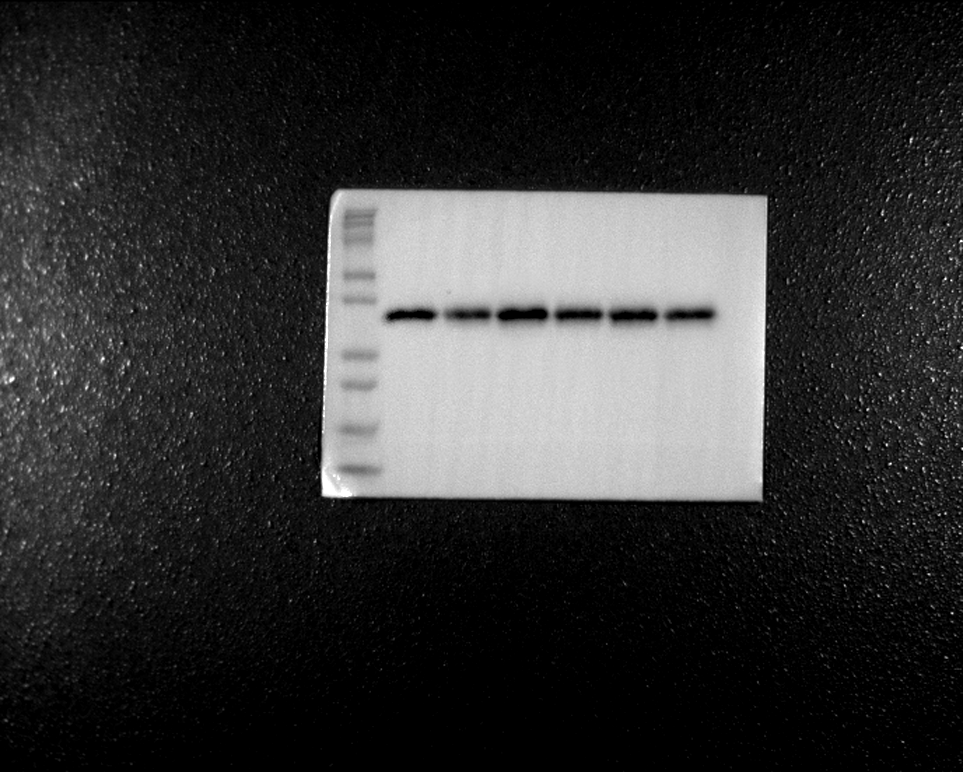

Supplement: Supplementary file 19 [file DataSheet15.ZIP › Raw image of Western blot in Striatum/PKA (3).tif]

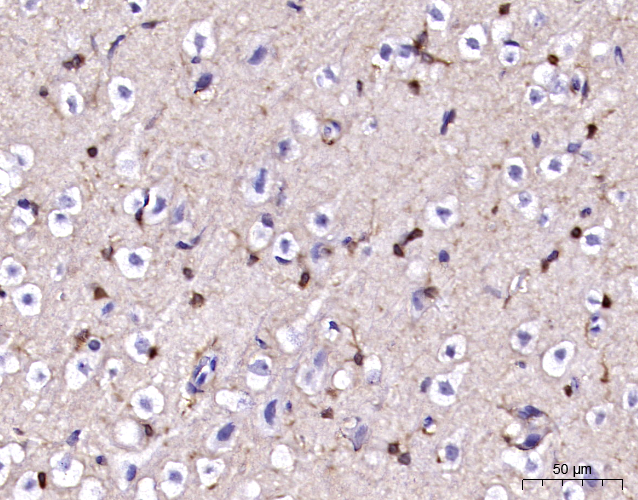

Supplement: Supplementary file 21 [file DataSheet5.ZIP › IHC Raw Image of BDNF in striatum (3)/H34 1-100 BDNF_20.0x.tif-1.tif]

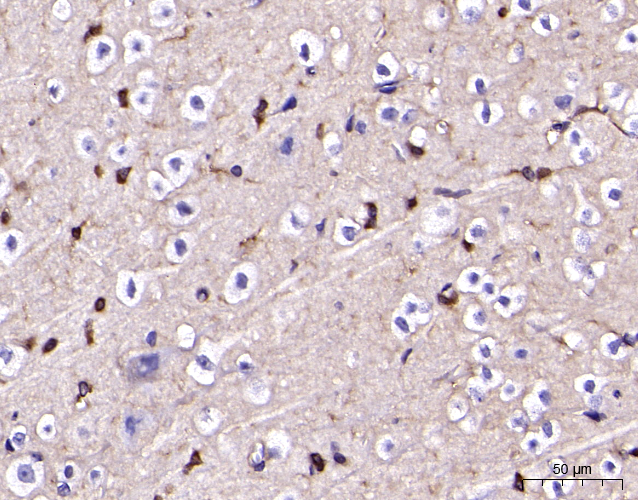

Supplement: Supplementary file 21 [file DataSheet5.ZIP › IHC Raw Image of BDNF in striatum (3)/H34 1-100 BDNF_20.0x.tif-W2.tif]

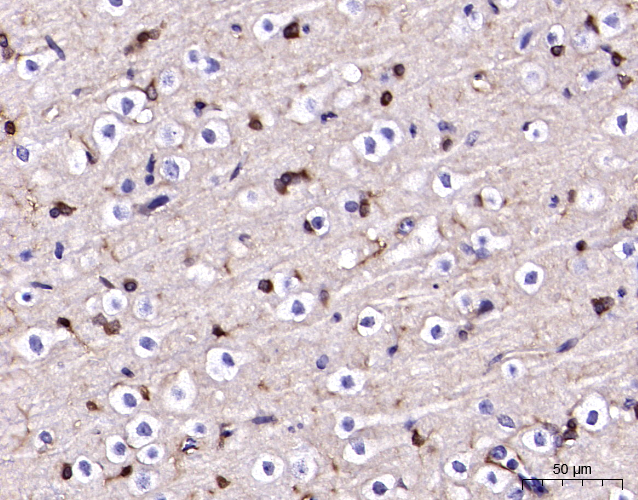

Supplement: Supplementary file 21 [file DataSheet5.ZIP › IHC Raw Image of BDNF in striatum (3)/H34 1-100 BDNF_20.0x.tif-W3.tif]

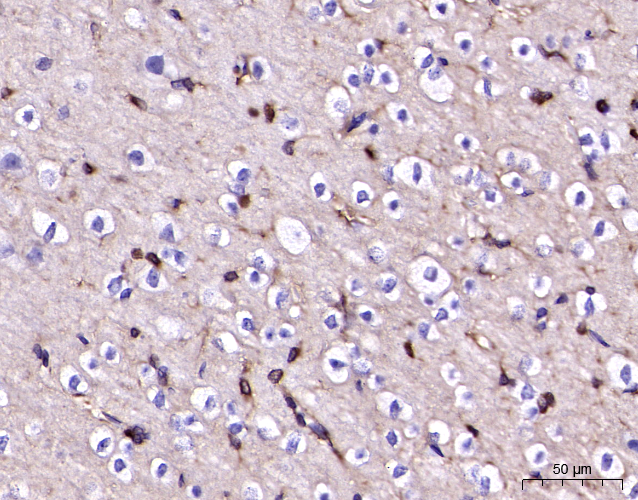

Supplement: Supplementary file 21 [file DataSheet5.ZIP › IHC Raw Image of BDNF in striatum (3)/H34 1-100 BDNF_20.0x.tif-W4.tif]

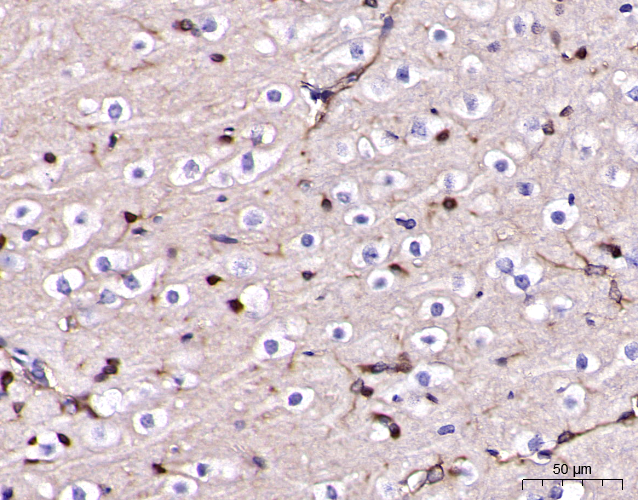

Supplement: Supplementary file 21 [file DataSheet5.ZIP › IHC Raw Image of BDNF in striatum (3)/H34 1-100 BDNF_20.0x.tif-W5.tif]

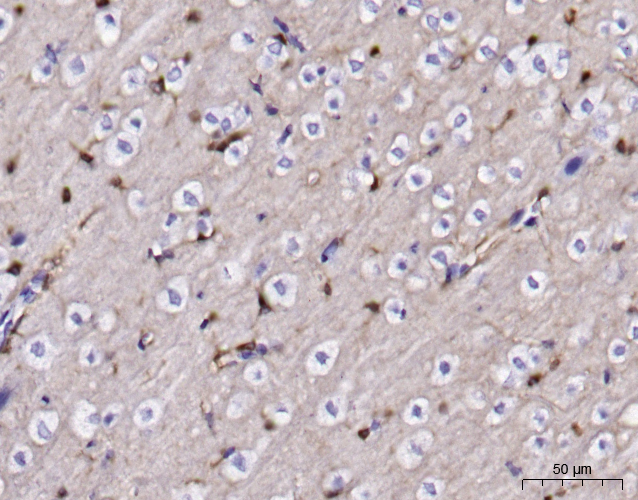

Supplement: Supplementary file 21 [file DataSheet5.ZIP › IHC Raw Image of BDNF in striatum (3)/H41 1-100 BDNF_20.0x.tif-W1.tif]

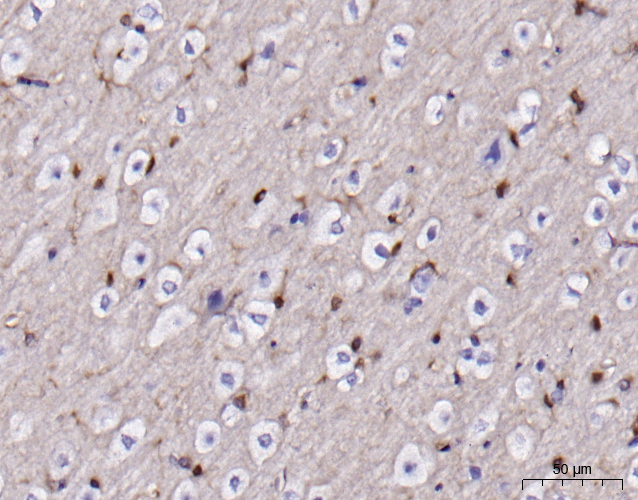

Supplement: Supplementary file 21 [file DataSheet5.ZIP › IHC Raw Image of BDNF in striatum (3)/H41 1-100 BDNF_20.0x.tif-W2.tif]

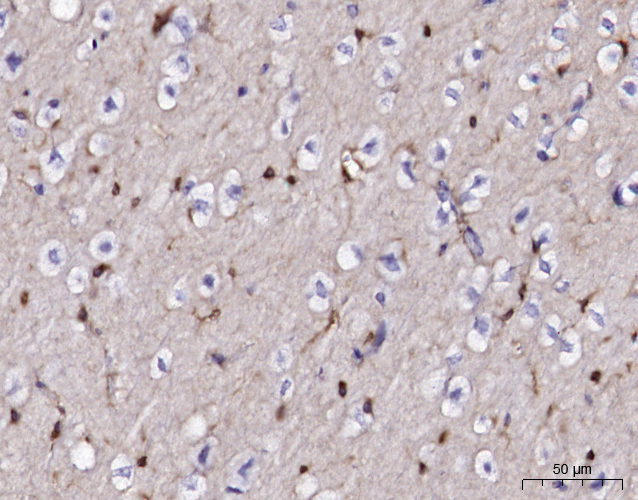

Supplement: Supplementary file 21 [file DataSheet5.ZIP › IHC Raw Image of BDNF in striatum (3)/H41 1-100 BDNF_20.0x.tif-W3.tif]

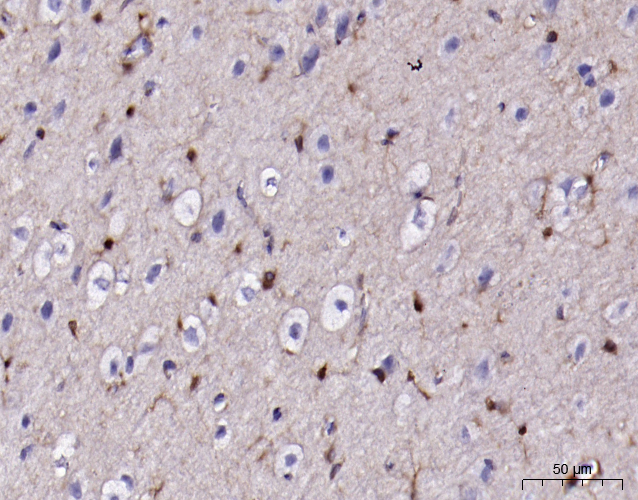

Supplement: Supplementary file 21 [file DataSheet5.ZIP › IHC Raw Image of BDNF in striatum (3)/H41 1-100 BDNF_20.0x.tif-W4.tif]

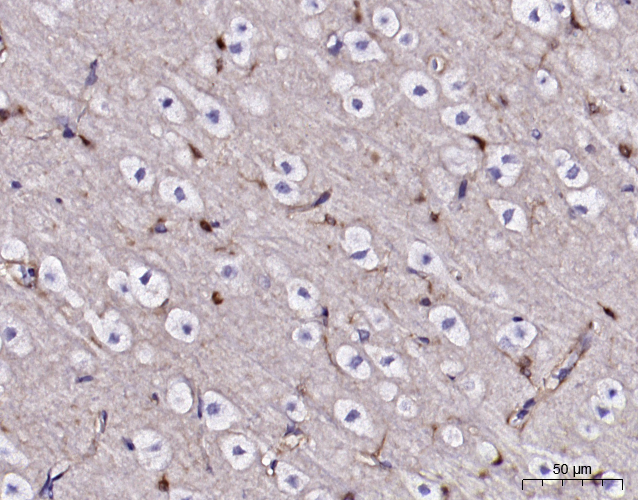

Supplement: Supplementary file 21 [file DataSheet5.ZIP › IHC Raw Image of BDNF in striatum (3)/H41 1-100 BDNF_20.0x.tif-W5.tif]

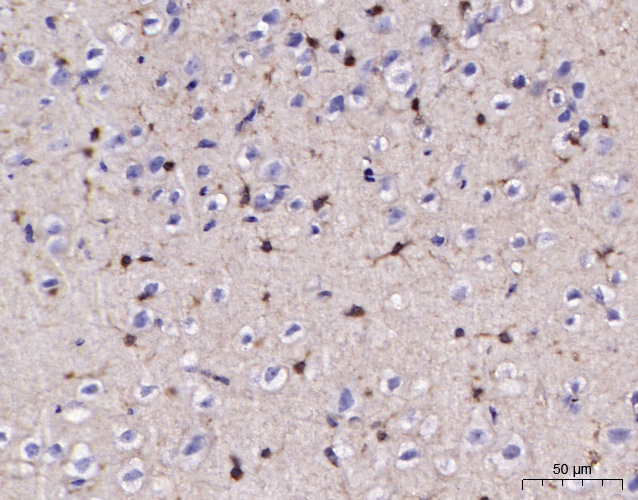

Supplement: Supplementary file 21 [file DataSheet5.ZIP › IHC Raw Image of BDNF in striatum (3)/H48 1-100 BDNF_20.0x.tif-W1.tif]

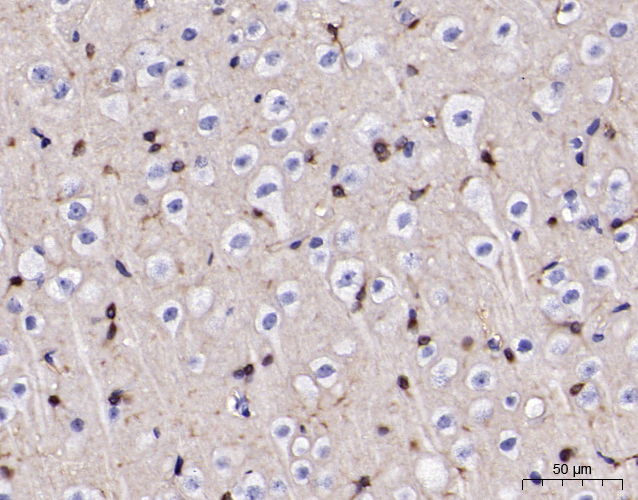

Supplement: Supplementary file 21 [file DataSheet5.ZIP › IHC Raw Image of BDNF in striatum (3)/H48 1-100 BDNF_20.0x.tif-W2.tif]

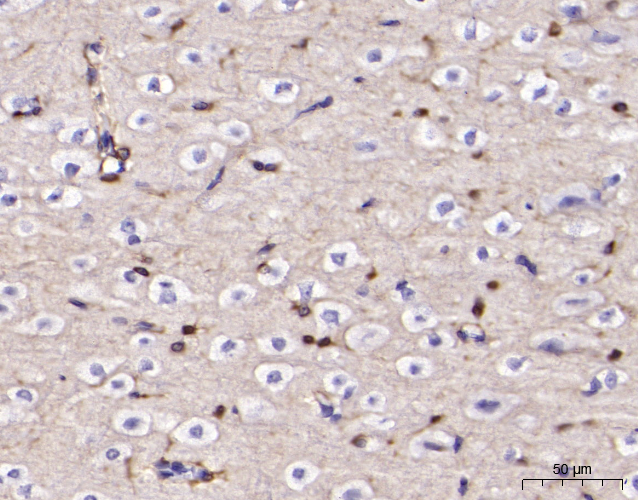

Supplement: Supplementary file 21 [file DataSheet5.ZIP › IHC Raw Image of BDNF in striatum (3)/H48 1-100 BDNF_20.0x.tif-W3.tif]

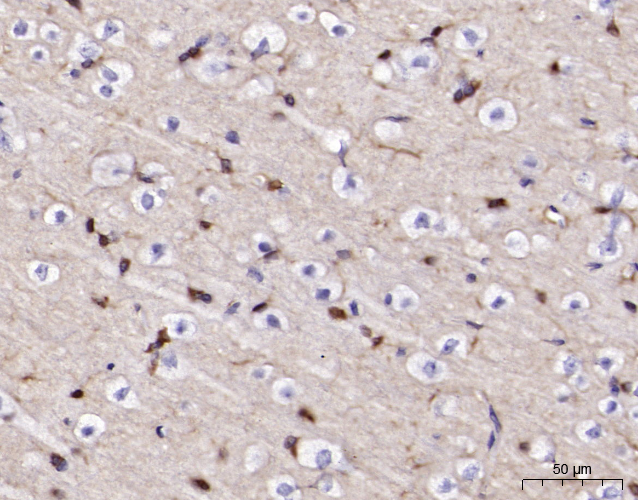

Supplement: Supplementary file 21 [file DataSheet5.ZIP › IHC Raw Image of BDNF in striatum (3)/H48 1-100 BDNF_20.0x.tif-W4.tif]

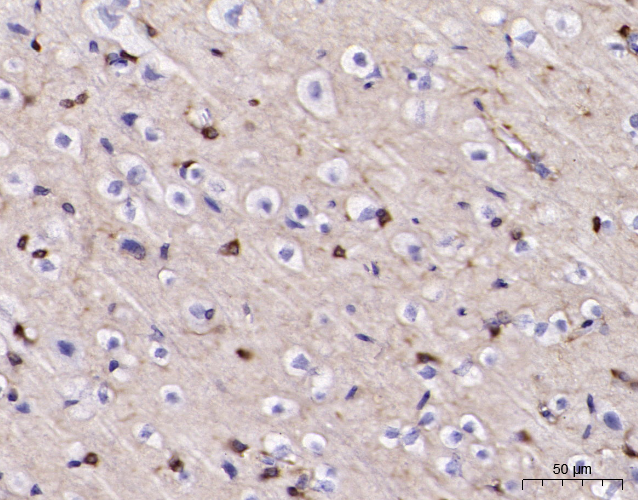

Supplement: Supplementary file 21 [file DataSheet5.ZIP › IHC Raw Image of BDNF in striatum (3)/H48 1-100 BDNF_20.0x.tif-W5.tif]

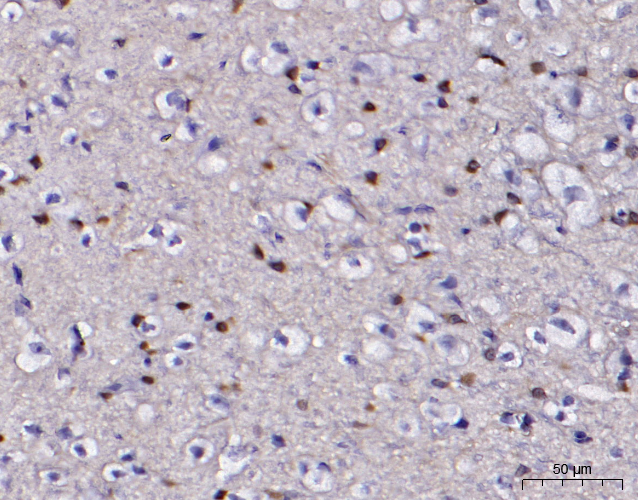

Supplement: Supplementary file 21 [file DataSheet5.ZIP › IHC Raw Image of BDNF in striatum (3)/H53 1-100 BDNF_20.0x.tif-W1.tif]

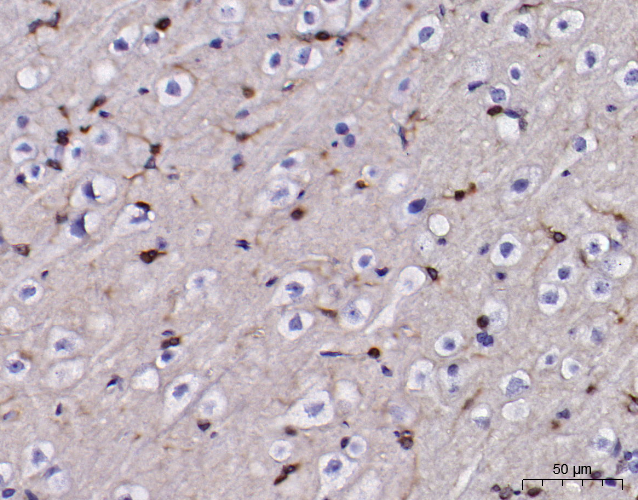

Supplement: Supplementary file 21 [file DataSheet5.ZIP › IHC Raw Image of BDNF in striatum (3)/H53 1-100 BDNF_20.0x.tif-W2.tif]

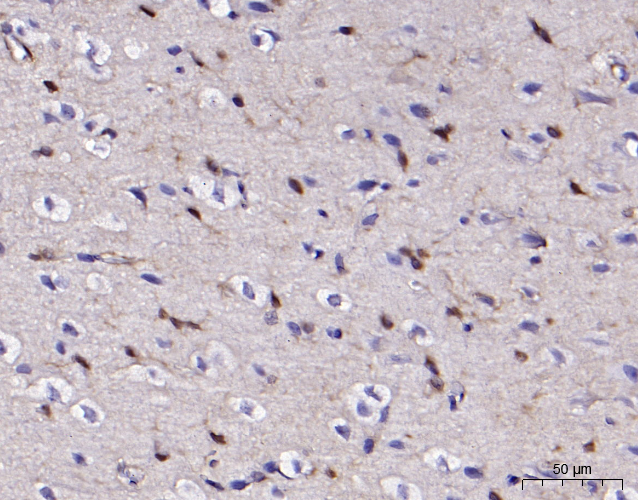

Supplement: Supplementary file 21 [file DataSheet5.ZIP › IHC Raw Image of BDNF in striatum (3)/H53 1-100 BDNF_20.0x.tif-W3.tif]

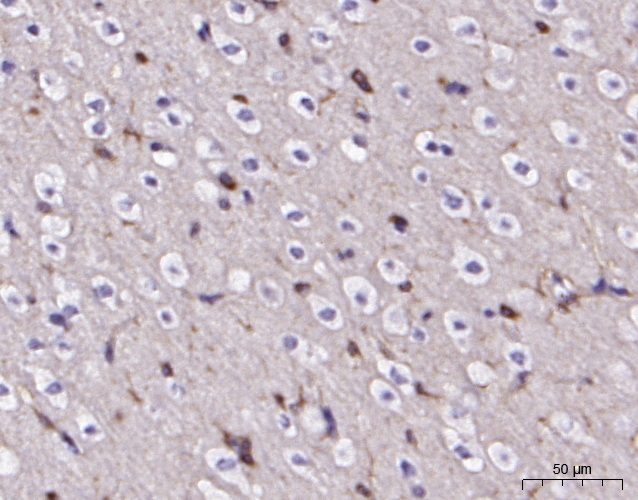

Supplement: Supplementary file 21 [file DataSheet5.ZIP › IHC Raw Image of BDNF in striatum (3)/H53 1-100 BDNF_20.0x.tif-W4.tif]

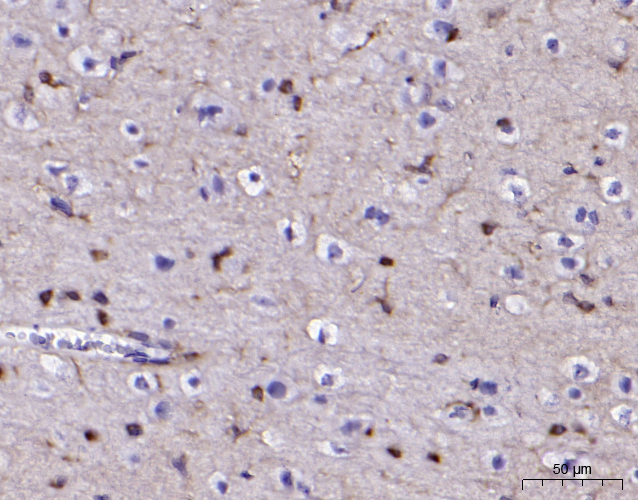

Supplement: Supplementary file 21 [file DataSheet5.ZIP › IHC Raw Image of BDNF in striatum (3)/H53 1-100 BDNF_20.0x.tif-W5.tif]

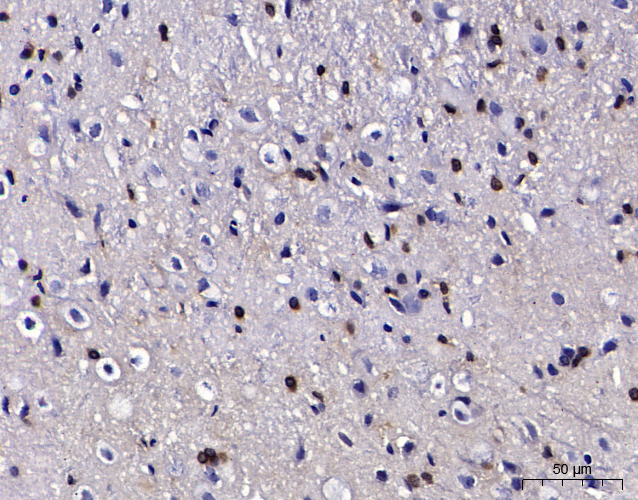

Supplement: Supplementary file 21 [file DataSheet5.ZIP › IHC Raw Image of BDNF in striatum (3)/M11 1-100 BDNF_20.0x.tif-W1.tif]

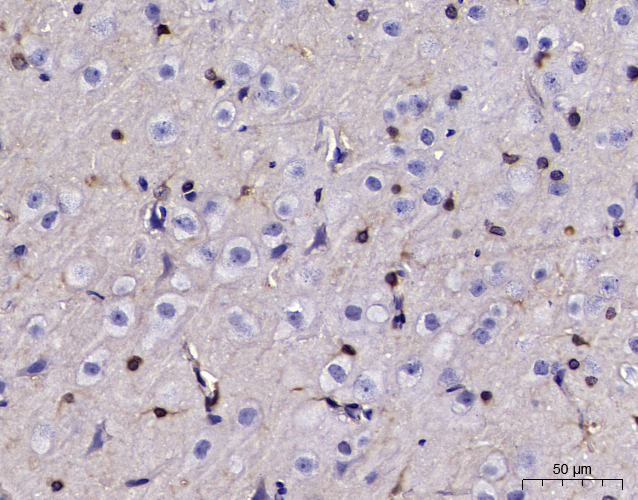

Supplement: Supplementary file 21 [file DataSheet5.ZIP › IHC Raw Image of BDNF in striatum (3)/M11 1-100 BDNF_20.0x.tif-W2.tif]

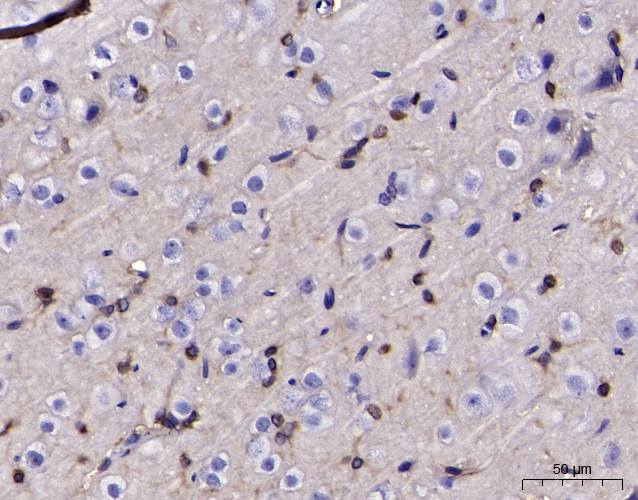

Supplement: Supplementary file 21 [file DataSheet5.ZIP › IHC Raw Image of BDNF in striatum (3)/M11 1-100 BDNF_20.0x.tif-W3.tif]

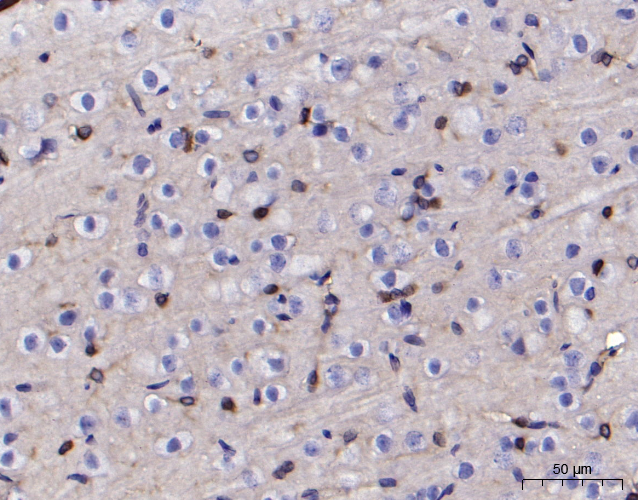

Supplement: Supplementary file 21 [file DataSheet5.ZIP › IHC Raw Image of BDNF in striatum (3)/M11 1-100 BDNF_20.0x.tif-W4.tif]

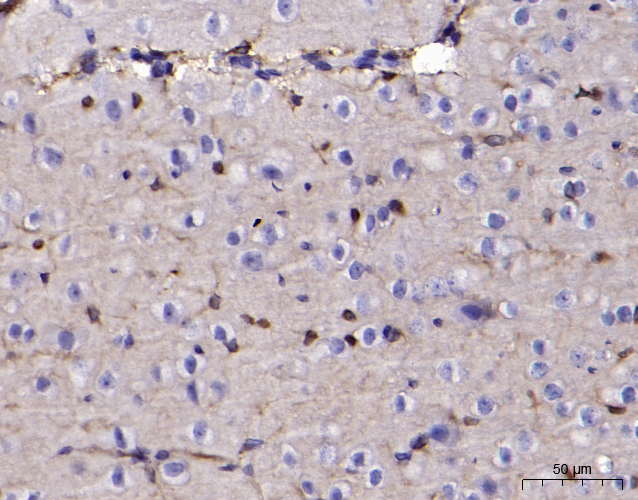

Supplement: Supplementary file 21 [file DataSheet5.ZIP › IHC Raw Image of BDNF in striatum (3)/M11 1-100 BDNF_20.0x.tif-W5.tif]

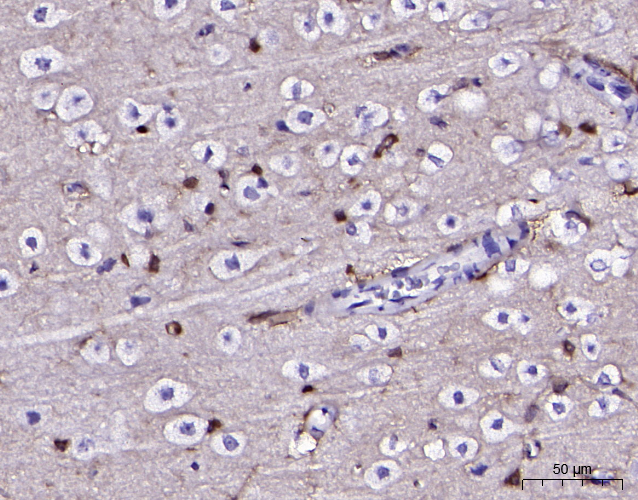

Supplement: Supplementary file 21 [file DataSheet5.ZIP › IHC Raw Image of BDNF in striatum (3)/M27 1-100 BDNF_20.0x.tif-W1.tif]

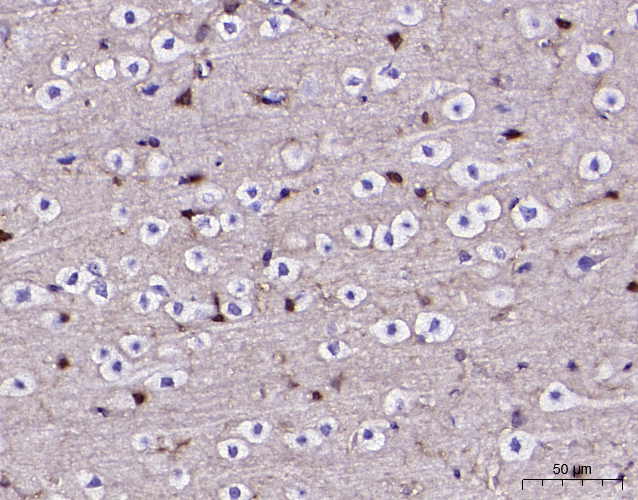

Supplement: Supplementary file 21 [file DataSheet5.ZIP › IHC Raw Image of BDNF in striatum (3)/M27 1-100 BDNF_20.0x.tif-W2.tif]

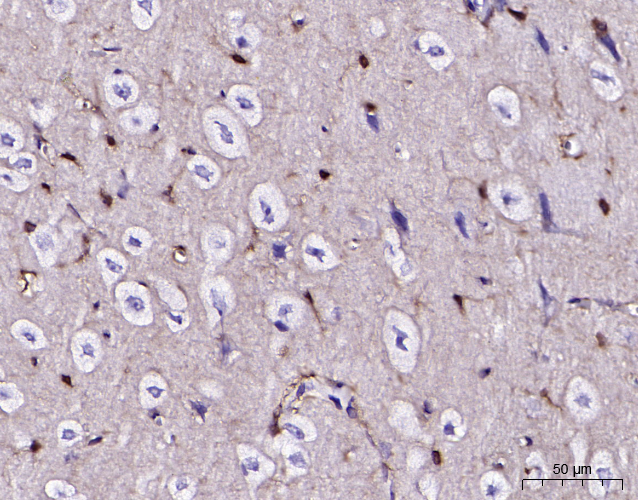

Supplement: Supplementary file 21 [file DataSheet5.ZIP › IHC Raw Image of BDNF in striatum (3)/M27 1-100 BDNF_20.0x.tif-W3.tif]

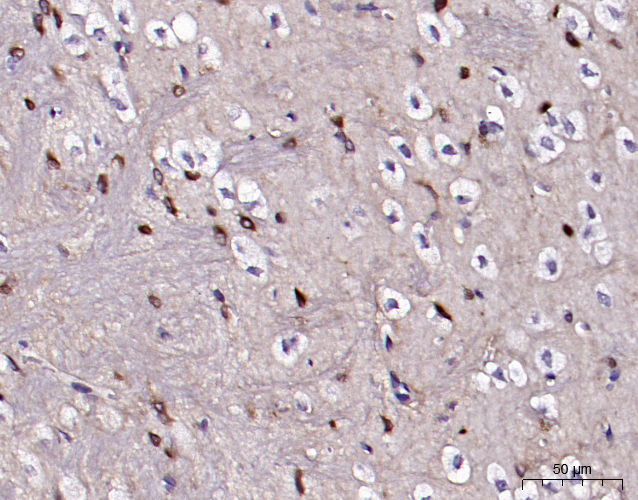

Supplement: Supplementary file 21 [file DataSheet5.ZIP › IHC Raw Image of BDNF in striatum (3)/M27 1-100 BDNF_20.0x.tif-W4.tif]

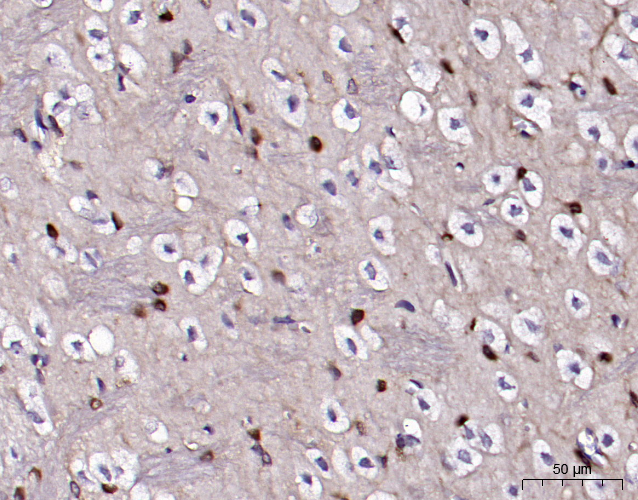

Supplement: Supplementary file 21 [file DataSheet5.ZIP › IHC Raw Image of BDNF in striatum (3)/M27 1-100 BDNF_20.0x.tif-W5.tif]

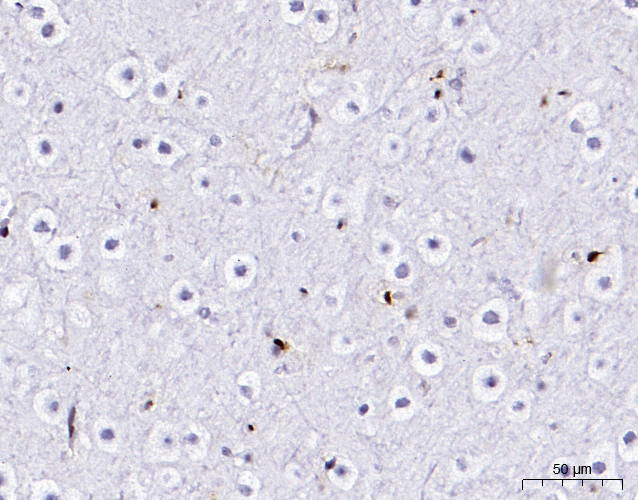

Supplement: Supplementary file 24 [file DataSheet7.ZIP › IHC Raw Image of P-CREB in PFC(2)/MX1 1-200 PCREB_20.0x.tif-Q1.tif]

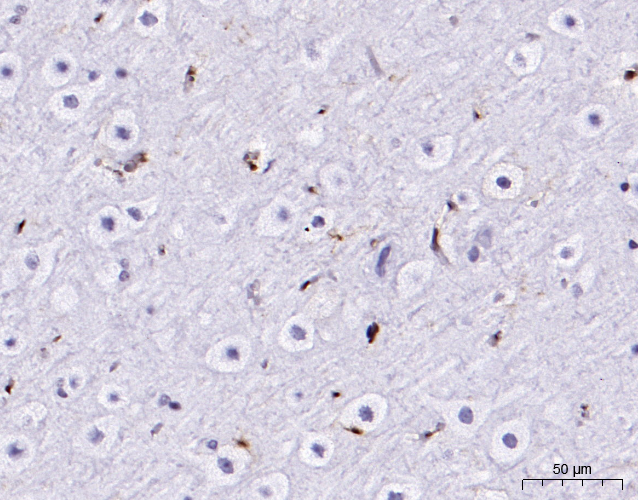

Supplement: Supplementary file 24 [file DataSheet7.ZIP › IHC Raw Image of P-CREB in PFC(2)/MX1 1-200 PCREB_20.0x.tif-Q2.tif]

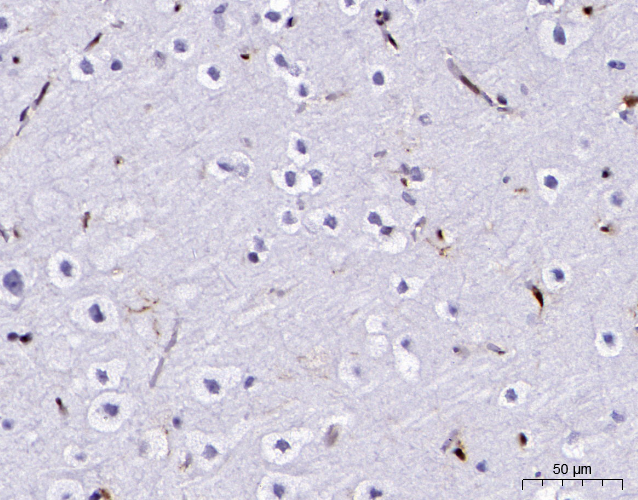

Supplement: Supplementary file 24 [file DataSheet7.ZIP › IHC Raw Image of P-CREB in PFC(2)/MX1 1-200 PCREB_20.0x.tif-Q3.tif]

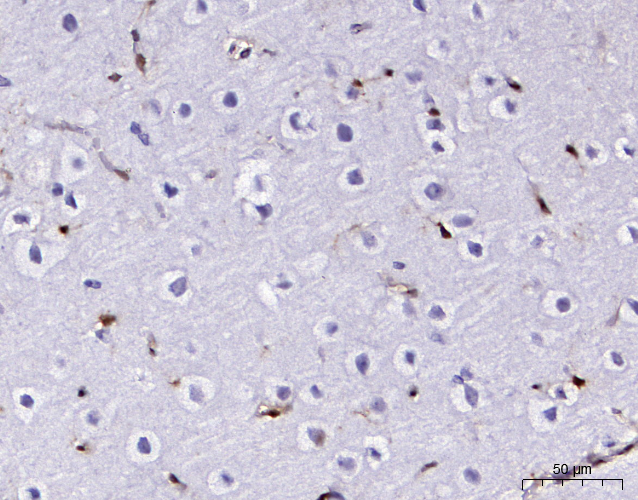

Supplement: Supplementary file 24 [file DataSheet7.ZIP › IHC Raw Image of P-CREB in PFC(2)/MX1 1-200 PCREB_20.0x.tif-Q4.tif]

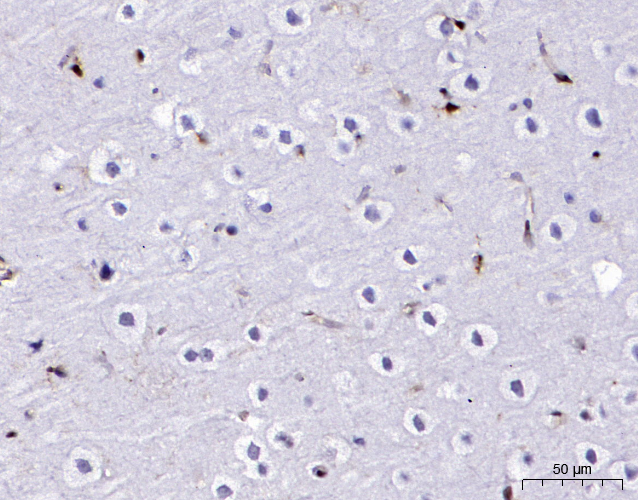

Supplement: Supplementary file 24 [file DataSheet7.ZIP › IHC Raw Image of P-CREB in PFC(2)/MX1 1-200 PCREB_20.0x.tif-Q5.tif]

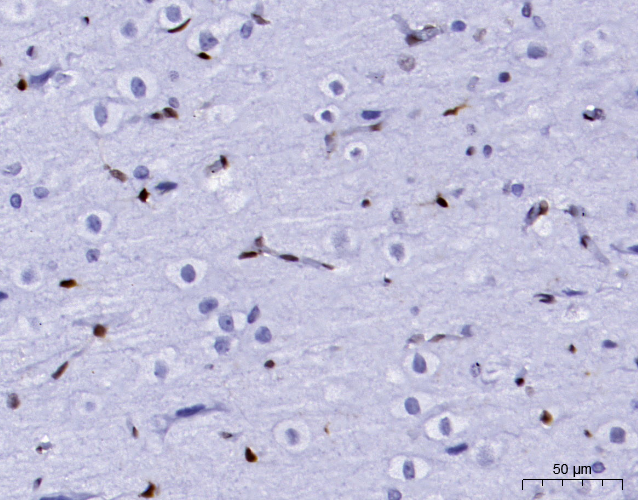

Supplement: Supplementary file 24 [file DataSheet7.ZIP › IHC Raw Image of P-CREB in PFC(2)/MX18 1-200 PCREB_20.0x.tif-Q1.tif]

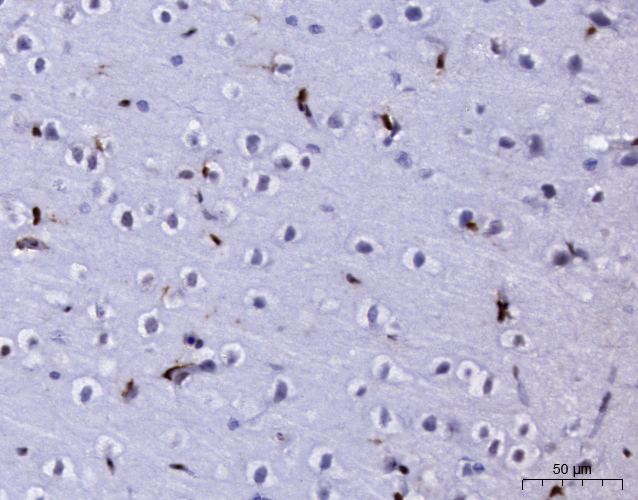

Supplement: Supplementary file 24 [file DataSheet7.ZIP › IHC Raw Image of P-CREB in PFC(2)/MX18 1-200 PCREB_20.0x.tif-Q2.tif]

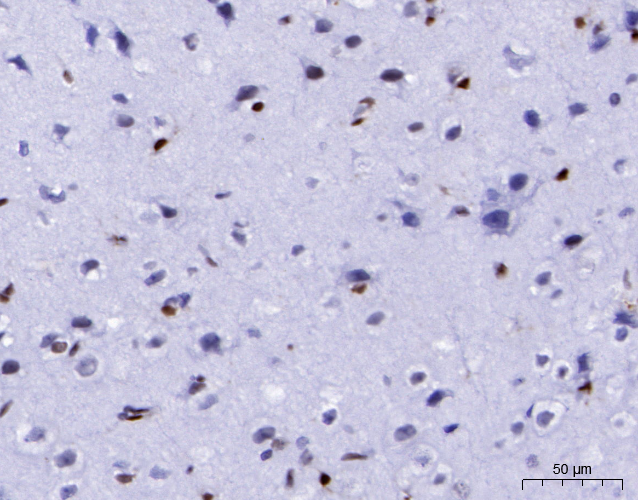

Supplement: Supplementary file 24 [file DataSheet7.ZIP › IHC Raw Image of P-CREB in PFC(2)/MX18 1-200 PCREB_20.0x.tif-Q3.tif]

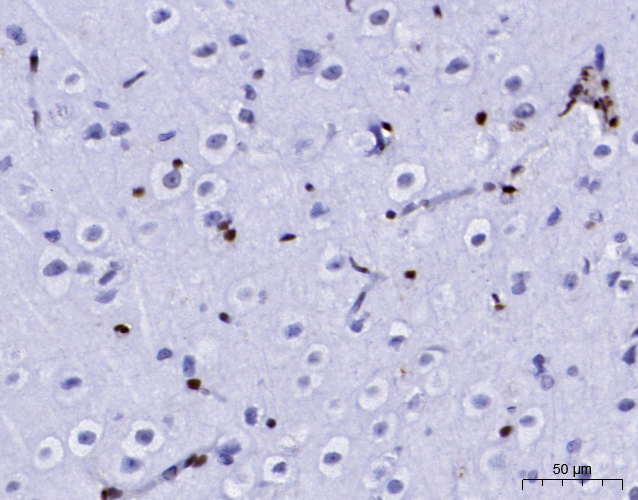

Supplement: Supplementary file 24 [file DataSheet7.ZIP › IHC Raw Image of P-CREB in PFC(2)/MX18 1-200 PCREB_20.0x.tif-Q4.tif]

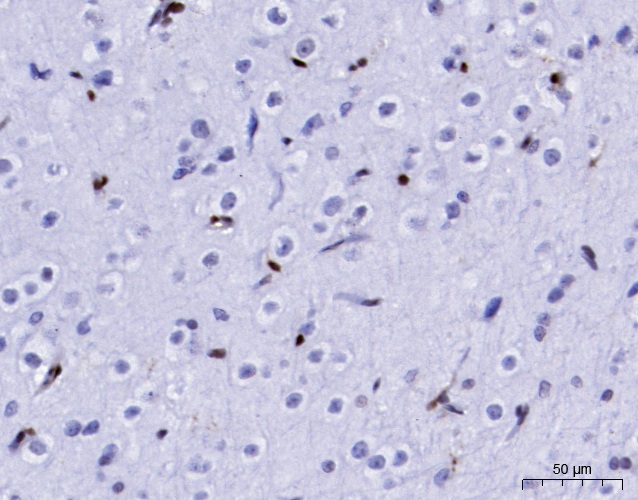

Supplement: Supplementary file 24 [file DataSheet7.ZIP › IHC Raw Image of P-CREB in PFC(2)/MX18 1-200 PCREB_20.0x.tif-Q5.tif]

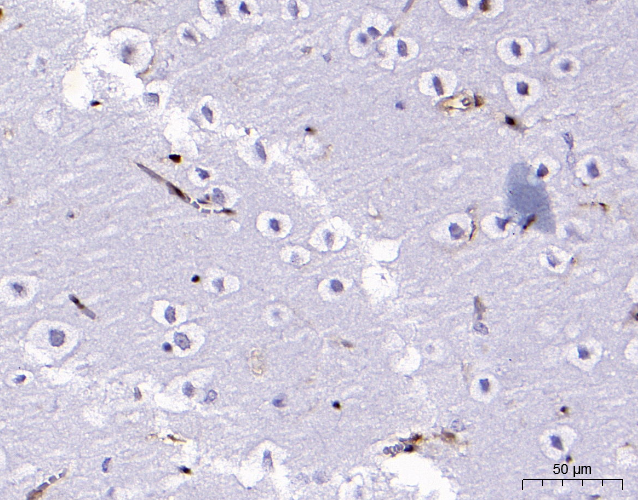

Supplement: Supplementary file 24 [file DataSheet7.ZIP › IHC Raw Image of P-CREB in PFC(2)/MX5 1-200 PCREB_20.0x.tif-Q1.tif]

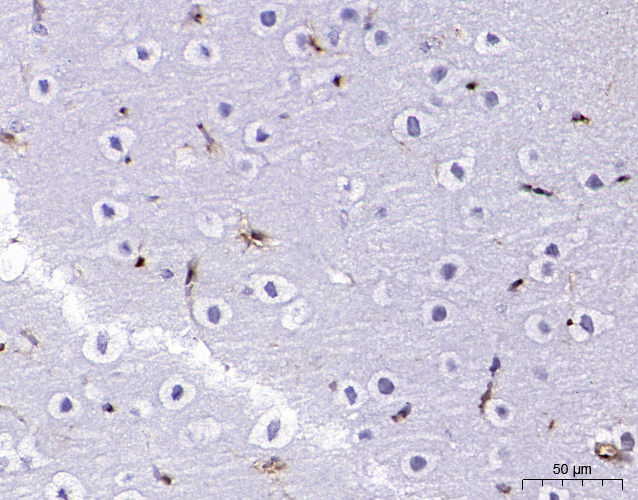

Supplement: Supplementary file 24 [file DataSheet7.ZIP › IHC Raw Image of P-CREB in PFC(2)/MX5 1-200 PCREB_20.0x.tif-Q2.tif]

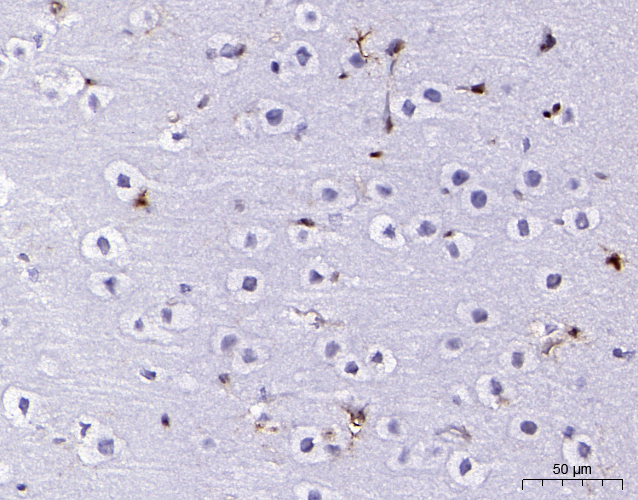

Supplement: Supplementary file 24 [file DataSheet7.ZIP › IHC Raw Image of P-CREB in PFC(2)/MX5 1-200 PCREB_20.0x.tif-Q3.tif]

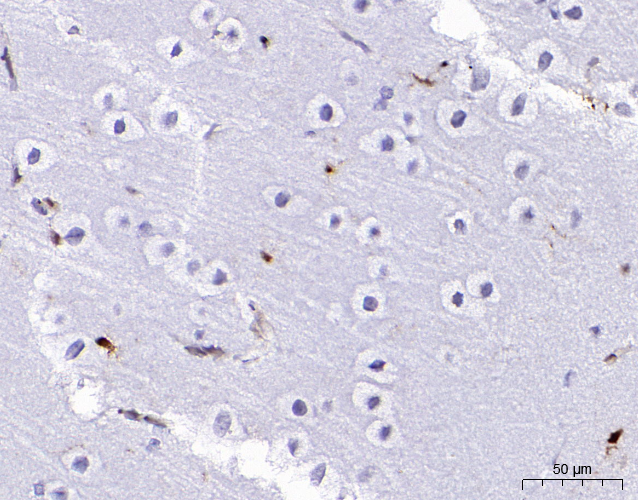

Supplement: Supplementary file 24 [file DataSheet7.ZIP › IHC Raw Image of P-CREB in PFC(2)/MX5 1-200 PCREB_20.0x.tif-Q4.tif]

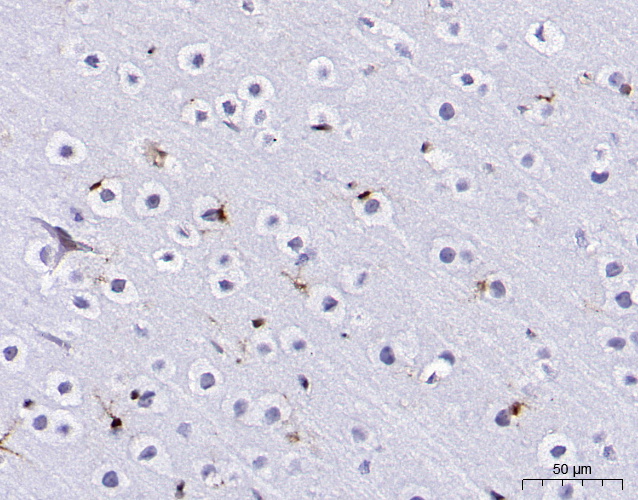

Supplement: Supplementary file 24 [file DataSheet7.ZIP › IHC Raw Image of P-CREB in PFC(2)/MX5 1-200 PCREB_20.0x.tif-Q5.tif]

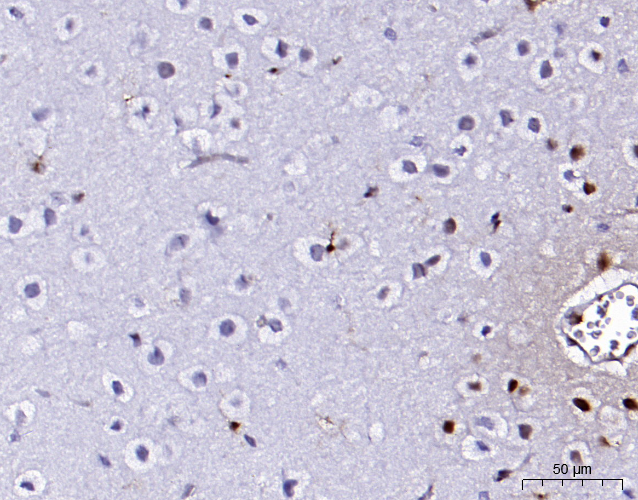

Supplement: Supplementary file 24 [file DataSheet7.ZIP › IHC Raw Image of P-CREB in PFC(2)/MXZ1 1-200 PCREB_20.0x.tif-Q1.tif]

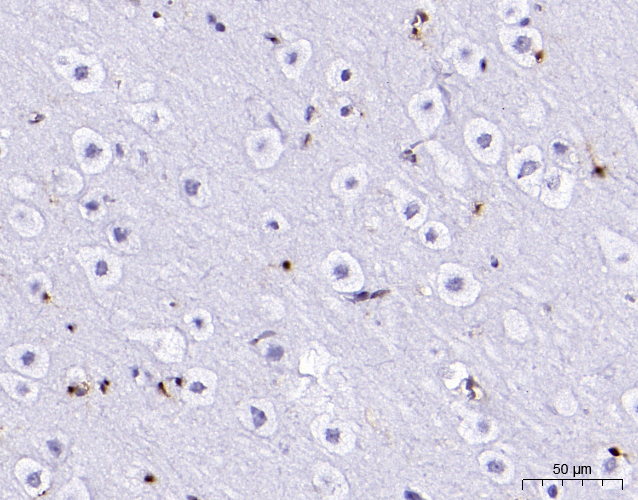

Supplement: Supplementary file 24 [file DataSheet7.ZIP › IHC Raw Image of P-CREB in PFC(2)/MXZ1 1-200 PCREB_20.0x.tif-Q2.tif]

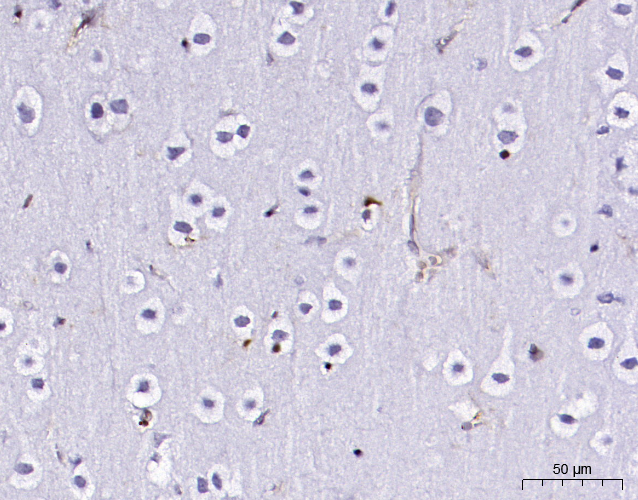

Supplement: Supplementary file 24 [file DataSheet7.ZIP › IHC Raw Image of P-CREB in PFC(2)/MXZ1 1-200 PCREB_20.0x.tif-Q3.tif]

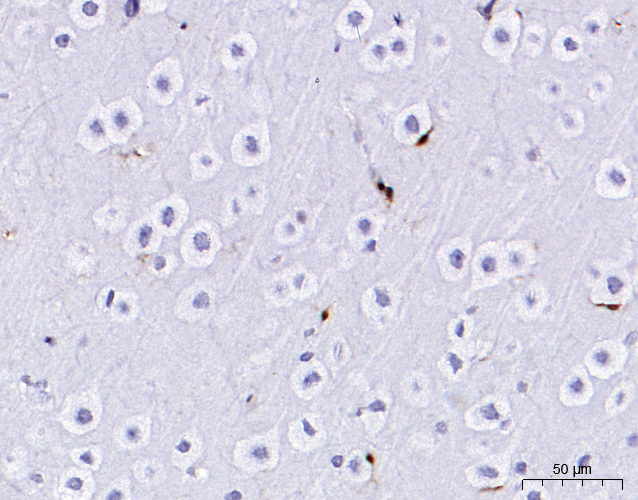

Supplement: Supplementary file 24 [file DataSheet7.ZIP › IHC Raw Image of P-CREB in PFC(2)/MXZ1 1-200 PCREB_20.0x.tif-Q4.tif]

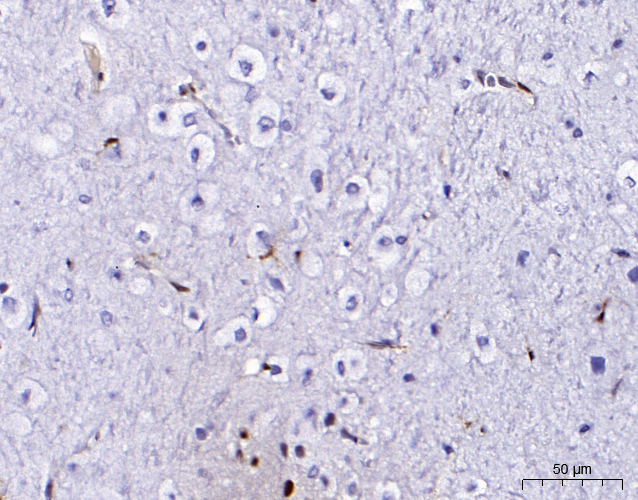

Supplement: Supplementary file 24 [file DataSheet7.ZIP › IHC Raw Image of P-CREB in PFC(2)/MXZ1 1-200 PCREB_20.0x.tif-Q5.tif]

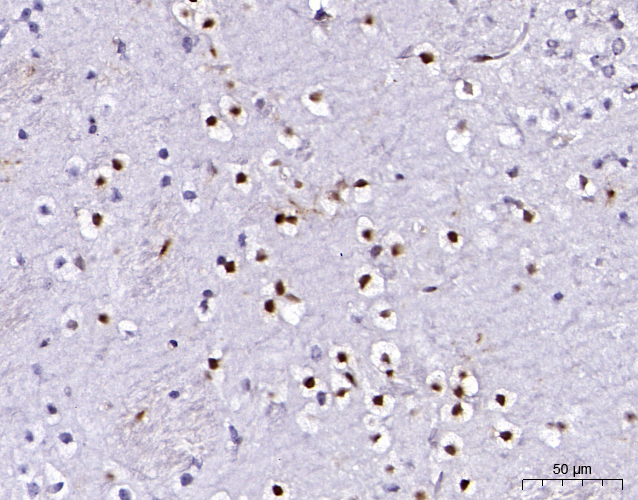

Supplement: Supplementary file 24 [file DataSheet7.ZIP › IHC Raw Image of P-CREB in PFC(2)/Z23 1-200 PCREB_20.0x.tif-Q1.tif]

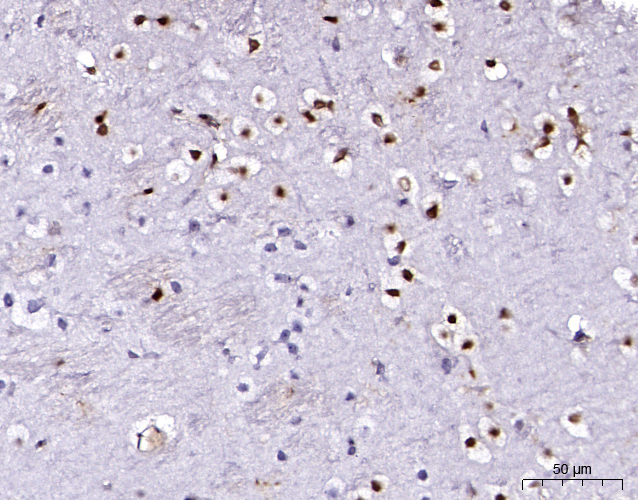

Supplement: Supplementary file 24 [file DataSheet7.ZIP › IHC Raw Image of P-CREB in PFC(2)/Z23 1-200 PCREB_20.0x.tif-Q2.tif]

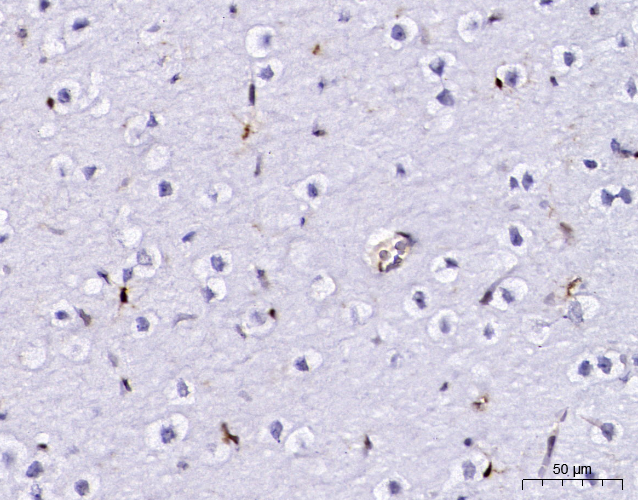

Supplement: Supplementary file 24 [file DataSheet7.ZIP › IHC Raw Image of P-CREB in PFC(2)/Z23 1-200 PCREB_20.0x.tif-Q3.tif]

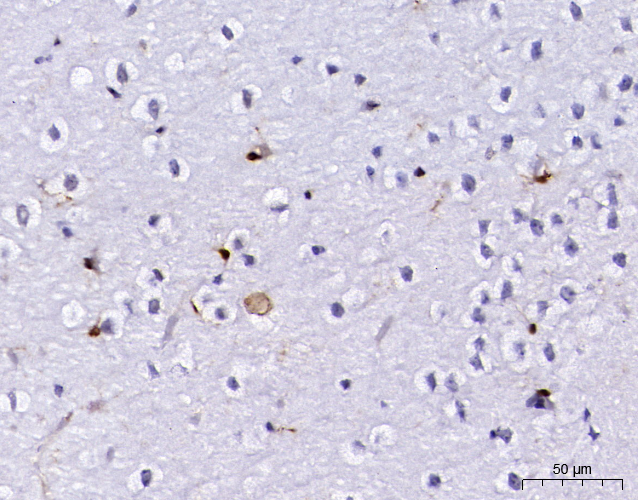

Supplement: Supplementary file 24 [file DataSheet7.ZIP › IHC Raw Image of P-CREB in PFC(2)/Z23 1-200 PCREB_20.0x.tif-Q4.tif]

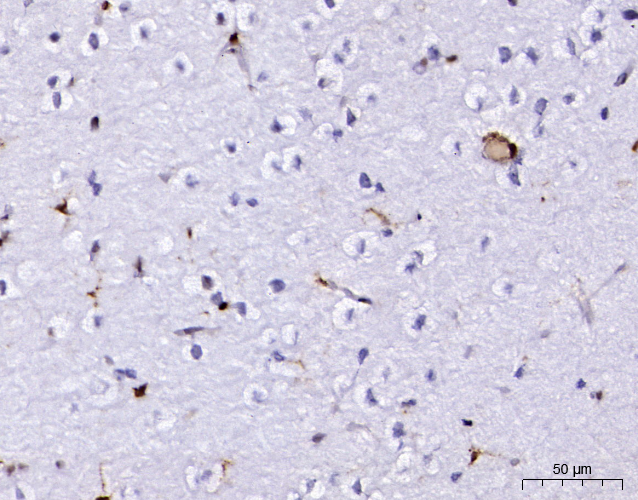

Supplement: Supplementary file 24 [file DataSheet7.ZIP › IHC Raw Image of P-CREB in PFC(2)/Z23 1-200 PCREB_20.0x.tif-Q5.tif]

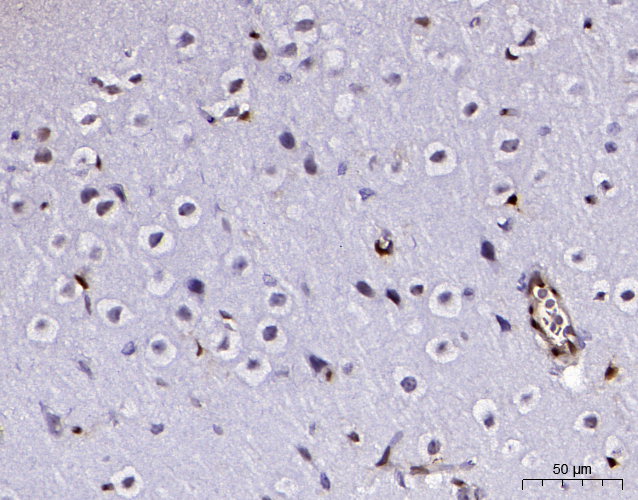

Supplement: Supplementary file 24 [file DataSheet7.ZIP › IHC Raw Image of P-CREB in PFC(2)/Z46 1-200 PCREB_20.0x.tif-Q1.tif]

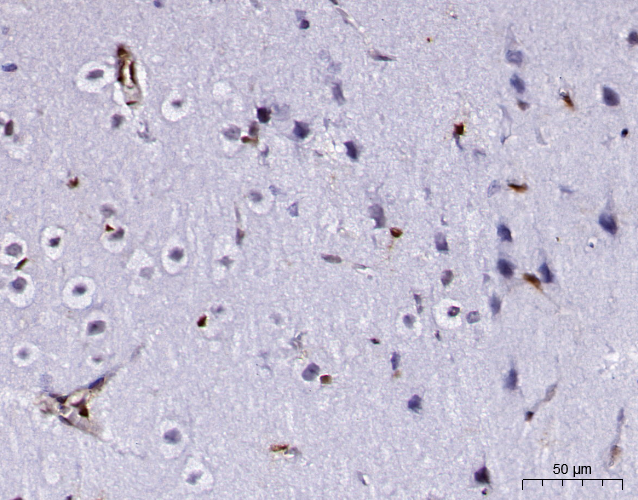

Supplement: Supplementary file 24 [file DataSheet7.ZIP › IHC Raw Image of P-CREB in PFC(2)/Z46 1-200 PCREB_20.0x.tif-Q2.tif]

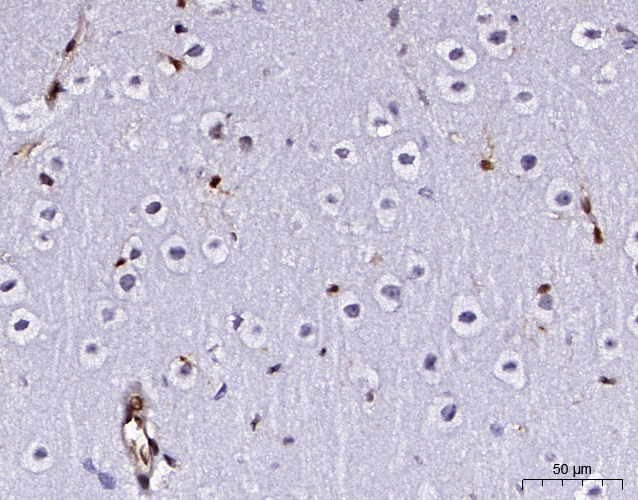

Supplement: Supplementary file 24 [file DataSheet7.ZIP › IHC Raw Image of P-CREB in PFC(2)/Z46 1-200 PCREB_20.0x.tif-Q3.tif]

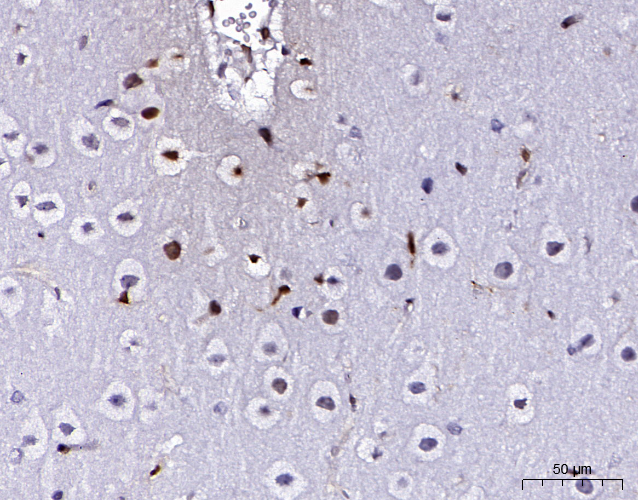

Supplement: Supplementary file 24 [file DataSheet7.ZIP › IHC Raw Image of P-CREB in PFC(2)/Z46 1-200 PCREB_20.0x.tif-Q4.tif]

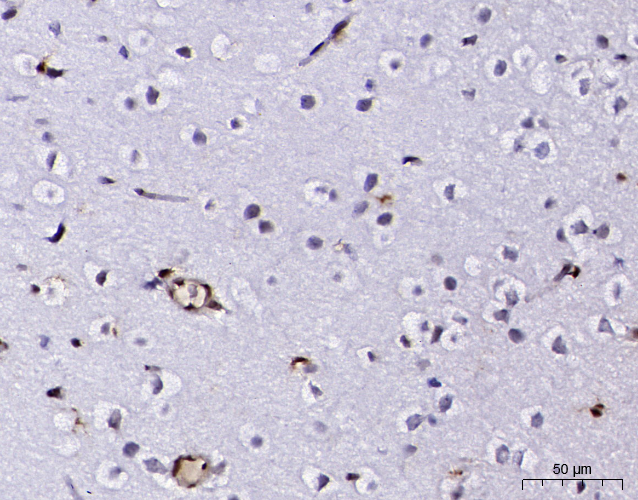

Supplement: Supplementary file 24 [file DataSheet7.ZIP › IHC Raw Image of P-CREB in PFC(2)/Z46 1-200 PCREB_20.0x.tif-Q5.tif]
